# Supplementary material for: Chalcogen bond-guided conformational isomerization enables catalytic dynamic kinetic resolution of sulfoxides
Source: Nat Commun. 2022 Aug 15;13:4793. doi: 10.1038/s41467-022-32428-4 (PMC9378665; doi:10.1038/s41467-022-32428-4)
Supplement: Supplementary file 1 — Supplementary Information [file 41467_2022_32428_MOESM1_ESM.pdf]

Supplementary Information

Chalcogen Bond-Guided Conformational Isomerization Enables Catalytic Dynamic Kinetic Resolution of Sulfoxides

Jianjian Liu[1], Mali Zhou[1], Rui Deng[1], Pengcheng Zheng[1]*, Yonggui Robin Chi[1,2]*

[1]State Key Laboratory Breeding Base of Green Pesticide and Agricultural Bioengineering, Key Laboratory of Green Pesticide and Agricultural Bioengineering, Ministry of Education, Guizhou University, Huaxi District, Guiyang 550025, China.

[2]Division of Chemistry & Biological Chemistry, School of Physical & Mathematical Sciences, Nanyang Technological University, Singapore 637371, Singapore.

*Corresponding authors e-mails:

pczheng@gzu.edu.cn.

robinchi@ntu.edu.sg.

Content

I. Supplementary Notes 3

General information 3

II. Supplementary Methods 4

Synthesis of substrates 4

Condition optimization 6

General procedure for the catalytic reactions 7

Synthetic transformations of **3a** and applications 8

III. Supplementary Discussion 14

Computational methods 15

Proposed mechanism 21

IV. Characterization of substrates and products 22

V. Supplementary Figures 40

^{1}H NMR, ^{13}C NMR and ^{19}F NMR spectra 40

HPLC and UPLC spectra 109

X-ray crystallography 145

VI. Supplementary References 148

I. Supplementary Notes

General information

Commercially available materials purchased from Energy Chemical were used as received. Unless otherwise specified, all reactions were prepared using 4.0 mL vial under N_2 atmosphere in glove-box from UNILAB SP. NMR spectra were recorded on a Brüker ASCEND 400 (400 MHz) spectrometer (^{1}H: 400 MHz, ^{13}C: 101 MHz, ^{19}F: 376 MHz). Chemical shifts (δ) for ^{1}H and ^{13}C NMR spectra are given in ppm relative to TMS. The residual solvent signals were used as references for ^{1}H and ^{13}C NMR spectra and the chemical shifts converted to the TMS scale ($CDCl_3$: δ H = 7.26 ppm, δ C = 77.16 ppm; DMSO-d_6: δ H = 2.50 ppm, δ C = 39.52 ppm). The following abbreviations were used to explain the multiplicities: s = singlet, d = doublet, t = triplet, q = quartet, m = multiplet, br = broad, and etc. All first-order splitting patterns were assigned on the base of the appearance of the multiplet. Splitting patterns that could not be easily interpreted are designated as multiplet (m) or broad (br). High resolution mass spectrometer analysis (HRMS) was performed on Thermo Fisher Q Exactive mass spectrometer. HPLC analyses were measured on Waters systems with Empower 3 system controller, Alliance column heater, and 2998 Diode Array Waters 2489 UV/Vis detector. Chiralcel brand chiral columns from Daicel Chemical Industries were used with models IA, IB, IG, IF, AD-H in 4.6 x 250 mm size. UPLC analyses were measured on Waters systems with Empower 3 system controller, Waters UPLC H-Class, and Waters ACQUITY UPLC PDA detector. Chiralcel brand chiral columns from Daicel Chemical Industries were used with models IA-U, IB-U, IC-U, or OD-3 in 3.0 x 100 mm size. All the crystals were determined by Xcalibur, Eos, Gemini diffractometer. Optical rotations were measured on an Insmark IP-digi Polarimeter in a 1 dm cuvette. The concentration (*c*) is given in g/100 mL. Melting point (m.p.): melting points were measured on a Beijing Tech Instrument X-4 digital display micro melting point apparatus and are uncorrected. Analytical thin-layer chromatography (TLC) was carried out on pre-coated silica gel plate (0.2 mm thickness). Visualization was performed using a UV lamp.

II. Supplementary Methods

Synthesis of substrates

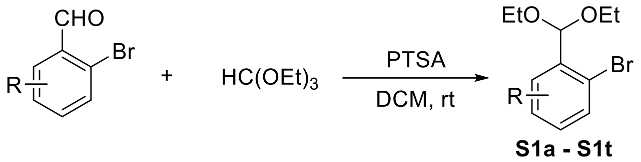

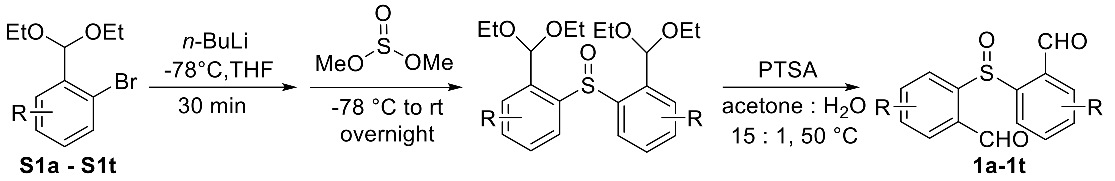

S1a-S1t were synthesized according to a modified procesure[1]. To a 100.0 mL round-bottomed flask was charged with 2-Bromobenzaldehydes (5.0 g, 27.0 mmol) and 50.0 mL dry CH_2Cl_2 and placed at 0°C, Triethyl orthoformate (9.0 mL, 54.0 mmol) was added, *p*-tolylsulfonicacid (232.5 mg, 1.35 mmol) was then added slowly at same temperature and stirred for 10 min, the mixture was warmed to room temperature and stirred for another 2 h. The mixture was concentrated under reduced pressure. The resulting crude residue was purified *via* column chromatography on silica gel by using petroleum ether / ethyl acetate to afford the desired products **S1a-S1t** with 95% yield.

To a 100.0 mL Schlenk flask was charged with **S1a** (5.2 g, 20.0 mmol) and 50.0 mL dry THF and placed at -78 °C, *n*-BuLi (2.5 M in hexane, 8.8 mL, 22.0 mmol) was added dropwise at same temperature and stirred for 30 min. Dimethyl sulfite (0.94 mL, 11.0 mmol) was added dropwise over 10 min and stirred for additional 30 min, the mixture was warmed to room temperature and stirred overnight. The reaction mixture was quenched with saturated aqueous ammonium chloride solution and extracted using ethyl acetate. The organic extracts were combined and dried over with anhydrous sodium sulphate and concentrated in vacuo, the residual viscous crude product was dissolved in acetone / water (30.0 mL, 15:1) mixture in a round bottom flask, PTSA (0.02 mmol) was added and stirred at 50°C and monitored by TLC. The reaction mixture was quenched with saturated aqueous sodium bicarbonate solution and extracted with ethyl acetate. The organic extracts were combined, dried over anhydrous

sodium sulphate and concentrated. The crude product was purified by silica gel column chromatography using petroleum ether/ethyl acetate as eluent to afford compounds **1a** to **1t**.

Substrates **1aa** and **1ab** was synthesized in the same method with corresponding bromobenzaldehydes.

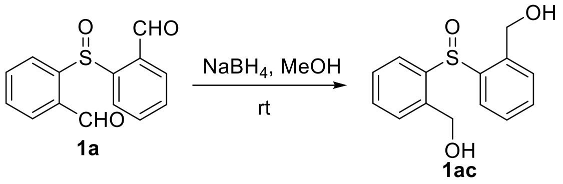

A reported molecule[2] **1ac** was synthesized from **1a**. To a solution of **1a** (0.2 mmol, 51.6 mg) in 3.0 mL MeOH, $NaBH_4$ (0.5 mmol, 18.9 mg) was added slowly, the reaction mixture was stirred at room temperature for 30 min. The reaction mixture was quenched with saturated aqueous ammonium chloride solution and extracted using ethyl acetate. The organic extracts were combined and dried over with anhydrous sodium sulphate then concentrated in vacuo. The crude product was purified by silica gel column chromatography using CH_2Cl_2 / MeOH as eluent to afford compound **1ac** (49.8 mg, 95%).

Condition optimization

Supplementary Table 1. Condition optimization for the synthesis of **3a**

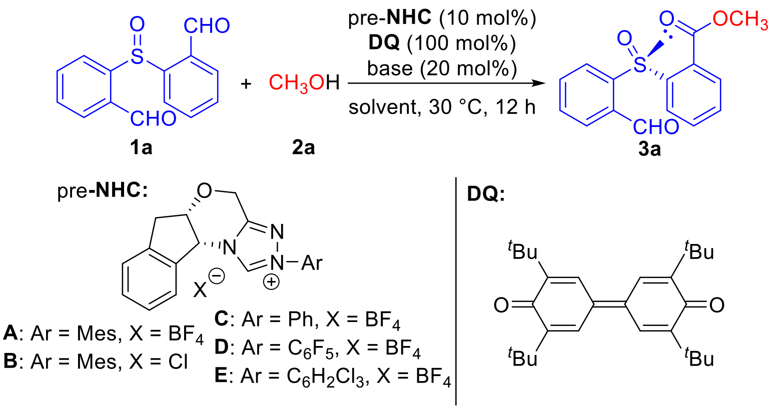

entry[a]	NHC	base	solvent	yield[b]	er[c]
1	A	K_2CO_3	THF	45%	99:1
2	B	K_2CO_3	THF	52%	99:1
3	C	K_2CO_3	THF	55%	98:2
4	D	K_2CO_3	THF	44%	92:8
5	E	K_2CO_3	THF	trace	--
6	B	Na_2CO_3	THF	56%	98:2
7	B	Cs_2CO_3	THF	35%	99:1
8	B	K_3PO_4	THF	85%	97:3
9	B	KOAc	THF	58%	99:1
10	B	NaOAc	THF	72%	98:2
11	B	DBU	THF	76%	94:6
12	B	Et_3N	THF	82%	96:4
13	B	K_3PO_4	CH_2Cl_2	89%	> 99:1
14	B	K_3PO_4	EtOAc	75%	98:2
15	B	K_3PO_4	1,4-Dioxane	80%	98:2
16	B	K_3PO_4	Toluene	50%	99:1

[a]Unless otherwise specified, the reactions were carried under N_2 atmosphere using **1a** (0.10 mmol), **DQ** (0.10 mmol), CH_3OH (0.12 mmol), **NHC** (0.01 mmol), base (0.02 mmol), solvent (2.0 mL), 30°C, 12 h. [b]Isolated yield of **3a**. [c]The er values of **3a** were determined via HPLC on the chiral stationary phase.

General procedure for the catalytic reactions

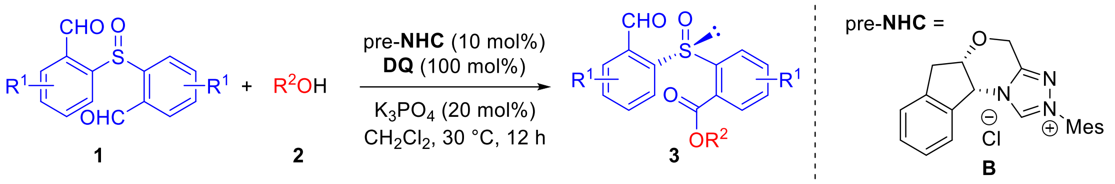

To a 4.0 mL over-dried vial equipped with a magnetic stir bar was added **1** (0.1 mmol), DQ (0.1 mmol), pre-NHC **B** (0.01 mmol) and K_3PO_4 (0.02 mmol). The vial was then sealed, purged and backfilled with N_2 three times in glovebox before adding CH_2Cl_2 (2.0 mL) and nucleophiles **2** (0.12 mmol), and the reaction mixture was stirred in oil bath at 30 °C for 12 h. The mixture was concentrated under reduced pressure. The resulting crude residue was purified *via* column chromatography on silica gel by using petroleum ether / ethyl acetate to afford the desired products **3**.

Gram scale synthesis of 3a

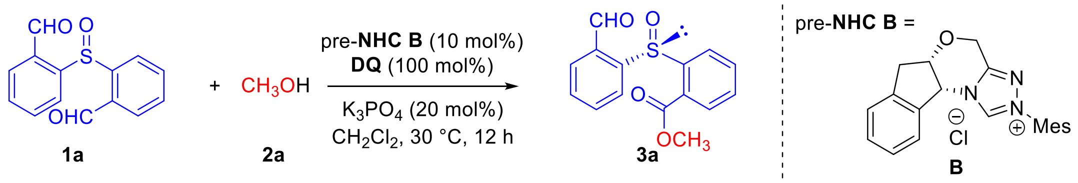

To a 100.0 mL over-dried round bottom flask equipped with a magnetic stir bar was added **1a** (1.0 g, 3.87 mmol), **DQ** (1.58g, 3.87 mmol), pre-**NHC B** (139.8 mg, 0.38 mmol) and K_3PO_4 (164.2 mg, 0.77 mmol). The flask was then sealed, purged and backfilled with N_2 three times in glovebox before adding CH_2Cl_2 (60.0 mL) and CH_3OH (0.19 mL, 4.65 mmol), and the reaction mixture was stirred in oil bath at 30 °C for 12 h. The mixture was concentrated under reduced pressure. The resulting crude residue was purified *via* column chromatography on silica gel by using petroleum ether / ethyl acetate (2:1) to afford the desired product **3a** (881.8 mg, 79% yield, > 99:1 er).

Synthetic transformations of 3a and applications

General procedure for the synthesis of (4a)[3]

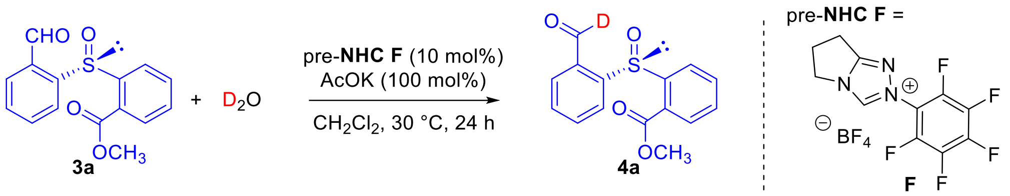

3a (0.1 mmol, 28.8 mg), pre-NHC **F** (0.01 mol, 3.6 mg) and AcOK (0.1 mmol, 9.8 mg) was dissolved in a mixture of D_2O (1.0 mL) and CH_2Cl_2 (0.25 mL) in a reaction vessel (4.0 mL). Then the reaction mixture was vigorously stirred at 30 °C for 24 hours. The reaction mixture was extracted with ethyl acetate and the organic layer was combined, dried over with anhydrous sodium sulphate and concentrated in vacuo, the residual was purified by silica gel column chromatography using petroleum ether / ethyl acetate (2:1) as eluent to afford compound **4a** in 77% yield with 100% D-incorporation (22.2 mg, > 99:1 er).

General procedure for the synthesis of (4b)[4]

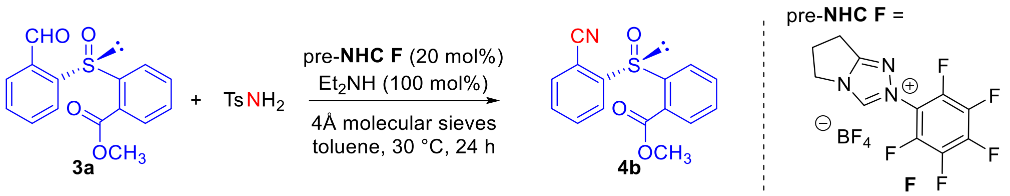

To a 4.0 mL over-dried vial equipped with a magnetic stir bar was added pre-NHC **F** (0.02 mmol, 7.2 mg), 4 Å molecular sieves (50.0 mg), substrate **3a** (0.10 mmol, 28.8 mg) and $TsNH_2$ (0.11 mmol, 18.8 mg). Then dried toluene (2.0 mL) and Et_2NH (0.10 mmol, 10.3 μL) was added via syringe in a glove box under N_2 atmosphere. Then the reaction mixture was stirred for 24 hours at 30 °C and then subjected to column chromatography on silica gel (2:1 petroleum ether / ethyl acetate) directly to give the desired pure product **4b** in 69% yield (19.6 mg, 98:2 er).

General procedure for the synthesis of (4c)

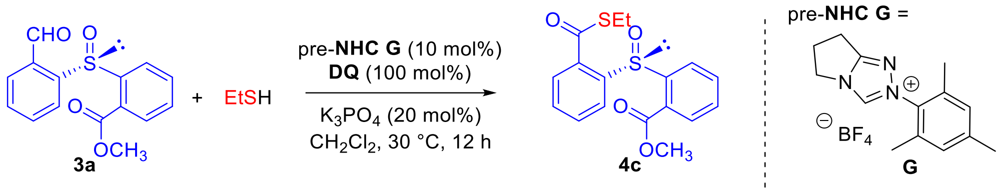

To a 4.0 mL over-dried vial equipped with a magnetic stir bar was added **3a** (0.1 mmol, 28.8 mg), **DQ** (0.1 mmol, 40.8 mg), pre-NHC **G** (0.01 mmol, 3.2 mg) and K_3PO_4 (0.02 mmol, 4.3 mg). The vial was then sealed, purged and backfilled with N_2 three times in glovebox before adding CH_2Cl_2 (2.0 mL) and EtSH (0.12 mmol, 8.4 μL), and the reaction mixture was stirred in oil bath at 30 °C for 12 h. The mixture was concentrated under reduced pressure. The resulting crude residue was purified *via* column chromatography on silica gel by using petroleum ether / ethyl acetate (2:1) to afford the desired product **4c** in 92% yield (32.0 mg, > 99:1 er).

General procedure for the synthesis of (4d)[5]

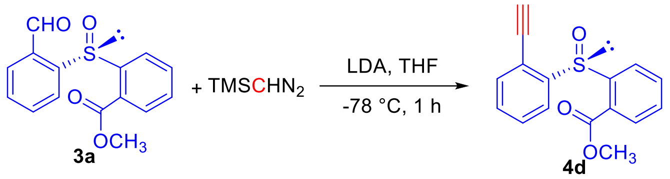

To a dried 10.0 mL Schlenk tube equipped with a magnetic stir bar and $TMSCHN_2$ (0.12 mmol, 17.8 μL) was dissolved in dry tetrahydrofuran (1.0 mL) under nitrogen at -78 °C. LDA (2.0 M in THF, 0.13 mmol, 65.0 μL) was added and the mixture was stirred at -78 °C for 30 min. **3a** (0.1 mmol, 28.8 mg) was dissolved in tetrahydrofuran (1.0 ml) and added dropwise at -78 °C. The mixture was stirred at -78 °C for 1 h and then warm to 0 ºC for 1 h. The reaction was monitored by TLC. Upon completion, water (1.0 mL) was added to quench the reaction and the mixture was stirred at 0 ºC for 5 min. The mixture was extracted with ethyl acetate (3 x 5 mL). The organic layers were dried over Na_2SO_4, filtered and concentrated under vacuum. The residue was purified *via* silica gel column chromatography by using petroleum ether / ethyl acetate (2:1) to afford the desired product **4d** in 57% yield (16.2 mg, > 99:1 er).

General procedure for the synthesis of 4e and 4f

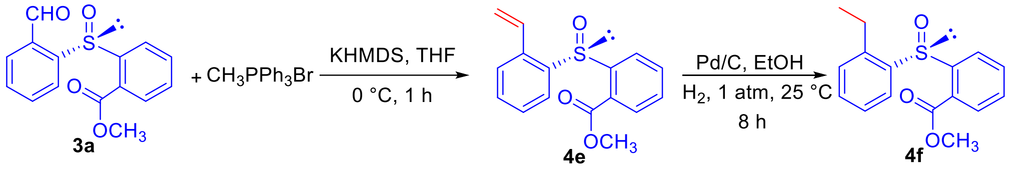

To a solution of Methyltriphenylphosphonium bromide (0.12 mmol, 42.9 mg) in dry THF (2.0 mL) was added KHMDS (1 M solution in THF, 0.12 mmol, 0.12 mL) at 0°C under argon. The mixture was stirred at 0 °C for 30 min, **3a** (0.1 mmol, 28.8 mg) was dissolved in tetrahydrofuran (1.0 mL) and added dropwise at 0 °C. The mixture was stirred at 0 °C for 1 h. The mixture was concentrated under reduced pressure. The resulting crude residue was purified *via* column chromatography on silica gel by using petroleum ether / ethyl acetate (5:1) to afford the desired product **4e** in 80% yield (22.9 mg, > 99:1 er).

To a 10.0 mL Schlenk tube, a mixture of compound **4e** (28.6 mg, 0.1 mmol) and 10% Pd/C (2.9 mg) in EtOH (2.0 mL) was flushed with hydrogen gas, the mixture was stirred at 25 °C under a balloon of hydrogen. After 8 h, the mixture was filtered through Celite and concentrated, purified *via* silica gel column chromatography by using petroleum ether / ethyl acetate (5:1) to afford the product **4f** in 94% yield (27.1 mg, 97:3 er).

General procedure for the synthesis of (4g)[6]

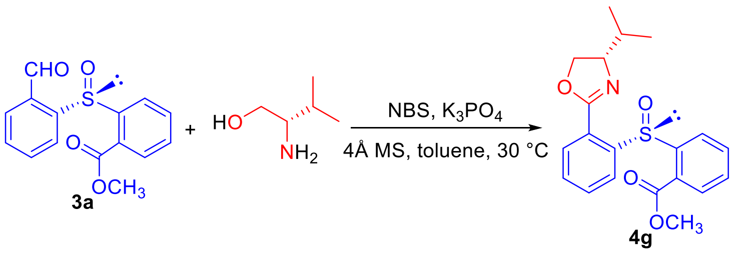

The L-valinol (0.5 mmol, 51.6 mg) was dissolved in toluene (5.0 mL) and the **3a** (0.5 mmol, 144.0 mg) was added. The mixture was stirred over 4 Å molecular sieve (0.75 g) at 30 °C for 12 h. K_3PO_4 was then added while stirring. After 5 min, NBS (0.5 mmol, 89.0 mg) was added and stirred for an additional 1.5 h. The mixture was filtered

and extracted with ethyl acetate, washed with sat. aq $NaHCO_3$ (10.0 mL) and H_2O (10.0 mL). The organic layer was dried (Na_2SO_4) and the solvent was evaporated, the products were purified *via* column chromatography by using petroleum ether / ethyl acetate (2:1) to afford the target product **4g** in 71% yield (131.8 mg).

General procedure for the synthesis of (4h)

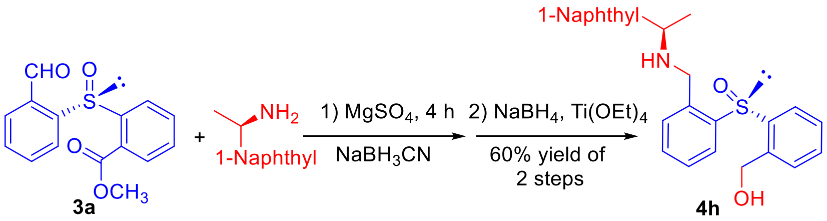

The (*R*)-1-(1-naphthyl)ethylamine (0.1 mmol, 17.1 mg) was dissolved in CH_2Cl_2 (2.0 mL) and the **3a** (0.1 mmol, 28.8 mg) was added. The mixture was stirred over $MgSO_4$ (50.0 mg) at 30 °C for 4 h. $NaBH_3CN$ was then added while stirring. After 2 h, the mixture was filtered and concentrated in vacuo. The crude product was dissolved in CH_2Cl_2 (2.0 mL), $Ti(OEt)_4$ (0.2 mmol, 42.0 μL) and $NaBH_4$ (0.2 mmol, 7.6 mg) were added. The mixture was stirred at 30 °C for 2 h, then quenched with sat. aq $NaHCO_3$, extracted with ethyl acetate and washed with sat. aq NaCl. The organic layer was dried (Na_2SO_4) and the solvent was evaporated, the products were purified via column chromatography by using petroleum ether / ethyl acetate (1:1) to afford **4h** in 60% yield (25.0 mg) over two steps.

General procedure for the synthesis of (4i)

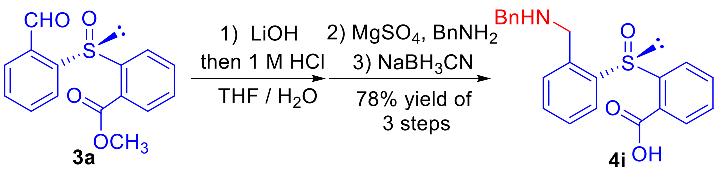

To a solution of **3a** (0.1 mmol, 28.8 mg) in 2.0 mL THF-H_2O (2:1) was added LiOH (0.15 mmol, 3.6 mg), the reaction mixture was stirred at 25 °C for 2 h, 1 M HCl was added slowly and acidified to pH 6. The aqueous phase was extracted with CH_2Cl_2 (three times), the combined organic layer was dried over with Na_2SO_4 and concentrated, the residue was dissolved in CH_2Cl_2 (2.0 mL) and the $BnNH_2$ (0.11 mmol, 12.0 μL)

was added. The mixture was stirred over $MgSO_4$ (50.0 mg) at 30 °C for 4 h. $NaBH_3CN$ was then added while stirring. After 3 h, the mixture was filtered and concentrated in vacuo, the crude product was purified via column chromatography by using CH_2Cl_2 / MeOH (20:1) to afford **4i** in 78% yield (28.5 mg, > 99:1 er) over three steps.

General procedure for the synthesis of (4j)[7]

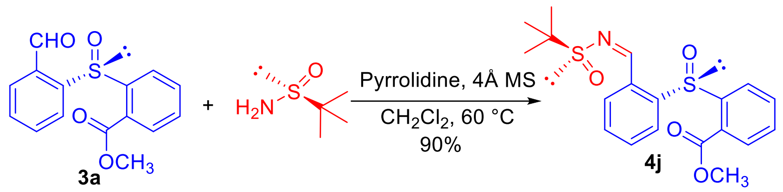

To a 0.33 M solution of (*S*)-*tert*-Butanesulfinamide (0.11 mmol, 13.3 mg) in dichloromethane and 4Å molecular sieves (100 mg), aldehyde **3a** (0.1 mmol, 28.8 mg) and 10 mol% of pyrrolidine were added. The mixture was stirred in a sealed vial at 60 ºC for 4 h. Then, the reaction mixture was filtered and concentrated in vacuo, the crude product was purified via column chromatography by using petroleum ether / ethyl acetate (2:1) to afford **4j** in 90% yield (35.2 mg).

General procedure for the synthesis of (5a)[8]

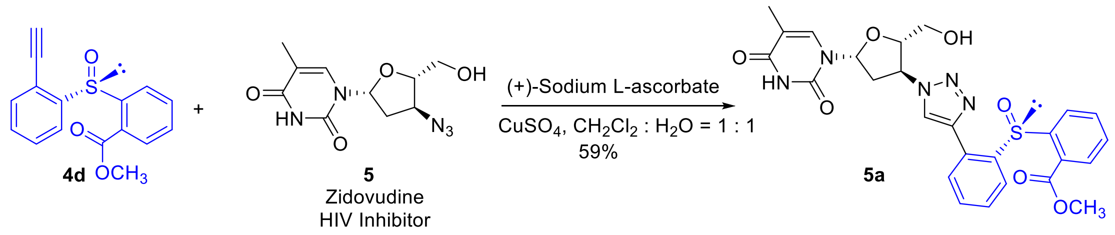

To a solution of **5** (0.1 mmol, 26.7 mg) and **4d** (0.1 mmol, 28.4 mg) in CH_2Cl_2 / H_2O (v / v=1 / 1, 2.0 mL) was added Sodium L-ascorbate (0.05 mmol, 9.9 mg) and $CuSO_4$ (0.03 mmol, 4.8 mg) at room temperature, and stirred for 12 hours. Then the solution was participated and the aqueous phase was extracted with CH_2Cl_2, the combined organic layer was dried with Na_2SO_4 and concentrated in vacuum to give the crude product. The crude product was purified by column chromatography on silica gel with petroleum ethyl / ethyl acetate (1:1) to afford the desired **5a** in 69% yield (38.0 mg).

General procedure for the synthesis of (8)[9]

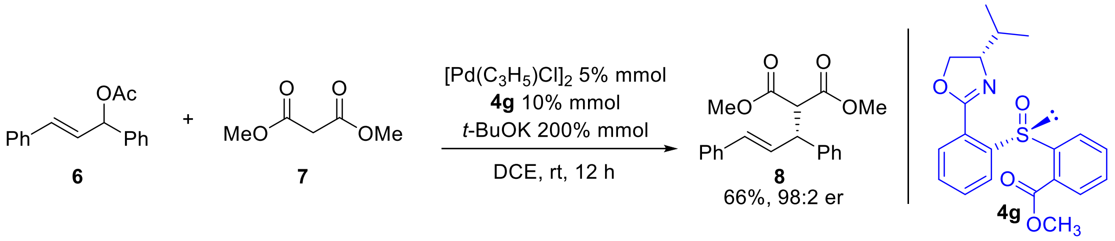

To a 4.0 mL over-dried vial equipped with a magnetic stir bar was added chiral ligand **4g** (0.01 mmol, 3.7 mg), $[Pd(C_3H_5)Cl]_2$ (0.005 mmol, 1.8 mg) and *t*-BuOK (0.2 mmol, 22.4 mg) in a glove box under N_2 atmosphere. The solution of the substrates **6** (0.15 mmol, 37.9 mg) and **7** (0.10 mmol, 13.2 mg) in dried DCE (2.0 mL) was added *via* syringe. Then the reaction mixture was stirred at 25 °C for 12 h and then subjected to column chromatography on silica gel (20:1 petroleum ether / ethyl acetate) to give the product **8** in 66% yield (21.4 mg, 98:2 er).

III. Supplementary Discussion

Supplementary Table 2. X-ray analysis data of compounds **1a** and **1aa**.

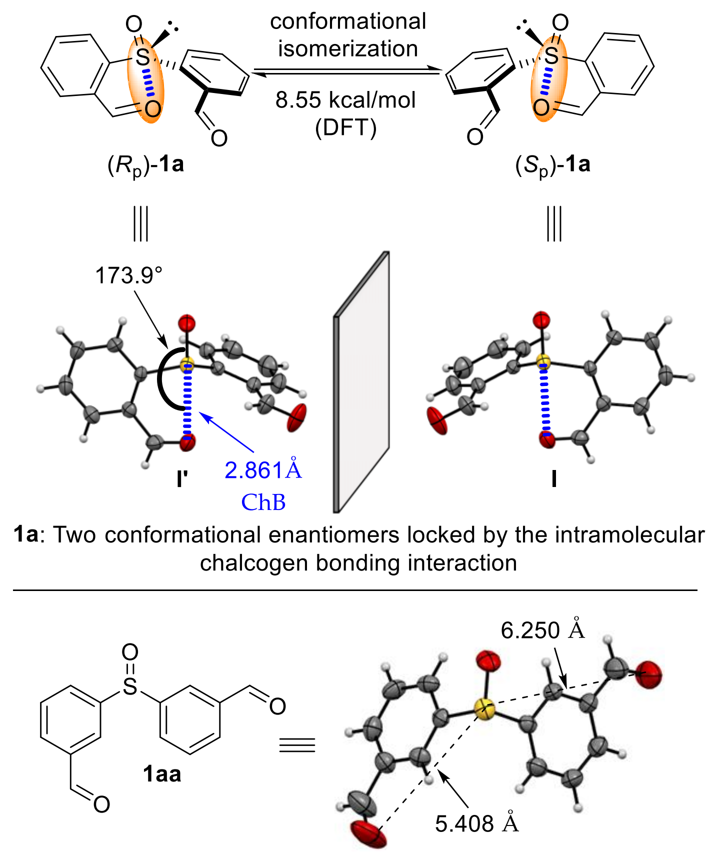

1a: Two conformational enantiomers locked by the intramolecular chalcogen bonding interaction

1aa: Without chalcogen bonding interaction

By analyzing the single crystal data of **1a**, we found that the exist of two conformations in unit cell, and the distance between the O atom of the formyl and the S atom of the sulfoxide is 2.861 Å, which is less than the sum of the van der Waals radii of O and S atoms (3.32 Å), and the angle between O-S-O is 173.9°, so we determined that there is an intramolecular chalcogen bonding interaction, and this effect leads to conformational locking and breaks the symmetry of **1a** and makes the entire molecule racemic. Thus, the two preferred conformations (also are enantiomers) can be crystallized. And **1aa** does not meet the conditions for the formation of chalcogen bond.

Computational methods

The initial cluster structures were optimized using global hybrid functional M06-2X with Karlsruhe-family double-ζ valence def2-SVP basis set for all atoms as implemented in Gaussian 16 (rev. A.03)[10]. Single point (SP) corrections were performed using M06-2X functional and def2-TZVPP basis set for all atoms. Minima and transition structures on the potential energy surface (PES) were confirmed as such by harmonic frequency analysis, showing respectively zero and one imaginary frequency. The implicit IEFPCM continuum solvation model for CH_2Cl_2 solvent was used to account for the effect of solvent on the potential energy surface. Gibbs energies were evaluated at 30 °C, which was used in the experiments, using a quasi-RRHO treatment of vibrational entropies. Vibrational entropies of frequencies below 100 cm^{-1} were obtained according to a free rotor description, using a smooth damping function to interpolate between the two limiting descriptions[11]. The three-dimensional structures were illustrated by using CYLview20[12]. In addition, the enantiomeric excess (ee) value, was calculated by using the Boltzmann distribution of diastereomeric transition states with the following equations[13]:

$$\frac{[R]}{[S]} = \frac{\exp\left(-\Delta G_{[R]}/RT\right)}{\exp\left(-\Delta G_{[S]}/RT\right)} = \exp\left(\frac{\Delta\Delta G_{[S]-[R]}}{RT}\right) \qquad (1)$$

$$ee = \frac{[R]-[S]}{[R]+[S]} \times 100\% = \frac{\frac{[R]}{[S]}-1}{\frac{[R]}{[S]}+1} \times 100\% \qquad (2)$$

where ΔG is the Gibbs free energy barrier of the diastereomeric transition state and ΔΔG is the difference between the Gibbs free energy barriers of two competing diastereomeric transition states.

Influence of substituents on benzene ring to the strength of chalcogen bond

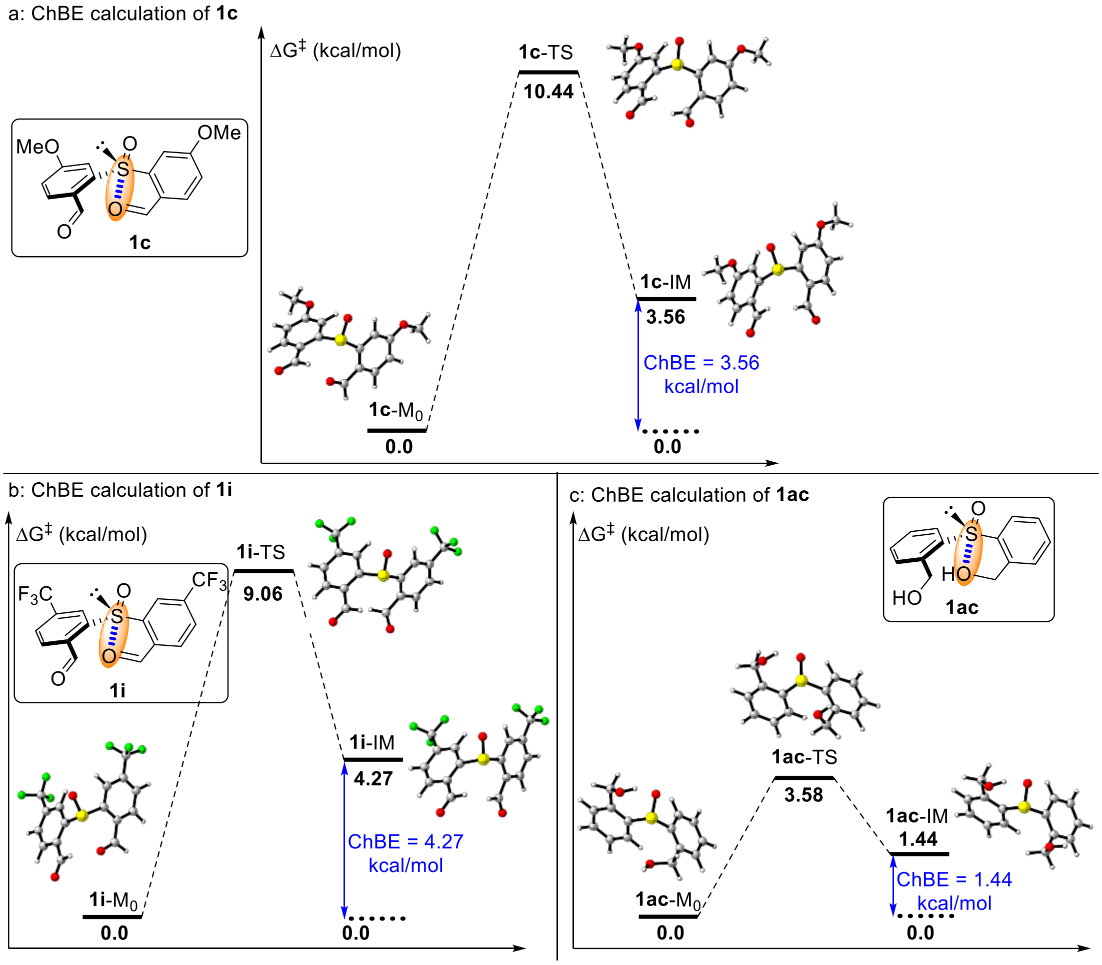

Supplementary Figure 1. The effect of different substituents and Lewis bases to chalcogen bonds

For the sake of evaluating the influence of substituents on benzene ring to the chalcogen bond energy (ChBE), two substrates bearing electron donating group (OMe, **1c**) and electron withdrawing group (CF_3, **1i**) on the *para*-position (relative to the aldehyde moiety) were picked out to process the DFT calculation. The rotation energy profile of the chalcogen bonded formyl group were listed in supplementary figure 1. Comparing to the model substrate **1a** (ChBE = 3.44 kcal/mol), the result suggested that introducing methyloxyl would increase the Lewis basicity of aldehyde oxygen atom therefore enhanced chalcogen bond energy slightly (3.56 kcal/mol, Supplementary Figure 1a). In addition, when the CF_3 group was installed on the *para*-position of formyl group, the ChBE was raised to 4.27 kcal/mol, it may due to the deeper σ-hole of sulfoxide sulfur atom (Supplementary Figure 1b). At the same time, in order to

investigate the effect of different Lewis base, diol substrate **1ac** was synthesized. Unfortunately, the chalcogen bond was weakened, and the ChBE was 1.44 kcal/mol (Supplementary Figure 1c).

DFT Calculations of Regio-selectivity of NHC to aldehyde motifs

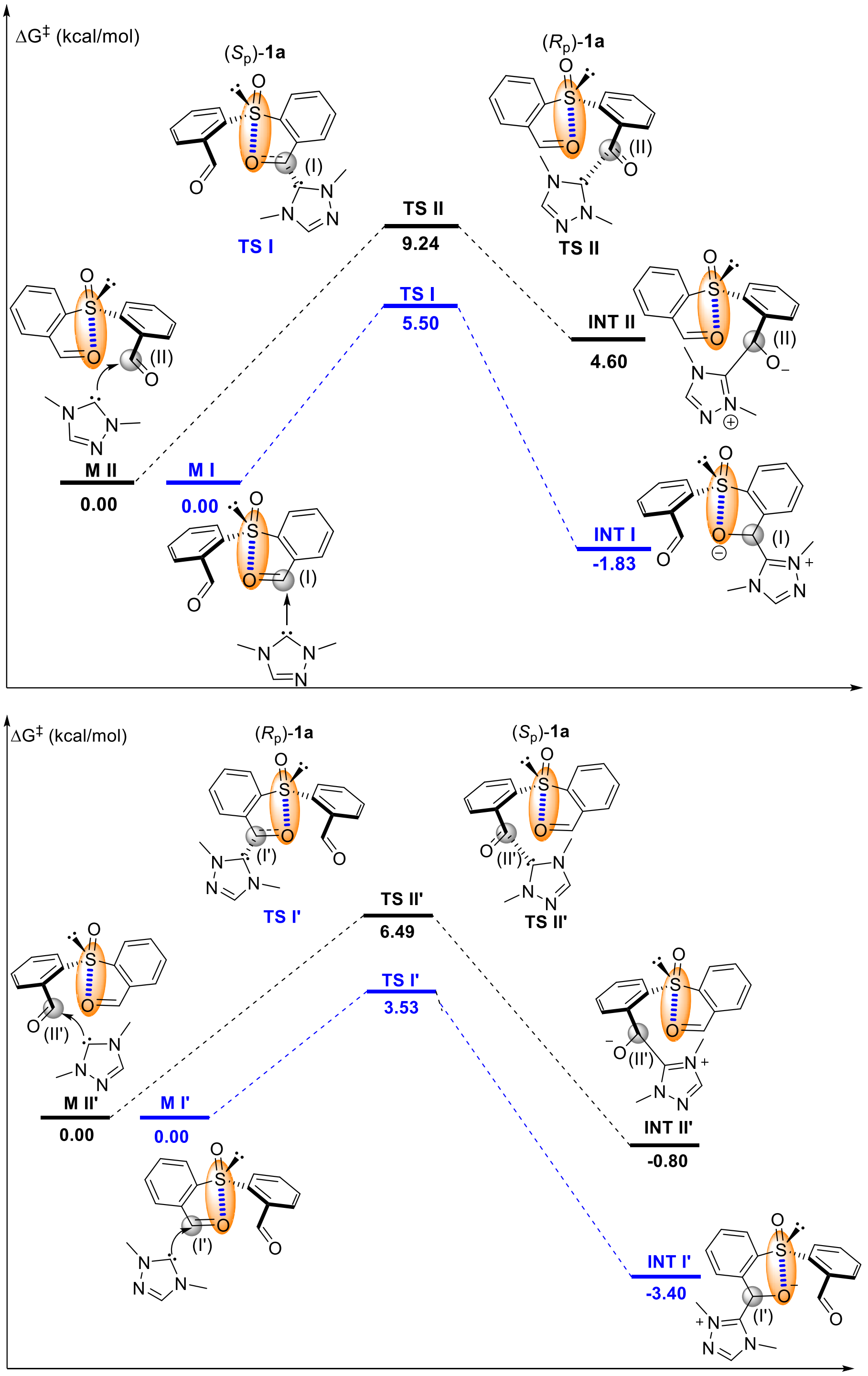

Supplementary Figure 2. Gibbs energy profile of NHC addition to formyl groups.

Based on the single crystal diffraction of **1a**, the aldehyde groups exist in four chemical environments. Subsequently, we evaluated the barrier of NHC addition to the four type formyl groups *via* DFT calculation. The Gibbs energy profile show that non-chalcogen bonded formyl groups (position II and II′) with higher energy barrier when NHC addition to it (the energy barrier of **TS II** and **TS II′** are 9.24 kcal/mol, 6.49 kcal/mol, respectively). However, the addition process of NHC to chalcogen bonded formyl groups (position **I** and **I′**) were easier with the barrier of 5.50 kcal/mol and 3.53 kcal/mol, respectively, it means that the formyl group on the position of **I** and **I′** were activated by chalcogen bond (Supplementary Figure 2). The optimized structures were listed in Table 3.

Supplementary Table 3: Optimized structure of NHC addition to formyl groups.

	**TS I**	**TS II**
**Optimized structure**	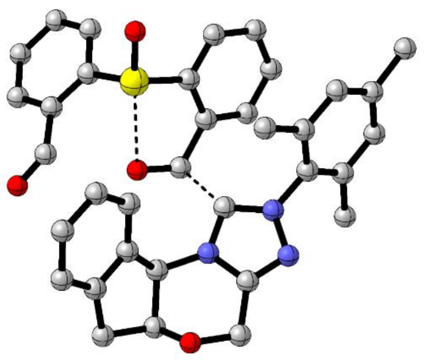 **$\Delta G^{\ddagger}$ = 5.50 kcal/mol**	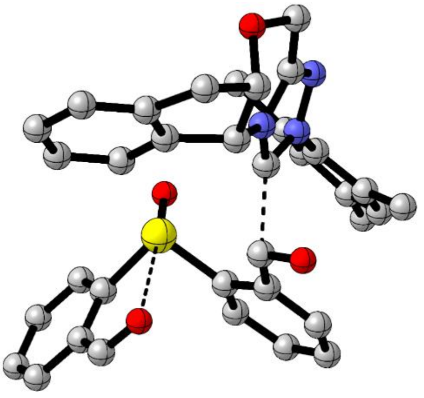 **$\Delta G^{\ddagger}$ = 9.24 kcal/mol**
	**TS I′**	**TS II′**
	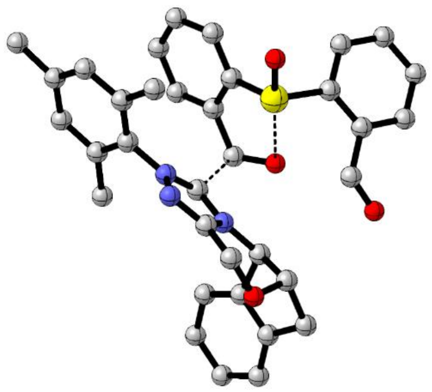 **$\Delta G^{\ddagger}$ = 3.53 kcal/mol**	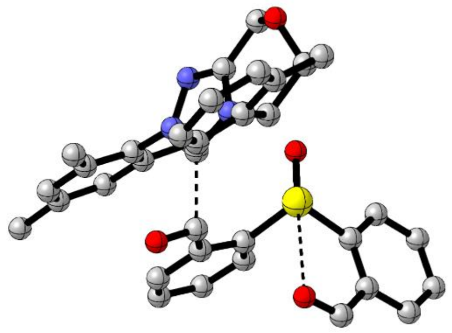 **$\Delta G^{\ddagger}$ = 6.49 kcal/mol**

DFT calculation of oxidational process

The addition energy barrier calculation of **NHC** to the formyl group disclosed that the chalcogen bonded aldehyde groups have preferential reactivity with **NHC**. And the oxidation barriers of Breslow intermedates were evalued subsequently *via* DFT calculation. Chalcogen bonded formyl group of (S_p)-**1a** generated Breslow intermediate with **NHC** has lower oxidation energy barrier than the Breslow intermediate of (R_p)-**1a**. The energy barrier is 14.16 kcal/mol and 22.22 kcal/mol, respectively (**Ox-Ts I** and **Ox-Ts I′**). The $\Delta\Delta G^{\ddagger}$ of 8.06 kcal/mol favors the observed enantioselectivity ratio by 6.38 x 10^5:1 and translates to an ee value of up to 99.9% (calculated by equation 1 and 2). The oxidational process and optimized structures were listed in supplementary figure 3 and Table 4 respectively.

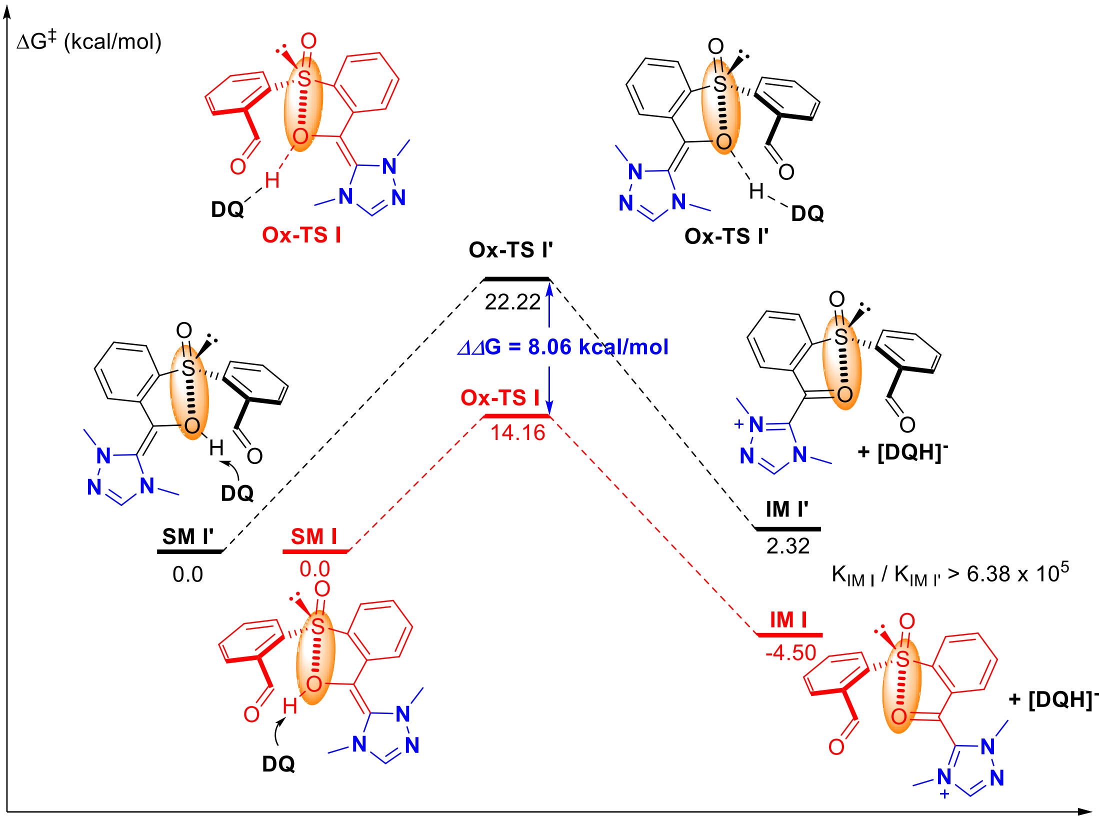

Supplementary Figure 3. Oxidized barrier of Breslow intermedates

Supplementary Table 4 : Optimized structure of oxidation process.

	Optimized structure
**Major: Ox-TS I**	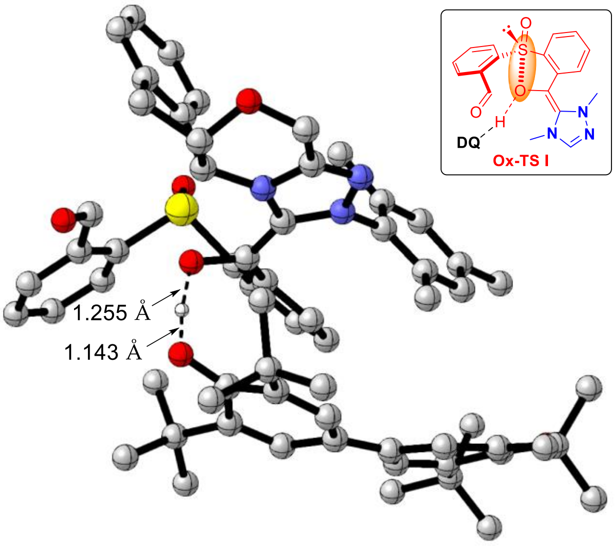 **ΔG‡ = 14.16 kcal/mol**
**Minor: Ox-TS I′**	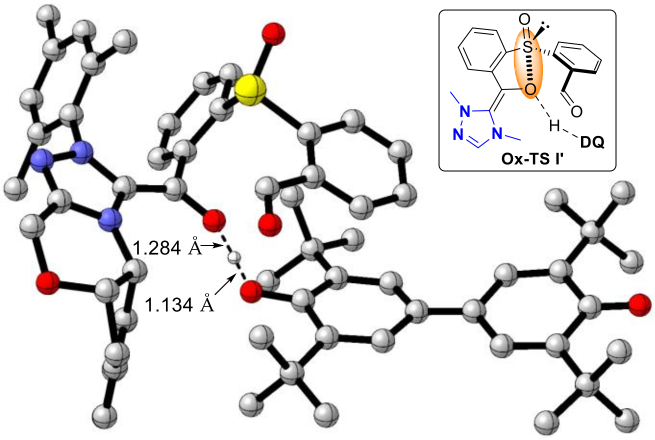 **ΔG‡ = 22.22 kcal/mol**

Proposed mechanism.

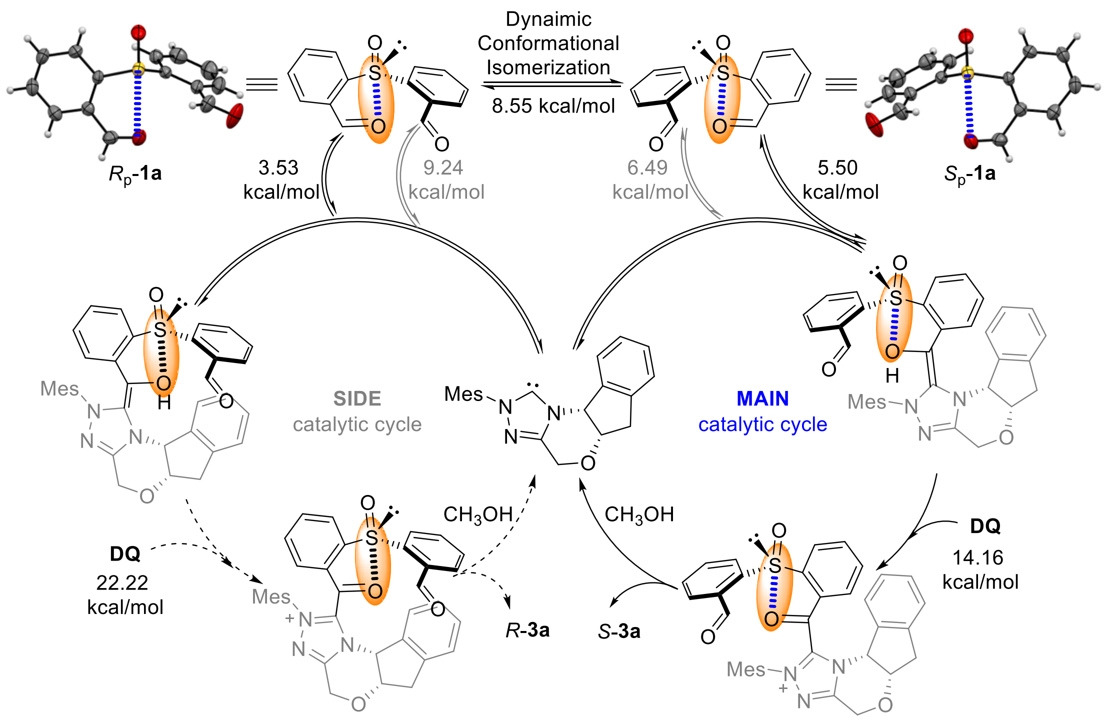

IV. Characterization of substrates and products

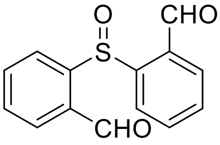

1a

2,2'-Sulfinyldibenzaldehyde, White Solid, 60% yield, m.p. 127.6 – 128.0 °C.

^{1}H NMR (400 MHz, CDCl$_3$) δ 10.50 (s, 2H), 8.00 (dd, *J* = 7.8, 1.2 Hz, 2H), 7.97 (dd, *J* = 7.6, 1.5 Hz, 2H), 7.78 (td, *J* = 7.6, 1.5 Hz, 2H), 7.65 (td, *J* = 7.5, 1.2 Hz, 2H).

^{13}C NMR (101 MHz, CDCl$_3$) δ 190.3, 147.2, 135.0, 133.6, 131.6, 131.4, 126.5.

HRMS (ESI, m/z): Mass calcd. for $C_{14}H_{11}O_3S^+$ $[M+H]^+$, 259.0423; found 259.0424.

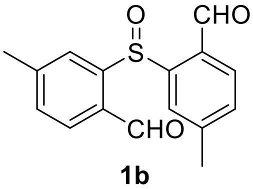

1b

2,2'-sulfinylbis(4-methylbenzaldehyde)，White Solid, 66% yield, m.p. 150.0 – 151.2 °C.

^{1}H NMR (400 MHz, CDCl$_3$) δ 10.44 (s, 2H), 7.86 (d, *J* = 7.8 Hz, 2H), 7.76 (s, 2H), 7.43 (d, *J* = 7.7 Hz, 2H), 2.48 (s, 6H).

^{13}C NMR (101 MHz, CDCl$_3$) δ 189.9, 146.9, 146.6, 132.1, 131.7, 131.5, 126.7, 22.2.

HRMS (ESI, m/z): Mass calcd. for $C_{16}H_{14}NaO_3S^+$ $[M+Na]^+$, 309.0556; found 309.0553.

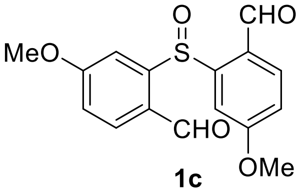

1c

2,2'-Sulfinylbis(4-methoxybenzaldehyde), White Solid, 57% yield, m.p. 148.2 – 148.9 °C.

^{1}H NMR (400 MHz, CDCl$_3$) δ 10.32 (s, 2H), 7.91 (d, *J* = 8.5 Hz, 2H), 7.44 (d, *J* = 2.5 Hz, 2H), 7.07 (dd, *J* = 8.5, 2.5 Hz, 2H), 3.89 (s, 6H).

^{13}C NMR (101 MHz, CDCl$_3$) δ 188.8, 165.0, 149.4, 133.9, 127.0, 116.3, 112.0, 56.2.

HRMS (ESI, m/z): Mass calcd. for $C_{16}H_{14}NaO_5S^+$ $[M+Na]^+$, 341.0454; found 341.0452.

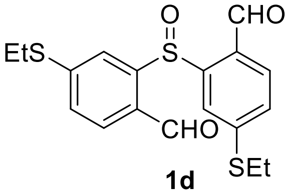

1d

2,2'-sulfinylbis(4-(ethylthio)benzaldehyde), White Solid, 42% yield, m.p. 141.6 – 142.0 °C.

^{1}H NMR (400 MHz, DMSO-*d*$_6$) δ 10.27 (s, 2H), 7.96 (d, *J* = 8.0 Hz, 2H), 7.62 – 7.59 (m, 4H), 3.16 – 3.02 (m, 4H), 1.21 (t, *J* = 7.3 Hz, 6H).

^{13}C NMR (101 MHz, DMSO-*d*$_6$) δ 190.5, 147.4, 146.9, 132.5, 129.4, 128.0, 121.8, 24.8, 13.5.

HRMS (ESI, m/z): Mass calcd. for $C_{18}H_{18}NaO_3S_3^+$ $[M+Na]^+$, 401.0310; found 401.0306.

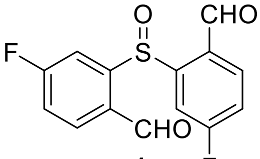

1e

2,2'-sulfinylbis(4-fluorobenzaldehyde), White Solid, 54% yield, m.p. 156.3 – 156.6 °C.

^{1}H NMR (400 MHz, CDCl$_3$) δ 10.42 (s, 2H), 8.03 (dd, *J* = 8.5, 5.2 Hz, 2H), 7.68 (dd, *J* = 8.2, 2.5 Hz, 2H), 7.34 (ddd, *J* = 8.4, 7.6, 2.5 Hz, 2H).

^{19}F NMR (376 MHz, CDCl$_3$) δ -98.6.

^{13}C NMR (101 MHz, CDCl$_3$) δ 188.7 , 166.8 (d, *J* = 262.5 Hz), 150.2 (d, *J* = 6.3 Hz), 134.3 (d, *J* = 9.0 Hz), 130.2 (d, *J* = 3.3 Hz), 119.1 (d, *J* = 22.2 Hz), 114.2 (d, *J* = 25.2 Hz).

HRMS (ESI, m/z): Mass calcd. for $C_{14}H_8F_2NaO_3S^+$ $[M+Na]^+$, 317.0054; found 317.0056.

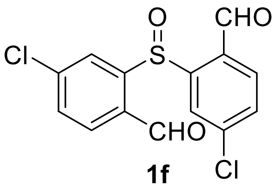

1f

2,2'-sulfinylbis(4-chlorobenzaldehyde), White Solid, 57% yield, m.p. 165.7 – 166.5 °C.

^{1}H NMR (400 MHz, DMSO-*d*$_6$) δ 10.31 (s, 2H), 8.12 (d, *J* = 2.3 Hz, 2H), 7.97 (dd, *J* = 8.5, 2.3 Hz, 2H), 7.88 (d, *J* = 8.4 Hz, 2H).

^{13}C NMR (101 MHz, DMSO-*d*$_6$) δ 190.7, 144.9, 136.5, 134.8, 134.5, 131.5, 128.1.

HRMS (ESI, m/z): Mass calcd. for $C_{14}H_8Cl_2NaO_3S^+$ $[M+Na]^+$, 348.9463; found 348.9457.

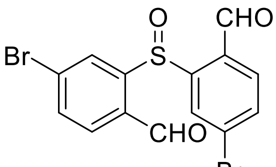

1g

2,2'-sulfinylbis(4-bromobenzaldehyde), White Solid, 48% yield, m.p. 164.8-165.6 °C.

^{1}H NMR (400 MHz, DMSO-*d*$_6$) δ 10.29 (s, 2H), 8.24 (d, *J* = 2.1 Hz, 2H), 8.10 (dd, *J* = 8.4, 2.2 Hz, 2H), 7.80 (d, *J* = 8.4 Hz, 2H).

^{13}C NMR (101 MHz, DMSO-*d*$_6$) δ 190.7, 145.3, 137.8, 134.6, 134.5, 128.2, 125.2.

HRMS (ESI, m/z): Mass calcd. for $C_{14}H_8Br_2NaO_3S^+$ $[M+Na]^+$, 436.8453; found 436.8446.

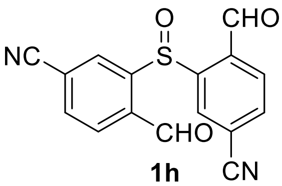

1h

3,3'-sulfinylbis(4-formylbenzonitrile), White Solid, 32% yield, m.p. > 250 °C.

^{1}H NMR (400 MHz, DMSO-*d*$_6$) δ 10.40 (s, 2H), 8.43 (d, *J* = 1.4 Hz, 2H), 8.26 (dd, *J* = 8.0, 1.5 Hz, 2H), 8.20 (d, *J* = 7.9 Hz, 2H).

^{13}C NMR (101 MHz, DMSO-*d*$_6$) δ 191.3, 146.7, 135.7, 135.6, 132.2, 130.4, 117.4, 117.3.

HRMS (ESI, m/z): Mass calcd. for $C_{16}H_8N_2NaO_3S^+$ $[M+Na]^+$, 331.0148; found 331.0135.

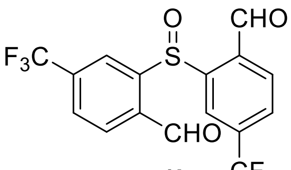

1i

2,2'-sulfinylbis(4-(trifluoromethyl)benzaldehyde), White Solid, 33% yield, m.p. 161.8 – 162.0 °C.

^{1}H NMR (400 MHz, DMSO-*d*$_6$) δ 10.45 (s, 2H), 8.30 (d, *J* = 7.9 Hz, 2H), 8.20 (dd, *J* = 8.0, 0.9 Hz, 2H), 8.12 (s, 2H).

^{19}F NMR (376 MHz, DMSO-*d*$_6$) δ -62.0.

^{13}C NMR (101 MHz, DMSO-*d*$_6$) δ 191.2, 147.3, 136.0, 134.0 (q, *J* = 32.9 Hz), 133.1, 128.8 (q, *J* = 3.6 Hz), 123.0 (q, *J* = 273.3 Hz), 122.2 (q, *J* = 4.2 Hz).

HRMS (ESI, m/z): Mass calcd. for $C_{16}H_9F_6O_3S^+$ $[M+H]^+$, 395.0171; found 395.0163.

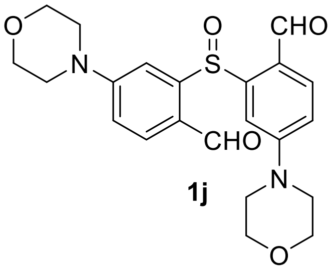

1j

2,2'-sulfinylbis(4-morpholinobenzaldehyde), White Solid, 43% yield, m.p. 219.4 – 219.7 °C.

^{1}H NMR (400 MHz, DMSO-*d*$_6$) δ 10.12 (s, 2H), 7.84 (d, *J* = 8.8 Hz, 2H), 7.26 (d, *J* = 2.5 Hz, 2H), 7.17 (dd, *J* = 8.8, 2.6 Hz, 2H), 3.71 (t, *J* = 4.9 Hz, 8H), 3.42 – 3.26 (m, 8H).

^{13}C NMR (101 MHz, DMSO-*d*$_6$) δ 188.6, 154.6, 148.7, 133.5, 123.0, 114.7, 109.1, 65.6, 46.4.

HRMS (ESI, m/z): Mass calcd. for $C_{22}H_{24}N_2NaO_5S^+$ $[M+Na]^+$, 451.1298; found 451.1297.

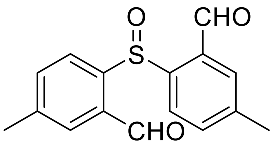

1k

2,2'-sulfinylbis(3-methylbenzaldehyde)，White Solid, 64% yield, m.p. 157.0 – 157.5 °C.

^{1}H NMR (400 MHz, CDCl$_3$) δ 10.44 (s, 2H), 7.86 (d, J = 8.0 Hz, 2H), 7.76 – 7.72 (d, J = 1.2 Hz, 2H), 7.56-7.54 (m, 2H), 2.45 (s, 6H).

^{13}C NMR (101 MHz, CDCl$_3$) δ 190.5, 144.4, 142.1, 135.6, 133.4, 132.0, 126.4, 21.2.

HRMS (ESI, m/z): Mass calcd. for $C_{16}H_{14}NaO_3S^+$ [M+Na]$^+$, 309.0556; found 309.0555.

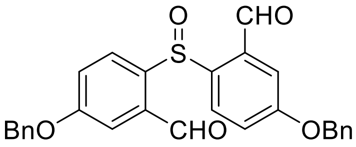

1l

6,6'-sulfinylbis(3-(benzyloxy)benzaldehyde), White Solid, 59% yield, m.p. 138.7 – 140.0 °C.

^{1}H NMR (400 MHz, DMSO-*d*$_6$) δ 10.35 (s, 2H), 7.80 (d, J = 8.7 Hz, 2H), 7.66 (d, J = 2.8 Hz, 2H), 7.52 (dd, J = 8.8, 2.7 Hz, 2H), 7.47 (d, J = 6.9 Hz, 4H), 7.44 – 7.32 (m, 6H), 5.23 (s, 4H).

^{13}C NMR (101 MHz, DMSO-*d*$_6$) δ 191.4, 160.4, 138.2, 136.1, 134.5, 128.6, 128.2, 128.0, 127.8, 121.3, 117.2, 70.0.

HRMS (ESI, m/z): Mass calcd. for $C_{28}H_{22}NaO_5S^+$ [M+Na]$^+$, 493.1080; found 493.1076.

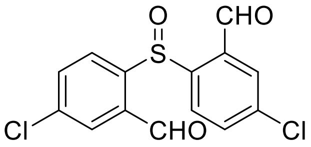

1m

6,6'-sulfinylbis(3-chlorobenzaldehyde), White Solid, 49% yield, m.p. 164.8-165.6 °C.

^{1}H NMR (400 MHz, DMSO-*d*$_6$) δ 10.30 (s, 2H), 8.12 (d, J = 2.3 Hz, 2H), 7.97 (dd, J = 8.4, 2.3 Hz, 2H), 7.88 (d, J = 8.4 Hz, 2H).

^{13}C NMR (101 MHz, DMSO-*d*$_6$) δ 190.8, 144.9, 136.5, 134.8, 134.5, 131.5, 128.1.

HRMS (ESI, m/z): Mass calcd. for $C_{14}H_8Cl_2NaO_3S^+$ [M+Na]$^+$, 348.9463; found 348.9456.

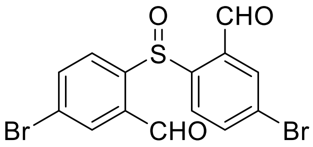

1n

6,6'-sulfinylbis(3-bromobenzaldehyde), White Solid, 43% yield, m.p. 178.9-179.4 °C.

^{1}H NMR (400 MHz, CDCl$_3$) δ 10.41 (s, 2H), 8.08 (d, J = 2.0 Hz, 2H), 7.90 (dd, J = 8.4, 2.0 Hz, 2H), 7.85 (d, J = 8.4 Hz, 2H).

^{13}C NMR (101 MHz, CDCl$_3$) δ 188.9, 145.7, 138.0, 134.7, 134.4, 128.2, 126.5.

HRMS (ESI, m/z): Mass calcd. for $C_{14}H_8Br_2NaO_3S^+$ [M+Na]$^+$, 436.8453; found 436.8446.

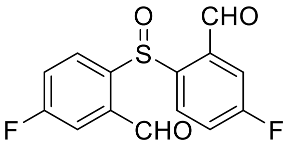

1o

6,6'-sulfinylbis(3-fluorobenzaldehyde), White Solid, 57% yield, m.p. 202.7 – 203.0 °C.

^{1}H NMR (400 MHz, CDCl$_3$) δ 10.44 (s, 2H), 8.00 (dd, J = 8.7, 4.9 Hz, 2H), 7.65 (dd, J = 8.1, 2.7 Hz, 2H), 7.46 (td, J = 8.1, 2.7 Hz, 2H)

^{19}F NMR (376 MHz, CDCl$_3$) δ -106.6.

^{13}C NMR (101 MHz, CDCl$_3$) δ 189.0 (d, J = 1.2 Hz), 164.35 (d, J = 255.8 Hz), 142.6 (d, J = 3.4 Hz), 135.6 (d, J = 6.5 Hz), 129.1 (d, J = 8.5 Hz), 122.2 (d, J = 22.0 Hz), 118.2 (d, J = 23.1 Hz).

HRMS (ESI, m/z): Mass calcd. for $C_{14}H_8F_2NaO_3S^+$ [M+Na]$^+$, 317.0054; found 317.0051.

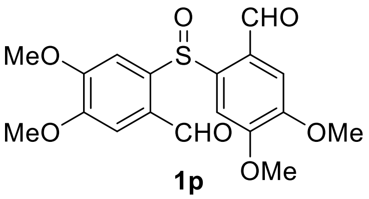

1p

6,6'-sulfinylbis(3,4-dimethoxybenzaldehyde), White Solid, 57% yield, m.p. 203.5-204.1 ℃.

^{1}H NMR (400 MHz, DMSO-*d*$_6$) δ 10.31 (s, 2H), 7.59 (s, 2H), 7.43 (s, 2H), 3.88 (s, 6H), 3.87 (s, 6H).

^{13}C NMR (101 MHz, DMSO-*d*$_6$) δ 189.7, 154.0, 150.5, 140.2, 126.5, 113.0, 107.6, 56.2, 56.0.

HRMS (ESI, m/z): Mass calcd. for $C_{18}H_{18}NaO_7S^+$ [M+Na]$^+$, 401.0665; found 401.0664.

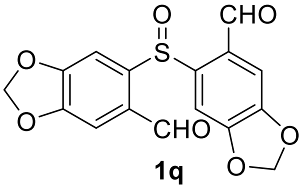

1q

5,5'-sulfinylbis(benzo[d][1,3]dioxole-4-carbaldehyde), White Solid, 47% yield, m.p. 247.3 – 247.6 ℃.

^{1}H NMR (400 MHz, DMSO-*d*$_6$) δ 10.25 (s, 2H), 7.52 (s, 2H), 7.40 (s, 2H), 6.28 (d, J = 0.8 Hz, 2H), 6.23 (d, J = 0.8 Hz, 2H).

^{13}C NMR (101 MHz, DMSO-*d*$_6$) δ 189.4, 153.2, 150.1, 143.1, 128.4, 109.7, 105.5, 103.5.

HRMS (ESI, m/z): Mass calcd. for $C_{16}H_{10}NaO_7S^+$ [M+Na]$^+$, 369.0039; found 369.0037.

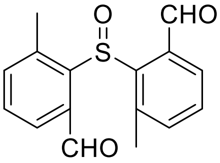

1r

2,2'-sulfinylbis(3-methylbenzaldehyde), White Solid, 37% yield, m.p. 151.0 – 151.9 ℃.

^{1}H NMR (400 MHz, CDCl$_3$) δ 10.82 (s, 2H), 7.71 (d, J = 7.6 Hz, 2H), 7.50 (t, J = 7.6 Hz, 2H), 7.34 (d, J = 7.5 Hz, 2H), 2.24 (s, 6H)

^{13}C NMR (101 MHz, CDCl$_3$) δ 189.7, 141.3, 137.7, 137.2, 135.8, 131.0, 127.1, 18.8.

HRMS (ESI, m/z): Mass calcd. for $C_{16}H_{14}NaO_3S^+$ [M+Na]$^+$, 309.0556; found 309.0557.

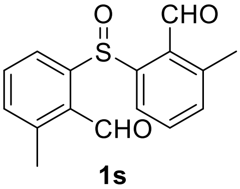

1s

2,2'-sulfinylbis(6-methylbenzaldehyde), White Solid, 45% yield, m.p. 121.3 – 121.9 ℃.

^{1}H NMR (400 MHz, CDCl$_3$) δ 10.82 (s, 2H), 7.81 (d, J = 7.9 Hz, 2H), 7.60 (t, J = 7.8 Hz, 2H), 7.37 (d, J = 7.6 Hz, 2H), 2.70 (s, 6H).

^{13}C NMR (101 MHz, CDCl$_3$) δ 190.9, 148.9, 142.0, 134.4, 134.1, 131.3, 124.5, 20.2.

HRMS (ESI, m/z): Mass calcd. for $C_{16}H_{14}NaO_3S^+$ [M+H]$^+$, 287.0736; found 287.0732.

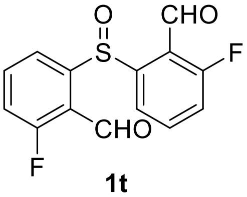

1t

2,2'-sulfinylbis(6-fluorobenzaldehyde), White Solid, 51% yield, m.p. 197.8 – 198.2 ℃.

^{1}H NMR (400 MHz, DMSO-*d*$_6$) δ 10.43 (s, 2H), 7.93 (td, J = 8.1, 5.4 Hz, 2H), 7.62 (dd, J = 10.4, 8.3 Hz, 4H).

^{19}F NMR (376 MHz, DMSO-*d*$_6$) δ -117.0.

^{13}C NMR (101 MHz, DMSO-*d*$_6$) δ 187.5 (d, J = 6.5 Hz), 163.3 (d, J = 261.5 Hz), 147.8 , 136.8 (d, J = 10.0 Hz), 122.2 (d, J = 3.4 Hz), 121.5 (d, J = 9.3 Hz), 119.4 (d, J = 21.2 Hz).

HRMS (ESI, m/z): Mass calcd. for $C_{14}H_8F_2NaO_3S^+$ [M+Na]$^+$, 317.0054; found 317.0049.

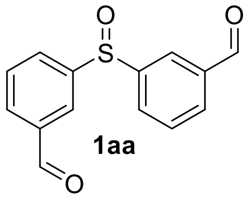
1aa

3,3'-sulfinyldibenzaldehyde, White Solid, 59% yield, m.p. 114.6 – 114.9 °C.
^{1}H NMR (400 MHz, CDCl$_3$) δ 10.03 (s, 2H), 8.18 (s, 2H), 7.96 (t, J = 8.0 Hz, 4H), 7.68 (t, J = 8.0 Hz, 2H).
^{13}C NMR (101 MHz, CDCl$_3$) δ 190.7, 146.9, 137.4, 132.5, 130.6, 130.0, 125.6.
HRMS (ESI, m/z): Mass calcd. for $C_{14}H_{11}O_3S^+$ [M+H]$^+$, 259.0423; found 259.0428.

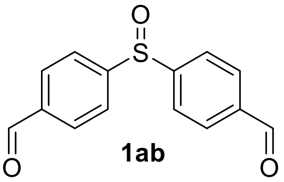
1ab

4,4'-sulfinyldibenzaldehyde, White Solid, 57% yield, m.p. 123.6–124.0 °C.
^{1}H NMR (400 MHz, CDCl$_3$) δ 10.03 (s, 2H), 7.98 (d, J = 8.0 Hz, 4H), 7.85 (d, J = 8.0 Hz, 4H).
^{13}C NMR (101 MHz, CDCl$_3$) δ 191.1, 151.3, 138.4, 130.8, 125.2.
HRMS (ESI, m/z): Mass calcd. for $C_{14}H_{11}O_3S^+$ [M+H]$^+$, 259.0423; found 259.0421.

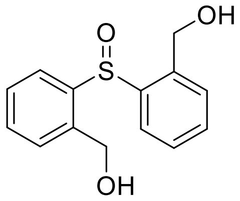
1ac

(sulfinylbis(2,1-phenylene))dimethanol, White Solid, 95% yield, m.p. 125.6–126.4 °C.
^{1}H NMR (400 MHz, CDCl$_3$) δ 7.70-7.67 (m, 2H), 7.52-7.43 (m, 6H), 4.81 (d, J = 13.0 Hz, 2H), 4.53 (d, J = 13.0 Hz, 2H), 3.64 (br, 2H).

methyl (*S*)-2-((2-formylphenyl)sulfinyl)benzoate

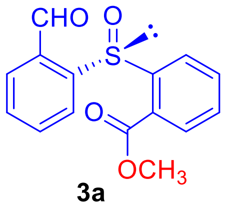
3a

White Solid, 25.7 mg, 89% yield, m.p. 121.8-122.3 °C.
[α]$^{25}_{D}$ = 33.8 (c = 0.5 in CHCl$_3$).
^{1}H NMR (400 MHz, CDCl$_3$) δ 10.85 (s, 1H), 8.37 (dd, J = 8.0, 1.2 Hz, 1H), 8.03 (dd, J = 7.7, 1.3 Hz, 1H), 7.97 (dd, J = 7.5, 1.2 Hz, 1H), 7.85 (td, J = 7.8, 1.3 Hz, 1H), 7.61 – 7.54 (m, 4H), 3.82 (s, 3H).
^{13}C NMR (101 MHz, CDCl$_3$) δ 190.1, 165.5, 148.9, 147.2, 134.8, 134.0, 133.8, 131.2, 131.1, 130.8, 128.8, 127.9, 126.7, 126.1, 52.9.
HRMS (ESI, m/z): Mass calcd. for $C_{15}H_{12}NaO_4S^+$ [M+Na]$^+$, 311.0349; found 311.0354.
UPLC analysis (Chiralcel IB-U; 25 °C, IPA/Hexane = 10/90, 0.5 mL/min, 254 nm), Rt$_1$ (major) = 11.8 min, Rt$_2$ (minor) = 17.5 min; >99 : 1 er.

methyl (*S*)-2-((2-formyl-5-methylphenyl)sulfinyl)-4-methylbenzoate

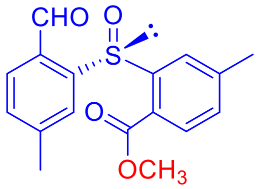
3b

White Solid, 25.6 mg, 81% yield, m.p. 130.6-131.5 °C.
[α]$^{25}_{D}$ = 55.1 (c = 0.5 in CHCl$_3$).
^{1}H NMR (400 MHz, CDCl$_3$) δ 10.82 (s, 1H), 8.16 (s, 1H), 7.93 (d, J = 7.9 Hz, 1H), 7.88 (d, J = 7.7 Hz, 1H), 7.38 (d, J = 7.9 Hz, 1H), 7.33 (d, J = 9.8 Hz, 2H), 3.79 (s, 3H), 2.56 (s, 3H), 2.34 (s, 3H).
^{13}C NMR (101 MHz, CDCl$_3$) δ 189.9, 165.5, 148.8, 146.8, 146.2, 145.1, 132.1, 131.9, 131.5, 131.2, 128.8, 126.8, 126.6, 125.2, 52.7, 22.1, 22.0.
HRMS (ESI, m/z): Mass calcd. for $C_{17}H_{17}O_4S^+$ [M+H]$^+$, 317.0842; found 317.0845.

HPLC analysis (Chiralcel IB; 25 °C, IPA / Hexane = 30/70, 0.5 mL/min, 254 nm), Rt_1 (major) = 23.4 min, Rt_2 (minor) = 32.0 min; > 99:1 er.

methyl (*S*)-2-((2-formyl-5-methoxyphenyl)sulfinyl)-4-methoxybenzoate

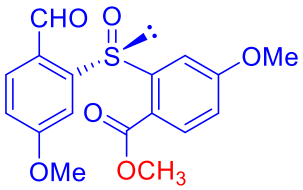

3c

White Solid, 28.6 mg, 82% yield, m.p. 136.5-136.9 °C.

$[\alpha]^{25}_{D}$ = 43.2 (*c* = 0.4 in $CHCl_3$).

^{1}H NMR (400 MHz, $CDCl_3$) δ 10.73 (s, 1H), 8.00 – 7.95 (m, 2H), 7.86 (d, *J* = 2.6 Hz, 1H), 7.05 – 7.00 (m, 3H), 3.97 (s, 3H), 3.79 (s, 3H), 3.78 (s, 3H).

^{13}C NMR (101 MHz, $CDCl_3$) δ 188.9, 165.1, 164.6, 164.2, 151.0, 149.5, 133.1, 131.2, 127.6, 120.1, 116.4, 112.0, 111.2, 56.2, 55.9, 52.6.

HRMS (ESI, m/z): Mass calcd. for $C_{17}H_{16}NaO_6S^+$ $[M+Na]^+$, 371.0560; found 371.0556.

HPLC analysis (Chiralcel IA; 25 °C, IPA / Hexane = 30/70, 0.5 mL/min, 254 nm), Rt_1 (major) = 42.8 min, Rt_2 (minor) = 47.7 min; > 99:1 er.

methyl (*S*)-4-(ethylthio)-2-((5-(ethylthio)-2-formylphenyl)sulfinyl) benzoate

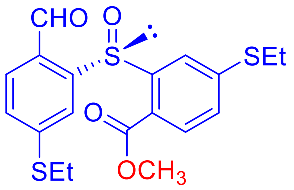

3d

White Solid, 33.9 mg, 83% yield, m.p. 78.1-78.9 °C.

$[\alpha]^{25}_{D}$ = 254.1 (*c* = 1.0 in $CHCl_3$).

^{1}H NMR (400 MHz, $CDCl_3$) δ 10.73 (s, 1H), 8.16 (d, *J* = 1.9 Hz, 1H), 7.89 (d, *J* = 8.2 Hz, 1H), 7.85 (d, *J* = 8.1 Hz, 1H), 7.37 (dd, *J* = 8.2, 1.9 Hz, 1H), 7.33 – 7.30 (m, 2H), 3.79 (s, 3H), 3.15-3.06 (m, 2H), 2.96-2.86 (m, 2H), 1.40 (t, *J* = 7.4 Hz, 3H), 1.24 (t, *J* = 7.3 Hz, 3H).

^{13}C NMR (101 MHz, $CDCl_3$) δ 189.1, 165.2, 149.1, 147.8, 147.6, 147.0, 131.2, 130.4, 129.0, 128.2, 127.8, 123.6, 123.1, 122.7, 52.7, 26.1, 25.9, 13.8, 13.6.

HRMS (ESI, m/z): Mass calcd. for $C_{19}H_{21}O_4S_3^+$ $[M+H]^+$, 409.0596; found 409.0595.

UPLC analysis (Chiralcel IB-U; 25 °C, IPA / Hexane = 10/90, 0.5 mL/min, 254 nm), Rt_1 (major) = 7.1 min, Rt_2 (minor) = 15.1 min; > 99:1 er.

methyl (*S*)-4-fluoro-2-((5-fluoro-2-formylphenyl)sulfinyl)benzoate

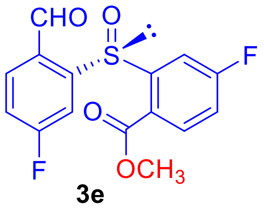

3e

White Solid, 24.6 mg, 76% yield, m.p. 107.3 – 107.8 °C.

$[\alpha]^{25}_{D}$ = 3.2 (*c* = 0.5 in $CHCl_3$).

^{1}H NMR (400 MHz, $CDCl_3$) δ 10.76 (s, 1H), 8.11-8.02 (m, 3H), 7.28 (ddd, *J* = 8.6, 7.4, 2.6 Hz, 1H), 7.25-7.22 (m, 2H), 3.83 (s, 3H).

^{19}F NMR (376 MHz, $CDCl_3$) δ -99.84, -100.84.

^{13}C NMR (101 MHz, $CDCl_3$) δ 188.3, 166.4 (d, *J* = 260.6 Hz), 166.3 (d, *J* = 261.6 Hz), 164.7 , 151.4 (d, *J* = 5.8 Hz), 150.8 (d, *J* = 5.9 Hz), 133.8 (d, *J* = 8.6 Hz), 131.7 (d, *J* = 8.8 Hz), 130.7 (d, *J* = 3.0 Hz), 124.0 (d, *J* = 3.6 Hz), 119.0 (d, *J* = 21.9 Hz), 118.3 (d, *J* = 22.2 Hz), 114.1 (d, *J* = 4.6 Hz), 113.9 (d, *J* = 3.4 Hz), 53.1.

HRMS (ESI, m/z): Mass calcd. for $C_{15}H_{10}F_2NaO_4S^+$ $[M+Na]^+$, 347.0160; found 347.0155.

HPLC analysis (Chiralcel IB; 25 °C, IPA / Hexane = 30/70, 0.5 mL/min, 254 nm), Rt_1 (major) = 22.8 min, Rt_2 (minor) = 30.0 min; > 99:1 er.

methyl (*S*)-4-chloro-2-((5-chloro-2-formylphenyl)sulfinyl)benzoate

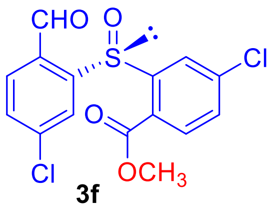

3f

White Solid, 29.3 mg, 82% yield, m.p. 98.6-99.1 °C.

$[\alpha]^{25}_D$ = 24.3 (c = 0.6 in $CHCl_3$).

^{1}H NMR (400 MHz, $CDCl_3$) δ 10.82 (s, 1H), 8.35 (d, J = 8.4 Hz, 1H), 8.02 (d, J = 2.3 Hz, 1H), 7.94 (d, J = 2.3 Hz, 1H), 7.84 (dd, J = 8.5, 2.2 Hz, 1H), 7.55 (dd, J = 8.5, 2.3 Hz, 1H), 7.47 (d, J = 8.5 Hz, 1H), 3.84 (s, 3H).

^{13}C NMR (101 MHz, $CDCl_3$) δ 188.5, 164.4, 146.9, 145.7, 138.2, 137.9, 135.3, 134.8, 134.0, 131.3, 128.9, 128.4, 128.3, 127.5, 53.3.

HRMS (ESI, m/z): Mass calcd. for $C_{15}H_{10}Cl_2NaO_4S^+$ $[M+Na]^+$, 378.9569; found 378.9564.

UPLC analysis (Chiralcel IA-U; 25 °C, IPA / Hexane = 10/90, 0.5 mL/min, 254 nm), Rt_1 (minor) = 9.8 min, Rt_2 (major) = 12.8 min; > 99:1 er.

methyl (*S*)-4-bromo-2-((5-bromo-2-formylphenyl)sulfinyl)benzoate

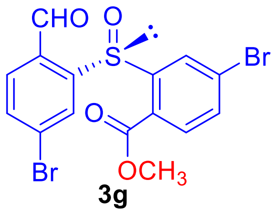

3g

White Solid, 41.9 mg, 94% yield, m.p. 121.7-122.1 °C.

$[\alpha]^{25}_D$ = 17.9 (c = 0.6 in $CHCl_3$).

^{1}H NMR (400 MHz, $CDCl_3$) δ 10.79 (s, 1H), 8.26 (d, J = 8.5 Hz, 1H), 8.16 (d, J = 2.1 Hz, 1H), 8.09 (d, J = 2.2 Hz, 1H), 7.99 (dd, J = 8.5, 2.1 Hz, 1H), 7.70 (dd, J = 8.4, 2.2 Hz, 1H), 7.39 (d, J = 8.4 Hz, 1H), 3.83 (s, 3H).

^{13}C NMR (101 MHz, $CDCl_3$) δ 188.4, 164.3, 147.3, 146.2, 137.7, 136.9, 135.2, 134.1, 131.4, 129.0, 128.5, 127.7, 126.3, 125.8, 53.3.

HRMS (ESI, m/z): Mass calcd. for $C_{15}H_{10}Br_2NaO_4S^+$ $[M+Na]^+$, 466.8559; found 466.8552.

HPLC analysis (Chiralcel IA; 25 °C, IPA / Hexane = 20/80, 0.5 mL/min, 254 nm), Rt_1 (minor) = 27.4 min, Rt_2 (major) = 39.8 min; 97:3 er.

methyl (*S*)-4-cyano-2-((5-cyano-2-formylphenyl)sulfinyl)benzoate

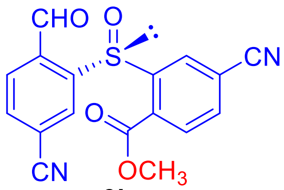

3h

White Solid, 20.3 mg, 60% yield, m.p. 190.8-191.2 °C.

$[\alpha]^{25}_D$ = 95.1 (c = 0.2 in $CHCl_3$).

^{1}H NMR (400 MHz, $CDCl_3$) δ 10.84 (s, 1H), 8.65 (d, J = 1.6 Hz, 1H), 8.18 (d, J = 8.0 Hz, 1H), 8.11 (d, J = 8.0 Hz, 1H), 7.95 (dd, J = 8.0, 1.6 Hz, 1H), 7.86 (dd, J = 8.0, 1.5 Hz, 1H), 7.82 (d, J = 1.5 Hz, 1H), 3.89 (s, 3H).

^{13}C NMR (101 MHz, $CDCl_3$) δ 188.2, 164.4, 149.2, 148.6, 136.3, 135.0, 134.9, 131.8, 131.1, 130.6, 129.8, 129.5, 118.6, 118.6, 116.8, 116.7, 53.8.

HRMS (ESI, m/z): Mass calcd. for $C_{17}H_{11}N_2O_4S^+$ $[M+H]^+$, 339.0434; found 339.0432.

HPLC analysis (Chiralcel ADH; 25 °C, IPA / Hexane = 30/70, 0.5 mL/min, 254 nm), Rt_1 (minor) = 50.5 min, Rt_2 (major) = 55.3 min; > 99:1 er.

methyl (*S*)-2-((2-formyl-5-(trifluoromethyl)phenyl)sulfinyl)-4-(trifluoromethyl)benzoate

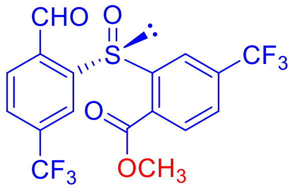

3i

White Solid, 25.9 mg, 61% yield, m.p. 131.3-131.6 °C.

$[\alpha]^{25}_{D}$ = 24.7 (*c* = 0.2 in $CHCl_3$).

^{1}H NMR (400 MHz, $CDCl_3$) δ 10.89 (s, 1H), 8.68 (s, 1H), 8.19 (d, *J* = 8.0 Hz, 1H), 8.12 (d, *J* = 8.0 Hz, 1H), 7.89 (d, *J* = 8.0 Hz, 1H), 7.82 (d, *J* = 8.0 Hz, 1H), 7.81(s, 1H), 3.89(s, 3H).

^{19}F NMR (376 MHz, $CDCl_3$) δ -63.17, -63.42.

^{13}C NMR (101 MHz, $CDCl_3$) δ 188.6, 164.7, 149.3, 148.7, 136.4 (q, *J* = 34.3 Hz), 136.3, 136.0, 131.8, 130.7, 129.5, 128.4 (q, *J* = 3.4 Hz), 128.3 (q, *J* = 3.6 Hz), 123.9 (q, *J* = 3.6 Hz), 123.3 (q, *J* = 3.6 Hz), 121.6 (q, *J* = 274.7 Hz), 121.5 (q, *J* = 270.7 Hz), 53.6.

HRMS (ESI, m/z): Mass calcd. for $C_{17}H_{10}F_6NaO_4S^+$ $[M+Na]^+$, 447.0096; found 447.0090.

HPLC analysis (Chiralcel IG; 25 °C, IPA / Hexane = 30/70, 0.5 mL/min, 254 nm), Rt_1 (minor) = 26.8 min, Rt_2 (major) = 30.3 min; 97:3 er.

methyl (*S*)-2-((2-formyl-5-morpholinophenyl)sulfinyl)-4-morpholinobenzoate

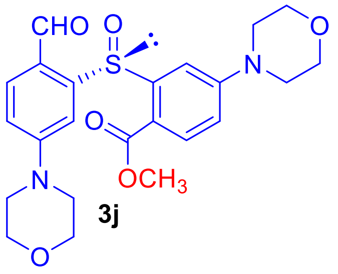

3j

White Solid, 37.6 mg, 82% yield, m.p. 91.9-92.4 °C.

$[\alpha]^{25}_{D}$ = 173.2 (*c* = 0.5 in $CHCl_3$).

^{1}H NMR (400 MHz, $CDCl_3$) δ 10.63 (s, 1H), 7.91 (d, *J* = 8.7 Hz, 1H), 7.87 (d, *J* = 8.6 Hz, 1H), 7.73 (d, *J* = 2.7 Hz, 1H), 6.91 (d, *J* = 2.6 Hz, 1H), 6.87 (dd, *J* = 8.6, 4.0 Hz, 2H), 3.86 (t, *J* = 4.8 Hz, 4H), 3.76 (t, *J* = 4.8 Hz, 4H), 3.74 (s, 3H), 3.44 – 3.34 (m, 4H), 3.26-3.13 (m, 4H).

^{13}C NMR (101 MHz, $CDCl_3$) δ 188.5, 165.2, 155.0, 154.4, 150.7, 148.5, 133.0, 130.9, 125.1, 117.1, 115.4, 114.3, 110.8, 110.4, 66.5, 66.4, 52.3, 47.5, 47.1.

HRMS (ESI, m/z): Mass calcd. for $C_{23}H_{26}N_2NaO_6S^+$ $[M+Na]^+$, 481.1404; found 481.1403.

UPLC analysis (Chiralcel IA-U; 25 °C, IPA / Hexane = 30/70, 0.5 mL/min, 254 nm), Rt_1 (major) = 11.2 min, Rt_2 (minor) = 18.8 min; > 99:1 er.

methyl (*S*)-2-((2-formyl-4-methylphenyl)sulfinyl)-5-methylbenzoate

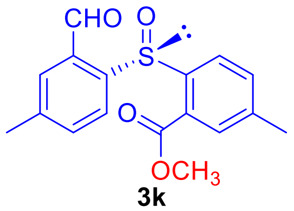

3k

White Solid, 26.9 mg, 85% yield, m.p. 105.1-105.6 °C.

$[\alpha]^{25}_{D}$ = 4.2 (*c* = 0.7 in $CHCl_3$).

^{1}H NMR (400 MHz, $CDCl_3$) δ 10.81 (s, 1H), 8.22 (d, *J* = 8.1 Hz, 1H), 7.82 (s, 1H), 7.75 (s, 1H), 7.63 (d, *J* = 8.0 Hz, 1H), 7.45 (d, *J* = 8.1 Hz, 1H), 7.38 (d, *J* = 8.0 Hz, 1H), 3.79 (s, 3H), 2.44 (s, 3H), 2.38 (s, 3H).

^{13}C NMR (101 MHz, $CDCl_3$) δ 190.3, 165.5, 146.2, 144.1, 141.7, 141.3, 135.5, 134.3, 133.8, 131.7, 128.0, 127.6, 126.7, 126.1, 52.7, 21.3, 21.2.

HRMS (ESI, m/z): Mass calcd. for $C_{17}H_{16}NaO_4S^+$ $[M+Na]^+$, 339.0662; found 339.0659.

HPLC analysis (Chiralcel IB; 25 °C, IPA / Hexane = 30/70, 0.5 mL/min, 254 nm), Rt_1 (major) = 30.3 min, Rt_2 (minor) = 35.2 min; > 99:1 er.

methyl (*S*)-5-(benzyloxy)-2-((4-(benzyloxy)-2-formylphenyl)sulfinyl)benzoate

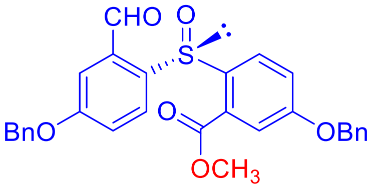

3l

White Solid, 40.0 mg, 80% yield, m.p. 173.3-174.0 °C.

$[\alpha]^{25}_D$ = 15.2 (c = 1.0 in $CHCl_3$).

^{1}H NMR (400 MHz, $CDCl_3$) δ 10.90 (s, 1H), 8.34 (d, J = 8.7 Hz, 1H), 7.63 (d, J = 2.7 Hz, 1H), 7.53 (d, J = 2.8 Hz, 1H), 7.46 – 7.32 (m, 12H), 7.15 (dd, J = 8.7, 2.8 Hz, 1H), 5.14 (s, 2H), 5.11 (s, 2H), 3.79 (s, 3H).

^{13}C NMR (101 MHz, $CDCl_3$) δ 189.8, 165.1, 160.7, 160.6, 135.9, 135.8, 135.7, 128.9, 128.9, 128.7, 128.6, 128.5, 127.7, 122.3, 119.7, 117.6, 112.1, 70.7, 70.6, 52.9.

HRMS (ESI, m/z): Mass calcd. for $C_{29}H_{24}NaO_6S^+$ $[M+Na]^+$, 523.1186; found 523.1192.

UPLC analysis (Chiralcel IA-U; 25 °C, IPA / Hexane = 30/70, 0.5 mL/min, 254 nm), Rt_1 (major) = 9.7 min, Rt_2 (minor) = 11.8 min; 98:2 er.

methyl (*S*)-5-chloro-2-((4-chloro-2-formylphenyl)sulfinyl)benzoate

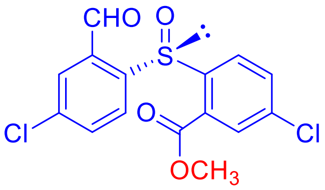

3m

White Solid, 25.4 mg, 71% yield, m.p. 108.6-109.1 °C.

$[\alpha]^{25}_D$ = 11.5 (c = 0.3 in $CHCl_3$).

^{1}H NMR (400 MHz, $CDCl_3$) δ 10.82 (s, 1H), 8.35 (d, J = 8.5 Hz, 1H), 8.02 (d, J = 2.2 Hz, 1H), 7.94 (d, J = 2.3 Hz, 1H), 7.84 (dd, J = 8.5, 2.3 Hz, 1H), 7.55 (dd, J = 8.5, 2.3 Hz, 1H), 7.47 (d, J = 8.5 Hz, 1H), 3.84 (s, 3H).

^{13}C NMR (101 MHz, $CDCl_3$) δ 188.5, 164.4, 146.8, 145.7, 138.1, 137.8, 135.2, 134.8, 134.0, 131.2, 128.9, 128.4, 128.3, 127.5, 53.3.

HRMS (ESI, m/z): Mass calcd. for $C_{15}H_{10}Cl_2NaO_4S^+$ $[M+Na]^+$, 378.9569; found 378.9568.

UPLC analysis (Chiralcel IA-U; 25 °C, IPA / Hexane = 20/80, 0.5 mL/min, 254 nm), Rt_1 (minor) = 4.5 min, Rt_2 (major) = 5.6 min; 98:2 er.

methyl (*S*)-5-bromo-2-((4-bromo-2-formylphenyl)sulfinyl)benzoate

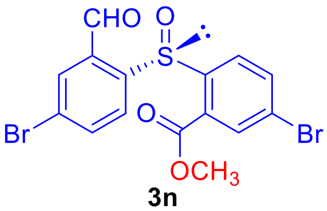

3n

White Solid, 34.4 mg, 77% yield, m.p. 118.6-119.3 °C.

$[\alpha]^{25}_D$ = 31.9 (c = 0.4 in $CHCl_3$).

^{1}H NMR (400 MHz, $CDCl_3$) δ10.80 (s, 1H), 8.27 (d, J = 8.5 Hz, 1H), 8.17 (d, J = 2.1 Hz, 1H), 8.09 (d, J = 2.2 Hz, 1H), 8.00 (dd, J = 8.5, 2.1 Hz, 1H), 7.71 (dd, J = 8.4, 2.2 Hz, 1H), 7.40 (d, J = 8.4 Hz, 1H), 3.84 (s, 3H).

^{13}C NMR (101 MHz, $CDCl_3$) δ 188.4, 164.3, 147.3, 146.2, 137.7, 137.0, 135.2, 134.1, 131.4, 129.0, 128.5, 127.7, 126.3, 125.8, 53.3.

HRMS (ESI, m/z): Mass calcd. for $C_{15}H_{10}Br_2NaO_4S^+$ $[M+Na]^+$, 466.8559; found 466.8552.

HPLC analysis (Chiralcel IA; 25 °C, IPA / Hexane = 50/50, 0.5 mL/min, 254 nm), Rt_1 (minor) = 27.8 min, Rt_2 (major) = 36.0 min; 98:2 er.

methyl (*S*)-5-fluoro-2-((4-fluoro-2-formylphenyl)sulfinyl)benzoate

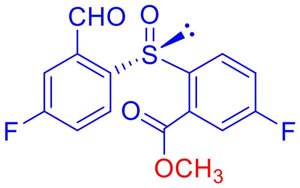

3o

White Solid, 26.0 mg, 80% yield, m.p. 113.8 – 114.2 °C.

$[\alpha]^{25}_{D}$ = 20.9 (c = 0.7 in $CHCl_3$).

^{1}H NMR (400 MHz, $CDCl_3$) δ 10.88 (d, J = 2.6 Hz, 1H), 8.45 (dd, J = 8.8, 5.2 Hz, 1H), 7.74 (dd, J = 8.6, 2.7 Hz, 1H), 7.65 (dd, J = 8.6, 2.8 Hz, 1H), 7.58 (ddd, J = 8.8, 7.4, 2.7 Hz, 1H), 7.52 (dd, J = 8.7, 4.9 Hz, 1H), 7.27 (ddd, J = 8.8, 7.4, 2.7 Hz, 1H), 3.83 (s, 3H).

^{19}F NMR (376 MHz, $CDCl_3$) δ -106.41, -108.33.

^{13}C NMR (101 MHz, $CDCl_3$) δ 188.4 (d, J = 1.1 Hz), 164.3 (d, J = 2.0 Hz), 164.1 (d, J = 255.5 Hz), 164.0 (d, J = 254.5 Hz), 144.5 (d, J = 2.6 Hz), 142.8 (d, J = 3.5 Hz), 136.5 (d, J = 6.8 Hz), 129.6, 129.5 (d, J = 8.7 Hz), 128.4 (d, J = 8.6 Hz), 122.1 (d, J = 22.6 Hz), 121.1 (d, J = 21.8 Hz), 118.6 (d, J = 24.6 Hz), 114.7 (d, J = 23.2 Hz), 53.3.

HRMS (ESI, m/z): Mass calcd. for $C_{15}H_{10}F_2NaO_4S^+$ $[M+Na]^+$, 347.0160; found 347.0156.

UPLC analysis (Chiralcel IB-U; 25 °C, IPA / Hexane = 20/80, 0.5 mL/min, 254 nm), Rt_1 (major) = 4.1 min, Rt_2 (minor) = 5.3 min; 98:2 er.

methyl (*S*)-2-((2-formyl-4,5-dimethoxyphenyl)sulfinyl)-4,5-dimethoxybenzoate

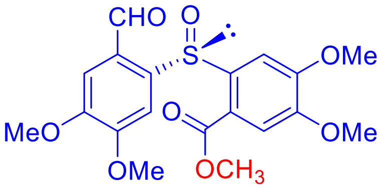

3p

White Solid, 35.5 mg, 87% yield, m.p. 164.8-165.6 °C.

$[\alpha]^{25}_{D}$ = 79.1 (c = 1.0 in $CHCl_3$).

^{1}H NMR (400 MHz, $CDCl_3$) δ 10.90 (s, 1H), 8.00 (s, 1H), 7.50 (s, 1H), 7.43 (s, 1H), 6.84 (s, 1H), 4.08 (s, 3H), 3.94 (s, 3H), 3.92 (s, 3H), 3.75 (s, 3H), 3.74 (s, 3H).

^{13}C NMR (101 MHz, $CDCl_3$) δ 188.7, 164.6, 154.1, 153.5, 151.2, 150.2, 142.6, 140.5, 128.4, 119.8, 113.3, 108.2, 107.9, 107.6, 56.8, 56.5, 56.3, 56.1, 52.6.

HRMS (ESI, m/z): Mass calcd. for $C_{19}H_{20}NaO_8S^+$ $[M+Na]^+$, 431.0771; found 431.0772.

HPLC analysis (Chiralcel IA; 25 °C, IPA / Hexane = 40/60, 0.5 mL/min, 254 nm), Rt_1 (major) = 27.0 min, Rt_2 (minor) = 31.0 min; 97:3 er.

methyl (*S*)-6-((6-formylbenzo[d][1,3]dioxol-5-yl)sulfinyl)benzo[d] [1,3]dioxole-5-carboxylate

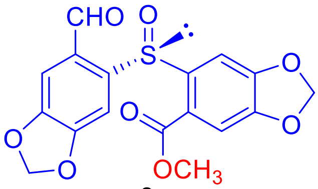

3q

White Solid, 32.0 mg, 85% yield, m.p. 149.6-150.0 °C.

$[\alpha]^{25}_{D}$ = 56.1 (c = 0.8 in $CHCl_3$).

^{1}H NMR (400 MHz, $CDCl_3$) δ 10.85 (s, 1H), 7.94 (s, 1H), 7.46 (s, 1H), 7.39 (s, 1H), 6.85 (s, 1H), 6.19 (q, J = 1.3 Hz, 2H), 6.05 (d, J = 1.2 Hz, 1H), 6.02 (d, J = 1.3 Hz, 1H), 3.76 (s, 3H).

^{13}C NMR (101 MHz, $CDCl_3$) δ 188.2, 164.5, 152.9, 152.9, 150.3, 149.9, 145.5, 143.4, 130.34, 121.7, 110.7, 106.3, 106.2, 106.1, 103.2, 102.8, 52.8.

HRMS (ESI, m/z): Mass calcd. for $C_{17}H_{13}O_8S^+$ $[M+H]^+$, 377.0326; found 377.0322.

HPLC analysis (Chiralcel IB; 25 °C, IPA / Hexane = 30/70, 0.5 mL/min, 254 nm), Rt_1 (major) = 9.8 min, Rt_2 (minor) = 12.0 min; 96:4 er.

methyl (*S*)-2-((2-formyl-6-methylphenyl)sulfinyl)-3-methylbenzoate

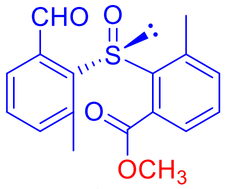

3r

White Solid, 15.9 mg, 47% yield, m.p. 81.6-82.3 °C.

$[\alpha]^{25}_D$ = -21.2 (*c* = 0.2 in $CHCl_3$).

^{1}H NMR (400 MHz, $CDCl_3$) δ 10.84 (s, 1H), 7.74 (d, *J* = 7.3 Hz, 1H), 7.48 (t, *J* = 7.6 Hz, 1H), 7.46 – 7.43 (m, 1H), 7.40 (t, *J* = 7.5 Hz, 1H), 7.35 (d, *J* = 7.4 Hz, 1H), 7.24 (d, *J* = 7.3 Hz, 1H), 3.80 (s, 3H), 2.29 (s, 3H), 2.17 (s, 3H).

^{13}C NMR (101 MHz, $CDCl_3$) δ 191.0, 168.6, 142.94, 140.33, 138.8, 138.7, 138.7, 136.5, 134.8, 134.2, 131.8, 131.2, 127.6, 127.3, 53.0, 20.0, 19.5.

HRMS (ESI, m/z): Mass calcd. for $C_{17}H_{17}O_4S^+$ $[M+H]^+$, 317.0842; found 317.0840.

HPLC analysis (Chiralcel IF; 25 °C, IPA / Hexane = 15/85, 0.5 mL/min, 254 nm), Rt_1 (minor) = 42.2 min, Rt_2 (major) = 51.7 min; > 99:1 er.

methyl (*S*)-2-((2-formyl-3-methylphenyl)sulfinyl)-6-methylbenzoate

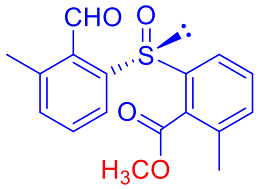

3s

White Solid, 12.0 mg, 38% yield, m.p. 90.4-90.8 °C.

$[\alpha]^{25}_D$ = -23.5 (*c* = 0.4 in $CHCl_3$).

^{1}H NMR (400 MHz, $CDCl_3$) δ 10.40 (s, 1H), 8.33 (d, *J* = 7.9 Hz, 1H), 7.77 (t, *J* = 7.8 Hz, 1H), 7.43 (d, *J* = 7.6 Hz, 1H), 7.30-7.24 (m, 2H), 7.17 (dd, *J* = 7.2, 1.9 Hz, 1H), 4.08 (s, 3H), 2.70 (s, 3H), 2.48 (s, 3H).

^{13}C NMR (101 MHz, $CDCl_3$) δ 190.1, 167.6, 148.8, 145.8, 143.0, 137.7, 134.4, 134.2, 133.2, 132.5, 131.0, 130.6, 124.5, 124.1, 52.6, 20.6, 19.4.

HRMS (ESI, m/z): Mass calcd. for $C_{17}H_{17}O_4S^+$ $[M+Na]^+$, 339.0662; found 339.0657.

HPLC analysis (Chiralcel IB; 25 °C, IPA / Hexane = 30/70, 0.5 mL/min, 254 nm), Rt_1 (major) = 53.4 min, Rt_2 (minor) = 65.0 min; 94:6 er.

methyl (*S*)-2-fluoro-6-((3-fluoro-2-formylphenyl)sulfinyl)benzoate

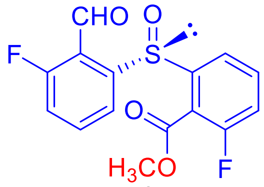

3t

White Solid, 25.9 mg, 80% yield, m.p. 84.9-85.3 °C.

$[\alpha]^{25}_D$ = -97.6 (*c* = 0.5 in $CHCl_3$).

^{1}H NMR (400 MHz, $CDCl_3$) δ 10.27 (s, 1H), 8.26 (d, *J* = 7.8 Hz, 1H), 7.92 (td, *J* = 8.1, 5.2 Hz, 1H), 7.42 (td, *J* = 8.1, 5.2 Hz, 1H), 7.38 (t, *J* = 8.1 Hz, 1H), 7.20 (d, *J* = 8.2 Hz, 2H), 4.11 (s, 3H).

^{19}F NMR (376 MHz, $CDCl_3$) δ -110.32, -117.28.

^{13}C NMR (101 MHz, $CDCl_3$) δ 186.4 (d, *J* = 8.0 Hz), 165.4 (d, *J* = 265.6 Hz), 163.8, 159.9 (d, *J* = 260.6 Hz), 149.8 , 147.5, 136.6 (d, *J* = 9.5 Hz), 133.1 (d, *J* = 8.7 Hz), 122.8 (d, *J* = 3.6 Hz), 121.9 (d, *J* = 3.2 Hz), 121.4 (d, *J* = 15.9 Hz), 121.0 (d, *J* = 8.7 Hz), 119.3 (d, *J* = 10.2 Hz), 119.0 (d, *J* = 9.2 Hz), 53.2.

HRMS (ESI, m/z): Mass calcd. for $C_{15}H_{10}F_2NaO_4S^+$ $[M+Na]^+$, 347.0160; found 347.0154.

UPLC analysis (Chiralcel IA-U; 25 °C, IPA / Hexane = 20/80, 0.5 mL/min, 254 nm), Rt_1 (major) = 5.2 min, Rt_2 (minor) = 6.0 min; 95:5 er.

isopropyl (*S*)-2-((2-formylphenyl)sulfinyl)benzoate

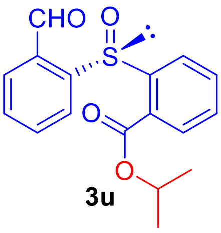

3u

White Solid, 22.2 mg, 70% yield, m.p. 78.8-79.6 °C.

[α]$^{25}_{D}$ = 7.5 (*c* = 0.5 in $CHCl_3$).

^{1}H NMR (400 MHz, $CDCl_3$) δ 10.87 (s, 1H), 8.38 (d, *J* = 7.9 Hz, 1H), 8.01 (d, *J* = 7.7 Hz, 1H), 7.96 (d, *J* = 7.5 Hz, 1H), 7.84 (t, *J* = 7.7 Hz, 1H), 7.60 – 7.50 (m, 4H), 5.14-5.04 (m, 1H), 1.31 (d, *J* = 6.2 Hz, 3H), 1.16 (d, *J* = 6.2 Hz, 3H).

^{13}C NMR (101 MHz, $CDCl_3$) δ 190.0, 164.6, 149.1, 147.3, 134.7, 134.1, 133.5, 131.1, 130.9, 130.7, 128.6, 128.4, 126.7, 126.0, 21.9, 21.8.

HRMS (ESI, m/z): Mass calcd. for $C_{17}H_{16}NaO_4S^+$ [M+Na]$^+$, 339.0662; found 339.0664.

UPLC analysis (Chiralcel IB-U; 25 °C, IPA / Hexane = 15/85, 0.5 mL/min, 254 nm), Rt_1 (major) = 4.8 min, Rt_2 (minor) = 6.5 min; 98:2 er.

***S*-ethyl (*S*)-2-((2-formylphenyl)sulfinyl)benzothioate**

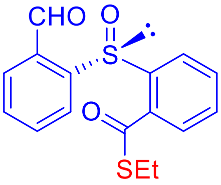

3v

White Solid, 12.7 mg, 40% yield, m.p. 101.0-101.6 °C.

[α]$^{25}_{D}$ = -82.4 (*c* = 0.5 in $CHCl_3$).

^{1}H NMR (400 MHz, $CDCl_3$) δ 10.97 (s, 1H), 8.44 (d, *J* = 7.9 Hz, 1H), 8.04 (d, *J* = 7.6 Hz, 1H), 7.98 (d, *J* = 7.6 Hz, 1H), 7.87 (t, *J* = 7.6 Hz, 1H), 7.63-7.50 (m, 4H), 2.93 (q, *J* = 7.4 Hz, 2H), 1.21 (t, *J* = 7.4 Hz, 3H).

^{13}C NMR (101 MHz, $CDCl_3$) δ 191.6, 189.9, 148.8, 145.0, 134.8, 134.4, 134.1, 133.9, 131.2, 131.0, 129.6, 128.2, 126.8, 126.1, 24.1, 14.5.

HRMS (ESI, m/z): Mass calcd. for $C_{16}H_{14}NaO_3S_2^+$ [M+Na]$^+$, 341.0277; found 341.0277.

HPLC analysis (Chiralcel IB; 25 °C, IPA / Hexane = 30/70, 0.5 mL/min, 254 nm), Rt_1 (major) = 27.7 min, Rt_2 (minor) = 36.9 min; 95:5 er.

benzyl (*S*)-2-((2-formylphenyl)sulfinyl)benzoate

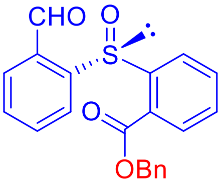

3w

White Solid, 31.0 mg, 85% yield, m.p. 91.1-91.5 °C.

[α]$^{25}_{D}$ = -26.3 (*c* = 1.0 in $CHCl_3$).

^{1}H NMR (400 MHz, $CDCl_3$) δ 10.91 (s, 1H), 8.42 (d, *J* = 7.9 Hz, 1H), 8.07 (d, *J* = 7.7 Hz, 1H), 7.96 (d, *J* = 7.1 Hz, 1H), 7.87 (t, *J* = 7.5 Hz, 1H), 7.56 (dt, *J* = 15.8, 7.0 Hz, 4H), 7.31 – 7.29 (m, 5H), 5.23 (dd, *J* = 16.0, 12.0 Hz, 2H).

^{13}C NMR (101 MHz, $CDCl_3$) δ 190.0, 164.8, 148.9, 147.5, 135.1, 134.8, 134.1, 133.9, 131.2, 131.1, 130.8, 128.7, 128.6, 128.5, 128.5, 127.7, 126.7, 126.1, 67.7.

HRMS (ESI, m/z): Mass calcd. for $C_{21}H_{16}NaO_4S^+$ [M+Na]$^+$, 387.0662; found 387.0665.

UPLC analysis (Chiralcel IB-U; 25 °C, IPA / Hexane = 15/85, 0.5 mL/min, 254 nm), Rt_1 (major) = 12.0 min, Rt_2 (minor) = 15.8 min; 95:5 er.

cyclohexyl (*S*)-2-((2-formylphenyl)sulfinyl)benzoate

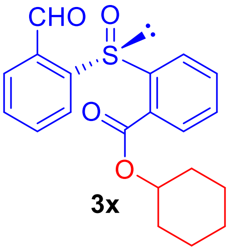

3x

White Solid, 22.4 mg, 63% yield, m.p. 87.7-88.2 °C.

[α]$^{25}_{D}$ = -49.8 (*c* = 0.3 in $CHCl_3$).

^{1}H NMR (400 MHz, $CDCl_3$) δ 10.86 (s, 1H), 8.36 (dd, *J* = 7.9, 1.2 Hz, 1H), 8.03 (dd, *J* = 7.7, 1.4 Hz, 1H), 7.97 (dd, *J* = 7.5, 1.4 Hz, 1H), 7.83 (td, *J* = 7.7, 1.4 Hz, 1H), 7.62-7.51 (m, 4H), 4.91-4.85 (m, 1H), 1.92-1.88 (m, 1H), 1.78-1.66 (m, 4H), 1.56-1.50 (m, 2H), 1.45-1.36 (m, 3H).

^{13}C NMR (101 MHz, CDCl$_3$) δ 190.1, 164.5, 149.1, 147.2, 134.8, 134.0, 133.5, 131.1, 130.9, 130.7, 128.9, 128.6, 126.7, 126.1, 74.6, 31.5, 31.4, 25.4, 23.7, 23.6.

HRMS (ESI, m/z): Mass calcd. for $C_{20}H_{20}NaO_4S^+$ [M+Na]$^+$, 379.0975; found 379.0974.

UPLC analysis (Chiralcel IB-U; 25 °C, IPA / Hexane = 15/85, 0.5 mL/min, 254 nm), Rt$_1$ (major) = 4.8 min, Rt$_2$ (minor) = 7.4 min; 98:2 er.

ethane-1,2-diyl bis(2-((*S*)-(2-formylphenyl)sulfinyl)benzoate)

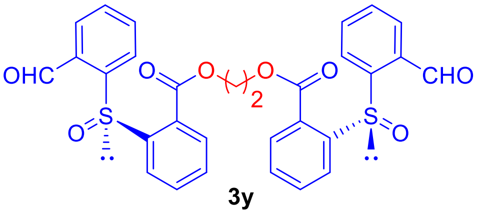

3y

White Solid, 24.1 mg, 84% yield, m.p. 67.6-67.9 °C.

[α]$^{25}_{D}$ = -29.1 (*c* = 0.8 in CHCl$_3$).

^{1}H NMR (400 MHz, CDCl$_3$) δ 10.69 (s, 2H), 8.27 (d, *J* = 7.6 Hz, 2H), 8.01 (d, *J* = 7.7 Hz, 2H), 7.94 (d, *J* = 7.0 Hz, 2H), 7.83 – 7.79 (m, 2H), 7.66 – 7.60 (m, 4H), 7.57 – 7.54 (m, 4H), 4.60 (d, *J* = 9.1 Hz, 2H), 4.44 (d, *J* = 9.1 Hz, 2H).

^{13}C NMR (101 MHz, CDCl$_3$) δ 190.1, 164.8, 148.6, 147.4, 134.9, 134.0, 133.9, 131.2, 131.1, 130.9, 129.4, 127.7, 126.6, 126.3, 63.3.

HRMS (ESI, m/z): Mass calcd. for $C_{30}H_{22}NaO_8S_2^+$ [M+Na]$^+$, 597.0648; found 597.0644.

HPLC analysis (Chiralcel IB; 25 °C, IPA / Hexane = 60/40, 1 mL/min, 254 nm), Rt$_1$ (major) = 38.9 min, Rt$_2$ (minor) = 66.1 min; > 99:1 er.

propane-1,3-diyl bis(2-((*S*)-(2-formylphenyl)sulfinyl)benzoate)

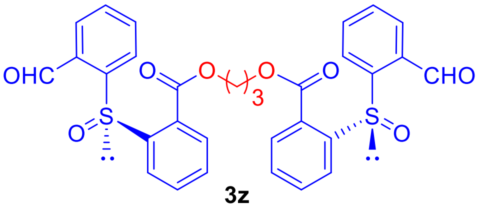

3z

White Solid, 25.0 mg, 85% yield, m.p. 68.4-68.6 °C.

[α]$^{25}_{D}$ = -23.1 (*c* = 0.8 in CHCl$_3$).

^{1}H NMR (400 MHz, CDCl$_3$) δ 10.73 (s, 2H), 8.30 (d, *J* = 7.8, 2H), 7.97 (dd, *J* = 7.8, 1.4 Hz, 2H), 7.92 (d, *J* = 4.0 Hz, 2H), 7.83 (td, *J* = 7.8, 1.4 Hz, 2H), 7.64-7.50 (m, 8H), 4.39 (dt, *J* = 12.0, 6.2 Hz, 2H), 4.28 (dt, *J* = 11.5, 6.0 Hz, 2H), 2.12-2.06 (m, 2H).

^{13}C NMR (101 MHz, CDCl$_3$) δ 190.1, 165.0, 148.8, 147.3, 134.9, 133.9, 131.2, 131.0, 130.9, 129.1, 129.0, 127.9, 126.7, 126.3, 62.7, 27.9.

HRMS (ESI, m/z): Mass calcd. for $C_{31}H_{24}NaO_8S_2^+$ [M+Na]$^+$, 611.0805; found 611.0798.

HPLC analysis (Chiralcel ADH; 25 °C, IPA / Hexane = 60/40, 1 mL/min, 254 nm), Rt$_1$ (major) = 41.8 min, Rt$_2$ (minor) = 65.2 min; > 99:1 er.

methyl (*S*)-2-((2-(formyl-d)phenyl)sulfinyl)benzoate

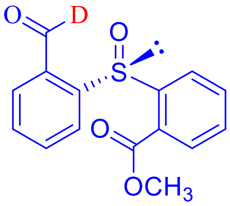

4a

White Solid, 22.3 mg, 77% yield, m.p. 115.1-115.6 °C.

[α]$^{25}_{D}$ = 17.1 (*c* = 0.3 in CHCl$_3$).

^{1}H NMR (400 MHz, CDCl$_3$) δ 8.39 (dd, *J* = 7.9, 1.3 Hz, 1H), 8.04 (dd, *J* = 7.7, 1.4 Hz, 1H), 7.98 (dd, *J* = 7.5, 1.4 Hz, 1H), 7.86 (td, *J* = 7.7, 1.4 Hz, 1H), 7.63-7.52 (m, 4H), 3.82 (s, 3H).

^{13}C NMR (101 MHz, CDCl$_3$) δ 189.7 (t, *J* = 26.2 Hz), 165.5, 148.9, 147.3, 134.8, 134.0 (t, *J* = 3.0 Hz), 133.8, 131.2, 131.1, 130.8, 128.7, 127.9, 126.7, 126.1, 52.9.

HRMS (ESI, m/z): Mass calcd. for $C_{15}H_{11}DNaO_4S^+$ [M+Na]$^+$, 312.0411; found 312.0414.

HPLC analysis (Chiralcel IB; 25 °C, IPA/Hexane = 30/70, 0.5 mL/min, 254 nm), Rt_1 (major) = 32.6 min, Rt_2 (minor) = 39.7 min; > 99:1 er.

methyl (*S*)-2-((2-cyanophenyl)sulfinyl)benzoate

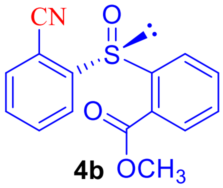

4b

White Solid, 19.7 mg, 69% yield, m.p. 142.8-143.3 °C.

[α]$^{25}_{D}$ = 74.5 (c = 0.3 in $CHCl_3$).

^{1}H NMR (400 MHz, $CDCl_3$) δ 8.58 (d, J = 7.9 Hz, 1H), 8.10 (dd, J = 7.7, 1.1 Hz, 1H), 7.95 (td, J = 7.8, 1.2 Hz, 1H), 7.76 (dd, J = 7.4, 1.5 Hz, 1H), 7.66 (td, J = 7.6, 1.0 Hz, 1H), 7.55 – 7.47 (m, 2H), 7.30 (dd, J = 7.7, 1.2 Hz, 1H), 3.84 (s, 1H).

^{13}C NMR (101 MHz, $CDCl_3$) δ 165.1, 149.6, 145.9, 133.9, 133.7, 133.6, 131.5, 131.2, 131.2, 127.4, 127.3, 126.4, 116.3, 113.8, 53.0.

HRMS (ESI, m/z): Mass calcd. for $C_{15}H_{11}NNaO_3S^+$ $[M+Na]^+$, 308.0352; found 308.0350.

HPLC analysis (Chiralcel ADH; 25 °C, IPA / Hexane = 40/60, 0.5 mL/min, 254 nm), Rt_1 (minor) = 20.0 min, Rt_2 (major) = 21.7 min; 98:2 er.

methyl (*R*)-2-((2-((ethylthio)carbonyl)phenyl)sulfinyl)benzoate

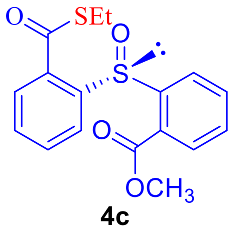

4c

White Solid, 32.0 mg, 92% yield, m.p. 93.0-93.4 °C.

[α]$^{25}_{D}$ = 89.3 (c = 1.0 in $CHCl_3$).

^{1}H NMR (400 MHz, $CDCl_3$) δ 8.03 (dd, J = 8.0, 1.2 Hz, 1H), 7.97 (dd, J = 7.8, 1.3 Hz, 1H), 7.93 (dd, J = 7.7, 1.4 Hz, 1H), 7.79 (dd, J = 8.0, 1.3 Hz, 1H), 7.68 (td, J = 7.7, 1.3 Hz, 1H), 7.59 (td, J = 7.7, 1.5 Hz, 1H), 7.52 (td, J = 7.6, 1.3 Hz, 1H), 7.47 (td, J = 7.5, 1.3 Hz, 1H), 3.96 (s, 3H), 3.02 (q, J = 7.4 Hz, 2H), 1.29 (t, J = 7.4 Hz, 3H).

^{13}C NMR (101 MHz, $CDCl_3$) δ 191.5, 166.2, 147.6, 146.2, 135.5, 133.4, 133.1, 130.6, 130.5, 130.3, 129.8, 129.0, 127.1, 126.6, 52.6, 24.1, 14.6.

HRMS (ESI, m/z): Mass calcd. for $C_{17}H_{16}NaO_4S_2^+$ $[M+Na]^+$, 371.0382; found 371.0381.

UPLC analysis (Chiralcel IA-U; 25 °C, IPA / Hexane = 20/80, 0.5 mL/min, 254 nm), Rt_1 (major) = 4.65 min, Rt_2 (minor) = 5.43 min; > 99:1 er.

methyl (*S*)-2-((2-ethynylphenyl)sulfinyl)benzoate

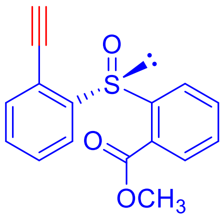

4d

White Solid, 16.2 mg, 57% yield, m.p. 132.4-132.8 °C.

[α]$^{25}_{D}$ = 56.8 (c = 0.1 in $CHCl_3$).

^{1}H NMR (400 MHz, $CDCl_3$) δ 8.42 (dd, J = 7.9, 1.3 Hz, 1H), 8.10 (dd, J = 7.7, 1.4 Hz, 1H), 7.88 (td, J = 7.7, 1.4 Hz, 1H), 7.63 (ddd, J = 14.9, 7.5, 1.3 Hz, 2H), 7.37 (td, J = 7.6, 1.3 Hz, 1H), 7.28 (td, J = 7.6, 1.3 Hz, 1H), 7.03 (dd, J = 7.9, 1.3 Hz, 1H), 3.81 (s, 3H), 3.56 (s, 1H).

^{13}C NMR (101 MHz, $CDCl_3$) δ 165.4, 148.6, 145.7, 133.8, 133.6, 131.4, 130.9, 130.8, 130.0, 128.3, 127.1, 125.7, 123.0, 84.9, 79.9, 52.8.

HRMS (ESI, m/z): Mass calcd. for $C_{16}H_{12}NaO_3S^+$ $[M+Na]^+$, 307.0399; found 307.0399.

HPLC analysis (Chiralcel IA; 25 °C, IPA / Hexane = 30/70, 0.5 mL/min, 254 nm), Rt_1 (major) = 22.8 min, Rt_2 (minor) = 24.4 min; 99:1 er.

methyl (*S*)-2-((2-vinylphenyl)sulfinyl)benzoate

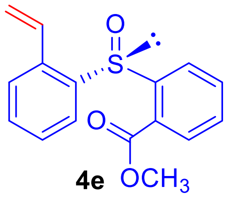

4e

White Solid, 22.9 mg, 80% yield, m.p. 126.2-126.9 °C.

$[\alpha]^{25}_{D}$ = -2.7 (*c* = 0.1 in $CHCl_3$).

^{1}H NMR (400 MHz, $CDCl_3$) δ 8.51 (dd, *J* = 8.0, 1.3 Hz, 1H), 8.08 (dd, *J* = 7.7, 1.4 Hz, 1H), 7.90 (td, *J* = 7.6, 1.4 Hz, 1H), 7.80 (dd, *J* = 17.3, 11.0 Hz, 1H), 7.64 - 7.60 (m, 2H), 7.36 (t, *J* = 7.8 Hz, 1H), 7.17 (td, *J* = 7.7, 1.4 Hz, 1H), 7.01 (dd, *J* = 7.9, 1.3 Hz, 1H), 5.86 (dd, *J* = 17.4, 1.1 Hz, 1H), 5.56 (dd, *J* = 11.0, 1.1 Hz, 1H), 3.75 (s, 3H).

^{13}C NMR (101 MHz, $CDCl_3$) δ 165.2, 146.6, 144.4, 138.4, 133.6, 133.1, 131.4, 131.3, 130.6, 128.9, 127.7, 126.8, 126.3, 126.2, 118.4, 52.7.

HRMS (ESI, m/z): Mass calcd. for $C_{16}H_{15}O_3S^+$ $[M+H]^+$, 287.0736; found 287.0735.

HPLC analysis (Chiralcel IB; 25 °C, IPA / Hexane = 40/60, 0.5 mL/min, 254 nm), Rt_1 (major) = 15.3 min, Rt_2 (minor) = 17.2 min; > 99:1 er.

methyl (*S*)-2-((2-ethylphenyl)sulfinyl)benzoate

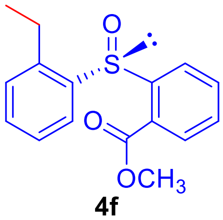

4f

White Solid, 27.1 mg, 94% yield, m.p. 121.3-121.6 °C.

$[\alpha]^{25}_{D}$ = -36.9 (*c* = 0.2 in $CHCl_3$).

^{1}H NMR (400 MHz, $CDCl_3$) δ 8.51 (d, *J* = 7.7 Hz, 1H), 8.07 (d, *J* = 7.7 Hz, 1H), 7.90 (t, *J* = 7.7 Hz, 1H), 7.61 (t, *J* = 7.5 Hz, 1H), 7.32 (d, *J* = 4.0 Hz, 2H), 7.10 - 7.06 (m, 1H), 6.91 (d, *J* = 7.9 Hz, 1H), 3.75 (s, 3H), 3.46 (dq, *J* = 15.1, 7.6 Hz, 1H), 3.18 (dq, *J* = 15.0, 7.6 Hz, 1H), 1.45 (t, *J* = 7.6 Hz, 3H).

^{13}C NMR (101 MHz, $CDCl_3$) δ 165.1, 146.8, 145.0, 144.7, 133.6, 131.3, 131.2, 130.5, 129.2, 127.7, 127.1, 126.9, 126.1, 52.5, 25.7, 15.7.

HRMS (ESI, m/z): Mass calcd. for $C_{16}H_{16}NaO_3S^+$ $[M+Na]^+$, 311.0712; found 311.0711.

UPLC analysis (Chiralcel OD-3; 25 °C, IPA/Hexane = 20/80, 0.5 mL/min, 254 nm), Rt_1 (major) = 6.0 min, Rt_2 (minor) = 8.1 min; 97:3 er.

methyl 2-((*S*)-(2-((*S*)-4-isopropyl-4,5-dihydrooxazol-2-yl)phenyl)sulfinyl)benzoate

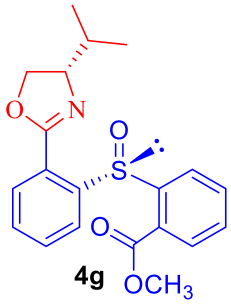

4g

White Solid, 26.3 mg, 71% yield, m.p. 110.6-110.9 °C.

$[\alpha]^{25}_{D}$ = 36.2 (*c* = 1.0 in $CHCl_3$).

^{1}H NMR (400 MHz, $CDCl_3$) δ 8.01 (d, *J* = 7.9 Hz, 1H), 7.96 (d, *J* = 7.5 Hz, 1H), 7.90 (dd, *J* = 7.5, 1.0 Hz, 1H), 7.67 – 7.44 (m, 5H), 4.24 (t, *J* = 8.0 Hz, 1H), 4.08 (t, *J* = 8.0 Hz, 1H), 3.99 – 3.94 (m, 1H), 3.93 (s, 3H), 1.84 (dq, *J* = 13.3, 6.7 Hz, 1H), 0.97 (d, *J* = 6.8 Hz, 3H), 0.86 (d, *J* = 6.7 Hz, 3H).

^{13}C NMR (101 MHz, $CDCl_3$) δ 166.2, 161.0, 147.8, 146.0, 133.2, 131.6, 130.5, 130.4, 130.3, 130.0, 127.0, 126.9, 126.7, 126.6, 72.7, 69.9, 52.6, 32.5, 19.0, 17.8.

HRMS (ESI, m/z): Mass calcd. for $C_{20}H_{21}NNaO_4S^+$ $[M+Na]^+$, 394.1083; found 394.1087.

(2-((*S*)-(2-((((*R*)-1-(naphthalen-1-yl)ethyl)amino)methyl)phenyl)sulfinyl)phenyl)methanol

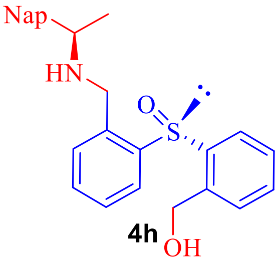

4h

White Solid, 24.9 mg, 60% yield of two steps, m.p. 64.8-65.6 °C.

[α]$^{25}_{D}$ = -7.1 (c = 0.7 in $CHCl_3$).

^{1}H NMR (400 MHz, $CDCl_3$) δ 8.11 (d, J = 7.9 Hz, 1H), 7.97 (dd, J = 7.5, 1.6 Hz, 1H), 7.90 (dd, J = 7.5, 1.0 Hz, 1H), 7.80 (d, J = 8.0 Hz, 1H), 7.57 (d, J = 6.8, 2H), 7.53 – 7.48 (m, 3H), 7.46 – 7.40 (m, 2H), 7.39 – 7.34 (m, 2H), 7.30 (dd, J = 7.2, 1.6 Hz, 1H), 7.24 (dd, J = 7.2, 1.6 Hz, 1H), 4.77 (d, J = 6.6, 2H), 4.53 (d, J = 12.0, 1H), 4.28 (dd, J = 16.0, 12.0 Hz, 2H), 3.76 (d, J = 12.0 Hz, 1H), 3.32 (br, 2H), 1.56 (d, J = 4.0 Hz, 3H).

^{13}C NMR (101 MHz, $CDCl_3$) δ 143.2, 142.6, 140.1, 140.0, 138.9, 134.1, 131.8, 131.2, 131.0, 130.2, 129.5, 129.4, 129.3, 128.4, 127.9, 127.8, 126.4, 126.3, 125.8, 125.7, 122.6, 122.5, 62.2, 53.6, 49.0, 22.6.

HRMS (ESI, m/z): Mass calcd. for $C_{26}H_{25}NNaO_2S^+$ $[M+Na]^+$, 438.1498; found 438.1497.

(*S*)-2-((2-((benzylamino)methyl)phenyl)sulfinyl)benzoic acid

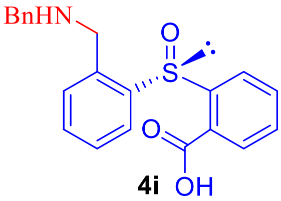

4i

White Solid, 28.4 mg, 78% yield of three steps, m.p. 147.0-147.6 °C.

[α]$^{25}_{D}$ = -12.1 (c = 0.3 in $CHCl_3$).

^{1}H NMR (400 MHz, $CDCl_3$) δ 12.70 (br, 1H), 9.46 (br, 1H), 8.46 (d, J = 7.8 Hz, 1H), 8.24 (d, J = 7.4 Hz, 1H), 7.83 (t, J = 7.3 Hz, 1H), 7.67 (t, J = 7.4 Hz, 1H), 7.48 (d, J = 7.4 Hz, 2H), 7.36 (t, J = 7.5 Hz, 2H), 7.29 (d, J = 7.3 Hz, 1H), 7.05 (d, J = 7.6 Hz, 1H), 6.97 (t, J = 7.6 Hz, 1H), 6.75 (d, J = 7.8 Hz, 1H), 6.64 (t, J = 7.5 Hz, 1H), 4.61 (d, J = 12.5 Hz, 1H), 3.91 (dd, J = 15.2, 13.1 Hz, 2H), 3.82 (d, J = 13.0 Hz, 1H).

^{13}C NMR (101 MHz, $CDCl_3$) δ 169.9, 149.3, 144.0, 134.7, 131.6, 131.2, 131.2, 131.0, 130.9, 130.6, 130.5, 130.4, 129.9, 129.6, 129.3, 126.1, 125.5, 51.4, 47.3.

HRMS (ESI, m/z): Mass calcd. for $C_{21}H_{18}NO_3S^-$ $[M-H]^-$, 364.1013; found 438.1497.

UPLC analysis (Chiralcel IB-U; 25 °C, IPA / Hexane = 30/70, 0.5 mL/min, 254 nm), Rt_1 (major) = 9.8 min, Rt_2 (minor) = 18.2 min; > 99:1 er.

methyl 2-((*S*)-(2-(((*S*)-tert-butylsulfinyl)imino)methyl)phenyl)sulfinyl)benzoate

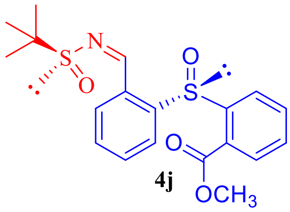

4j

White Solid, 35.2 mg, 90% yield, m.p. 158.0-159.0 °C.

[α]$^{25}_{D}$ = 219.8 (c = 0.5 in $CHCl_3$).

^{1}H NMR (400 MHz, $CDCl_3$) δ 9.53 (s, 1H), 8.56 (dd, J = 8.0, 1.3 Hz, 1H), 8.04 (td, J = 8.1, 1.5 Hz, 2H), 7.92 (td, J = 7.6, 1.4 Hz, 1H), 7.62 (td, J = 7.6, 1.3 Hz, 1H), 7.47 (td, J = 7.5, 1.4 Hz, 1H), 7.41 (td, J = 7.6, 1.6 Hz, 1H), 7.28 (dd, J = 7.8, 1.4 Hz, 1H), 3.73 (s, 3H), 1.32 (s, 9H).

^{13}C NMR (101 MHz, $CDCl_3$) δ 164.9, 160.8, 147.6, 147.3, 133.8, 133.5, 132.8, 131.3, 131.1, 130.7, 128.7, 127.3, 127.2, 126.2, 58.3, 52.8, 22.9.

HRMS (ESI, m/z): Mass calcd. for $C_{19}H_{21}NNaO_4S_2^+$ $[M+Na]^+$, 414.0804; found 414.0800.

methyl 2-((*S*)-(2-(1-((2*S*,3*S*,5*R*)-2-(hydroxymethyl)-5-(5-methyl-2,4-dioxo-3,4-dihydropyrimidin-1(2H)-yl)tetrahydrofuran-3-yl)-1H-1,2,3-triazol-4-yl)phenyl)sulfinyl)benzoate

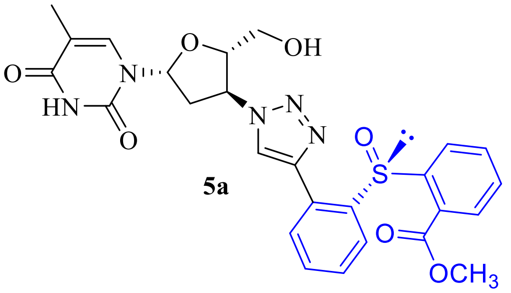

5a

White Solid, 32.5 mg, 59% yield, m.p. > 250 °C.
[α]$^{25}_{D}$ = 14.2 (c = 0.5 in $CHCl_3$).
^{1}H NMR (400 MHz, DMSO-*d*$_6$) δ 11.37 (s, 1H), 8.85 (s, 1H), 8.10 (dd, J = 7.9, 1.2 Hz, 1H), 7.99 (dd, J = 7.7, 1.4 Hz, 1H), 7.93 (td, J = 7.7, 1.4 Hz, 1H), 7.87 – 7.85 (m, 2H), 7.70 (td, J = 7.5, 1.3 Hz, 1H), 7.60 (td, J = 7.5, 1.3 Hz, 1H), 7.47 (td, J = 7.6, 1.4 Hz, 1H), 7.33 (dd, J = 7.9, 1.3 Hz, 1H), 6.50 (t, J = 6.6 Hz, 1H), 5.51 (dt, J = 8.6, 5.3 Hz, 1H), 5.33 (t, J = 5.2 Hz, 1H), 4.32 (dt, J = 5.4, 3.6 Hz, 1H), 3.79-3.71 (m, 2H), 3.69 (s, 3H), 2.84 (ddd, J = 13.9, 6.8, 5.2 Hz, 1H), 2.73 (ddd, J = 14.4, 8.6, 6.5 Hz, 1H), 1.83 (s, 3H).
^{13}C NMR (101 MHz, DMSO-*d*$_6$) δ 165.0, 163.7, 150.5, 146.0, 143.8, 143.7, 136.2, 133.6, 131.3, 131.0, 130.6, 130.3, 129.2, 129.1, 128.2, 126.6, 125.9, 124.4, 109.67, 84.5, 83.9, 60.8, 59.5, 52.6, 37.1, 12.3.
HRMS (ESI, m/z): Mass calcd. for $C_{26}H_{25}N_5NaO_7S^+$ [M+Na]$^+$, 574.1367; found 574.1365.

dimethyl (*R*,*E*)-2-(1,3-diphenylallyl)malonate

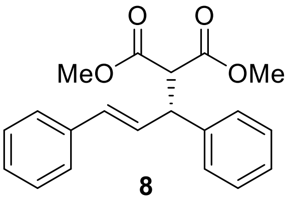

8

Colorless oil, 21.4 mg, 66% yield.
[α]$^{25}_{D}$ = 22.5 (c = 0.1 in $CHCl_3$).
^{1}H NMR (400 MHz, CDCl$_3$) δ 7.33-7.26 (m, 7H), 7.25-7.17 (m, 3H), 6.47 (d, J = 15.8 Hz, 1H), 6.32 (dd, J = 15.7, 8.6 Hz, 1H), 4.26 (dd, J = 10.7, 8.7 Hz, 1H), 3.95 (d, J = 10.9 Hz, 1H), 3.70 (s, 3H), 3.51 (s, 3H).
^{13}C NMR (101 MHz, CDCl$_3$) δ 168.4, 167.9, 140.3, 137.0, 132.0, 129.2, 128.9, 128.6, 128.0, 127.7, 127.3, 126.5, 57.8, 52.8, 52.6, 49.3.
HRMS (ESI, m/z): Mass calcd. for $C_{20}H_{20}NaO_4^+$ [M+Na]$^+$, 347.1254; found 347.1254.
HPLC analysis (Chiralcel ADH; 25 °C, IPA / Hexane = 10/90, 0.5 mL/min, 254 nm), Rt$_1$ (minor) = 29.0 min, Rt$_2$ (major) = 40.1 min; 98:2 er.

methyl 3-((3-formylphenyl)sulfinyl)benzoate

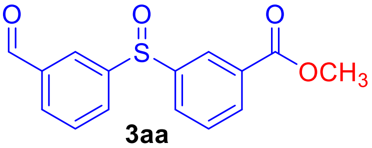

3aa

White Solid, 15 mg, 52% yield, m.p. 60.6-60.9 °C.
^{1}H NMR (400 MHz, CDCl$_3$) δ 10.04 (s, 1H), 8.33 (t, J = 1.8 Hz, 1H), 8.18 (t, J = 1.7 Hz, 1H), 8.14 (dt, J = 7.8, 1.4 Hz, 1H), 7.98-7.93 (m, 2H), 7.89 (dt, J = 7.8, 1.5 Hz, 1H), 7.67 (t, J = 7.7 Hz, 1H), 7.59 (t, J = 7.8 Hz, 1H), 3.94 (s, 3H).
^{13}C NMR (101 MHz, CDCl$_3$) δ 190.8, 165.7, 147.2, 146.0, 137.4, 132.7, 132.2, 131.8, 130.5, 130.1, 130.0, 128.7, 125.9, 125.7, 52.7.
HRMS (ESI, m/z): Mass calcd. for $C_{15}H_{12}NaO_4S^+$ [M+Na]$^+$, 311.0349; found 311.0346.
UPLC analysis (Chiralcel IB-U; 25 °C, IPA/Hexane = 10/90, 0.5 mL/min, 254 nm), Rt$_1$ (minor) = 13.3 min, Rt$_2$ (major) = 14.4 min; 48:52 er.

dimethyl 3,3'-sulfinyldibenzoate

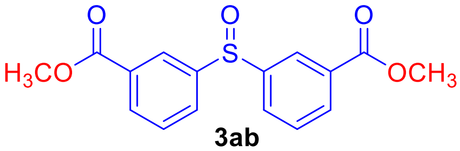

3ab

White Solid, 11.8 mg, 37% yield, m.p. 58.1-58.8 °C.

^{1}H NMR (400 MHz, CDCl$_3$) δ 8.31 (t, J = 1.5 Hz, 2H), 8.12 (dt, J = 7.7, 1.2 Hz, 2H), 7.87 (dt, J = 7.8, 1.4 Hz, 2H), 7.57 (t, J = 7.8 Hz, 2H), 3.93 (s, 6H).

^{13}C NMR (101 MHz, CDCl$_3$) δ 165.8, 146.3, 132.5, 131.7, 129.9, 128.8, 125.9, 52.7.

HRMS (ESI, m/z): Mass calcd. for $C_{16}H_{14}NaO_5S^+$ [M+Na]$^+$, 341.0454; found 341.0458.

methyl 4-((4-formylphenyl)sulfinyl)benzoate

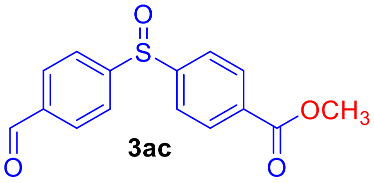

3ac

White Solid, 8.6 mg, 29% yield, m.p. 118.9-119.7 °C.

^{1}H NMR (400 MHz, CDCl$_3$) δ 10.03 (s, 1H), 8.14 (d, J = 8.5 Hz, 2H), 7.98 (d, J = 8.4 Hz, 2H), 7.84 (d, J = 8.3 Hz, 2H), 7.76 (d, J = 8.5 Hz, 2H), 3.92 (s, 3H).

^{13}C NMR (101 MHz, CDCl$_3$) δ 191.1, 165.9, 151.6, 149.8, 138.3, 133.1, 130.9, 130.7, 125.1, 124.6, 52.7.

HRMS (ESI, m/z): Mass calcd. for $C_{15}H_{13}O_4S^+$ [M+H]$^+$, 289.0529; found 289.0532.

UPLC analysis (Chiralcel IC-U; 25 °C, IPA/Hexane = 20/80, 0.5 mL/min, 254 nm), Rt_1 (minor) = 15.6 min, Rt_2 (major) = 14.7 min; 47:53 er.

dimethyl 4,4'-sulfinyldibenzoate

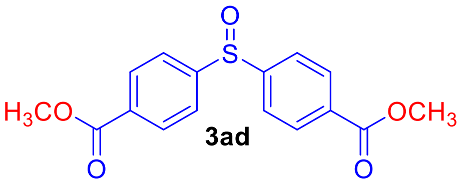

3ad

White Solid, 18.8 mg, 59% yield, m.p. 116.3-116.7 °C.

^{1}H NMR (400 MHz, CDCl$_3$) δ 8.12 (d, J = 8.5 Hz, 4H), 7.73 (d, J = 8.5 Hz, 4H), 3.92 (s, 3H).

^{13}C NMR (101 MHz, CDCl$_3$) δ 166.0, 150.0, 133.0, 130.8, 124.6, 52.7.

HRMS (ESI, m/z): Mass calcd. for $C_{16}H_{14}NaO_5S^+$ [M+Na]$^+$, 341.0454; found 341.0451.

V. Supplementary Figures

^{1}H NMR, ^{13}C NMR and ^{19}F NMR spectra

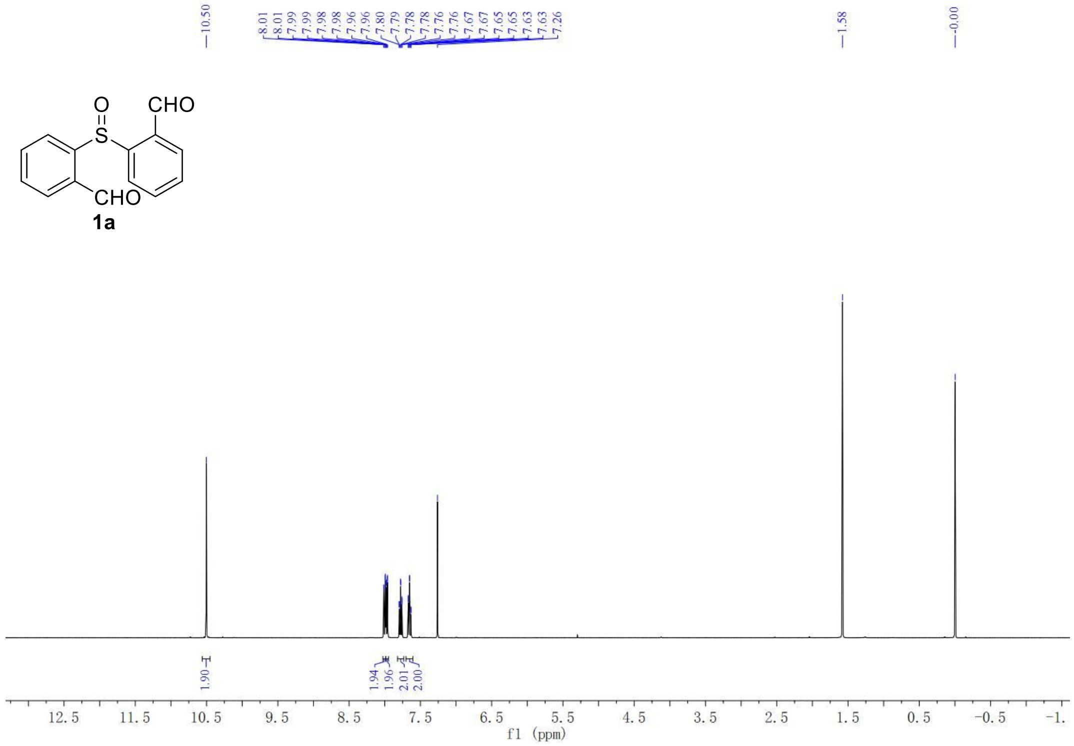

Supplementary Figure 4 ^{1}H NMR (400 MHz, $CDCl_3$) of **1a**

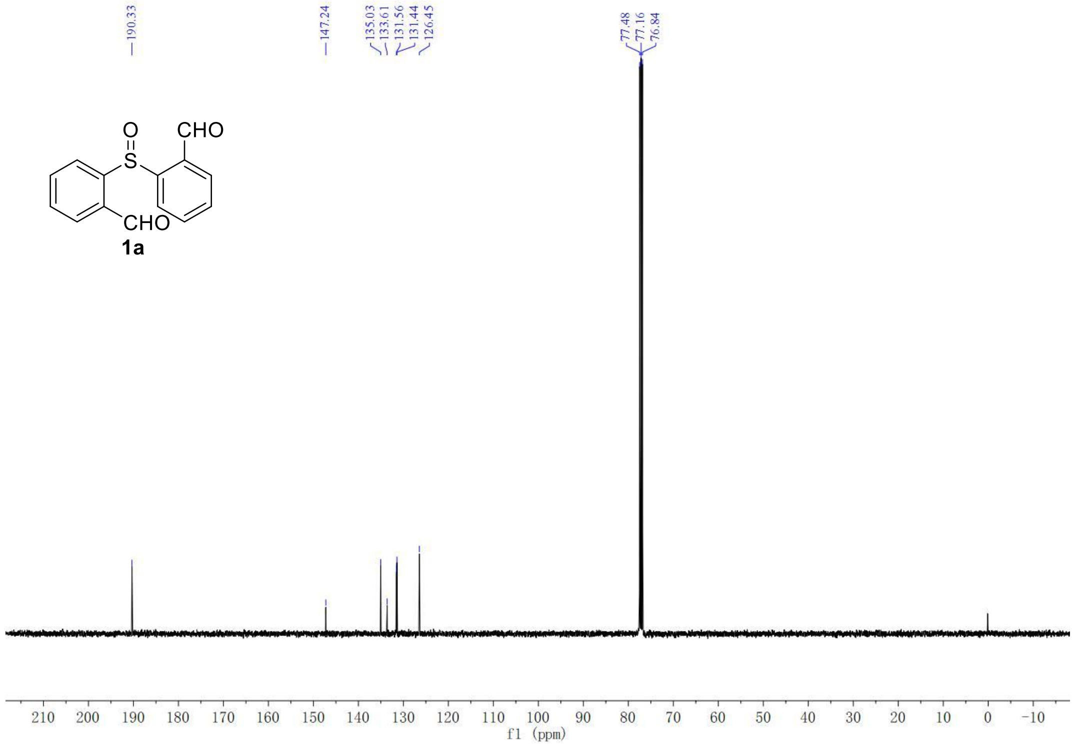

Supplementary Figure 5 ^{13}C NMR (101 MHz, $CDCl_3$) of **1a**

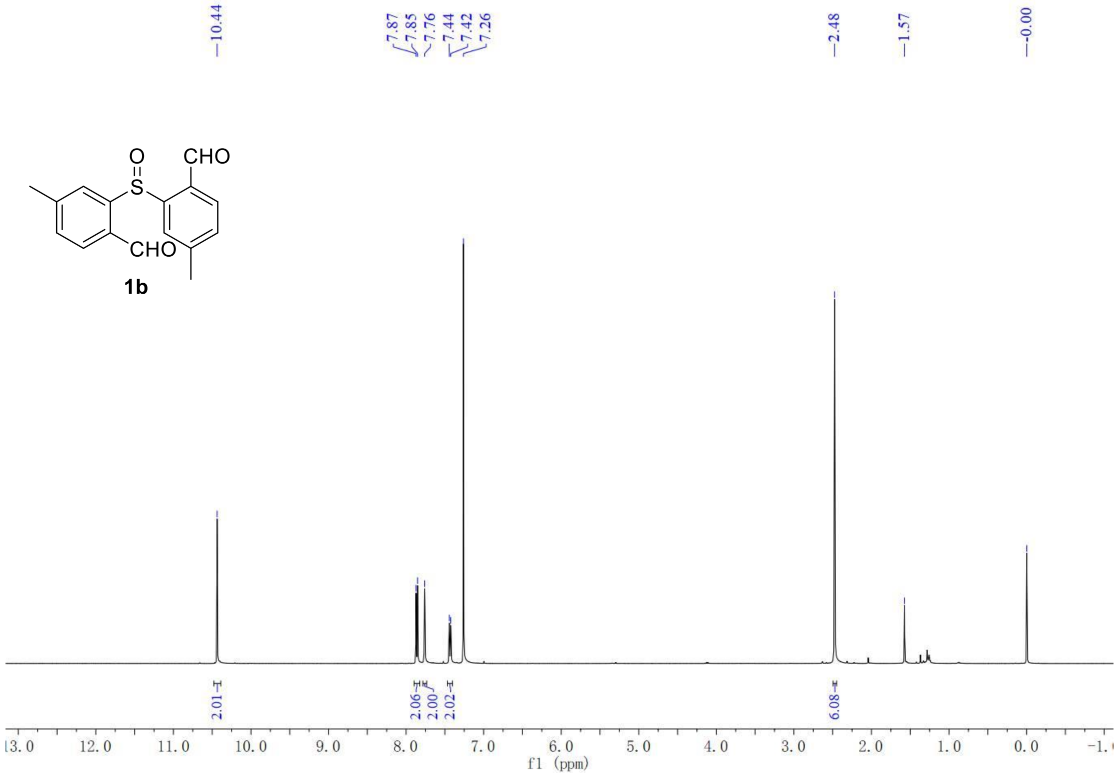

Supplementary Figure 6 ^{1}H NMR (400 MHz, $CDCl_3$) of **1b**

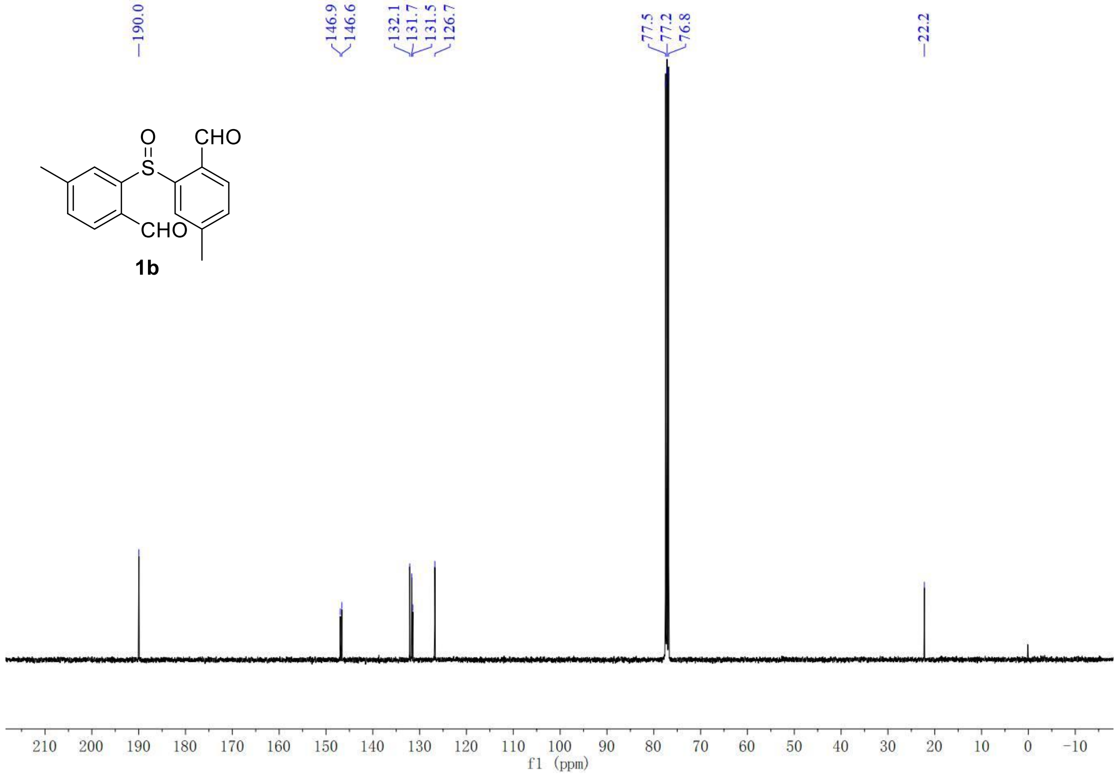

Supplementary Figure 7 ^{13}C NMR (101 MHz, $CDCl_3$) of **1b**

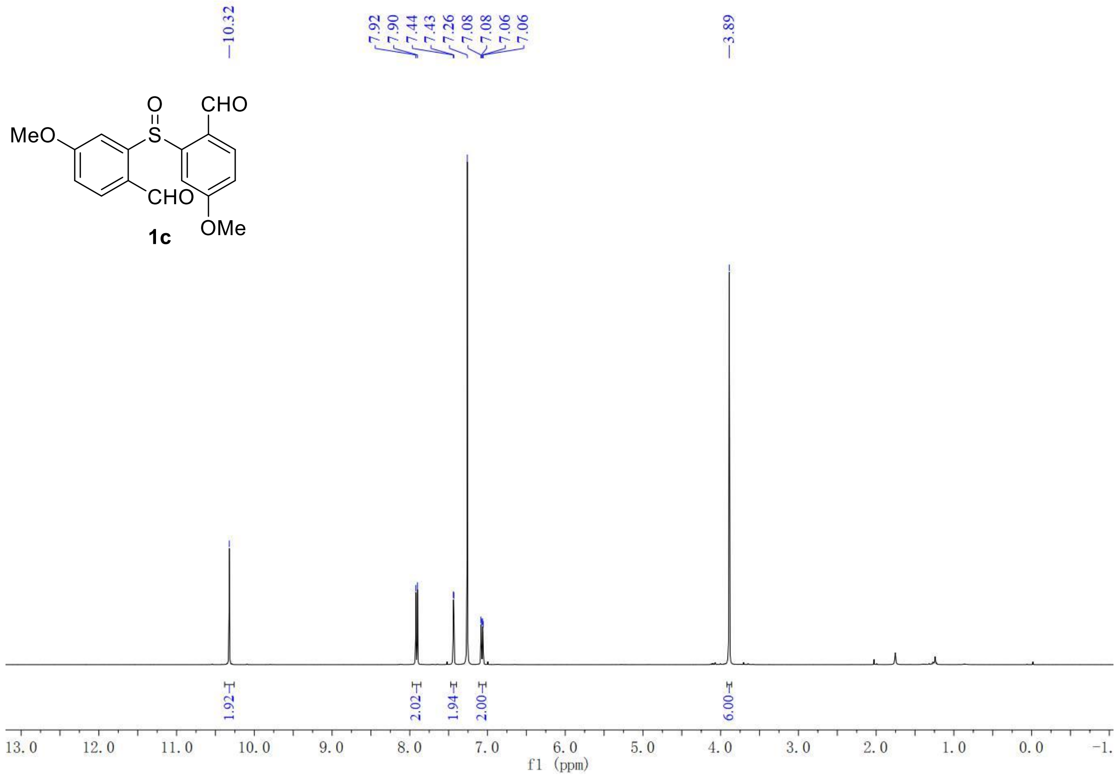

Supplementary Figure 8 ^{1}H NMR (400 MHz, $CDCl_3$) of **1c**

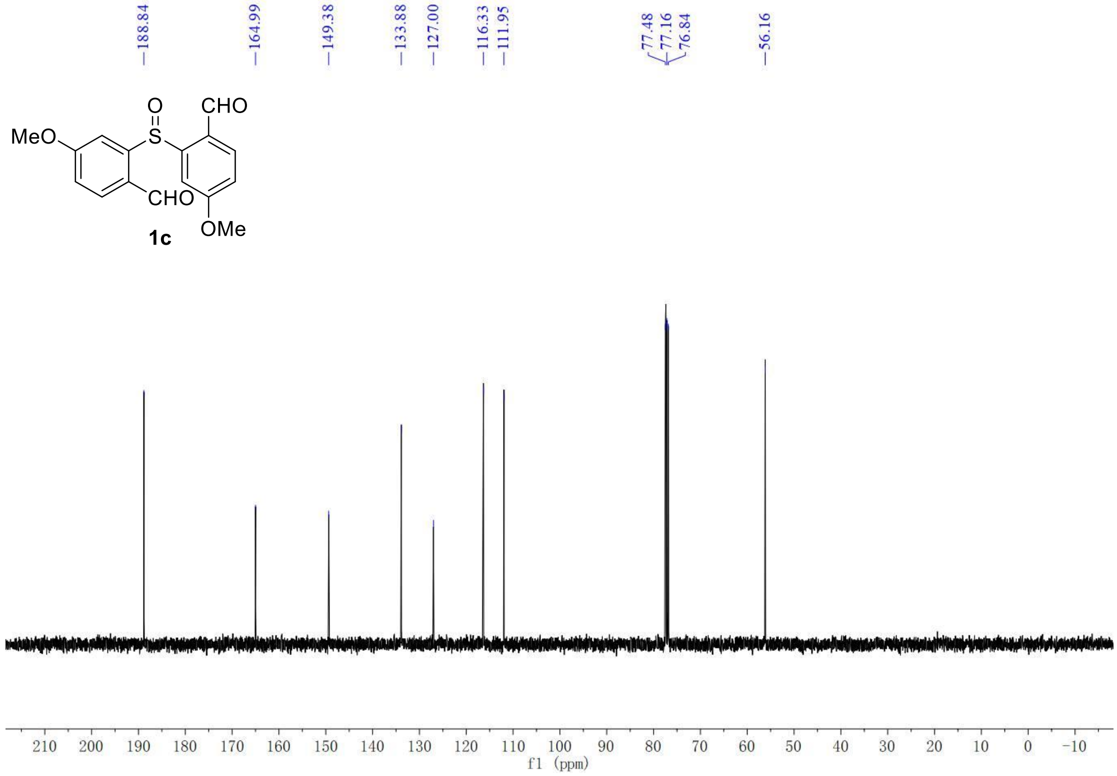

Supplementary Figure 9 ^{13}C NMR (101 MHz, $CDCl_3$) of **1c**

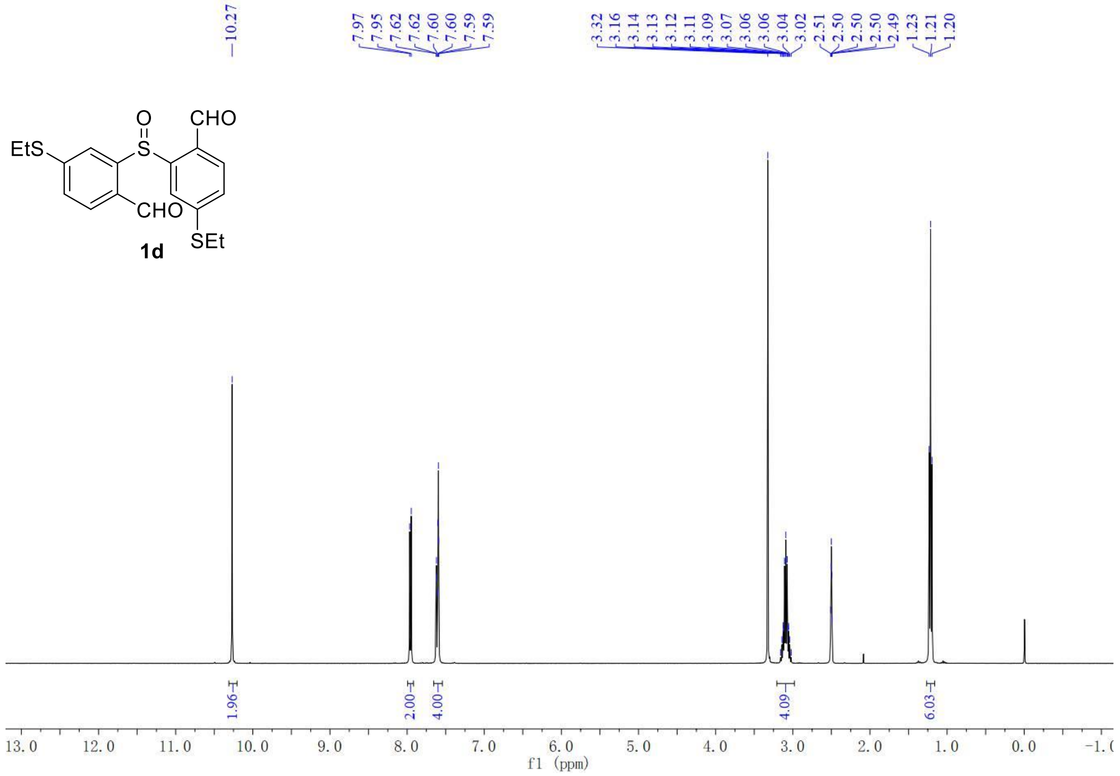

Supplementary Figure 10 ^{1}H NMR (400 MHz, DMSO-d_6) of **1d**

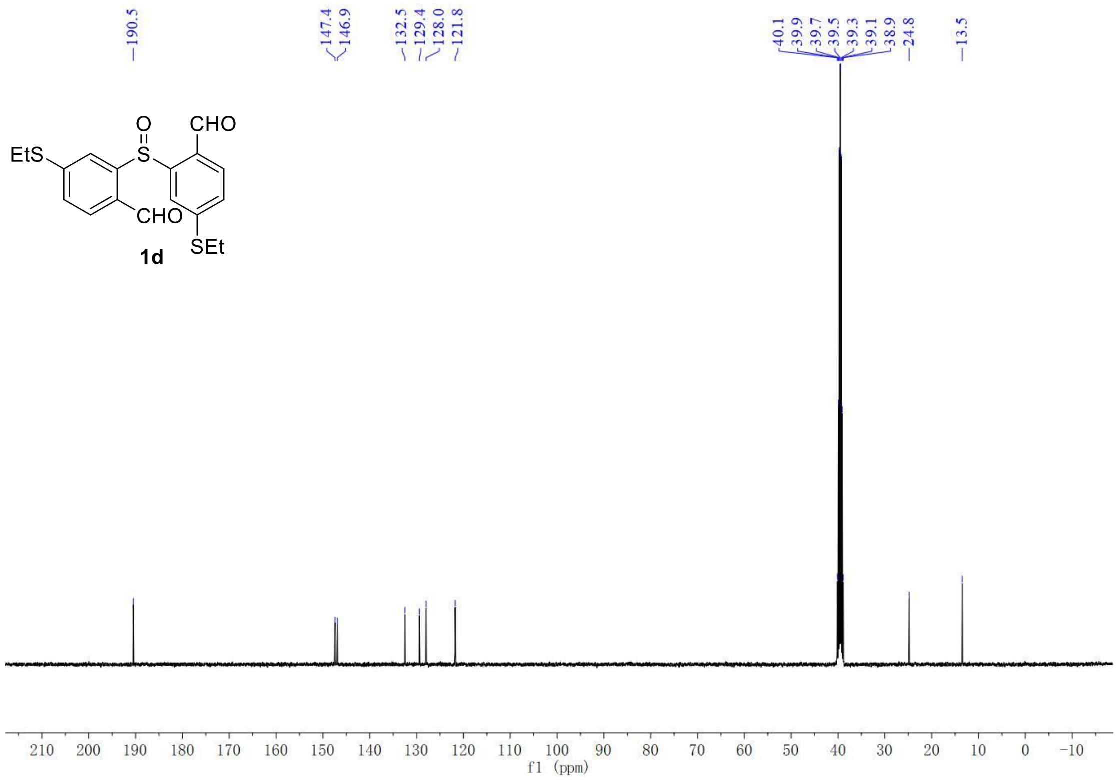

Supplementary Figure 11 ^{13}C NMR (101 MHz, DMSO-d_6) of **1d**

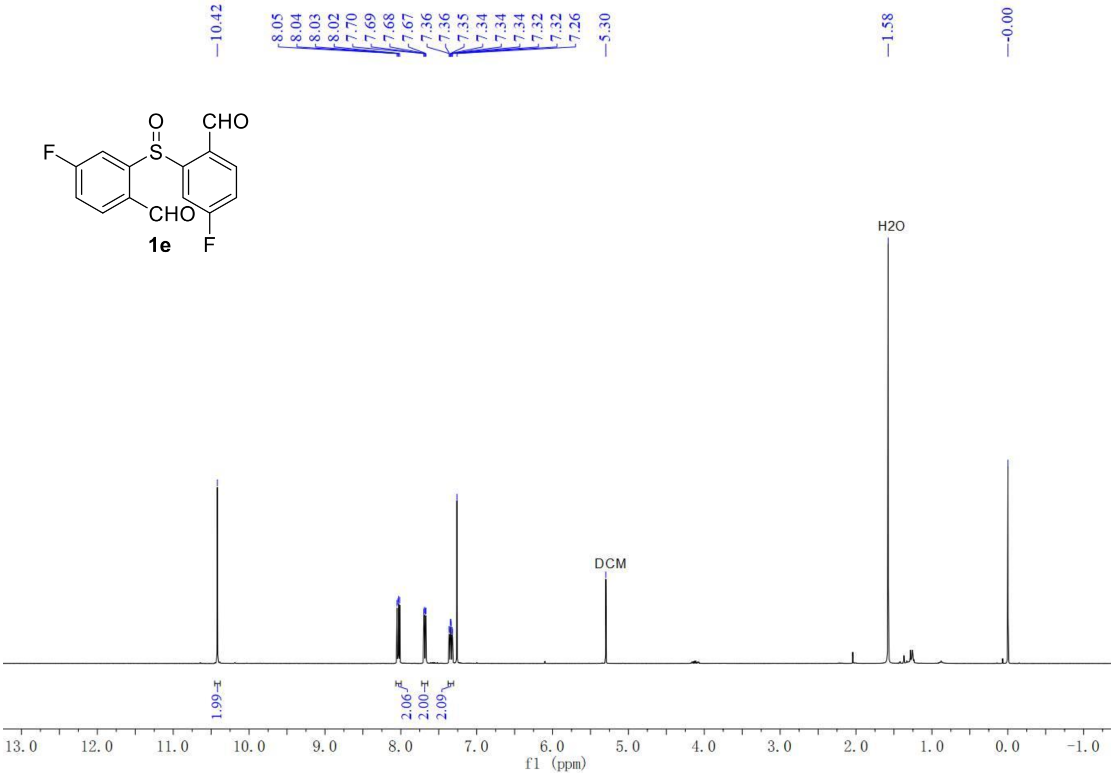

Supplementary Figure 12 ^{1}H NMR (400 MHz, $CDCl_3$) of **1e**

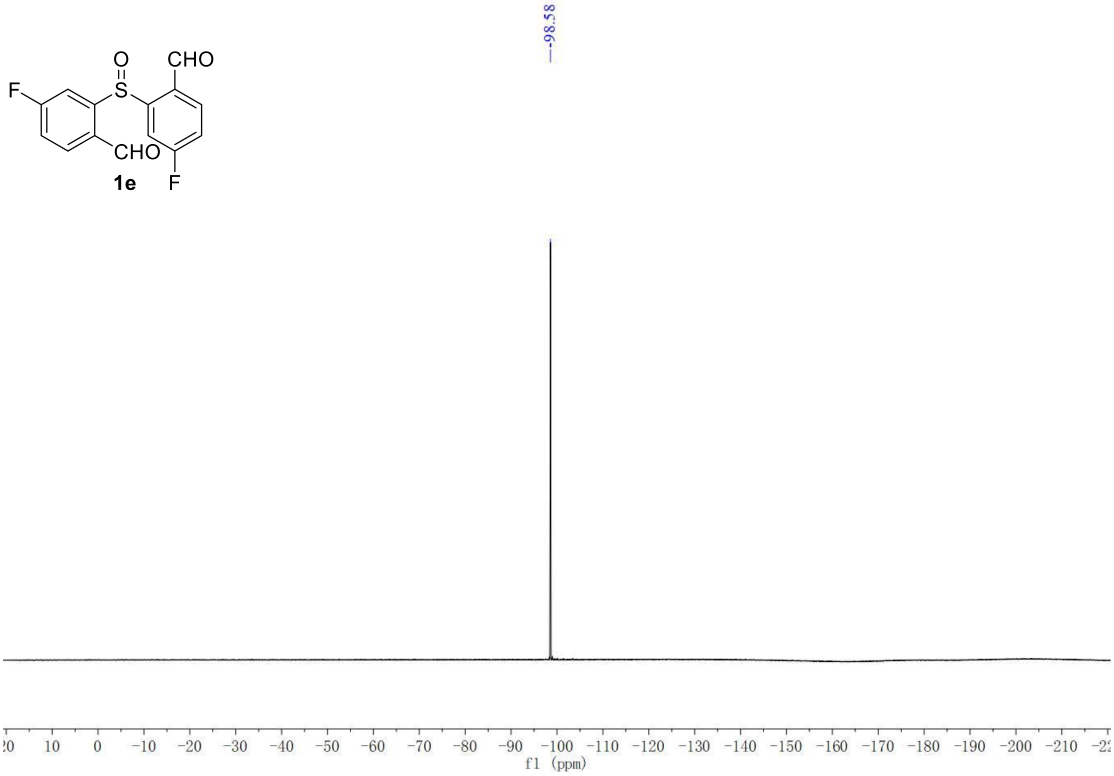

Supplementary Figure 13 ^{19}F NMR (376 MHz, $CDCl_3$) of **1e**

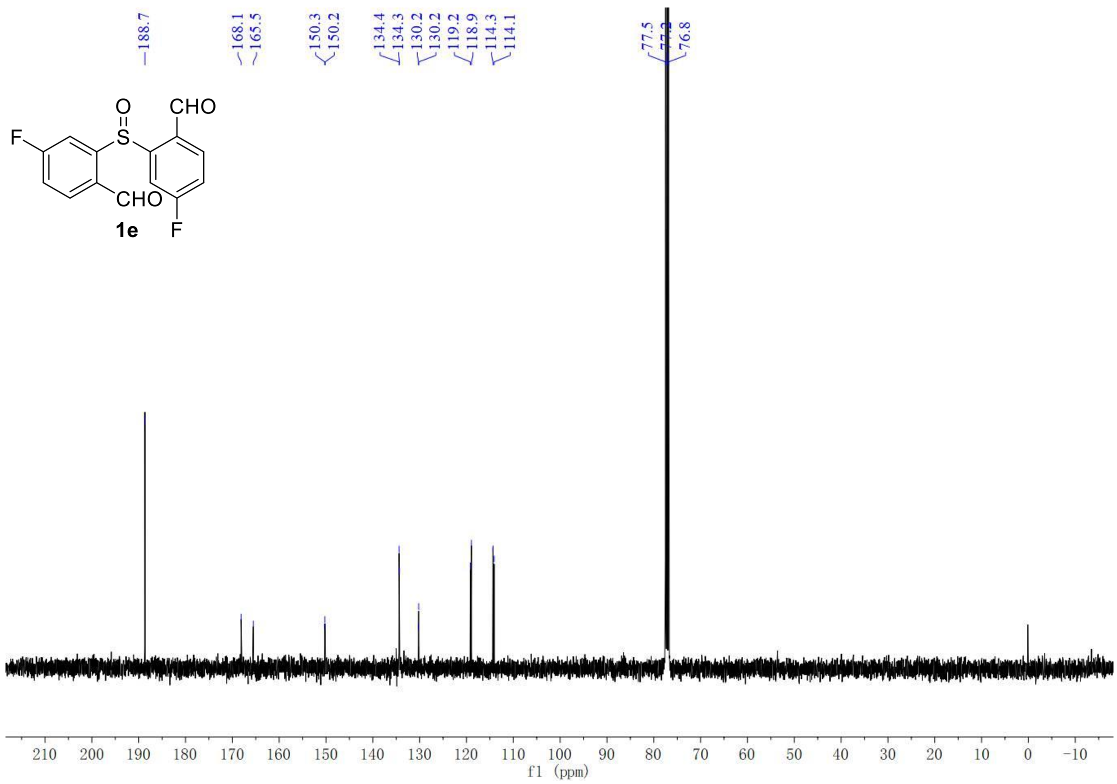

Supplementary Figure 14 ^{13}C NMR (101 MHz, $CDCl_3$) of **1e**

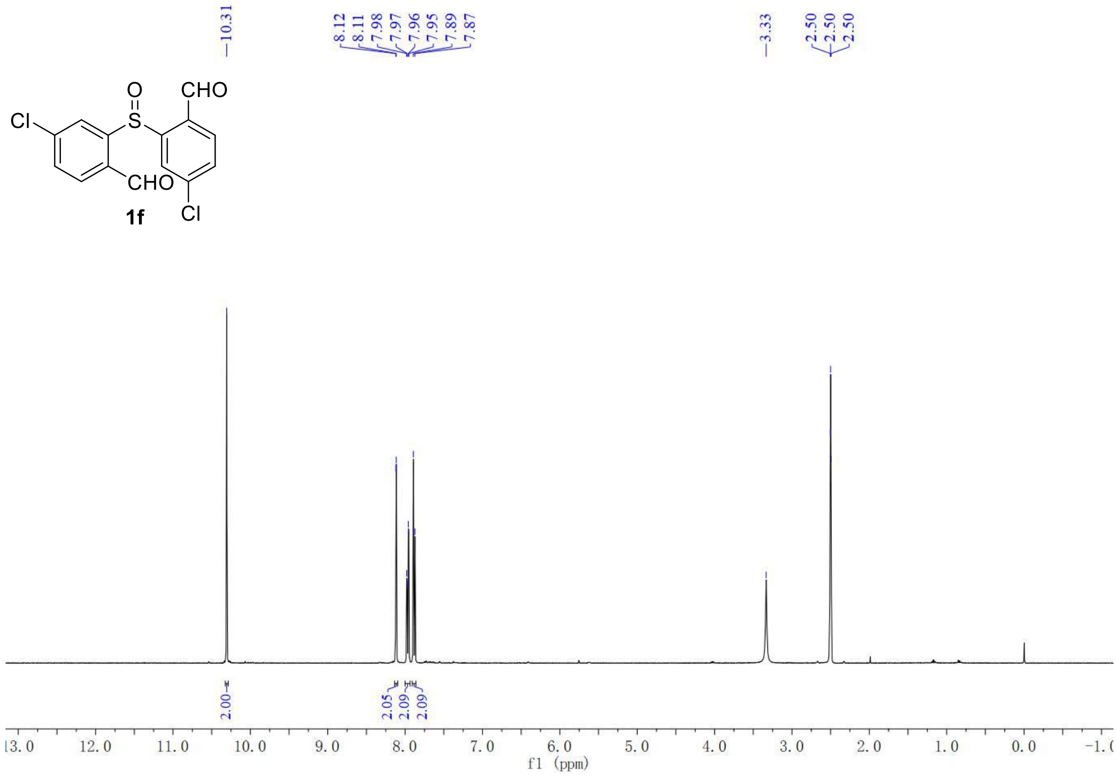

Supplementary Figure 15 ^{1}H NMR (400 MHz, DMSO-d_6) of **1f**

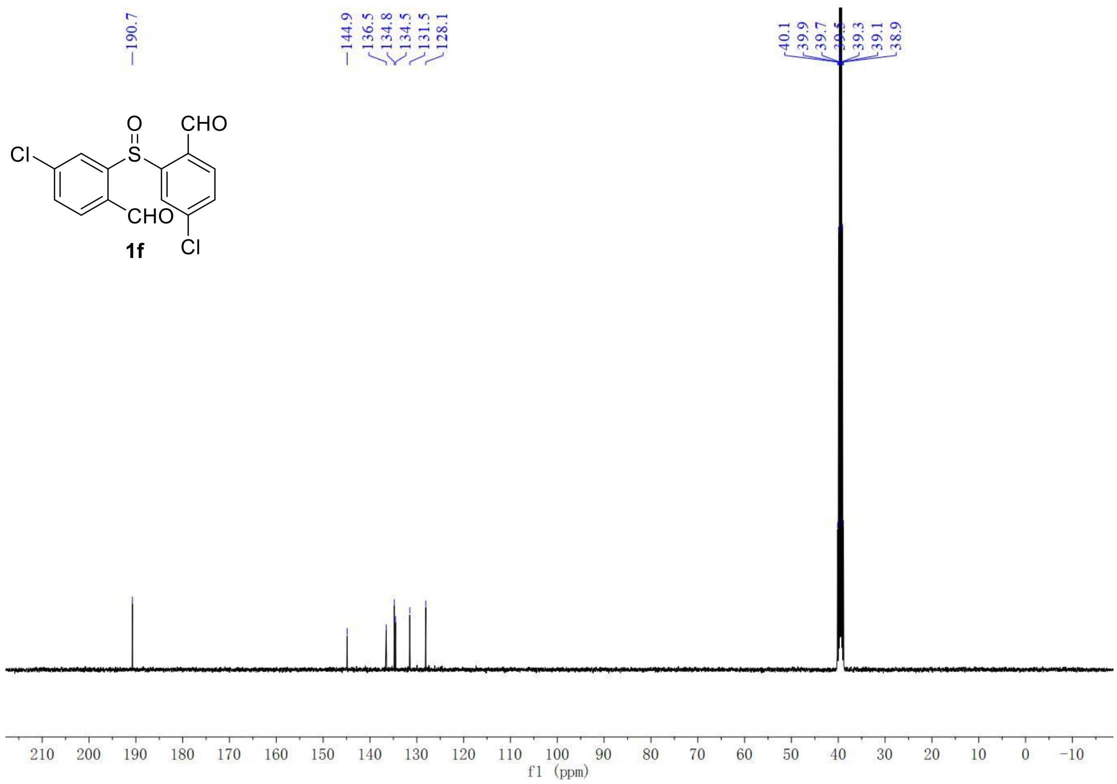

Supplementary Figure 16 ^{13}C NMR (101 MHz, DMSO-d_6) of **1f**

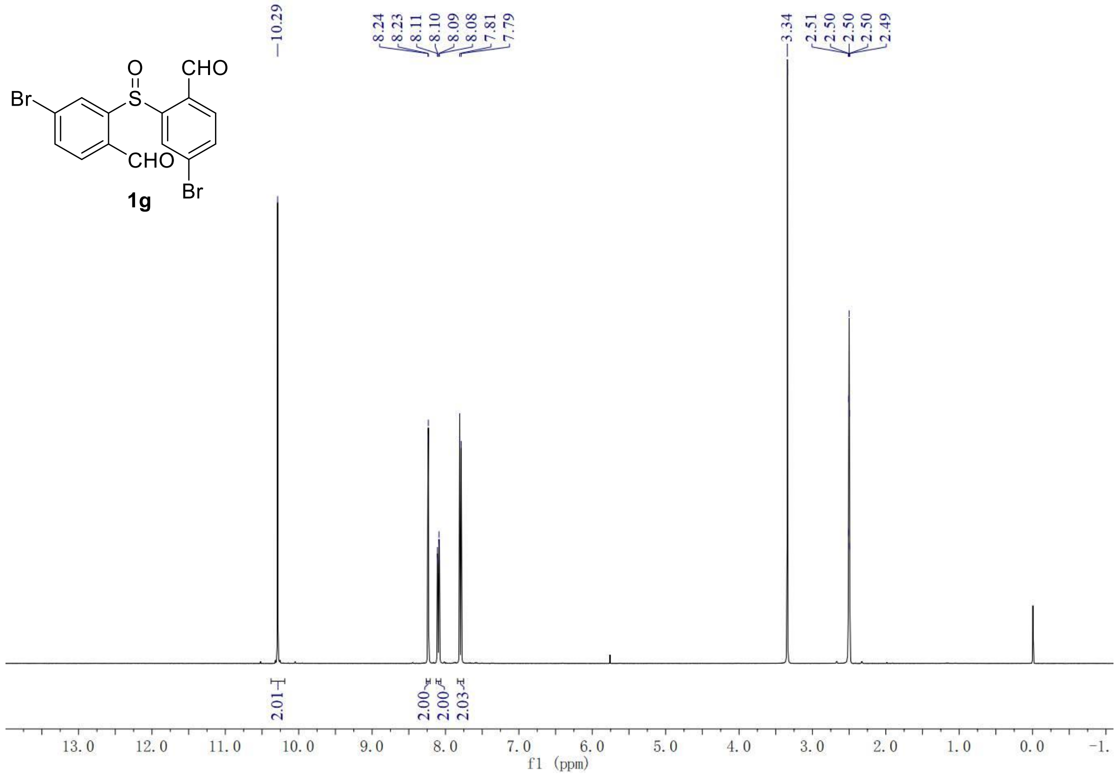

Supplementary Figure 17 ^{1}H NMR (400 MHz, DMSO-d_6) of **1g**

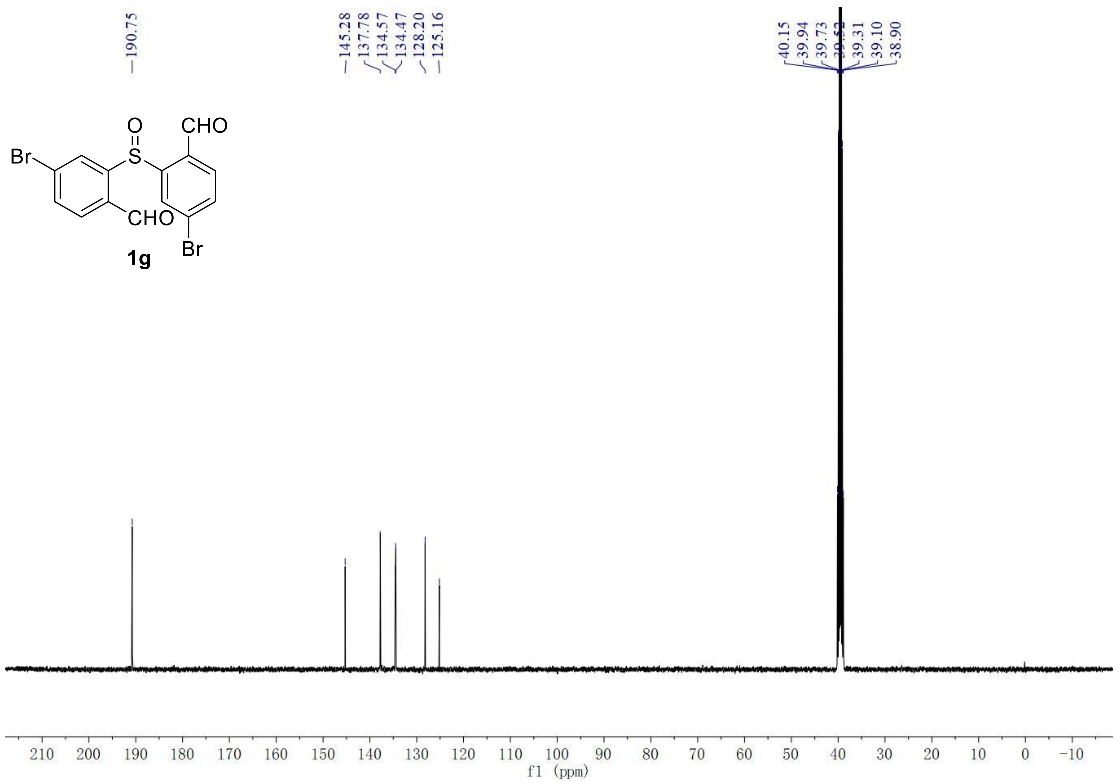

Supplementary Figure 18 ^{13}C NMR (101 MHz, DMSO-d_6) of **1g**

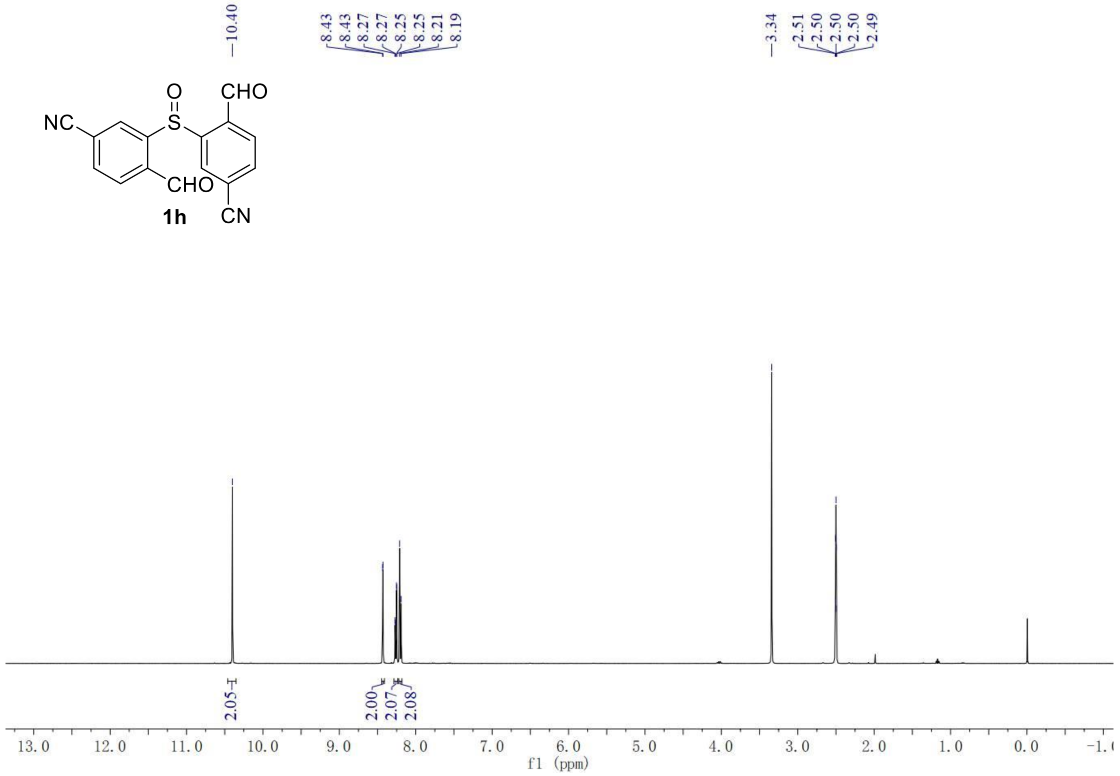

Supplementary Figure 19 ^{1}H NMR (400 MHz, DMSO-d_6) of **1h**

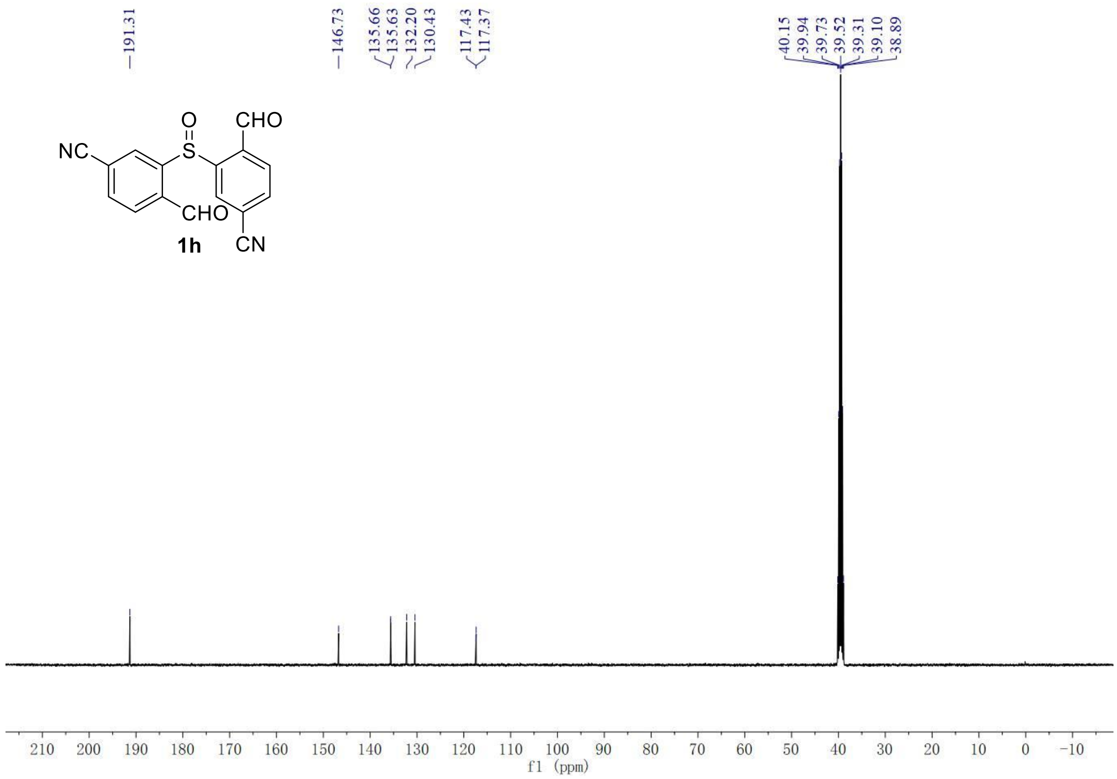

Supplementary Figure 20 ^{13}C NMR (101 MHz, DMSO-d_6) of **1h**

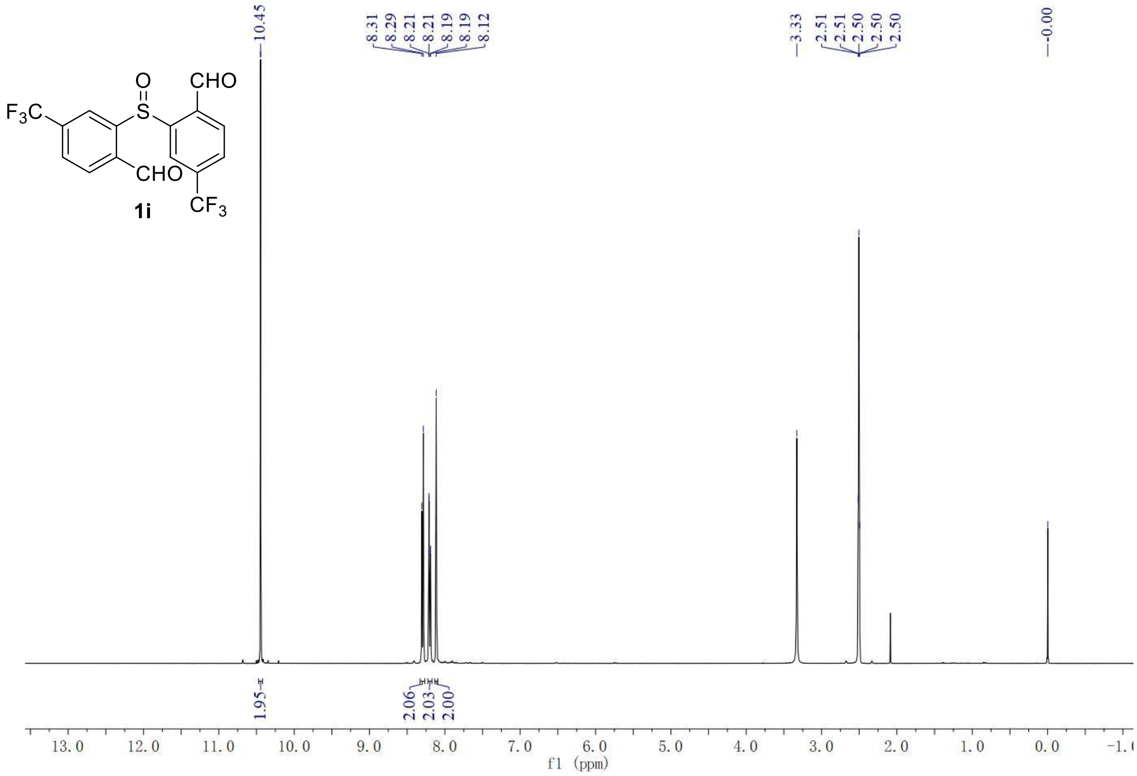

Supplementary Figure 21 ^{1}H NMR (400 MHz, DMSO-d_6) of **1i**

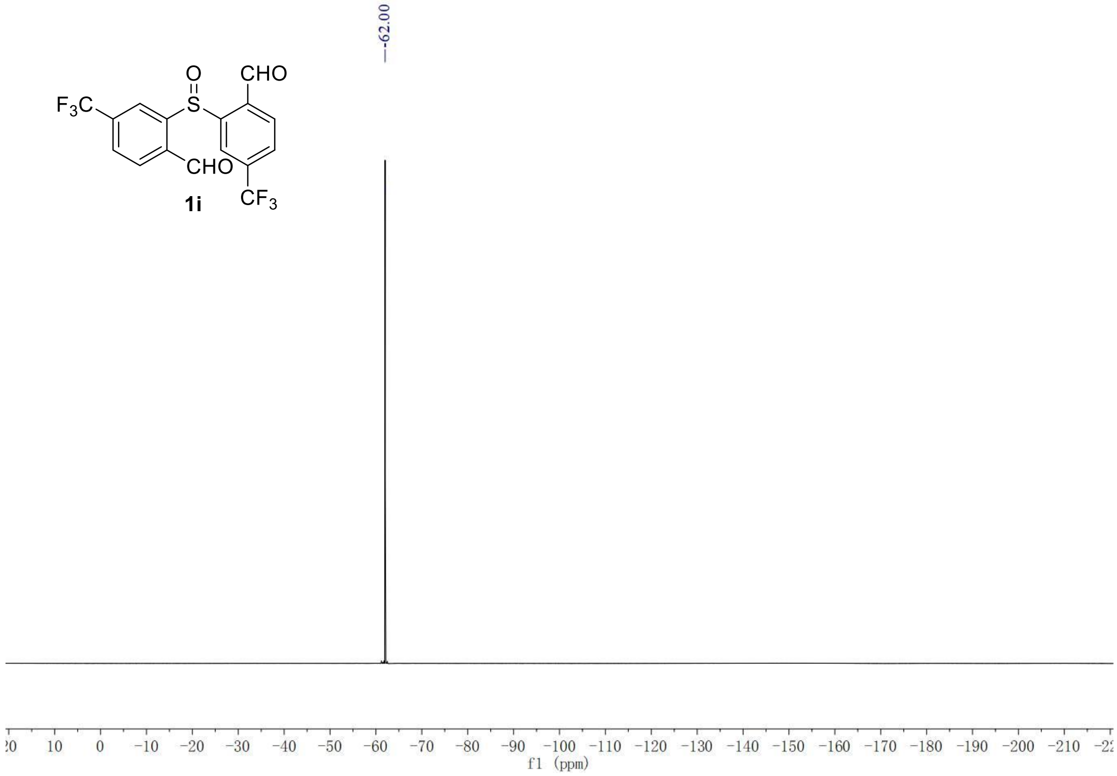

Supplementary Figure 22 ^{19}F NMR (376 MHz, DMSO-d_6) of **1i**

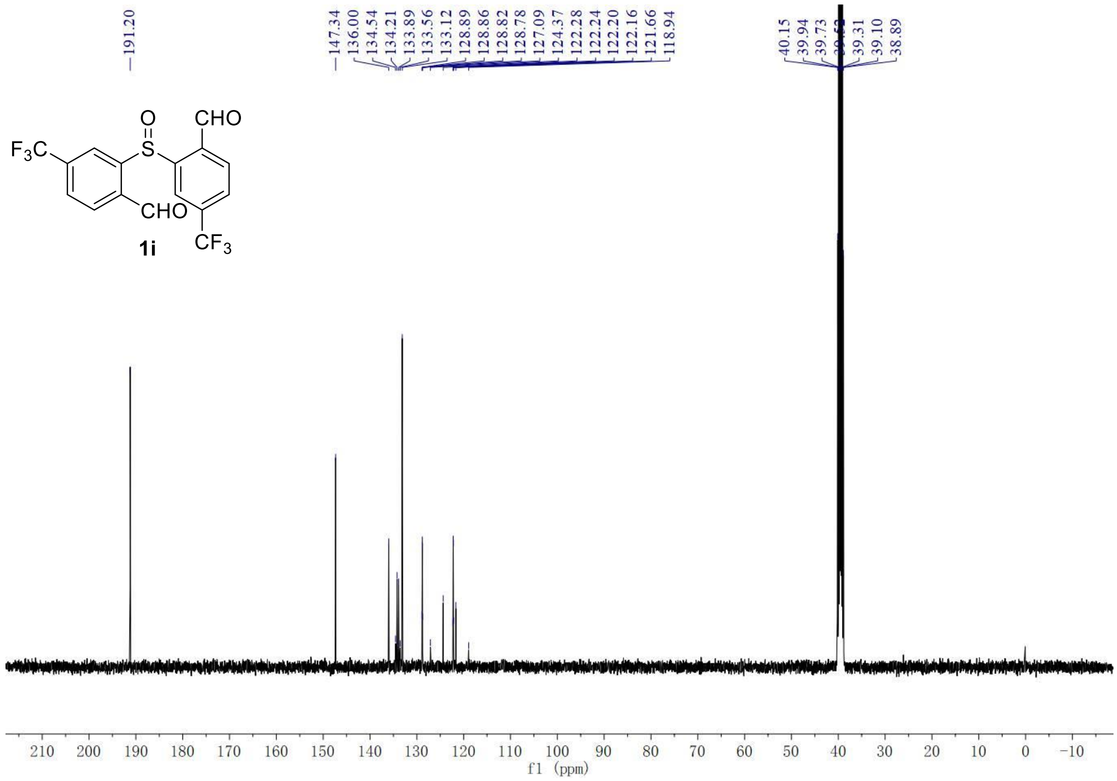

Supplementary Figure 23 ^{13}C NMR (101 MHz, DMSO-d_6) of **1i**

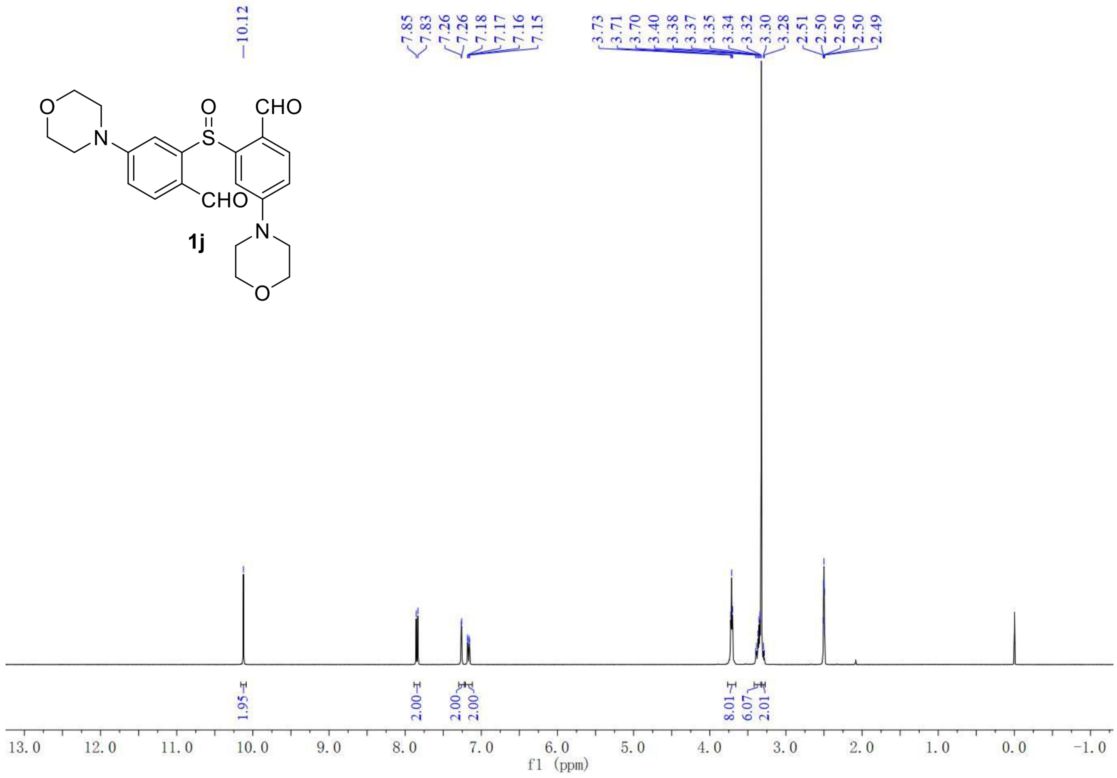

Supplementary Figure 24 ^{1}H NMR (400 MHz, DMSO-d_6) of **1j**

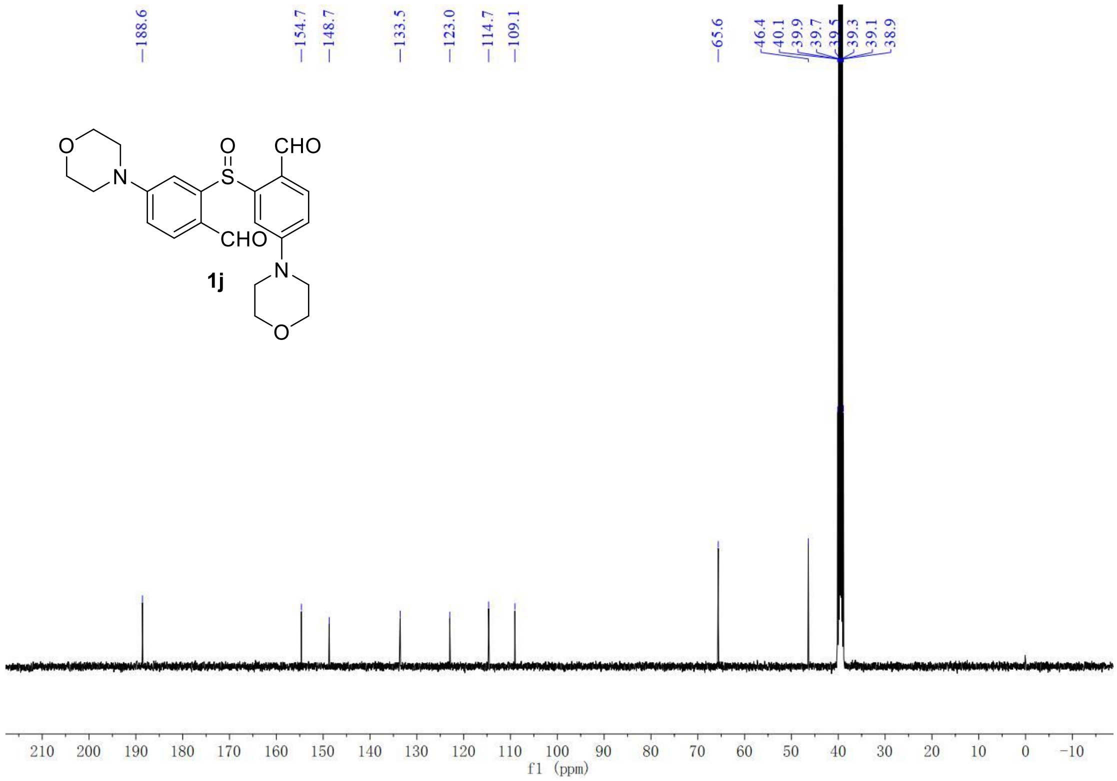

Supplementary Figure 25 ^{13}C NMR (101 MHz, DMSO-d_6) of **1j**

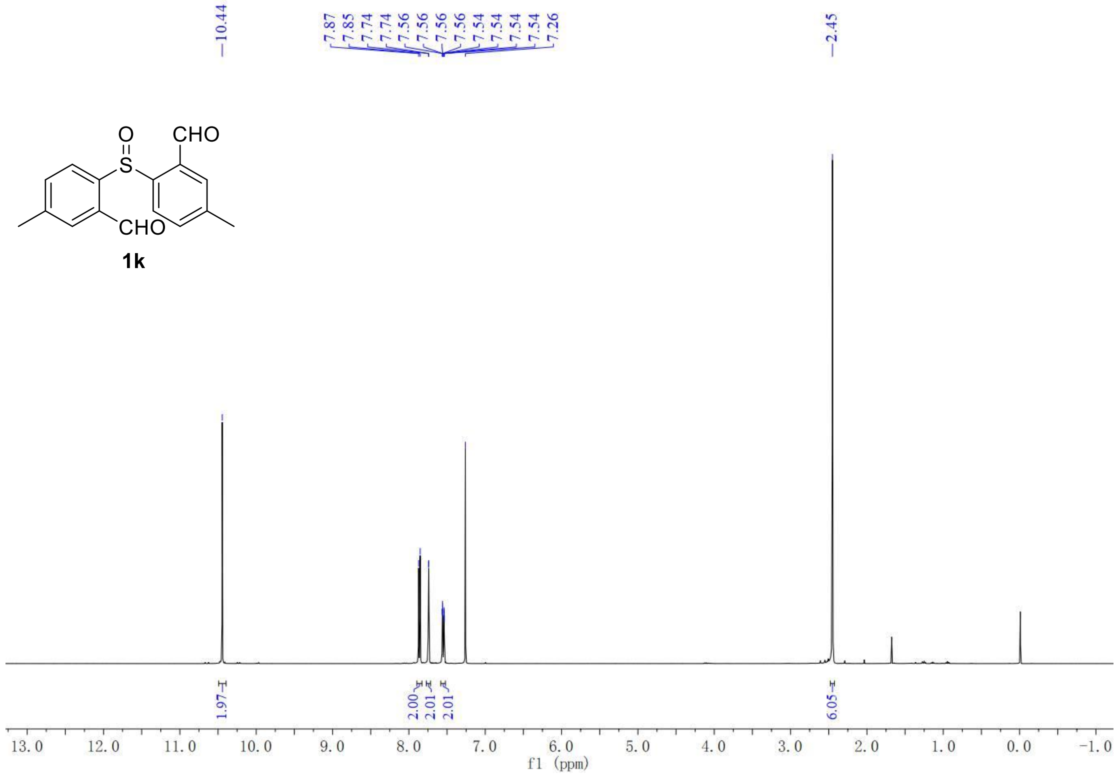

Supplementary Figure 26 ^{1}H NMR (400 MHz, $CDCl_3$) of **1k**

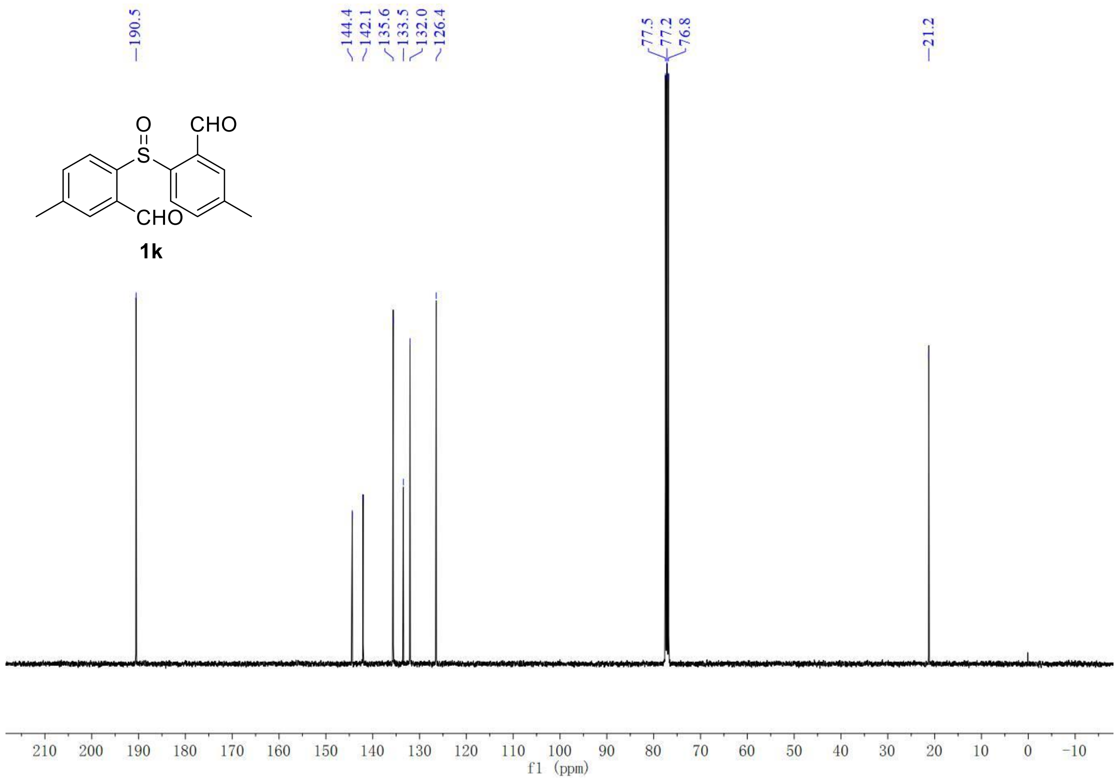

Supplementary Figure 27 ^{13}C NMR (101 MHz, $CDCl_3$) of **1k**

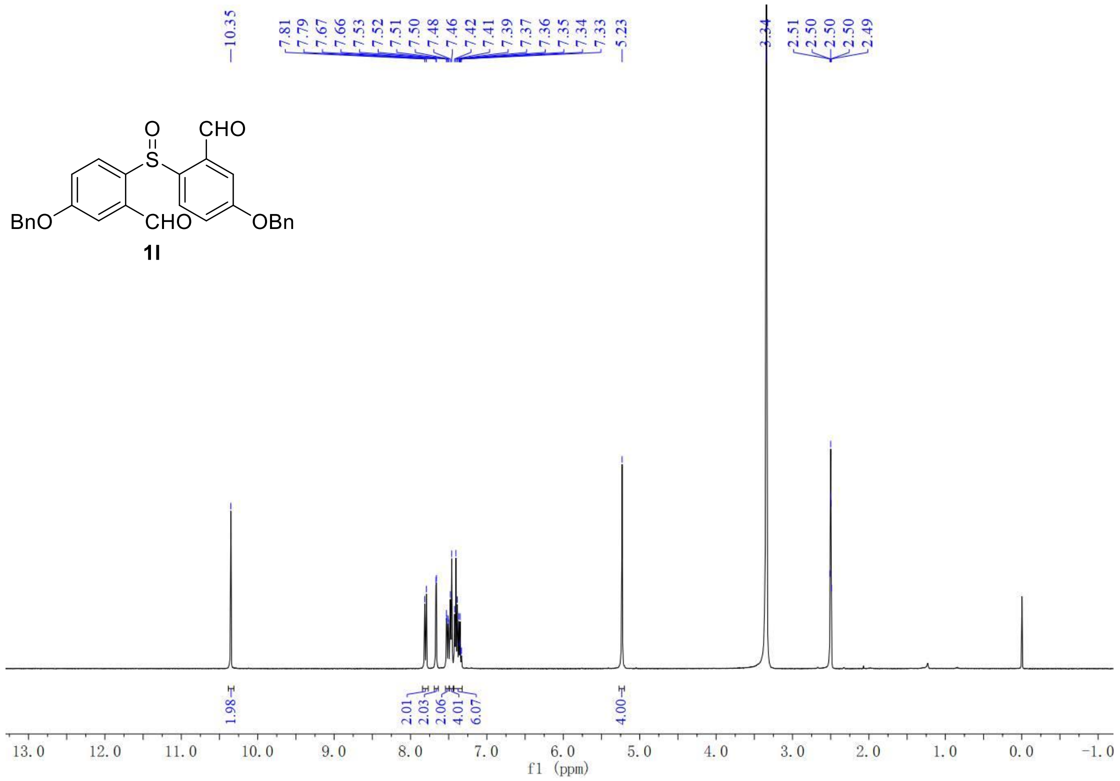

Supplementary Figure 28 ^{1}H NMR (400 MHz, DMSO-d_6) of **1l**

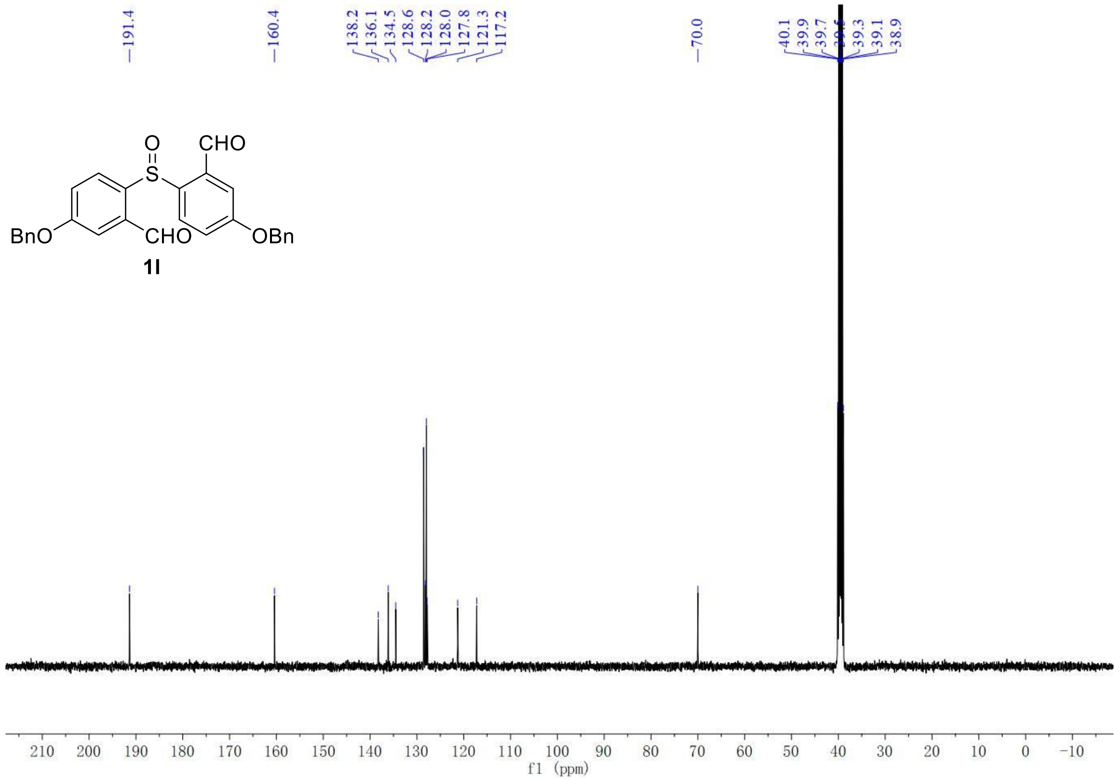

Supplementary Figure 29 ^{13}C NMR (101 MHz, DMSO-d_6) of **1l**

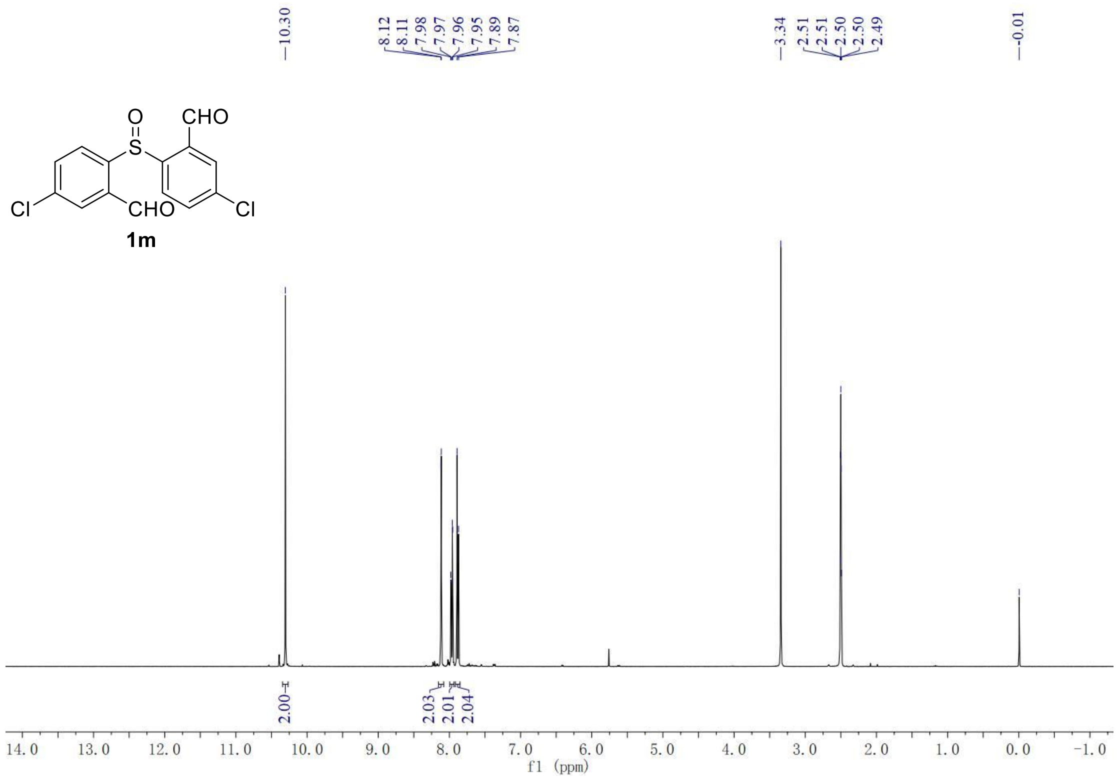

Supplementary Figure 30 ^{1}H NMR (400 MHz, DMSO-d_6) of **1m**

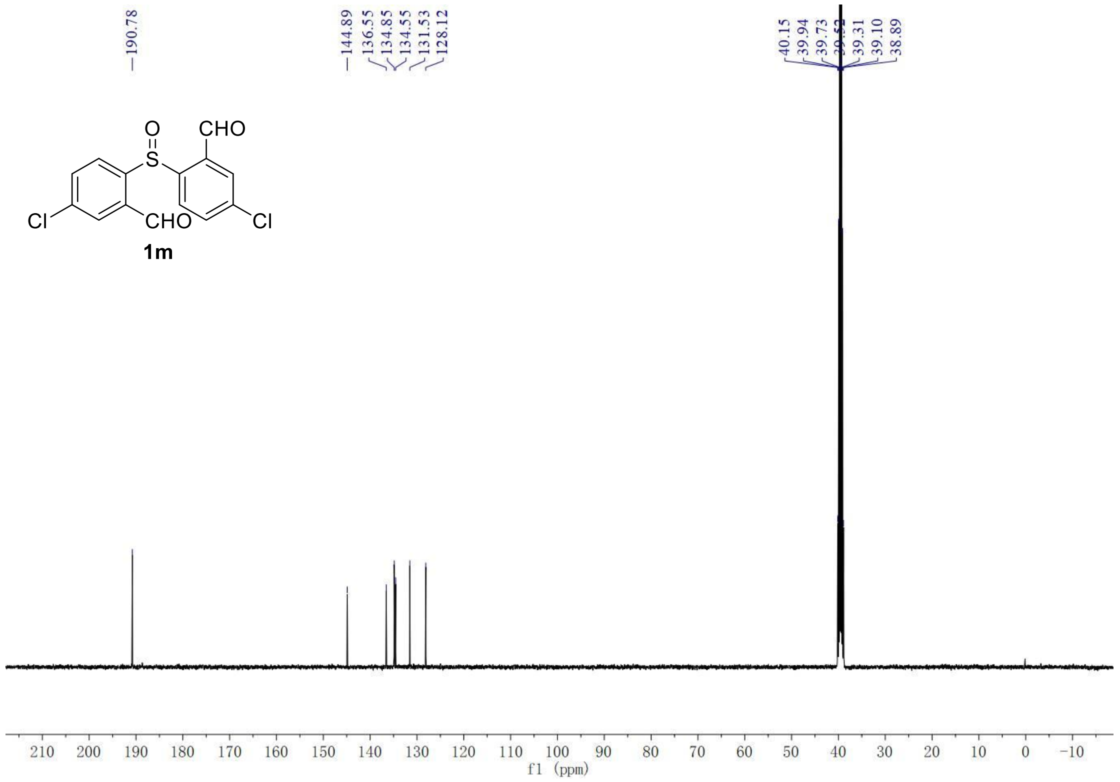

Supplementary Figure 31 ^{13}C NMR (101 MHz, DMSO-d_6) of **1m**

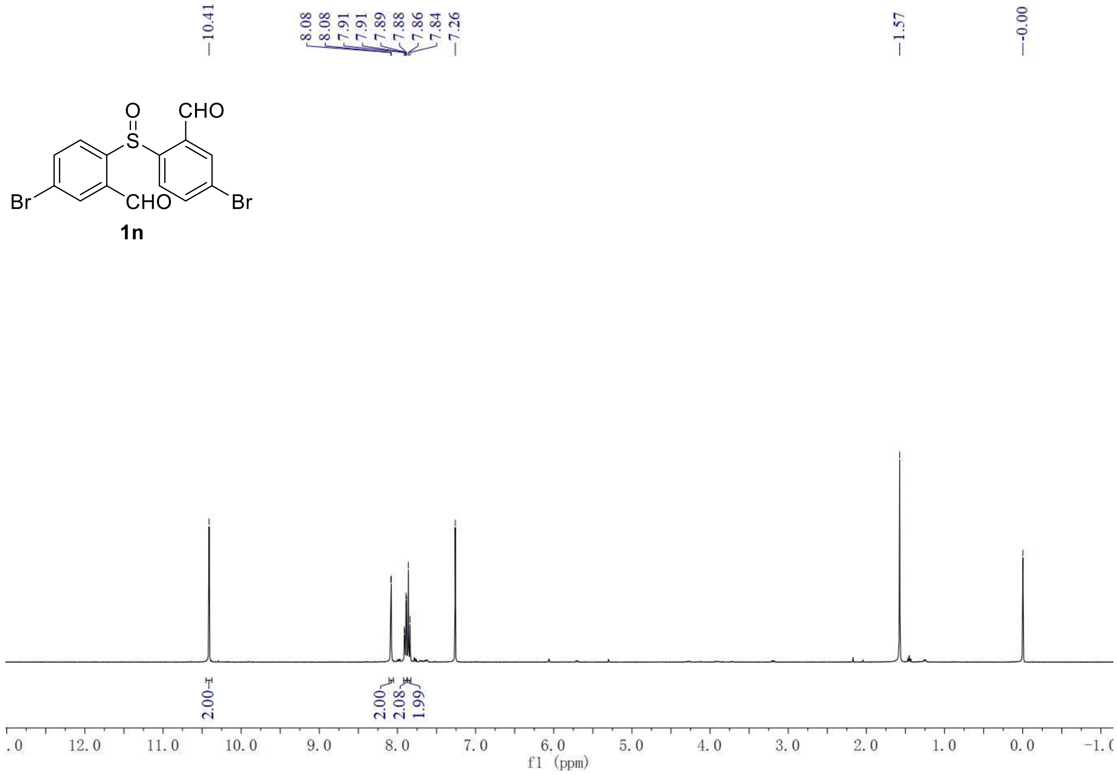

Supplementary Figure 32 ^{1}H NMR (400 MHz, $CDCl_3$) of **1n**

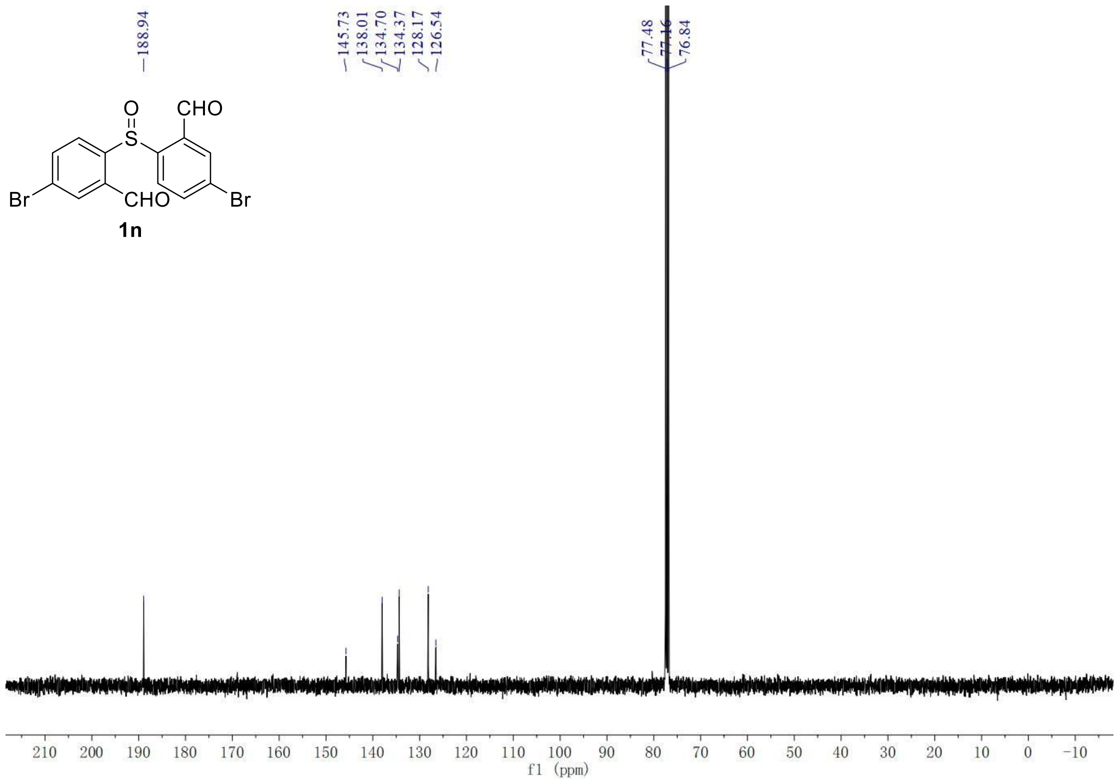

Supplementary Figure 33 ^{13}C NMR (101 MHz, $CDCl_3$) of **1n**

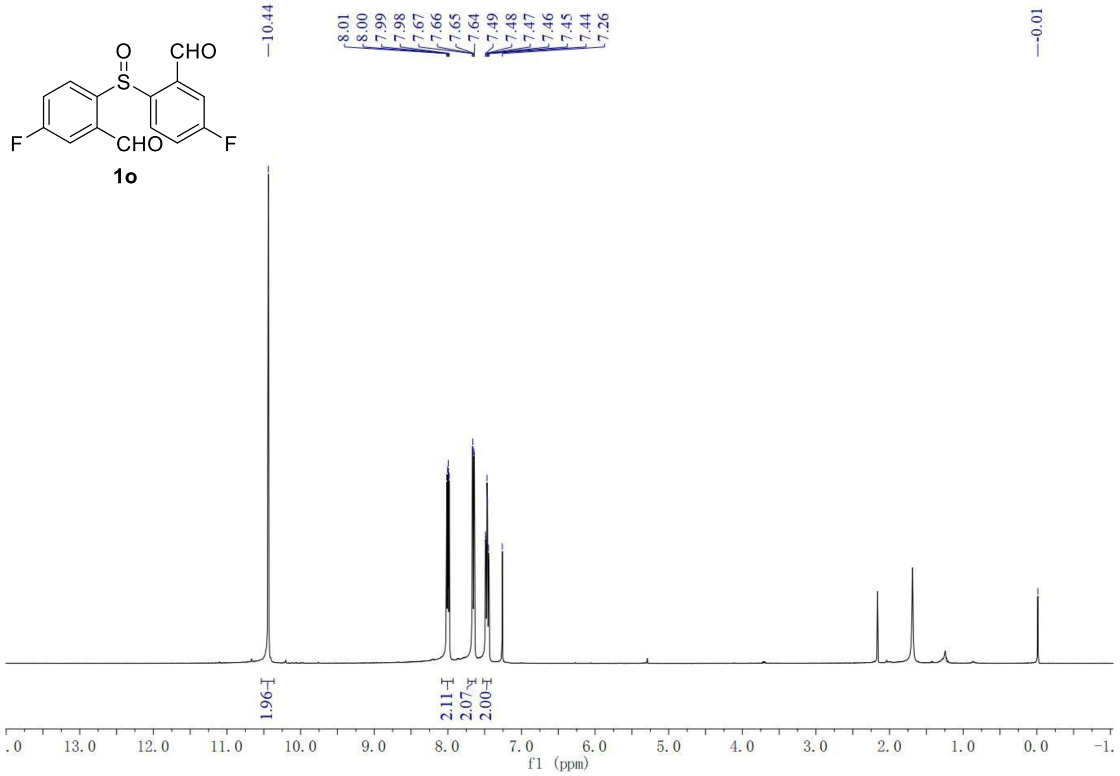

Supplementary Figure 34 ^{1}H NMR (400 MHz, $CDCl_3$) of **1o**

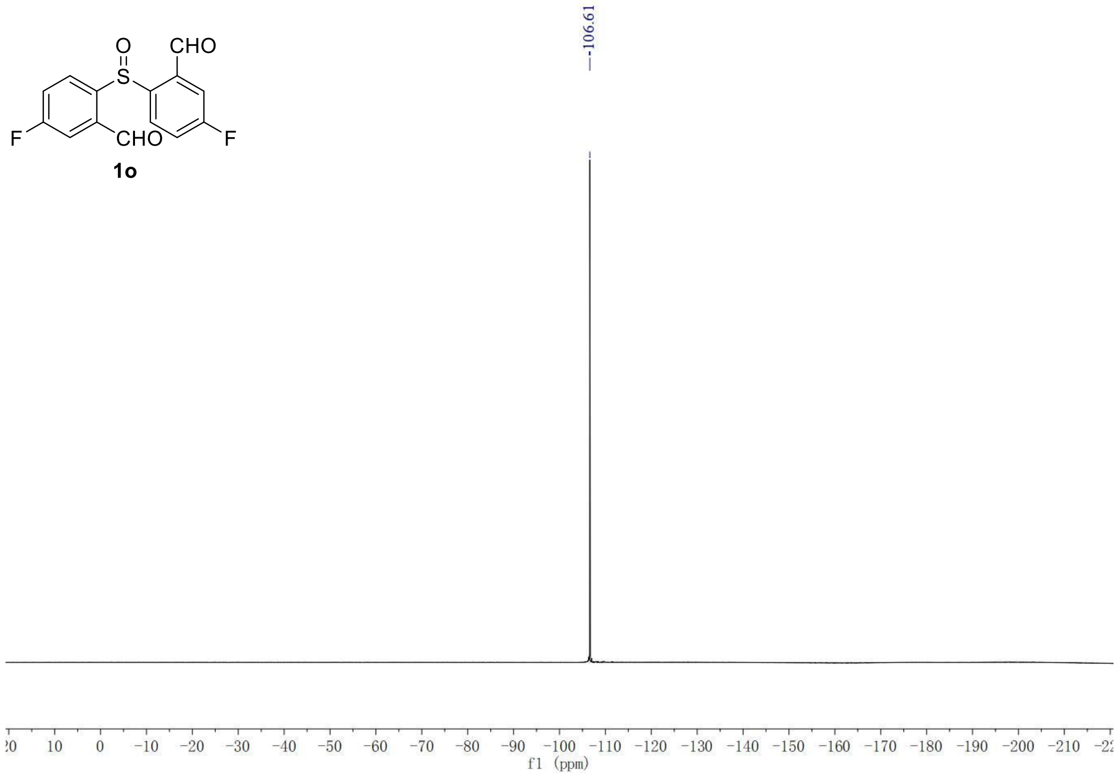

Supplementary Figure 35 ^{19}F NMR (376 MHz, $CDCl_3$) of **1o**

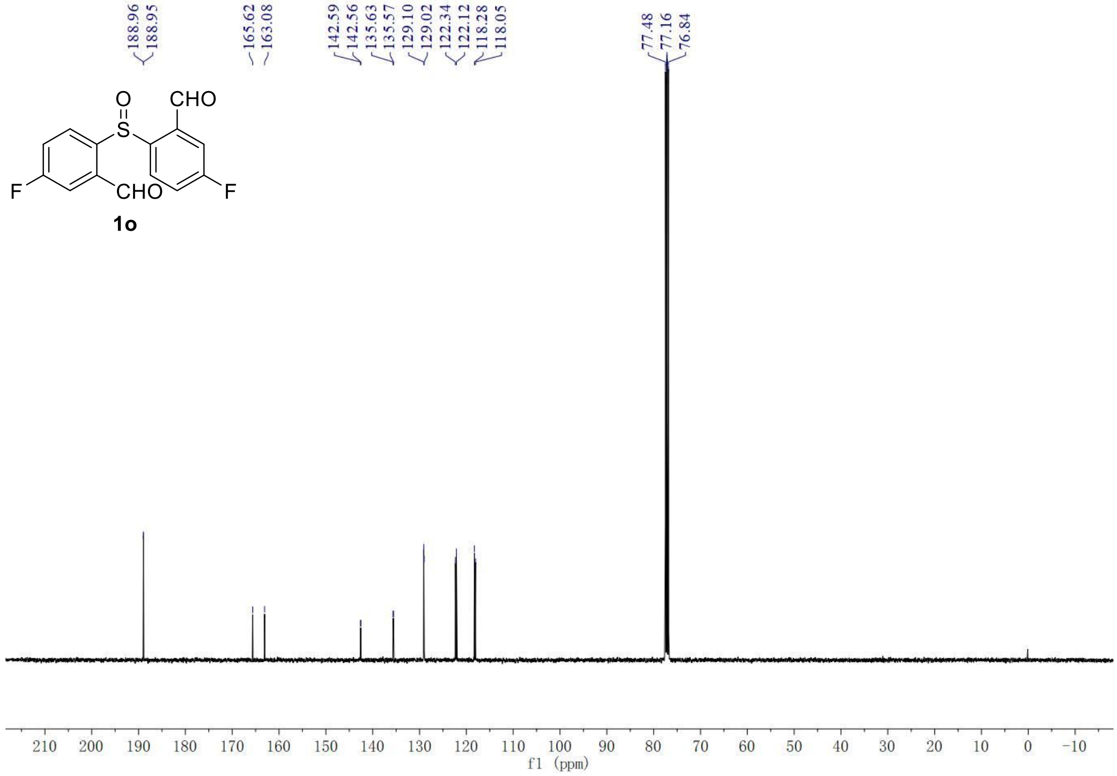

Supplementary Figure 36 ^{13}C NMR (101 MHz, $CDCl_3$) of **1o**

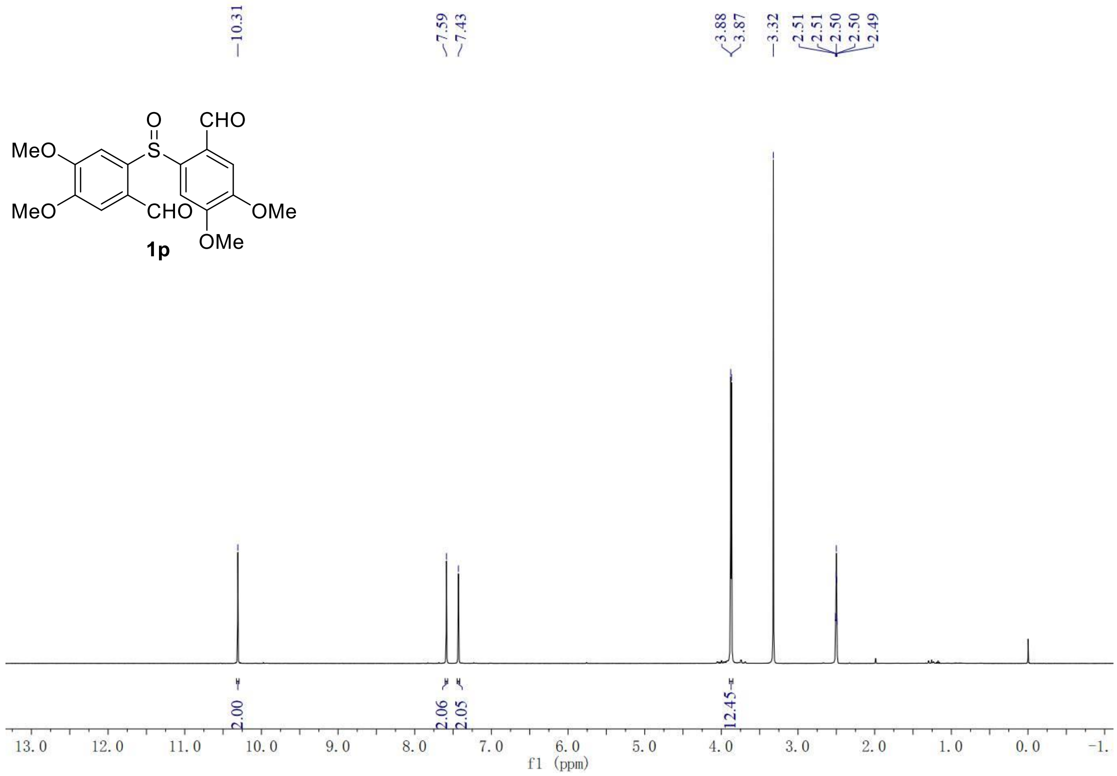

Supplementary Figure 37 ^{1}H NMR (400 MHz, DMSO-d_6) of **1p**

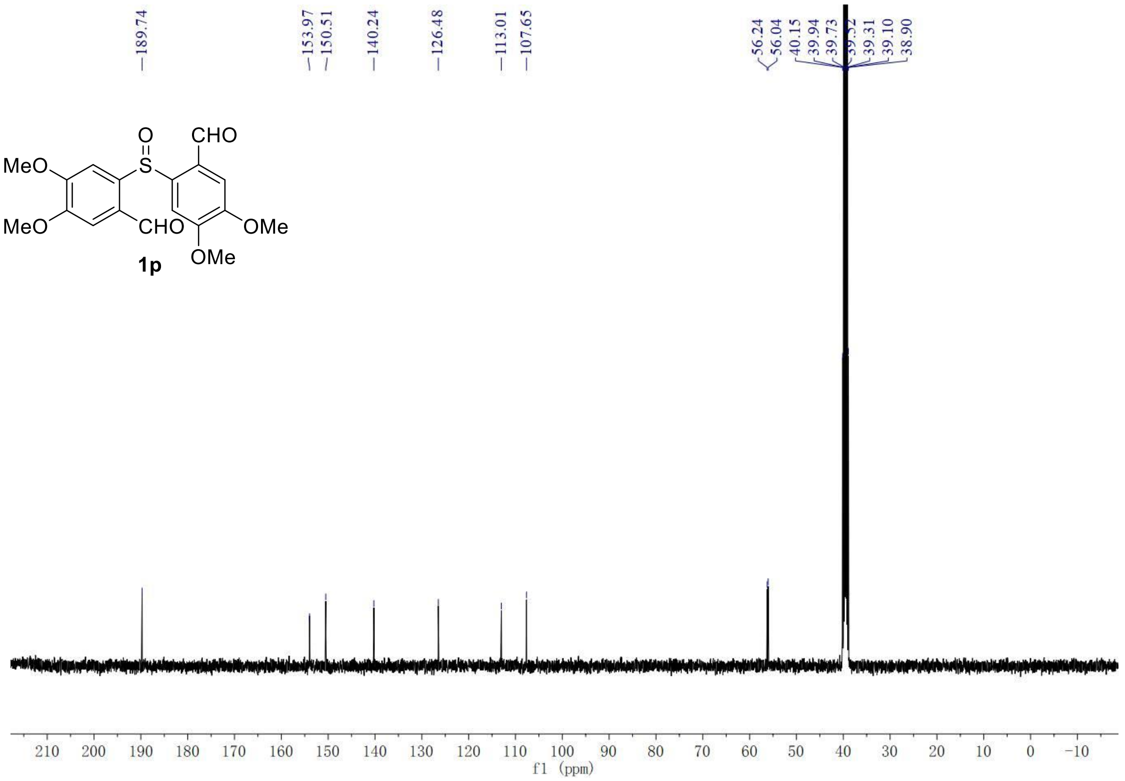

Supplementary Figure 38 ^{13}C NMR (101 MHz, DMSO-d_6) of **1p**

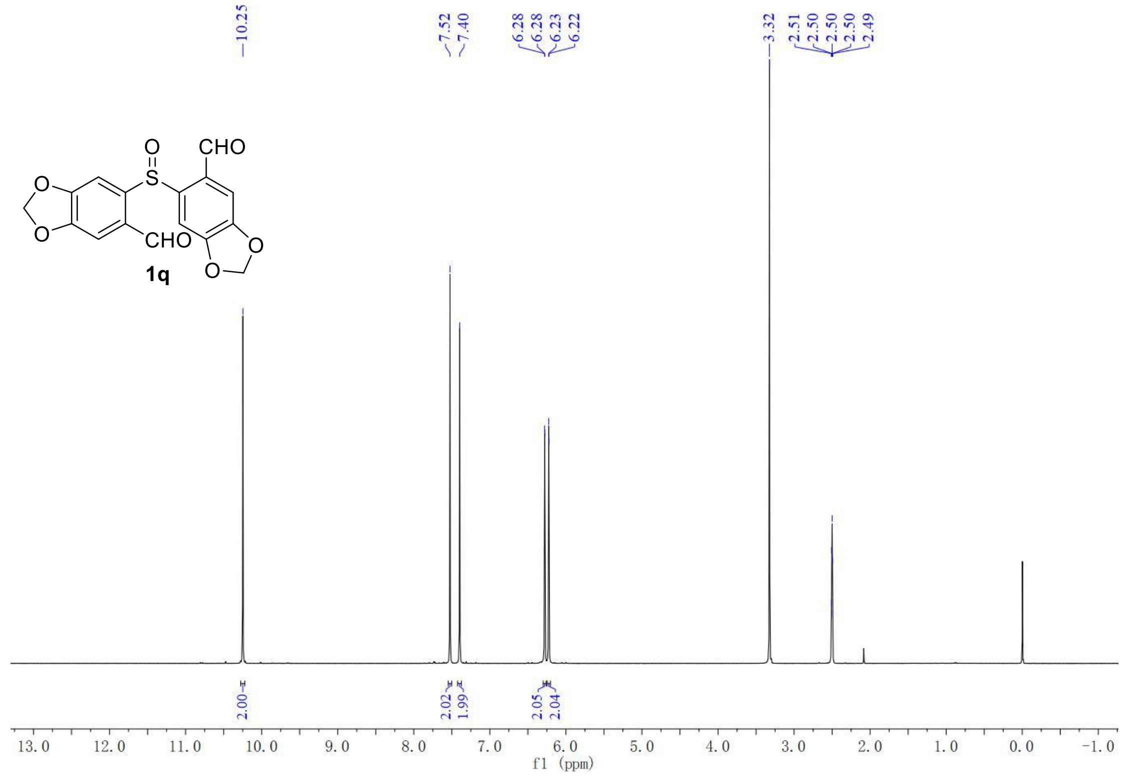

Supplementary Figure 39 ^{1}H NMR (400 MHz, DMSO-d_6) of **1q**

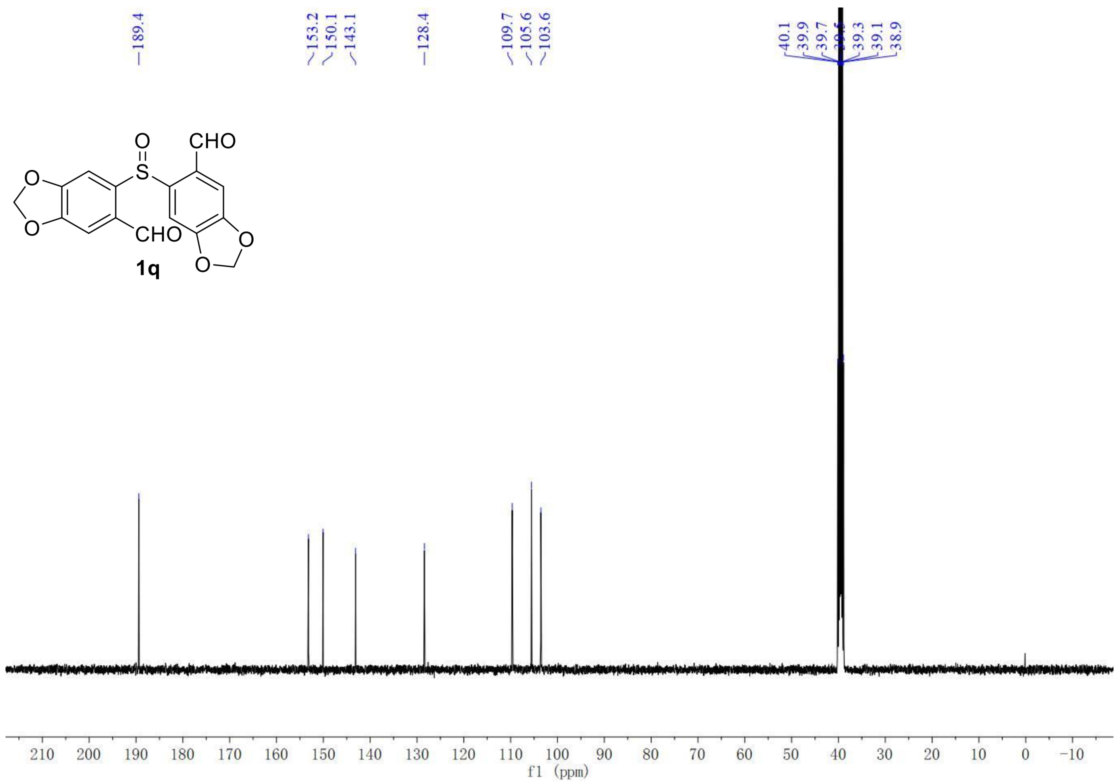

Supplementary Figure 40 ^{13}C NMR (101 MHz, DMSO-d_6) of **1q**

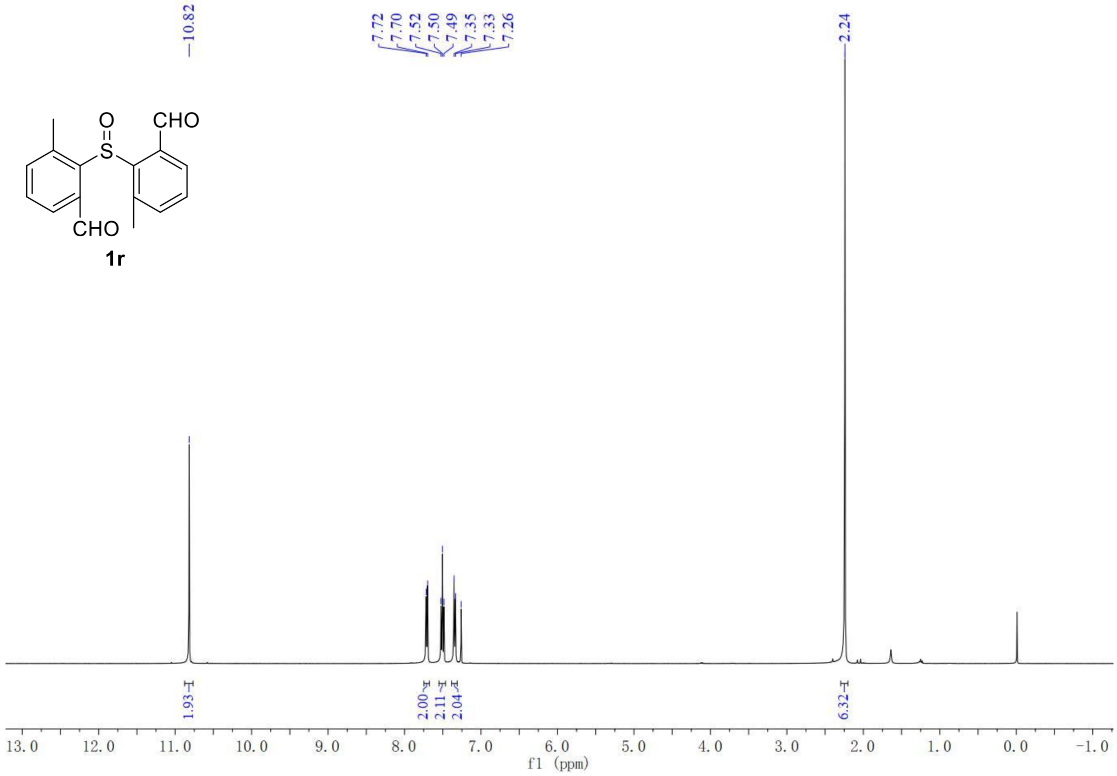

Supplementary Figure 41 ^{1}H NMR (400 MHz, $CDCl_3$) of **1r**

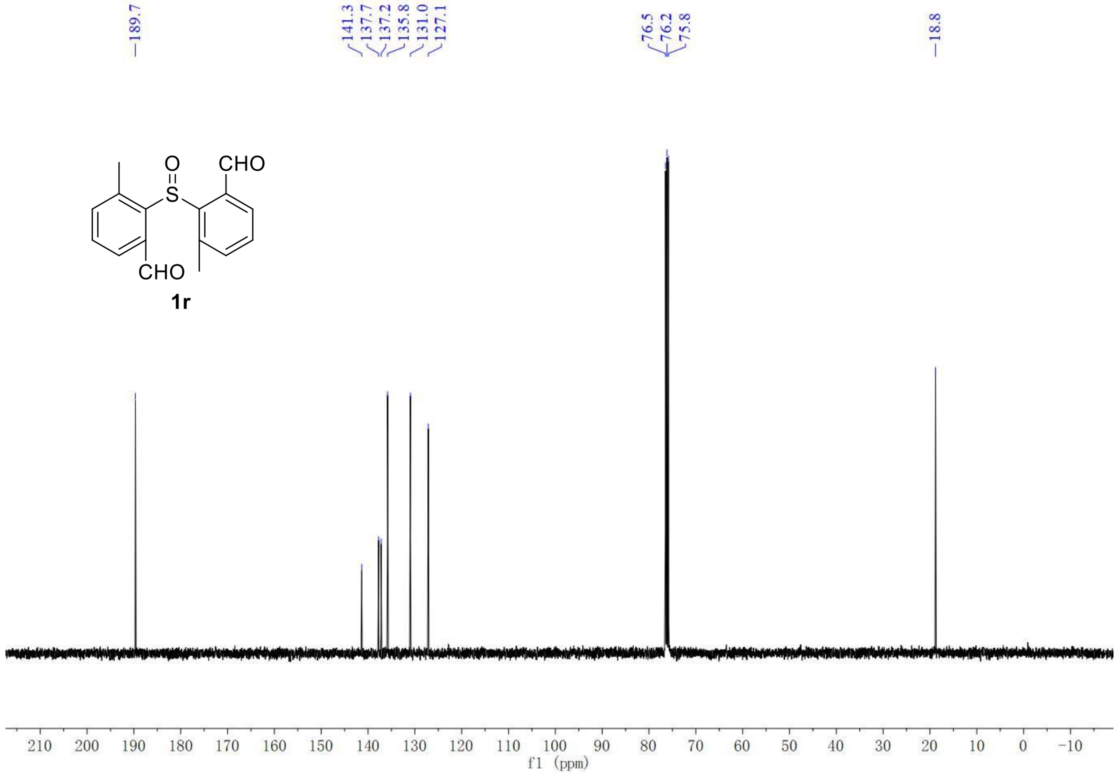

Supplementary Figure 42 ^{13}C NMR (101 MHz, $CDCl_3$) of **1r**

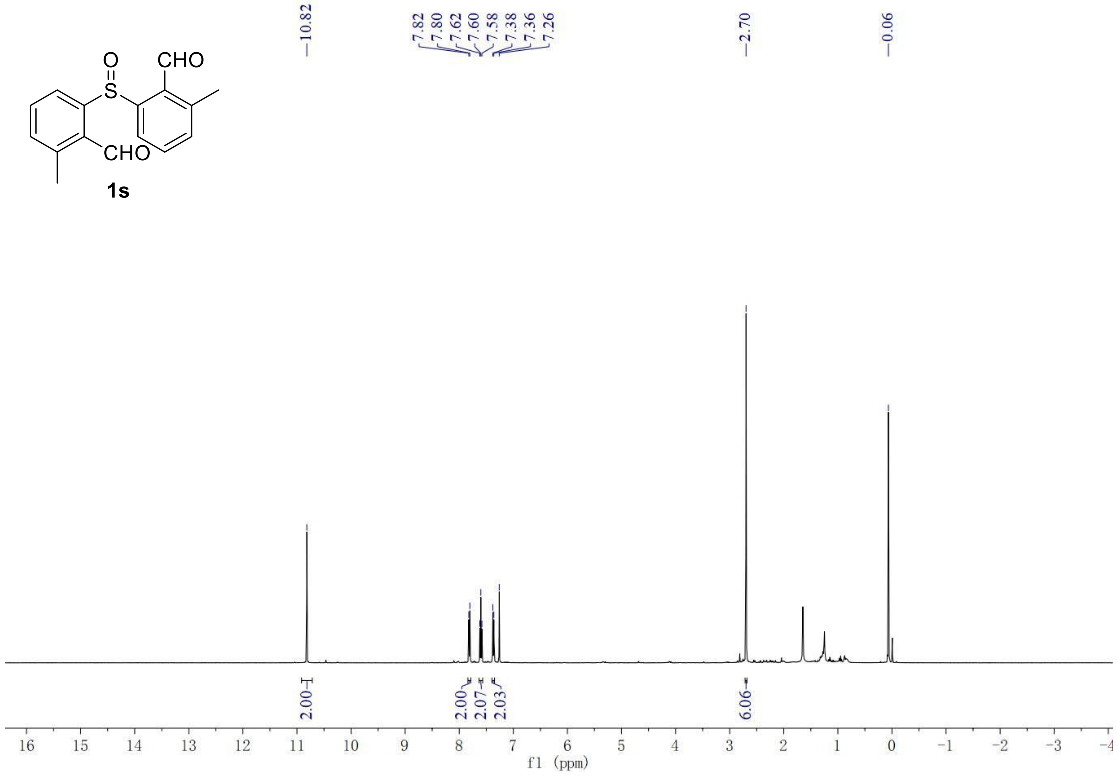

Supplementary Figure 43 ^{1}H NMR (400 MHz, $CDCl_3$) of **1s**

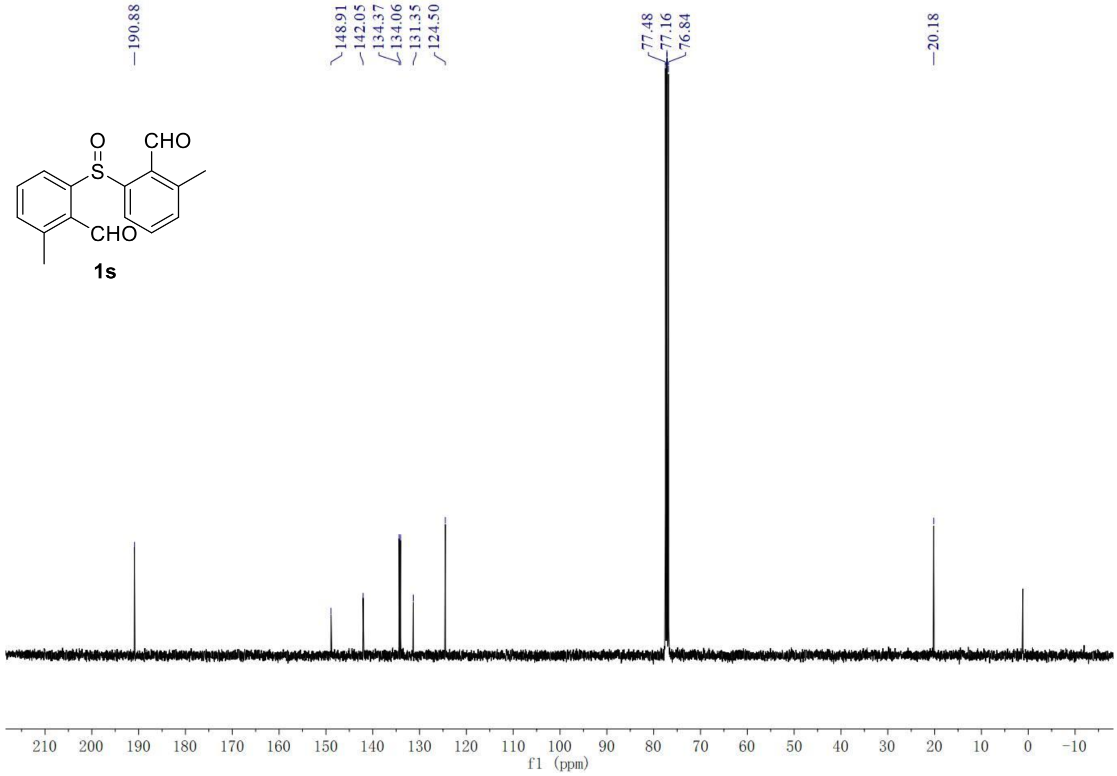

Supplementary Figure 44 ^{13}C NMR (101 MHz, $CDCl_3$) of **1s**

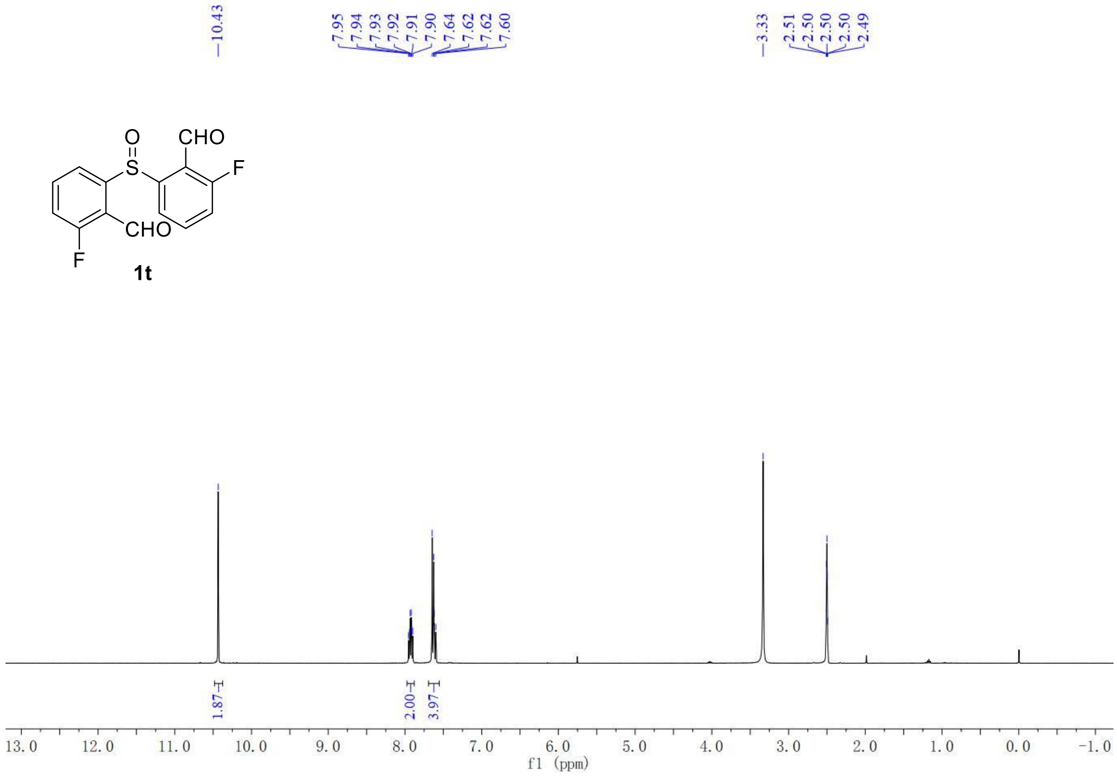

Supplementary Figure 45 ^{1}H NMR (400 MHz, $CDCl_3$) of **1t**

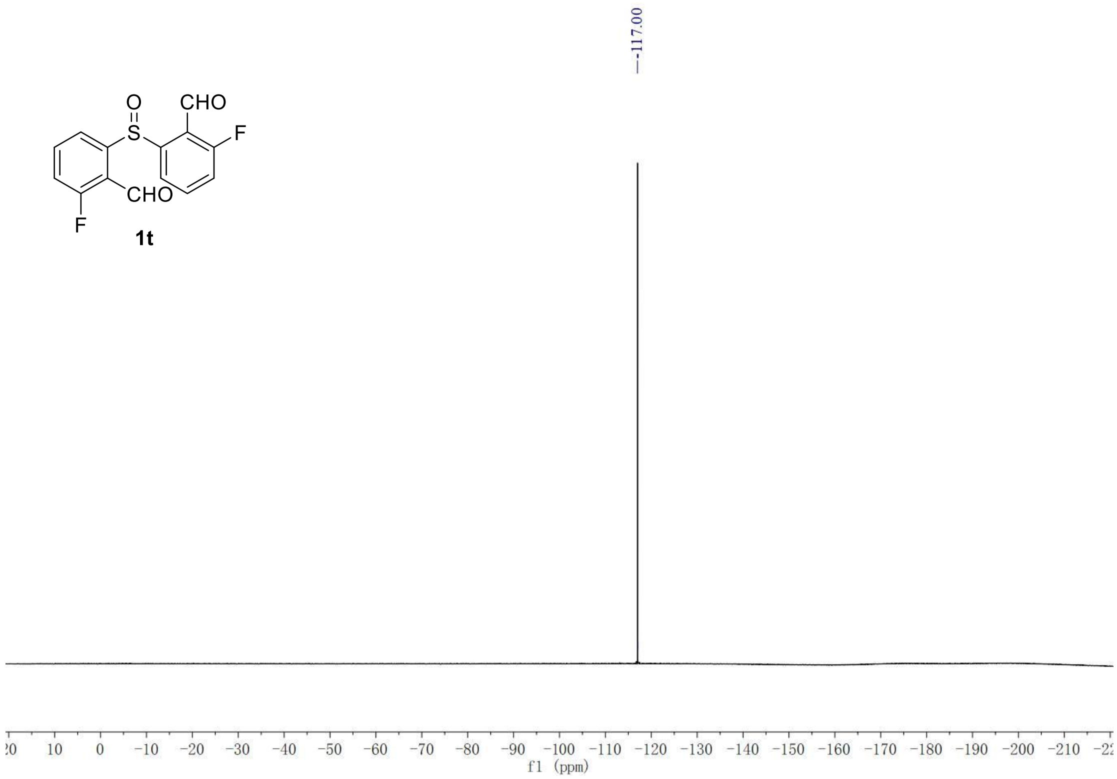

Supplementary Figure 46 ^{19}F NMR (376 MHz, $CDCl_3$) of **1t**

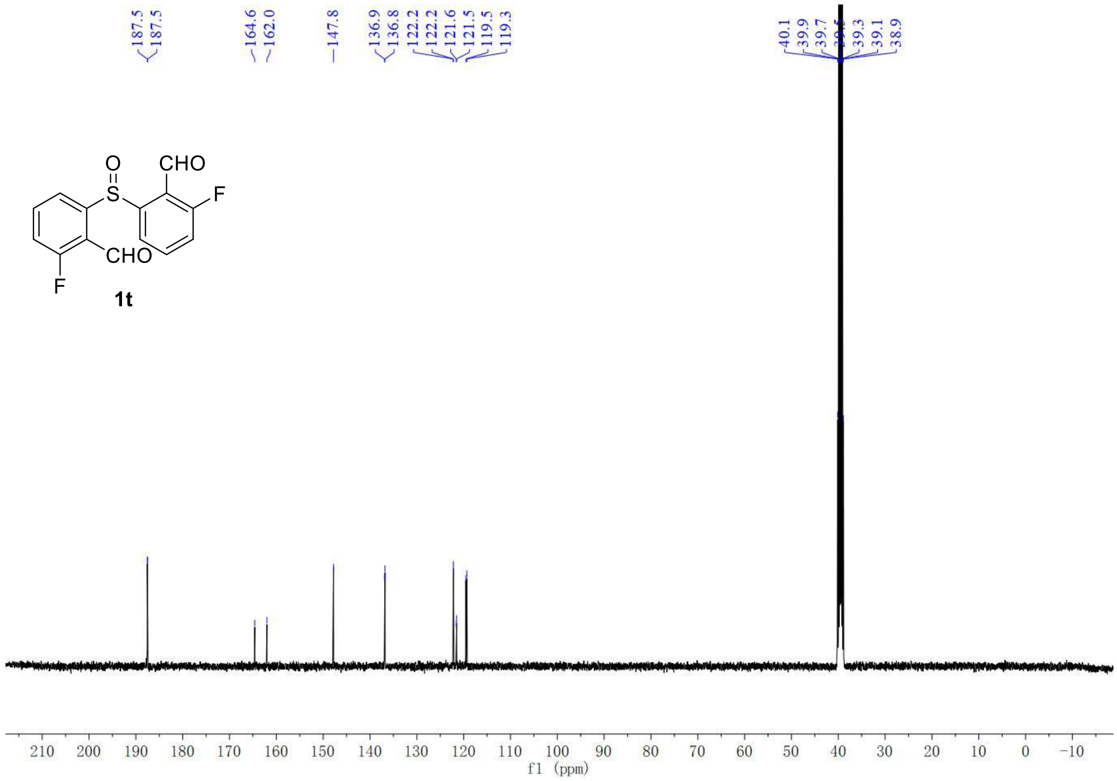

Supplementary Figure 47 ^{13}C NMR (101 MHz, $CDCl_3$) of **1t**

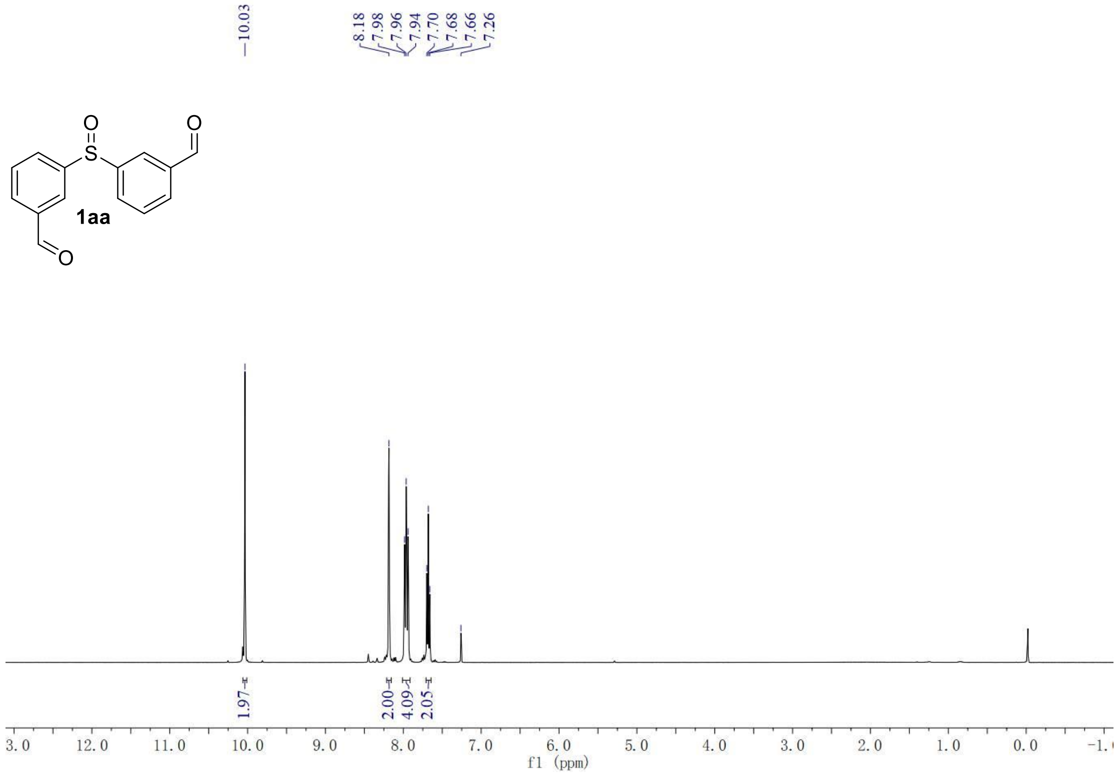

Supplementary Figure 48 ^{1}H NMR (400 MHz, $CDCl_3$) of **1aa**

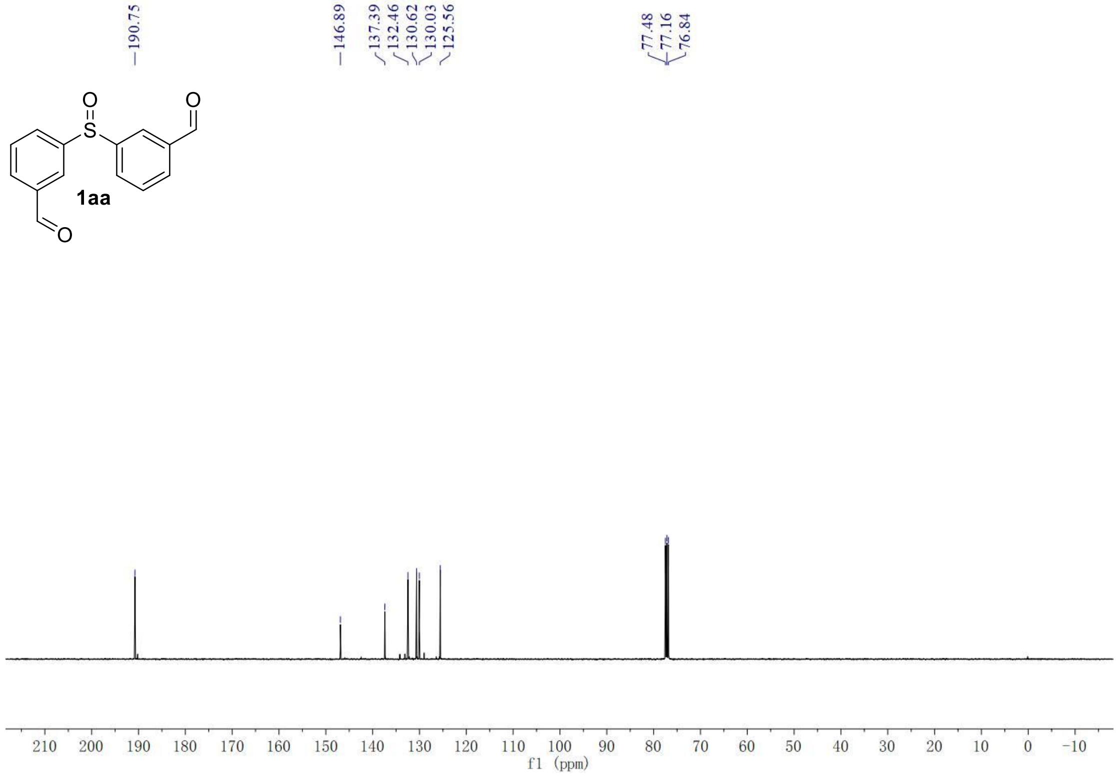

Supplementary Figure 49 ^{13}C NMR (101 MHz, $CDCl_3$) of **1aa**

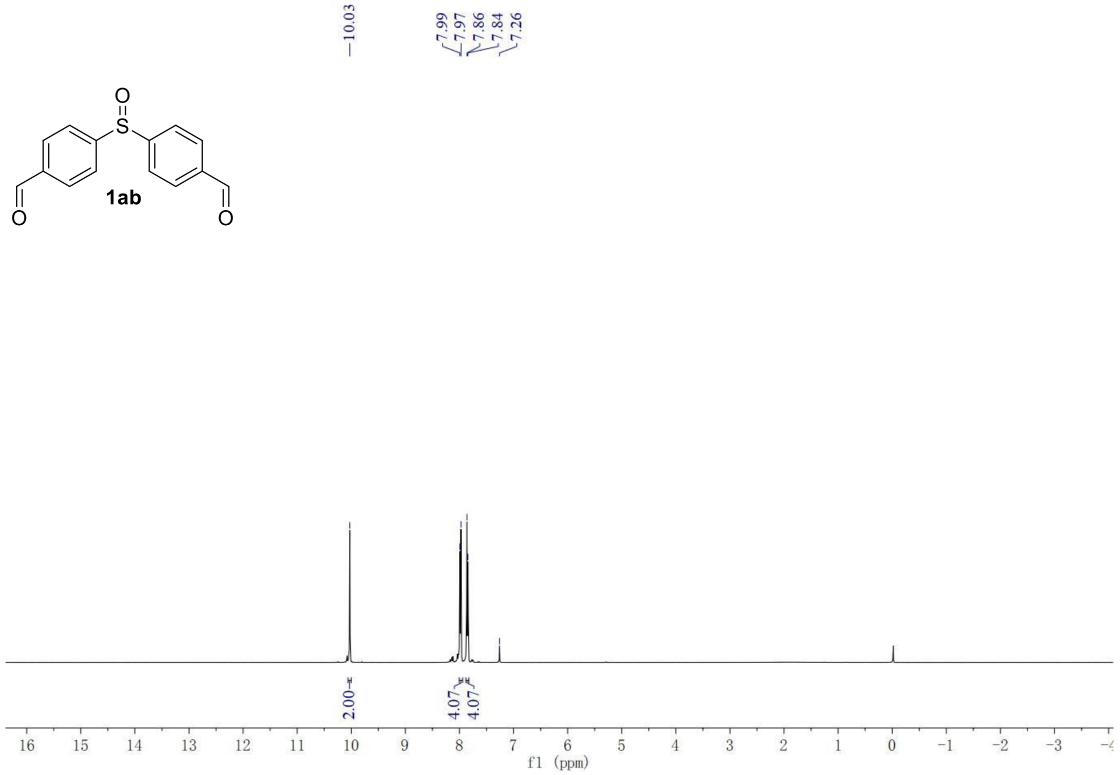

Supplementary Figure 50 ^{1}H NMR (400 MHz, $CDCl_3$) of **1ab**

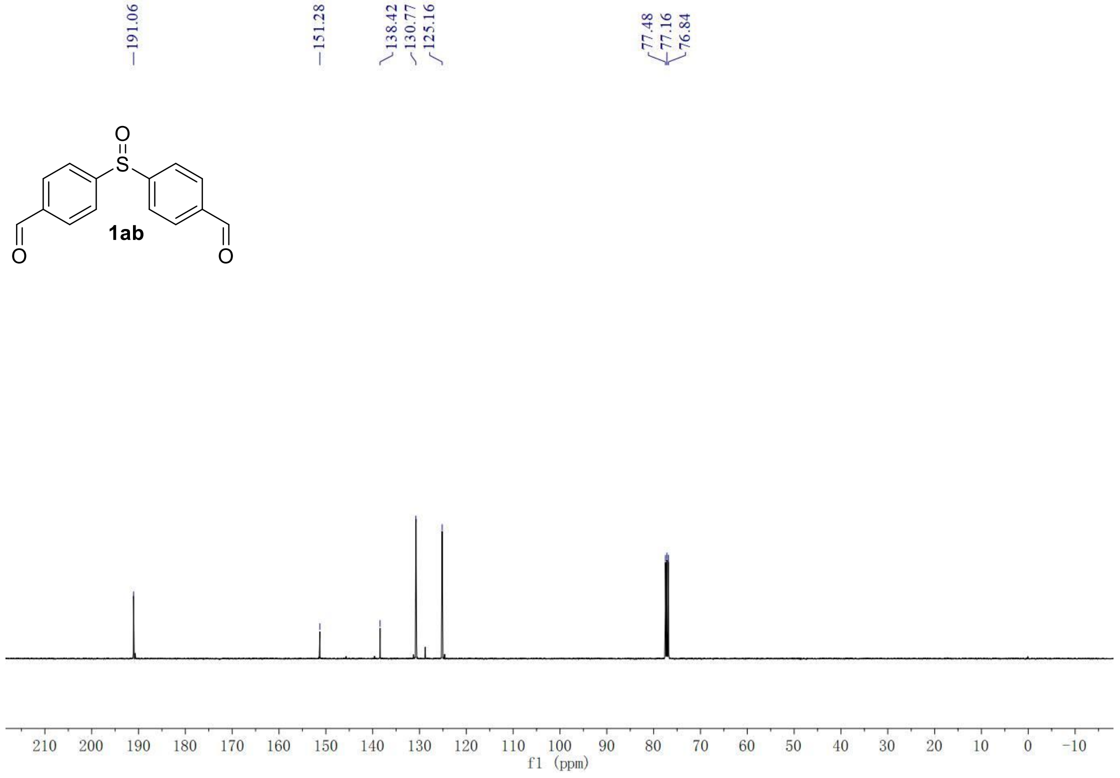

Supplementary Figure 51 ^{13}C NMR (101 MHz, $CDCl_3$) of **1ab**

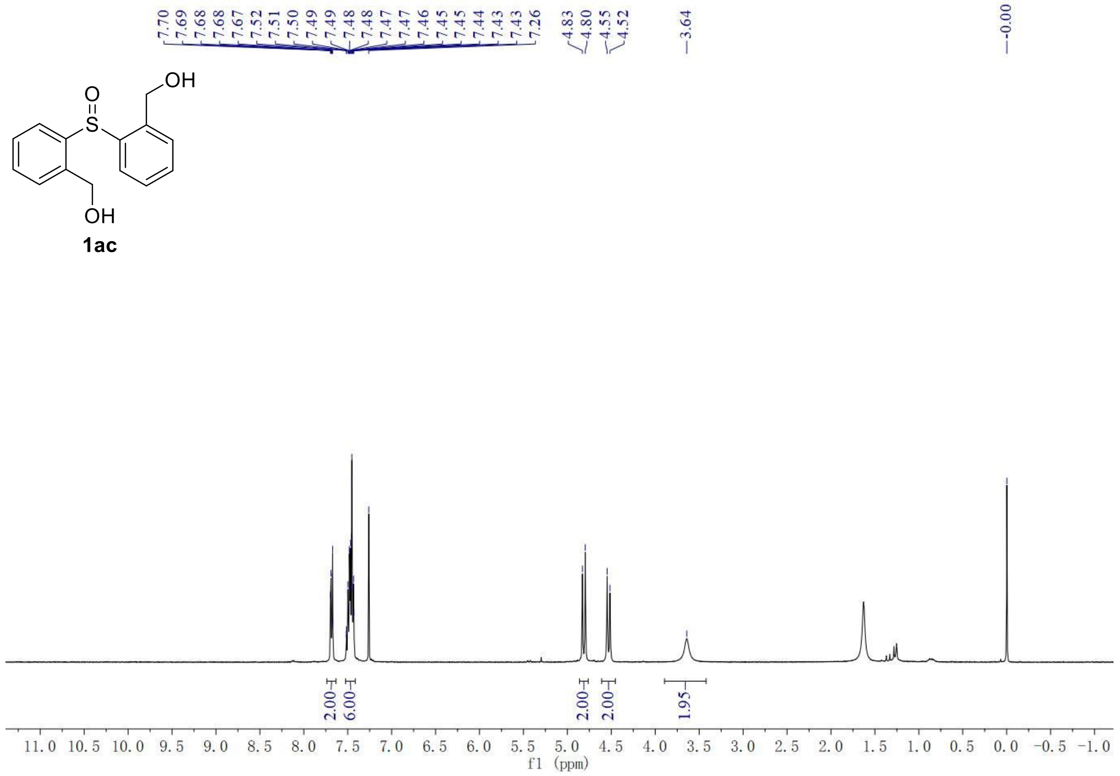

Supplementary Figure 52 ^{1}H NMR (400 MHz, $CDCl_3$) of **1ac**

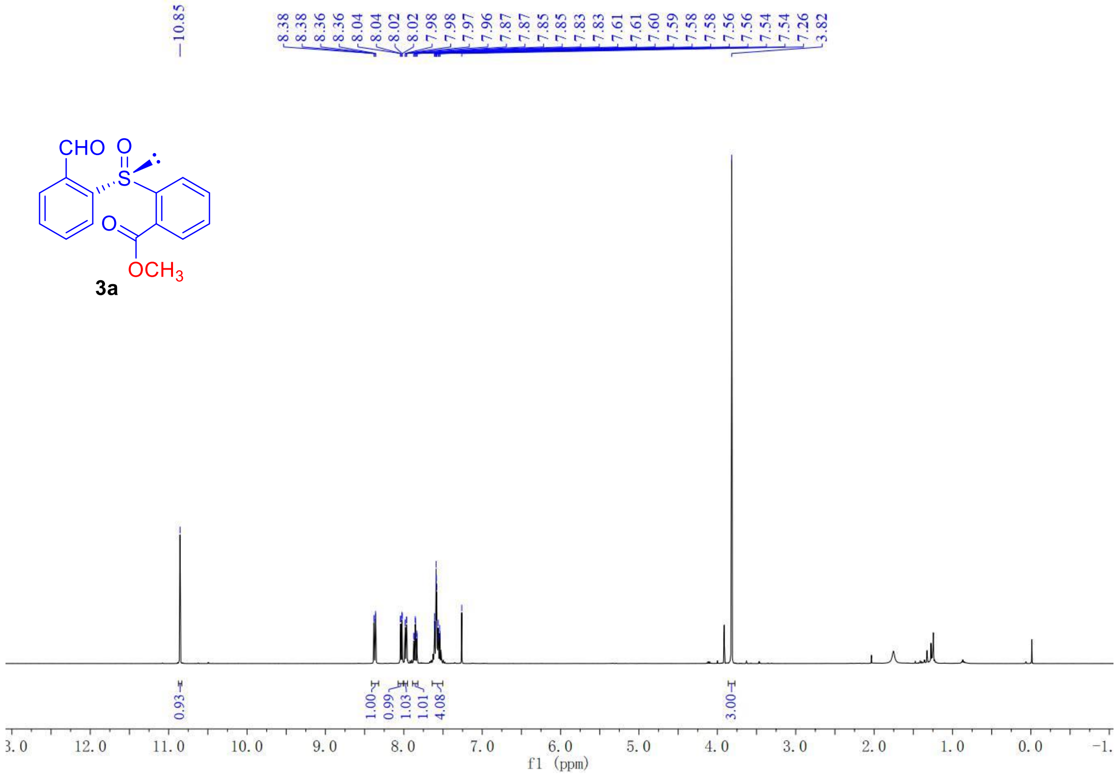

Supplementary Figure 53 ^{1}H NMR (400 MHz, $CDCl_3$) of **3a**

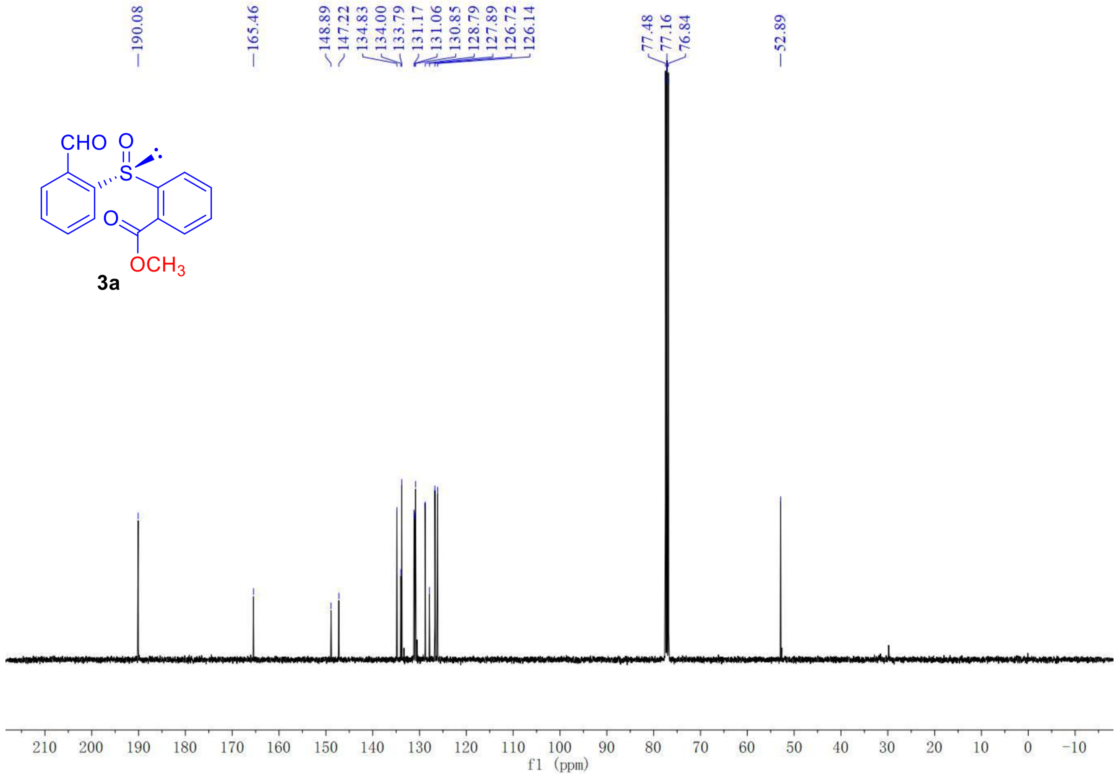

Supplementary Figure 54 ^{13}C NMR (101 MHz, $CDCl_3$) of **3a**

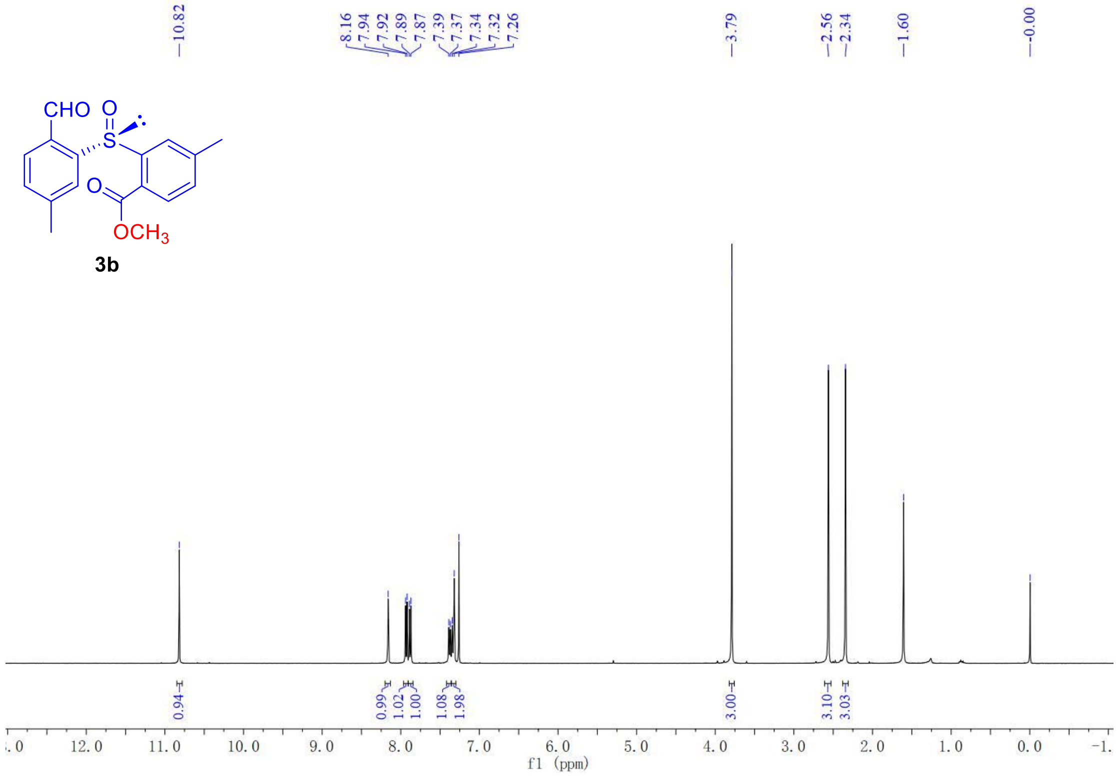

Supplementary Figure 55 ^{1}H NMR (400 MHz, $CDCl_3$) of **3b**

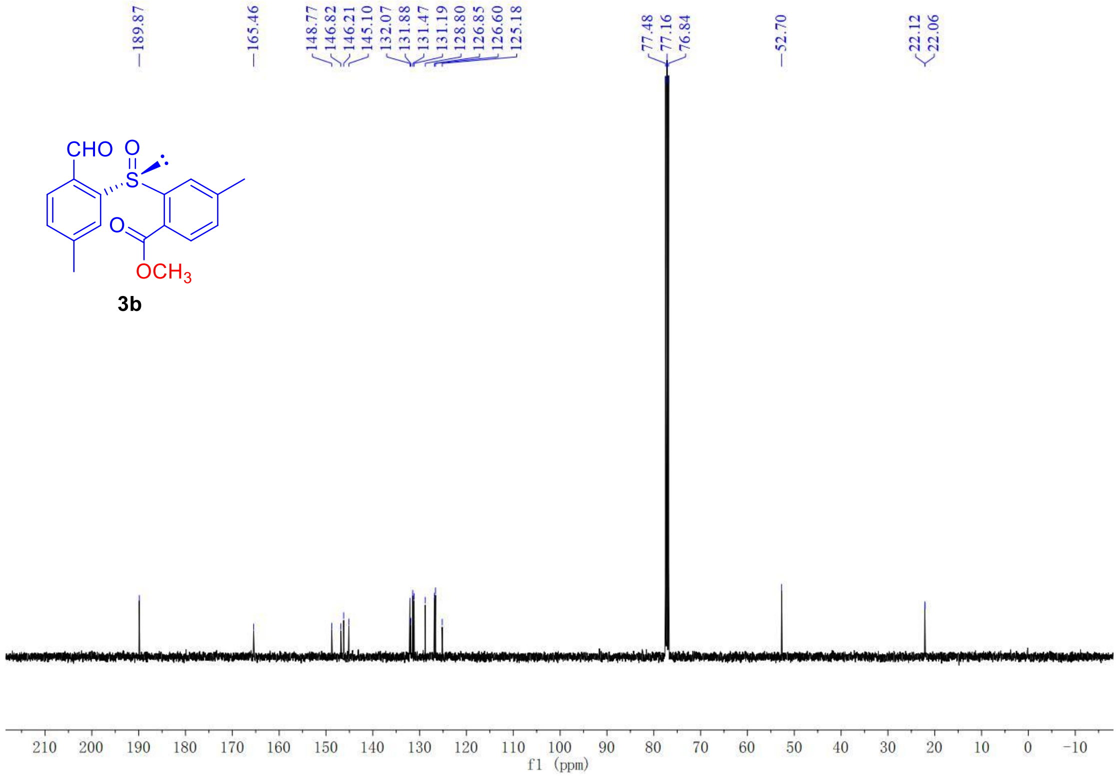

Supplementary Figure 56 ^{13}C NMR (101 MHz, $CDCl_3$) of **3b**

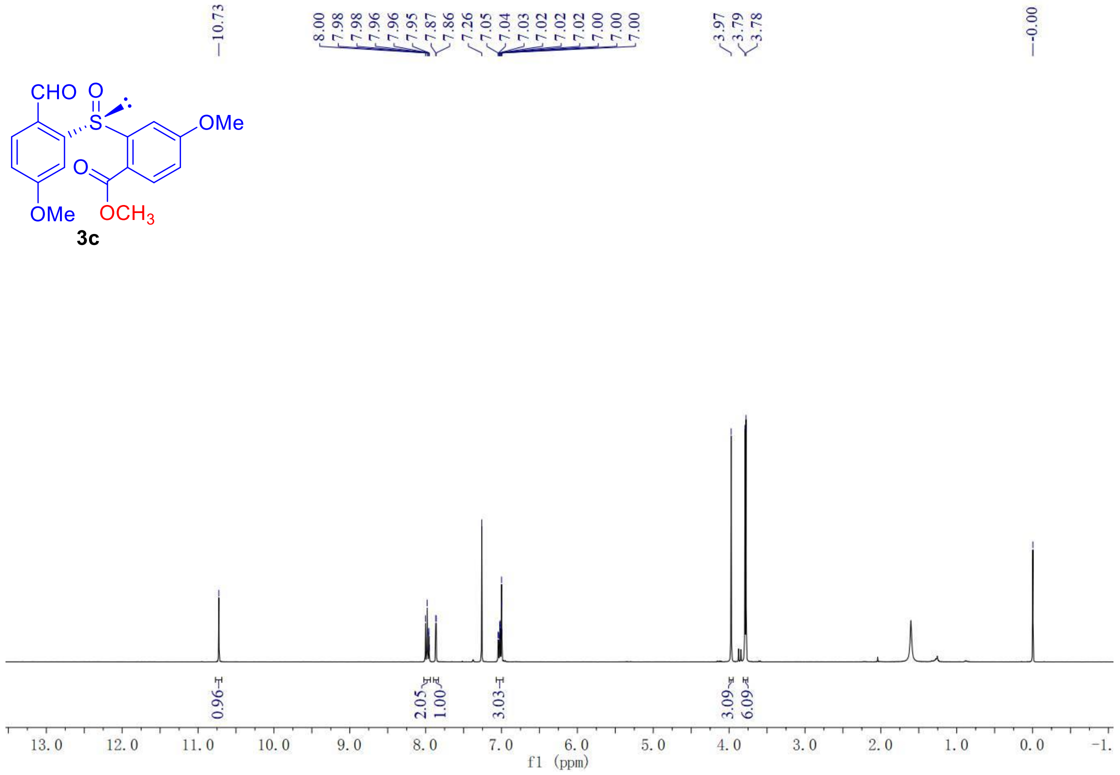

Supplementary Figure 57 ^{1}H NMR (400 MHz, $CDCl_3$) of **3c**

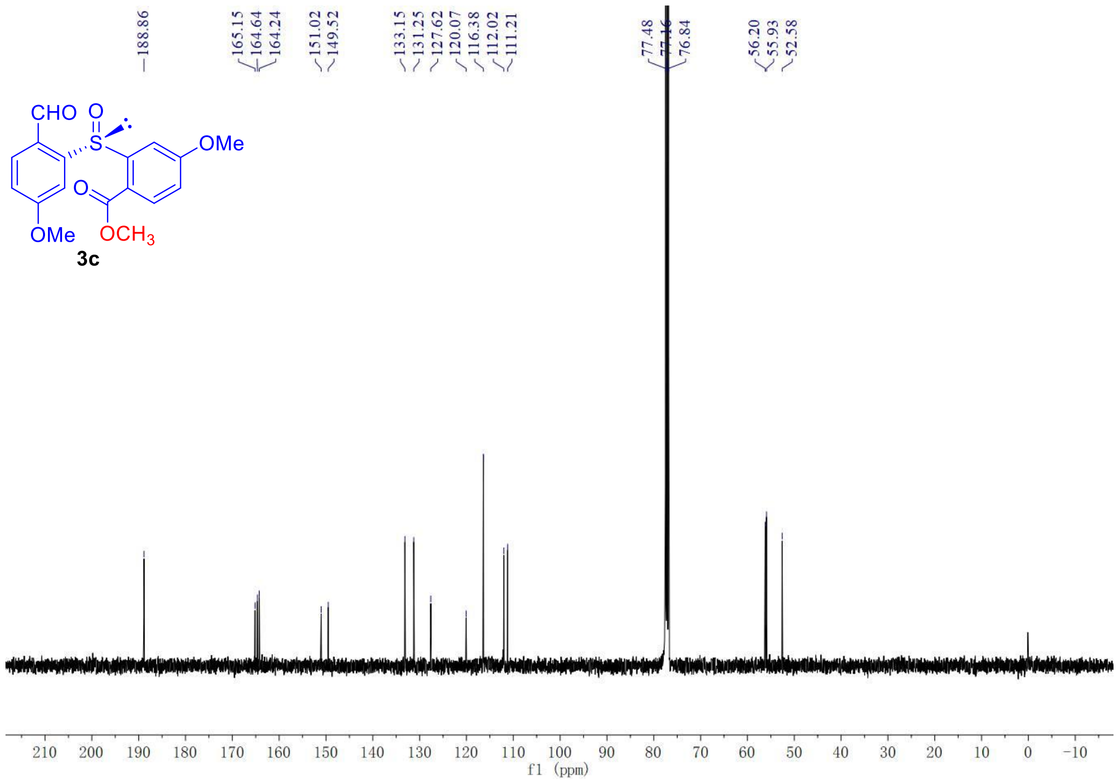

Supplementary Figure 58 ^{13}C NMR (101 MHz, $CDCl_3$) of **3c**

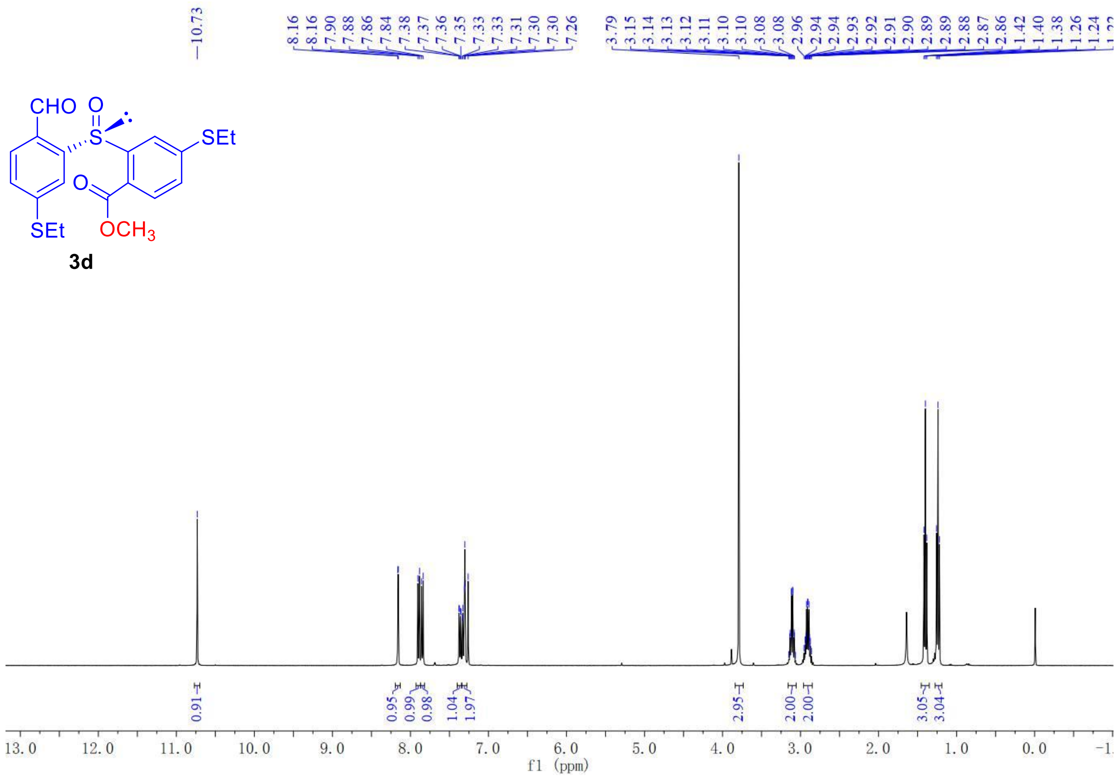

Supplementary Figure 59 ^{1}H NMR (400 MHz, $CDCl_3$) of **3d**

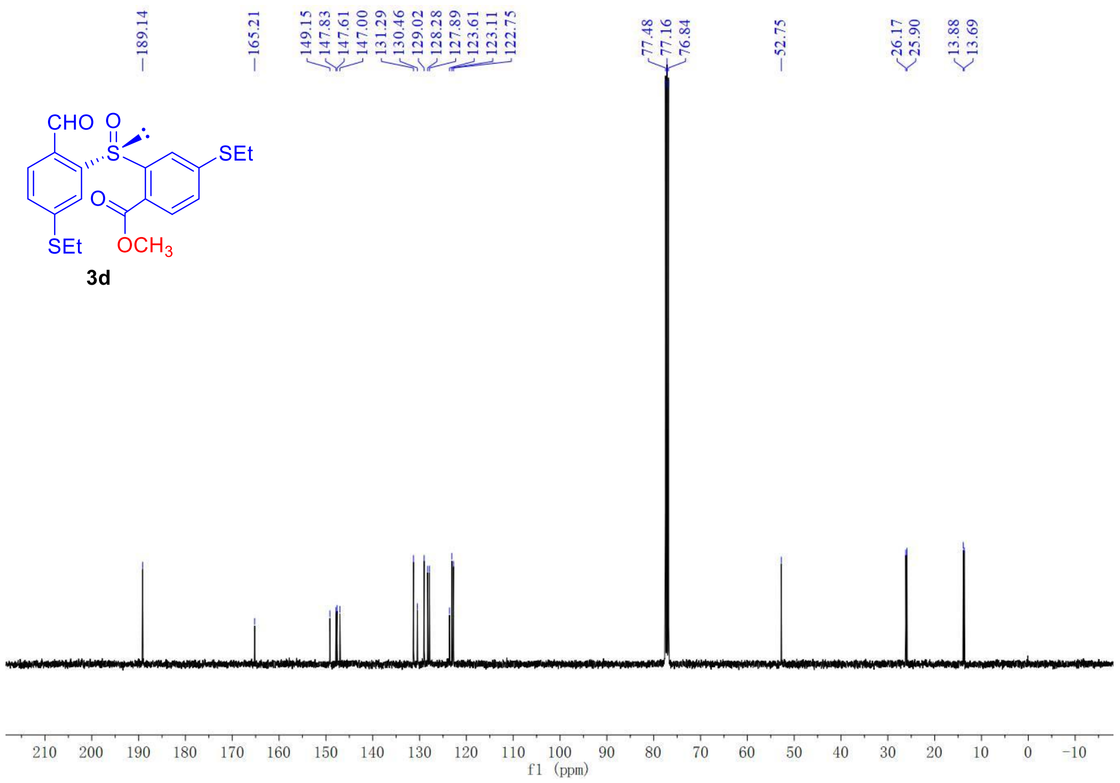

Supplementary Figure 60 ^{13}C NMR (101 MHz, $CDCl_3$) of **3d**

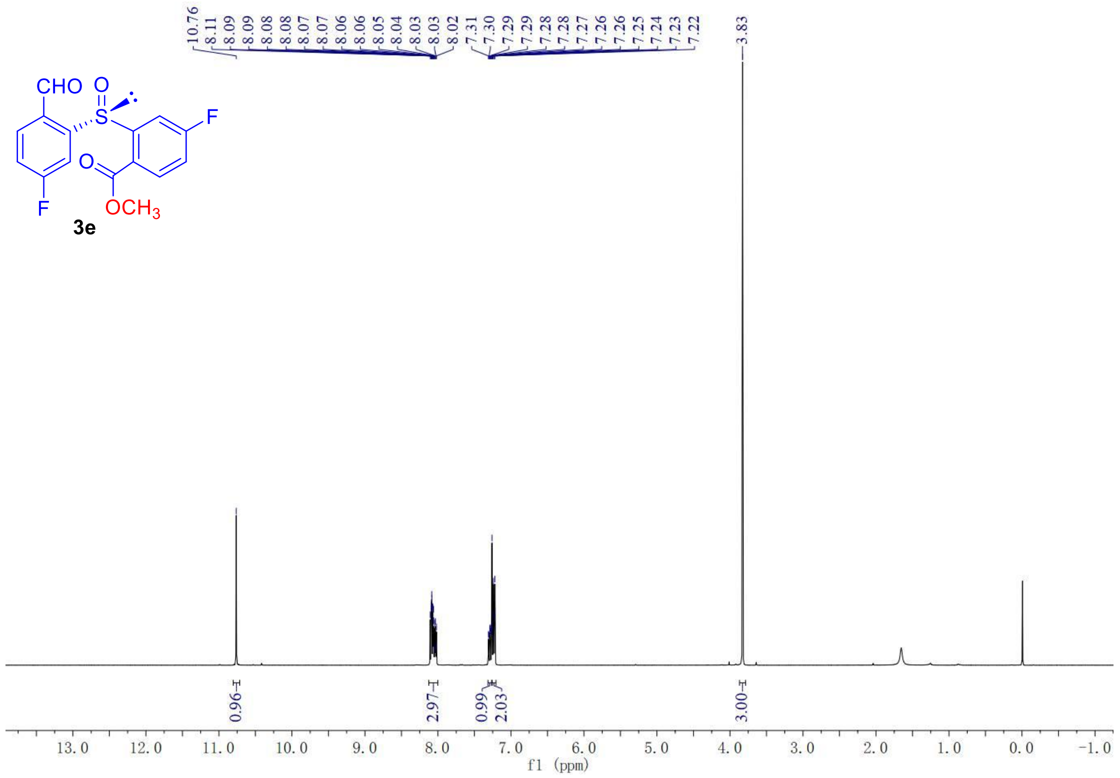

Supplementary Figure 61 ^{1}H NMR (400 MHz, $CDCl_3$) of **3e**

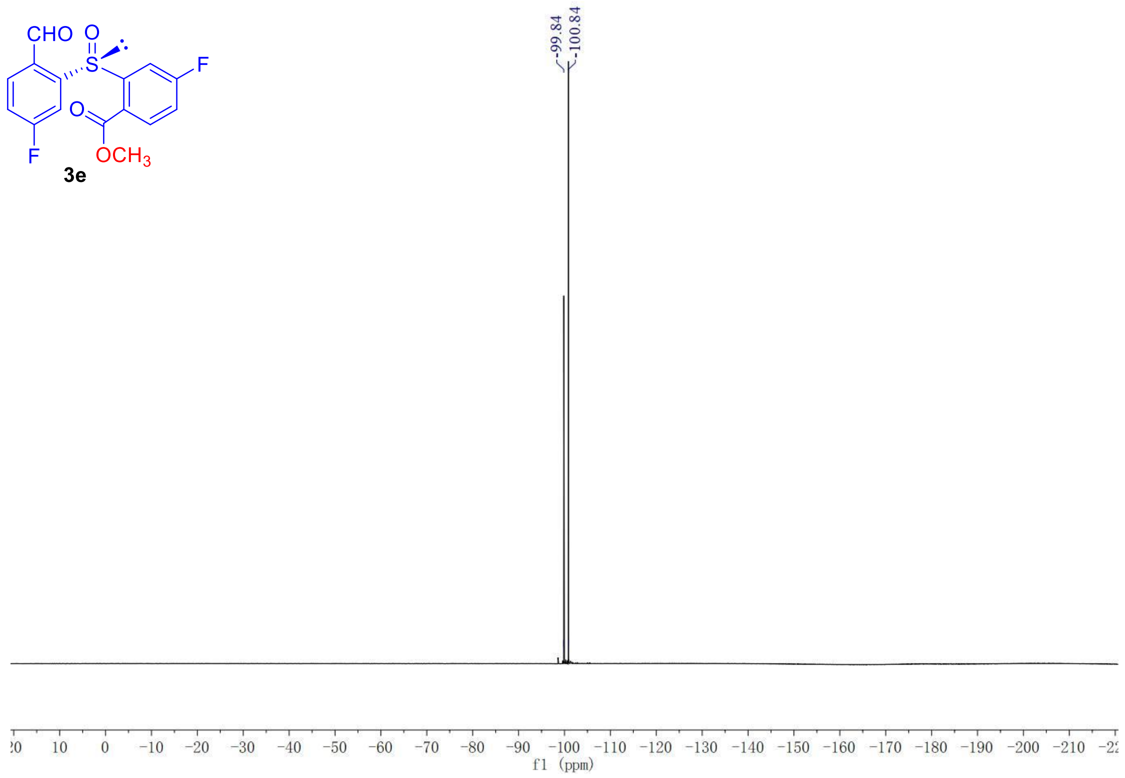

Supplementary Figure 62 ^{19}F NMR (376 MHz, $CDCl_3$) of **3e**

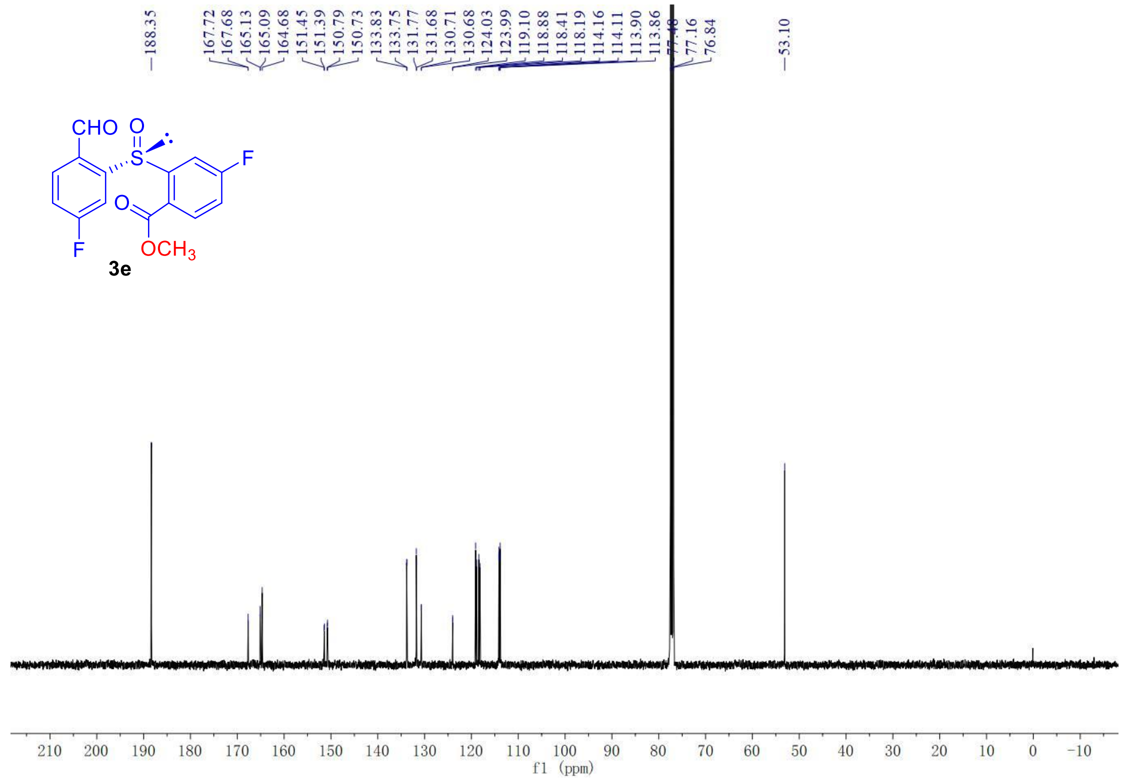

Supplementary Figure 63 ^{13}C NMR (101 MHz, $CDCl_3$) of **3e**

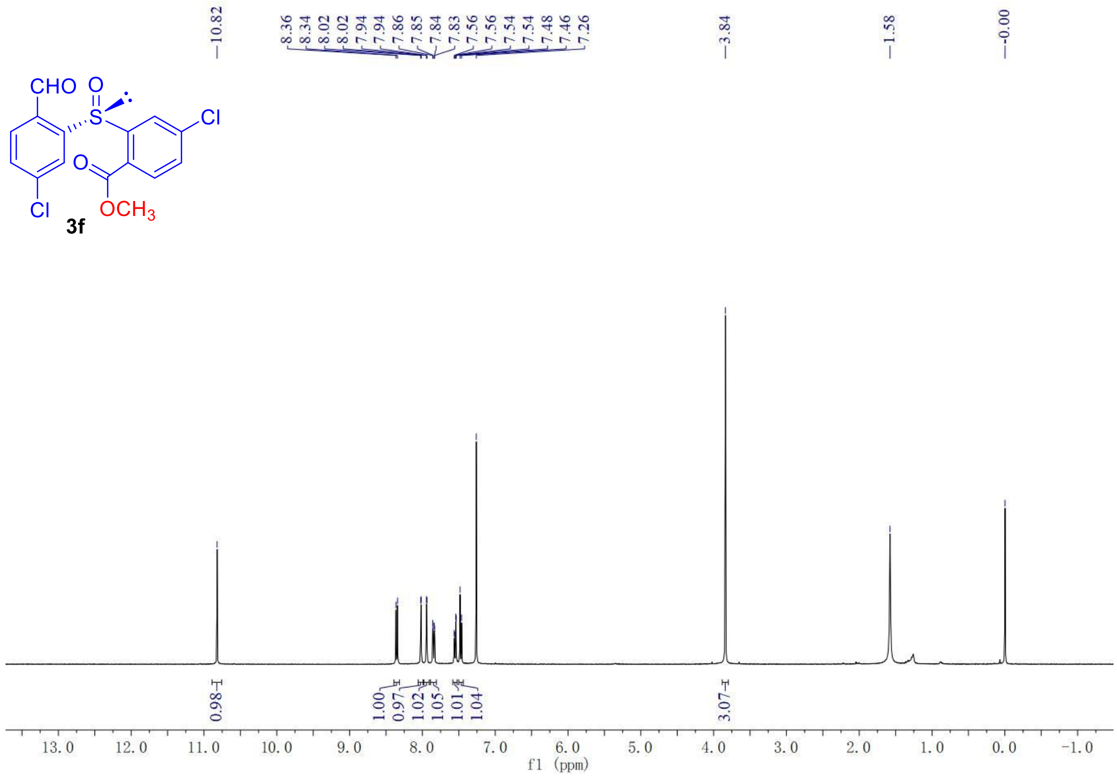

Supplementary Figure 64 ^{1}H NMR (400 MHz, $CDCl_3$) of **3f**

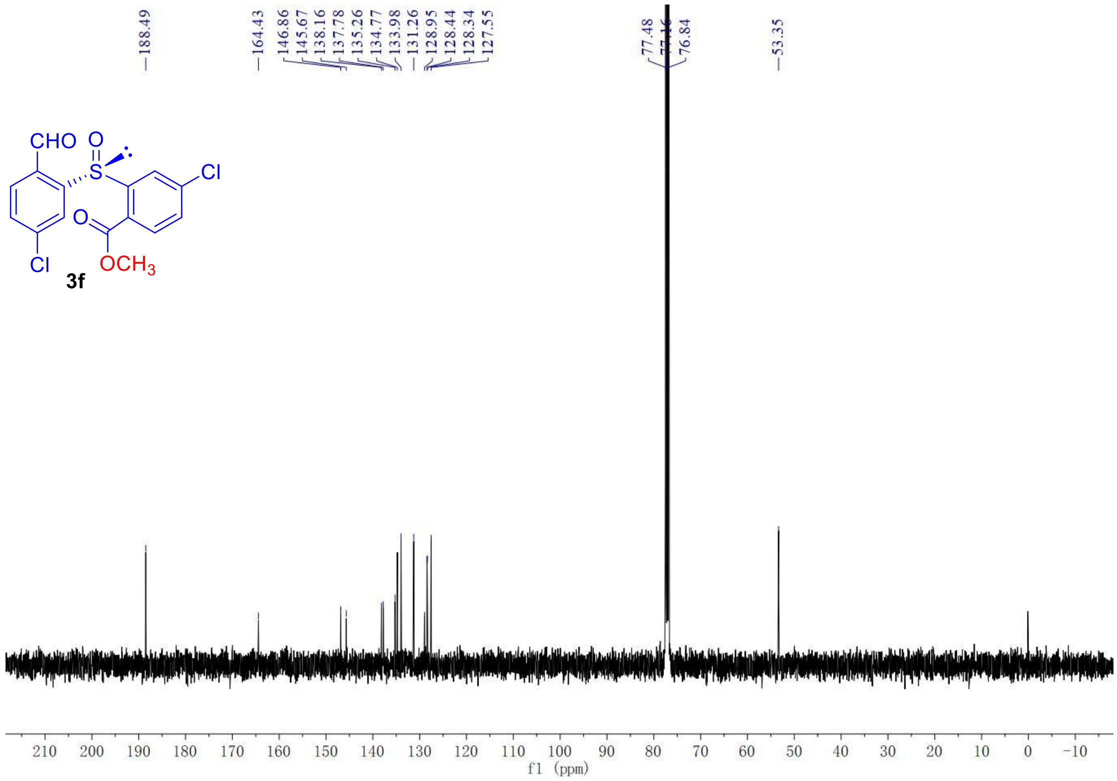

Supplementary Figure 65 ^{13}C NMR (101 MHz, $CDCl_3$) of **3f**

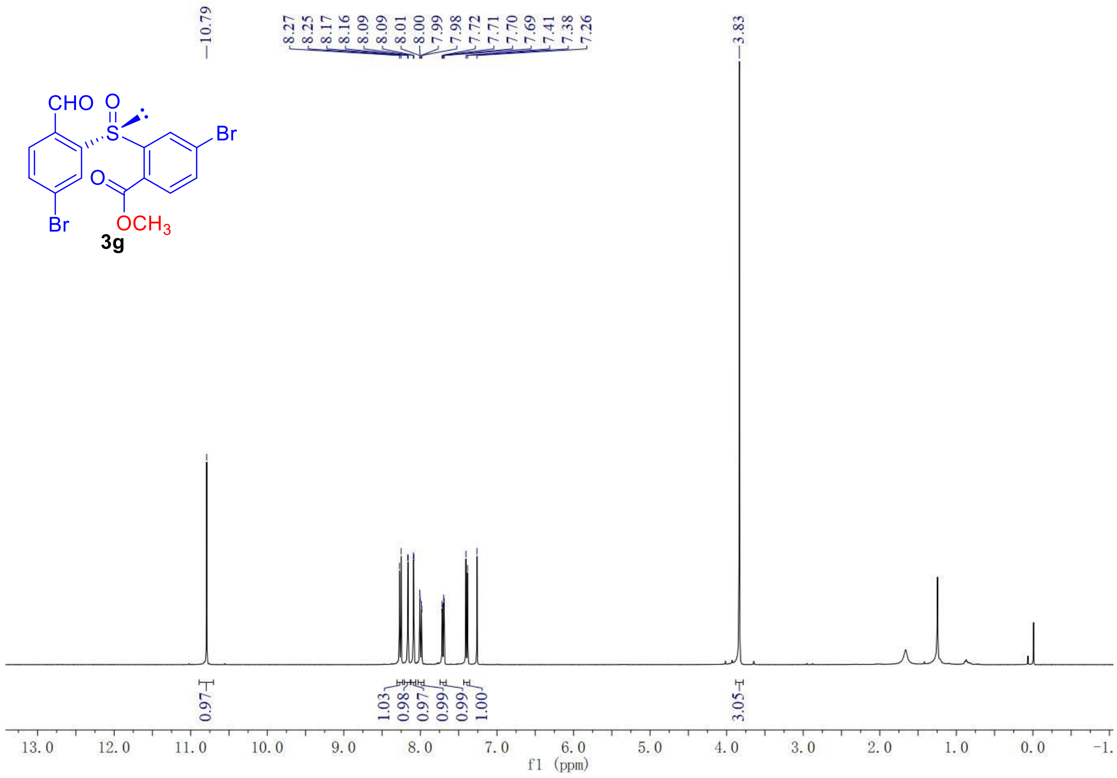

Supplementary Figure 66 ^{1}H NMR (400 MHz, $CDCl_3$) of **3g**

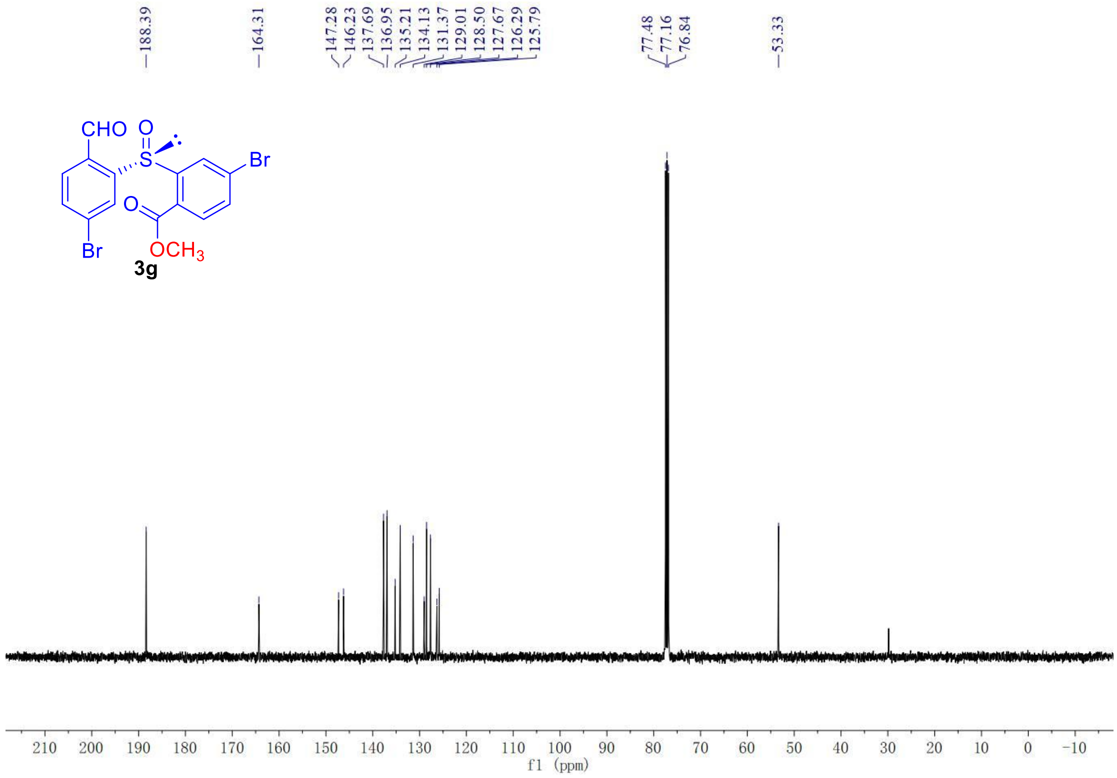

Supplementary Figure 67 ^{13}C NMR (101 MHz, $CDCl_3$) of **3g**

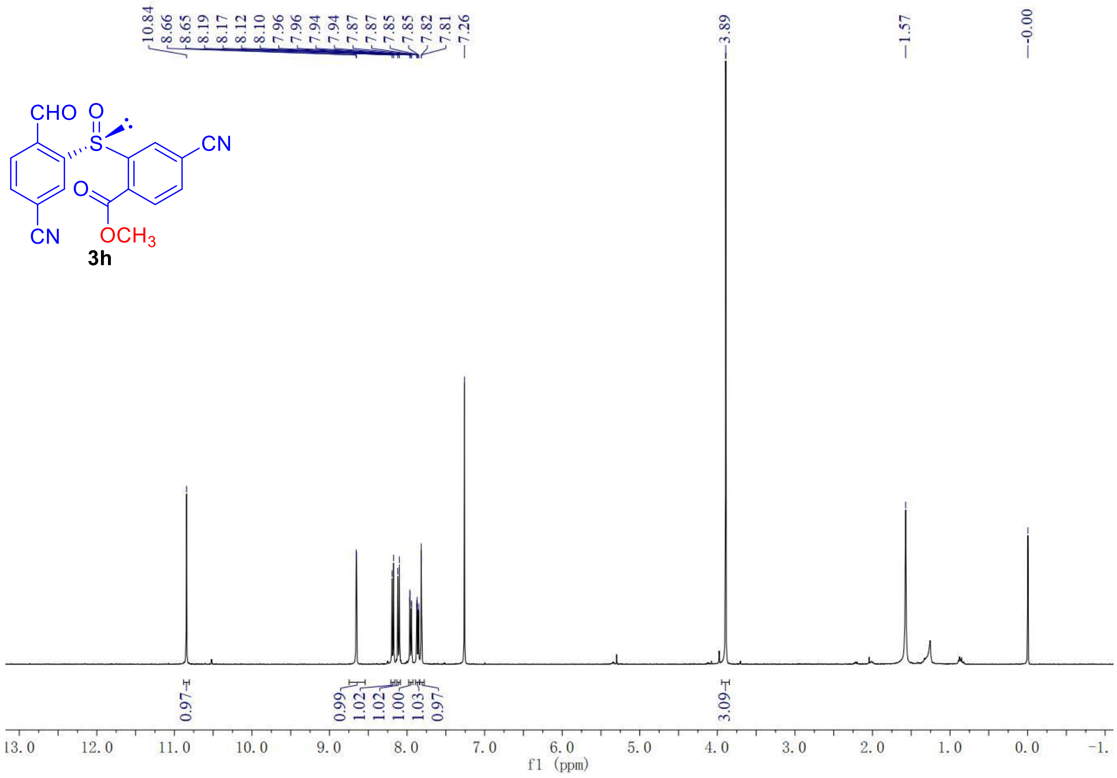

Supplementary Figure 68 ^{1}H NMR (400 MHz, $CDCl_3$) of **3h**

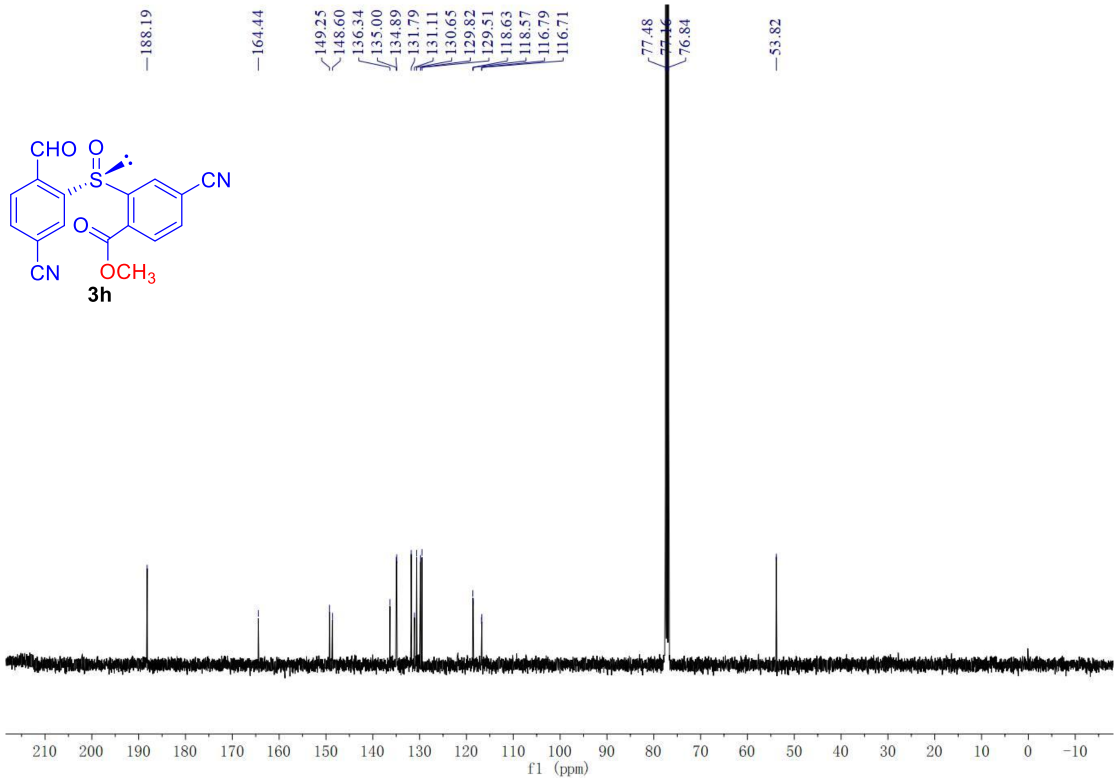

Supplementary Figure 69 ^{13}C NMR (101 MHz, $CDCl_3$) of **3h**

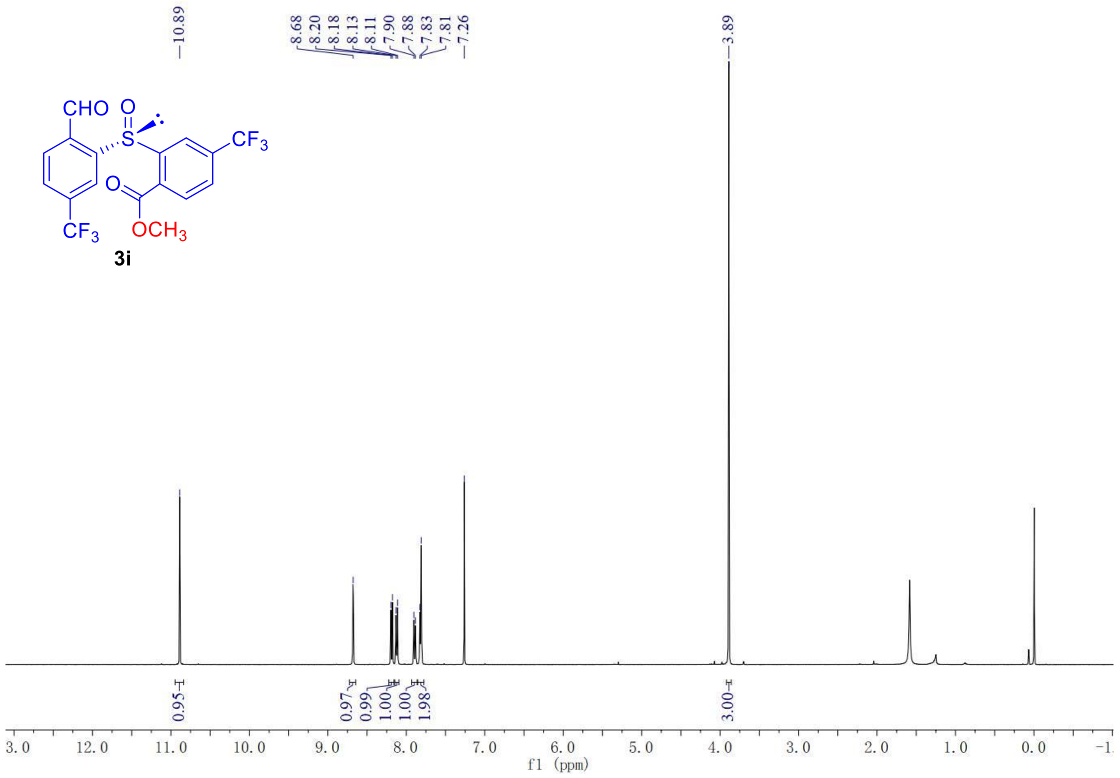

Supplementary Figure 70 ^{1}H NMR (400 MHz, $CDCl_3$) of **3i**

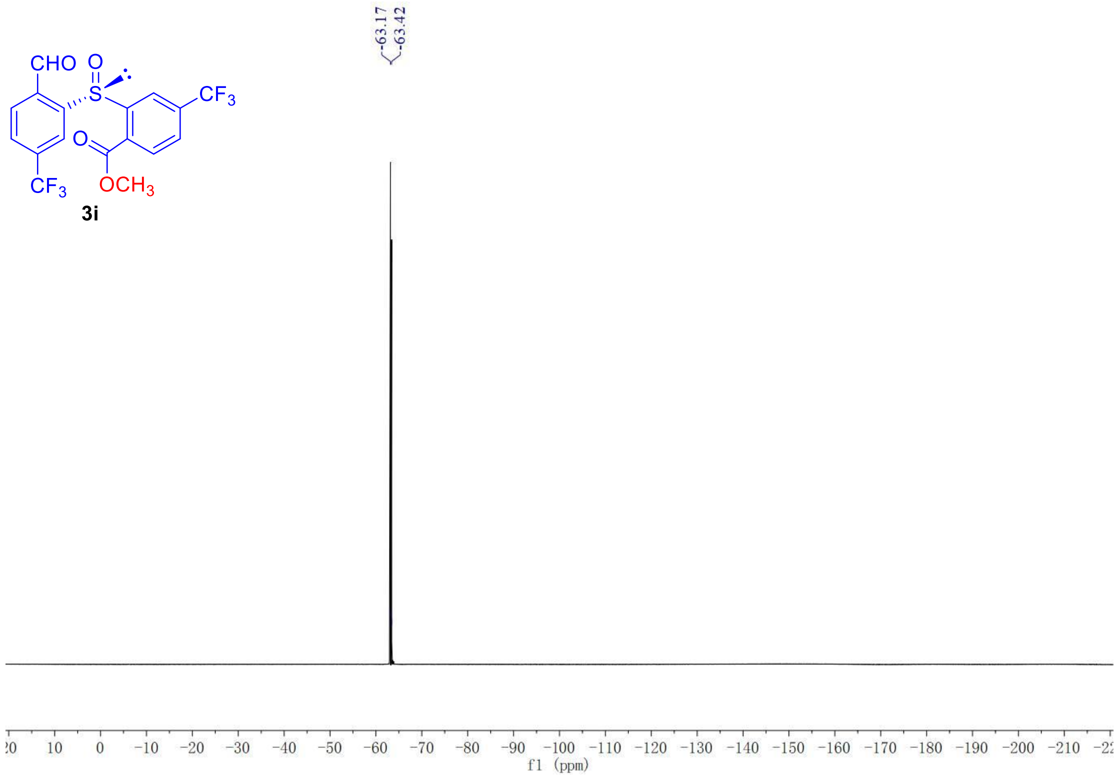

Supplementary Figure 71 ^{19}F NMR (376 MHz, $CDCl_3$) of **3i**

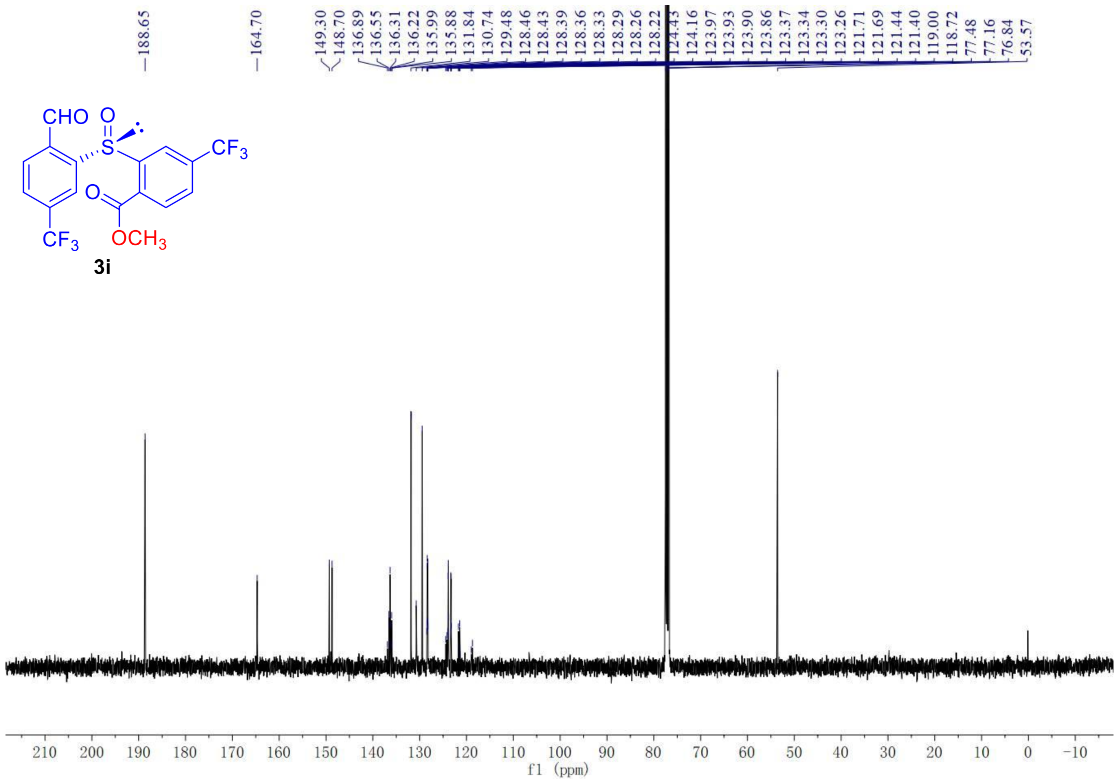

Supplementary Figure 72 ^{13}C NMR (101 MHz, $CDCl_3$) of **3i**

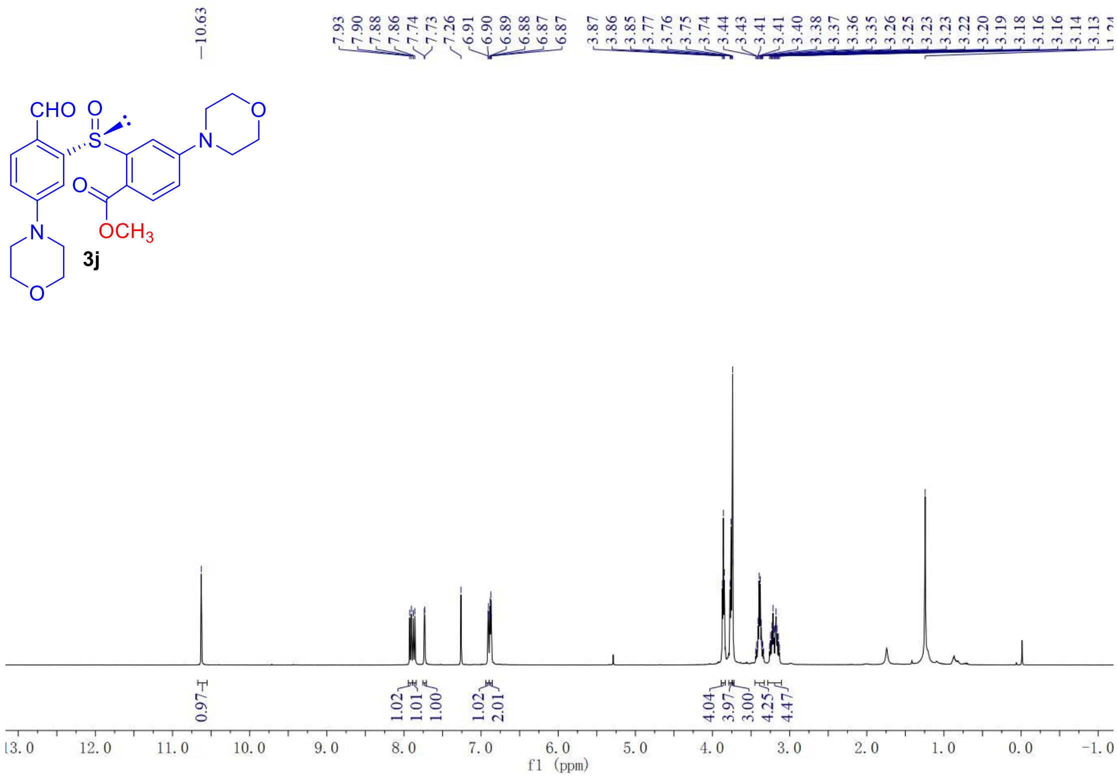

Supplementary Figure 73 ^{1}H NMR (400 MHz, $CDCl_3$) of **3j**

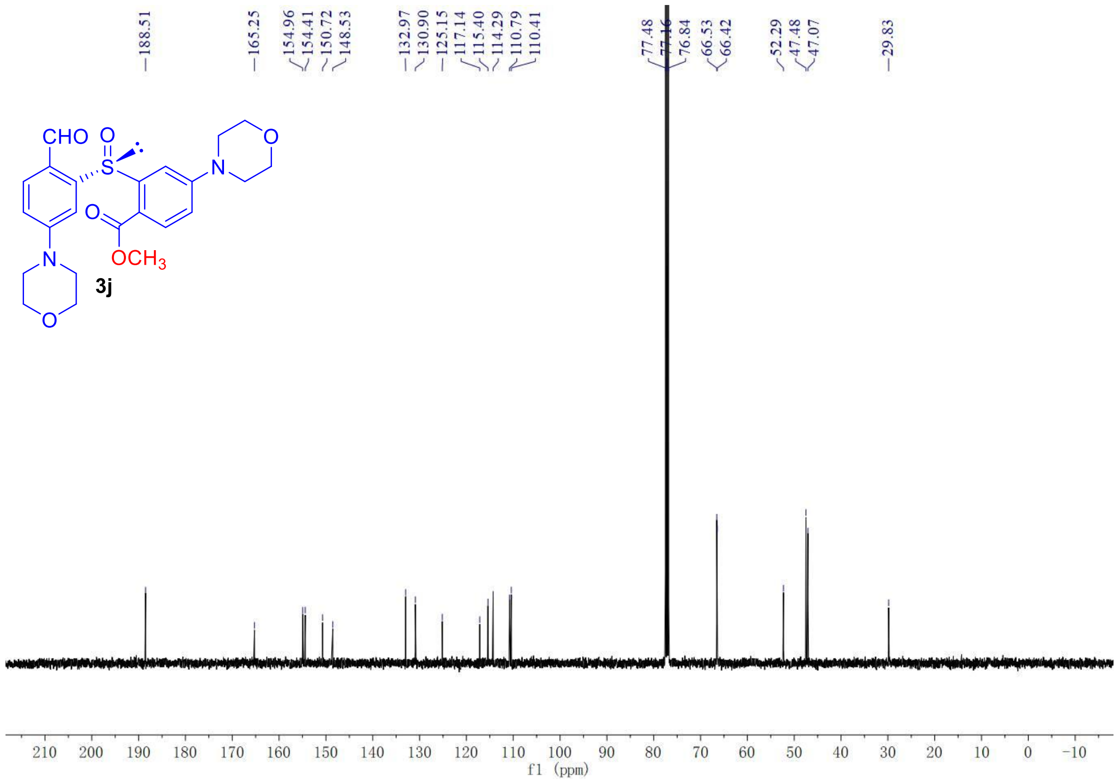

Supplementary Figure 74 ^{13}C NMR (101 MHz, $CDCl_3$) of **3j**

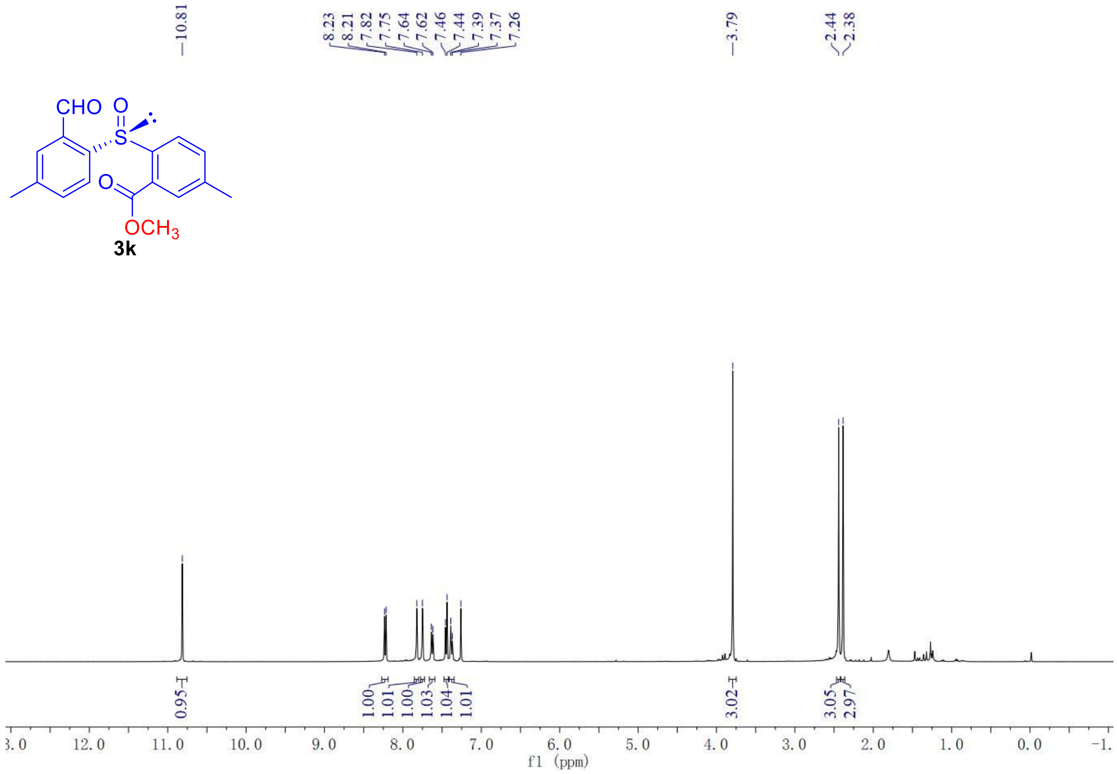

Supplementary Figure 75 ^{1}H NMR (400 MHz, $CDCl_3$) of **3k**

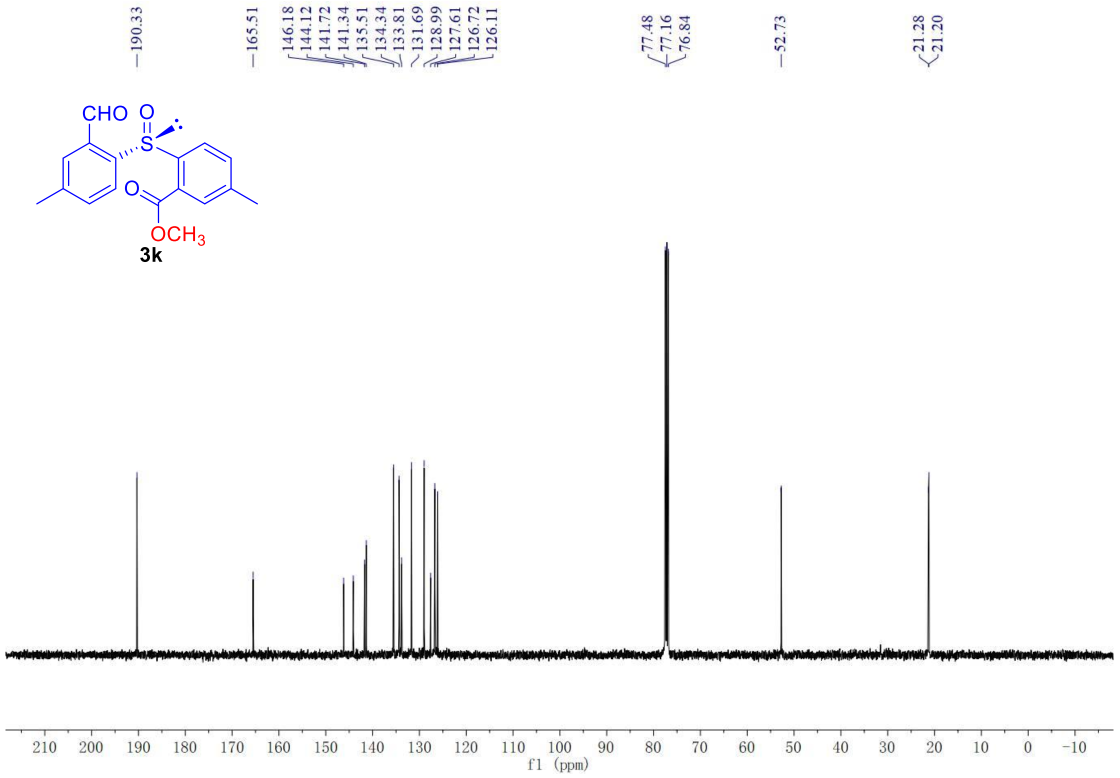

Supplementary Figure 76 ^{13}C NMR (101 MHz, $CDCl_3$) of **3k**

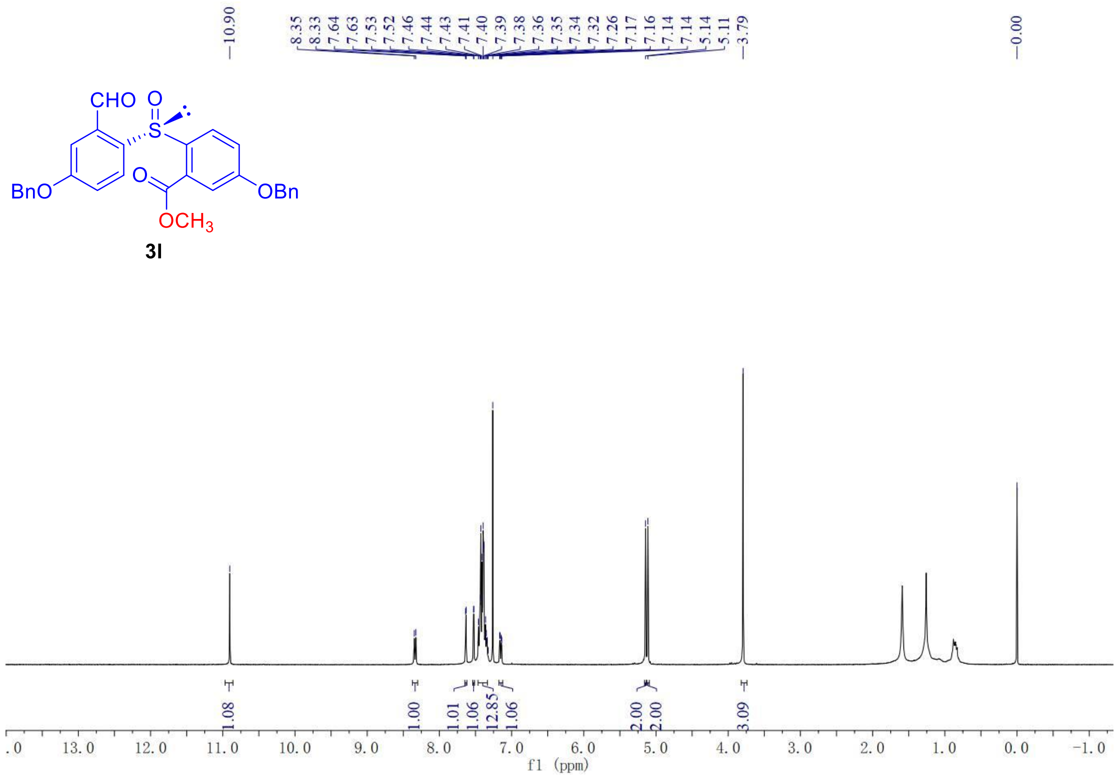

Supplementary Figure 77 ^{1}H NMR (400 MHz, $CDCl_3$) of **3l**

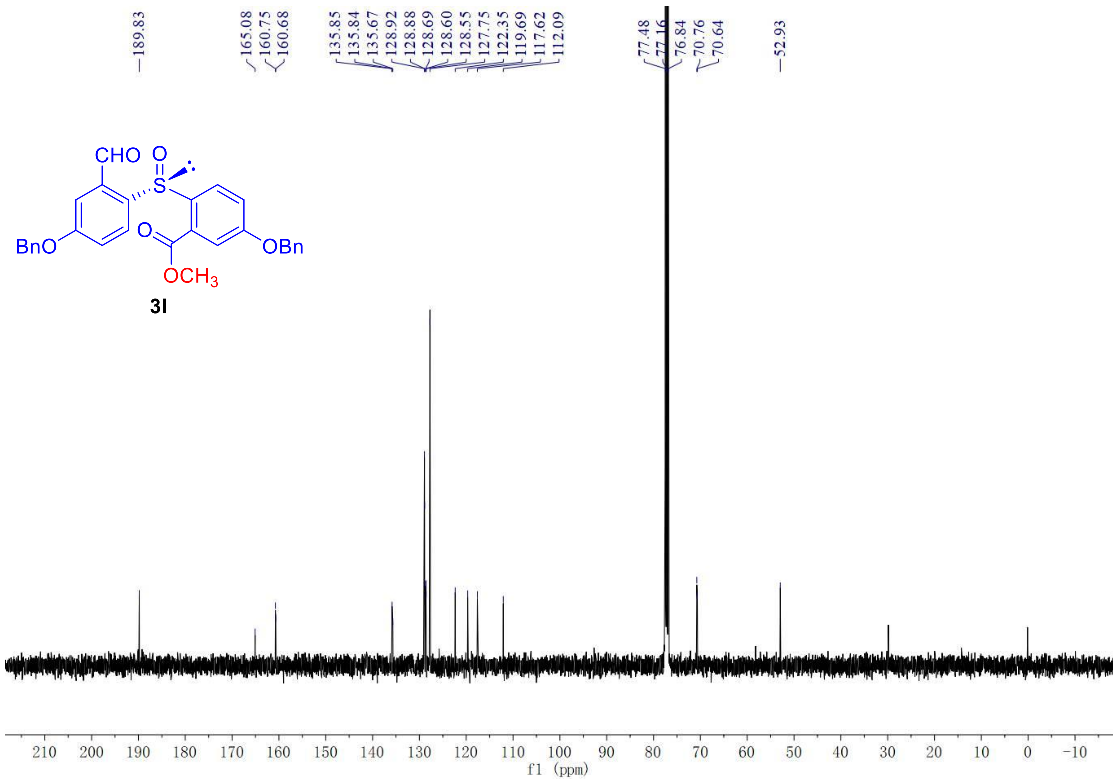

Supplementary Figure 78 ^{13}C NMR (101 MHz, $CDCl_3$) of **3l**

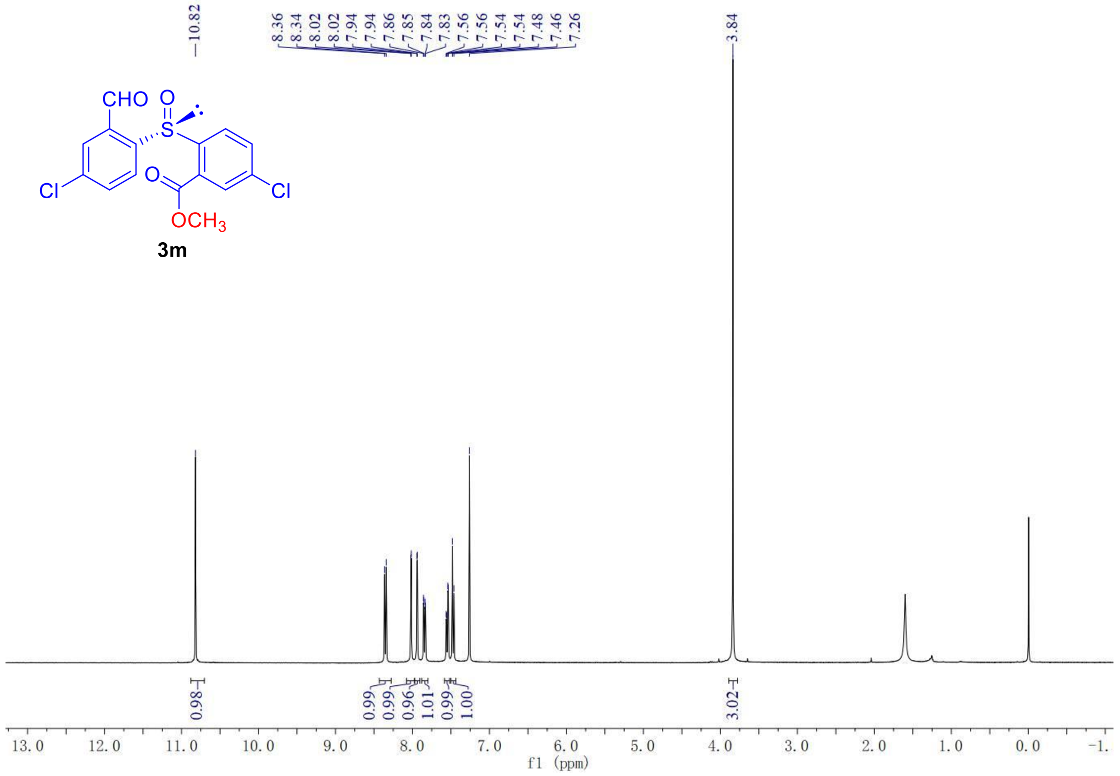

Supplementary Figure 79 ^{1}H NMR (400 MHz, $CDCl_3$) of **3m**

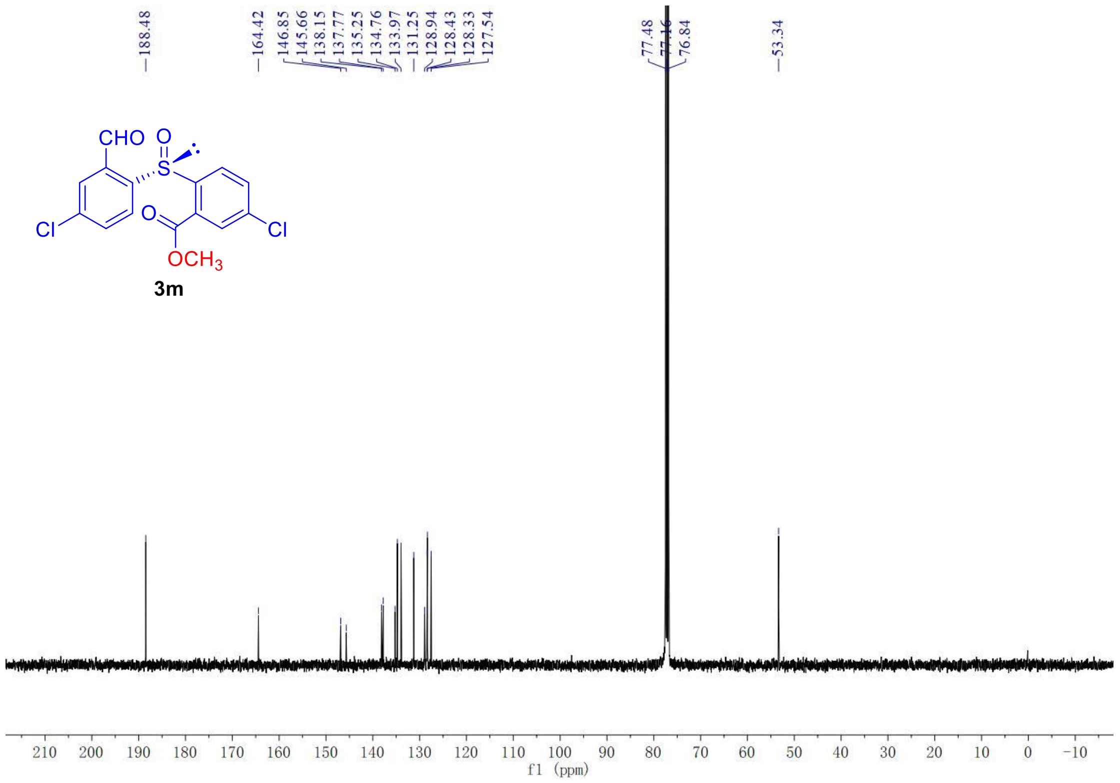

Supplementary Figure 80 ^{13}C NMR (101 MHz, $CDCl_3$) of **3m**

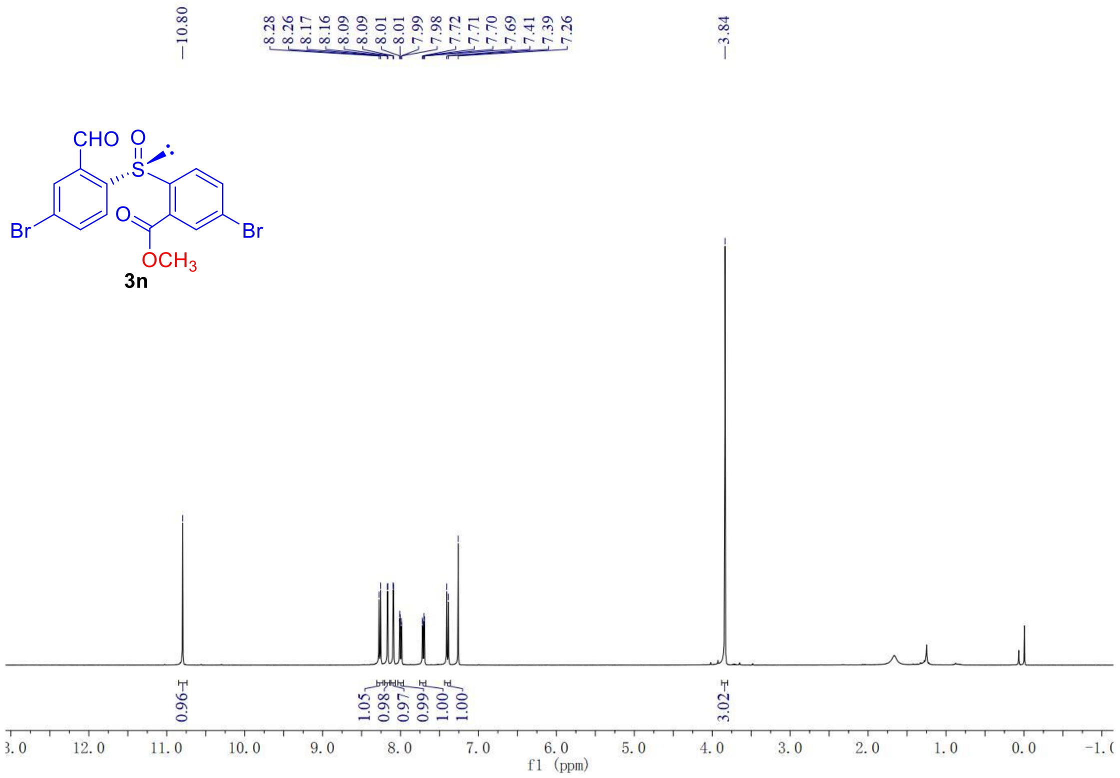

Supplementary Figure 81 ^{1}H NMR (400 MHz, $CDCl_3$) of **3n**

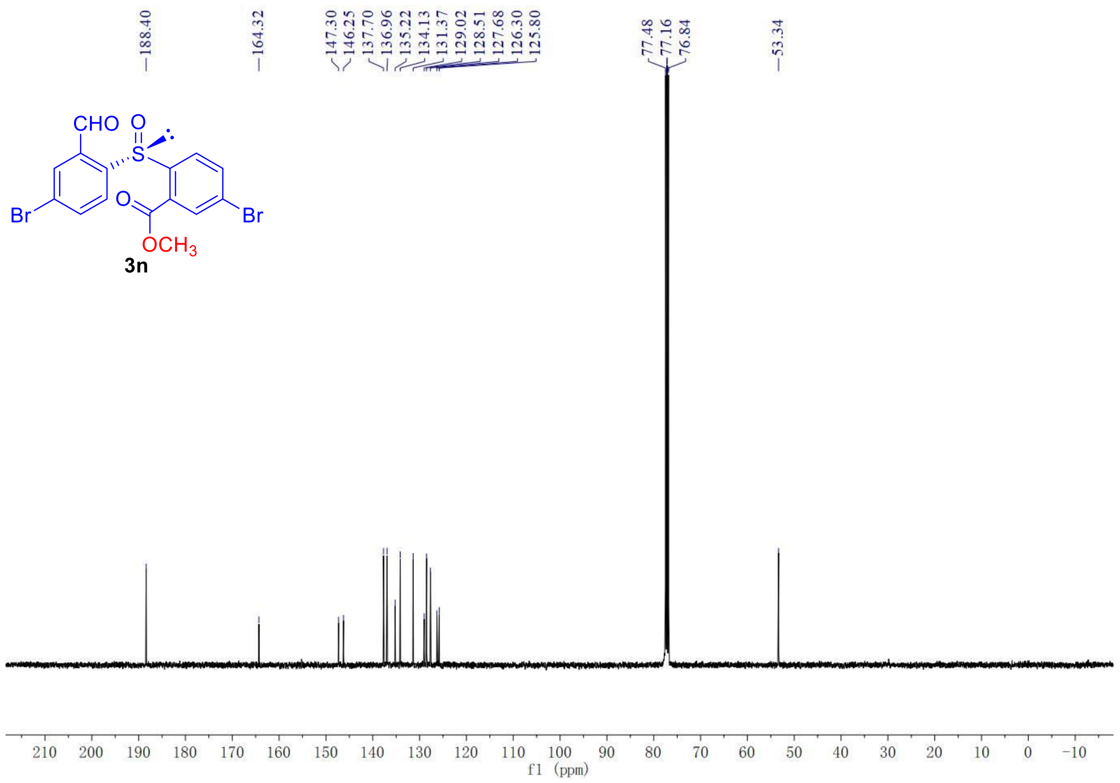

Supplementary Figure 82 ^{13}C NMR (101 MHz, $CDCl_3$) of **3n**

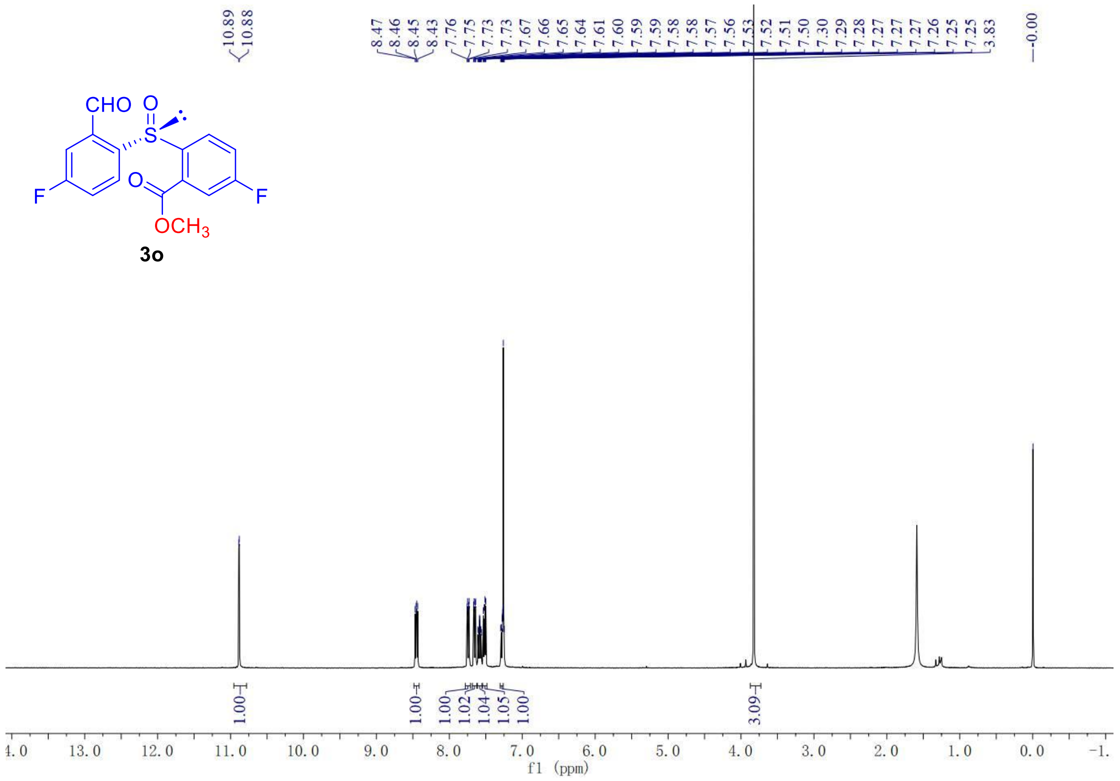

Supplementary Figure 83 ^{1}H NMR (400 MHz, $CDCl_3$) of **3o**

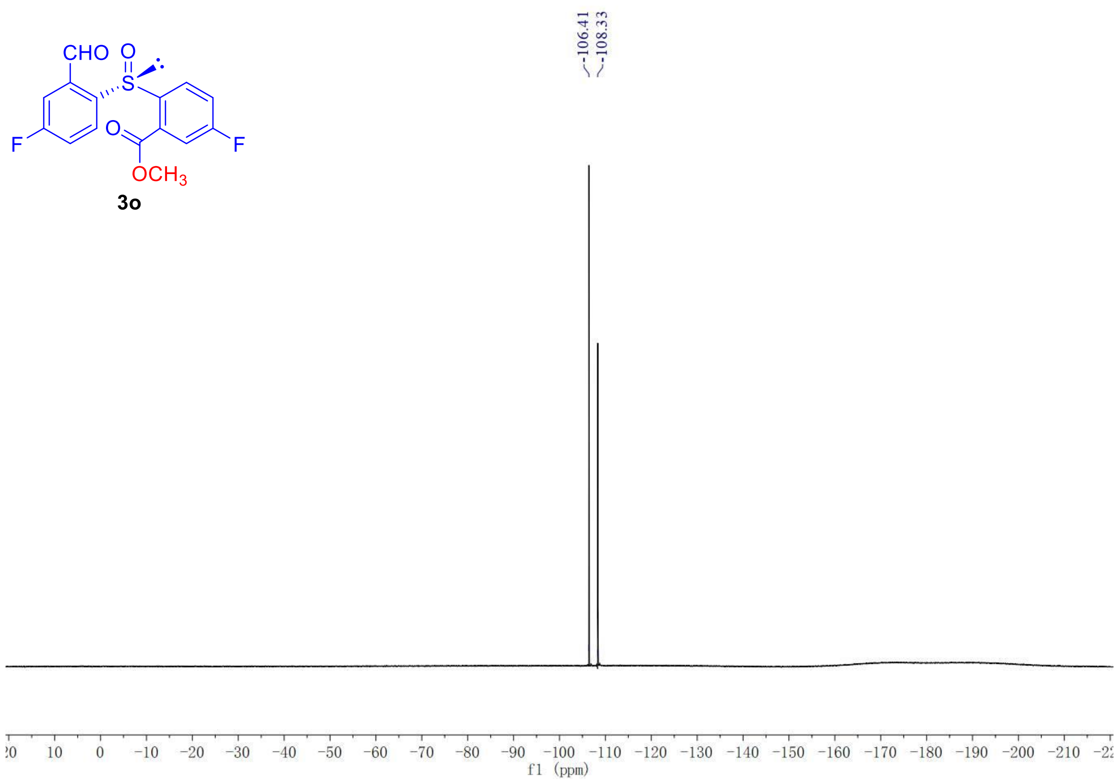

Supplementary Figure 84 ^{19}F NMR (376 MHz, $CDCl_3$) of **3o**

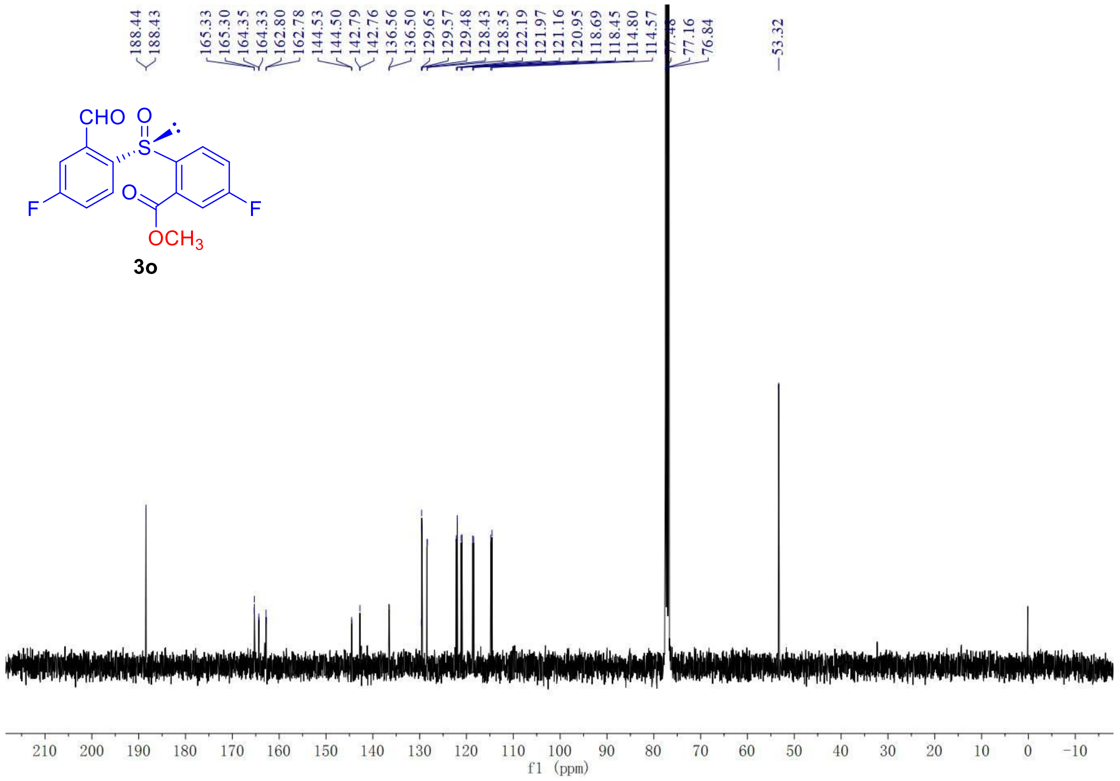

Supplementary Figure 85 ^{13}C NMR (101 MHz, $CDCl_3$) of **3o**

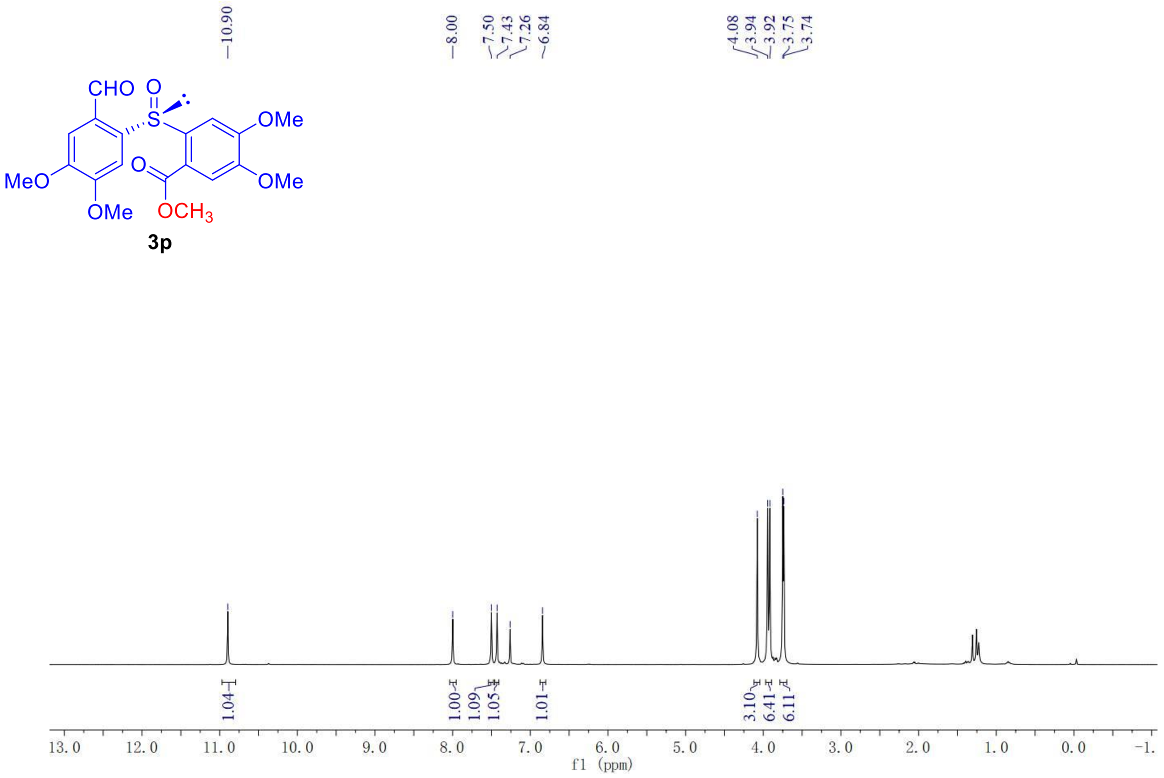

Supplementary Figure 86 ^{1}H NMR (400 MHz, $CDCl_3$) of **3p**

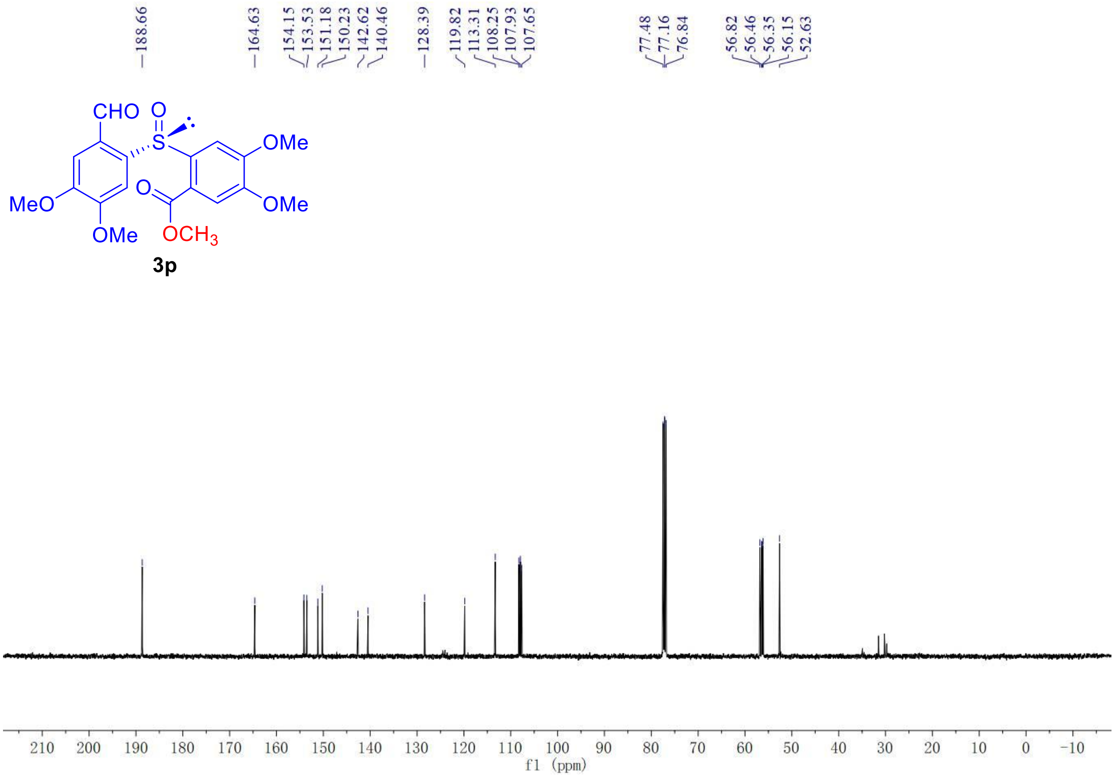

Supplementary Figure 87 ^{13}C NMR (101 MHz, $CDCl_3$) of **3p**

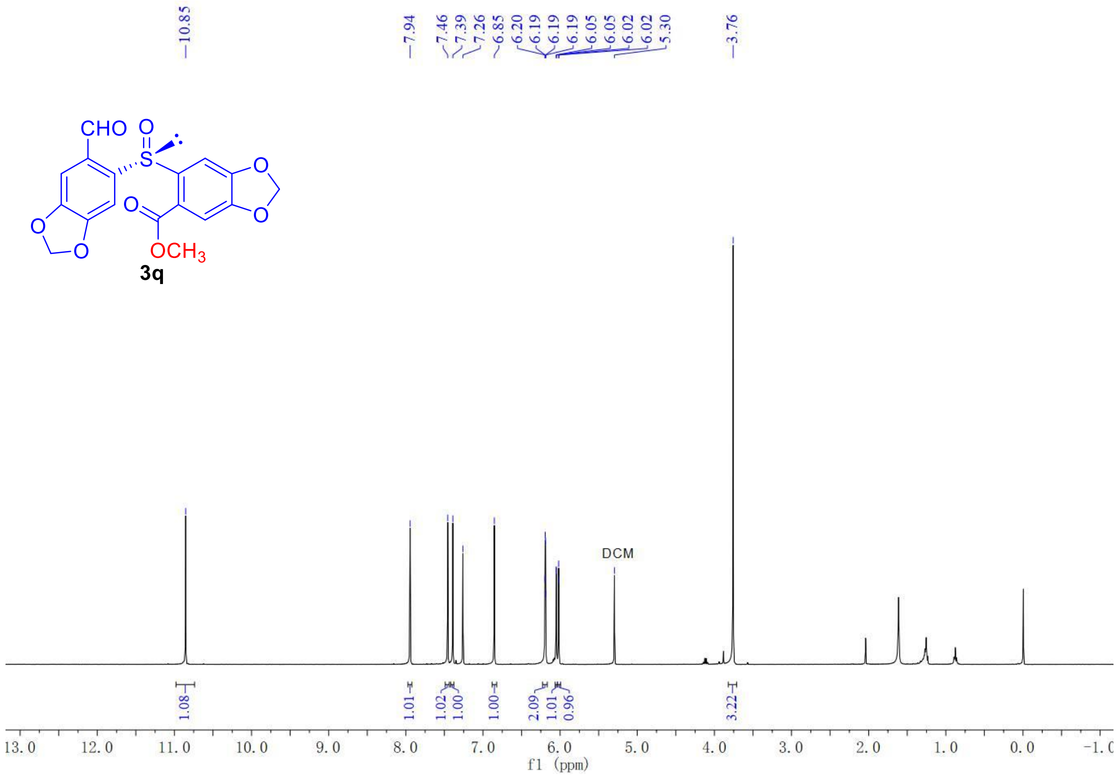

Supplementary Figure 88 ^{1}H NMR (400 MHz, $CDCl_3$) of **3q**

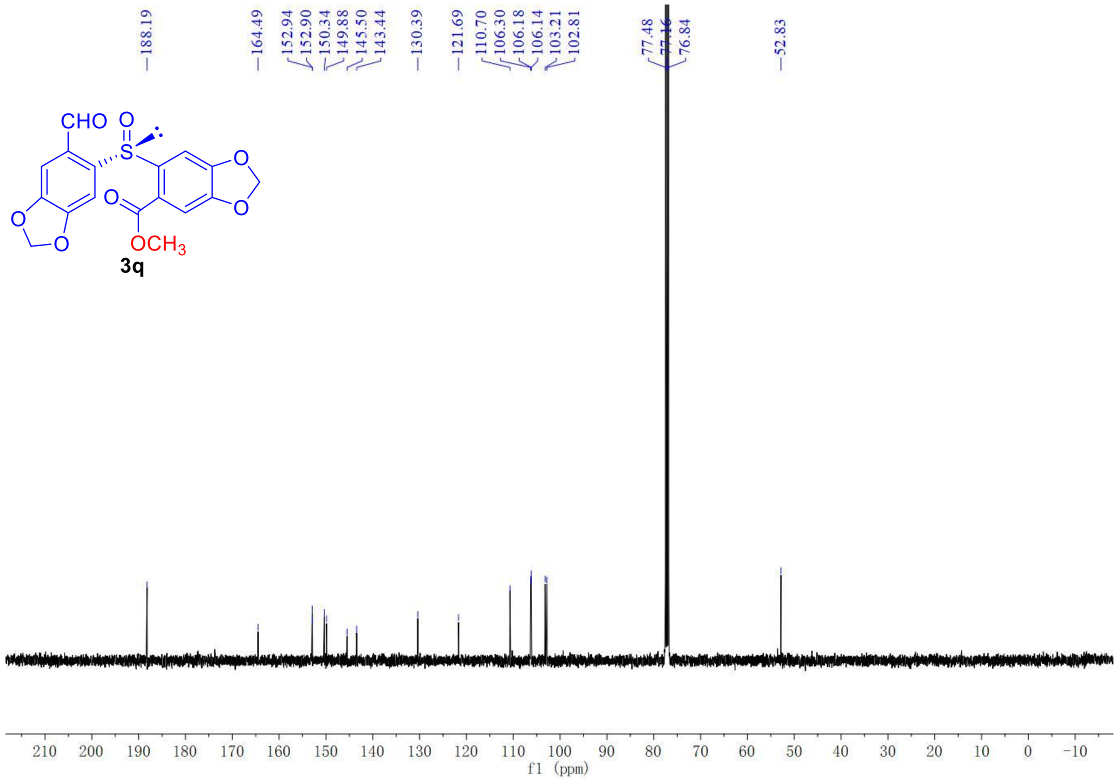

Supplementary Figure 89 ^{13}C NMR (101 MHz, $CDCl_3$) of **3q**

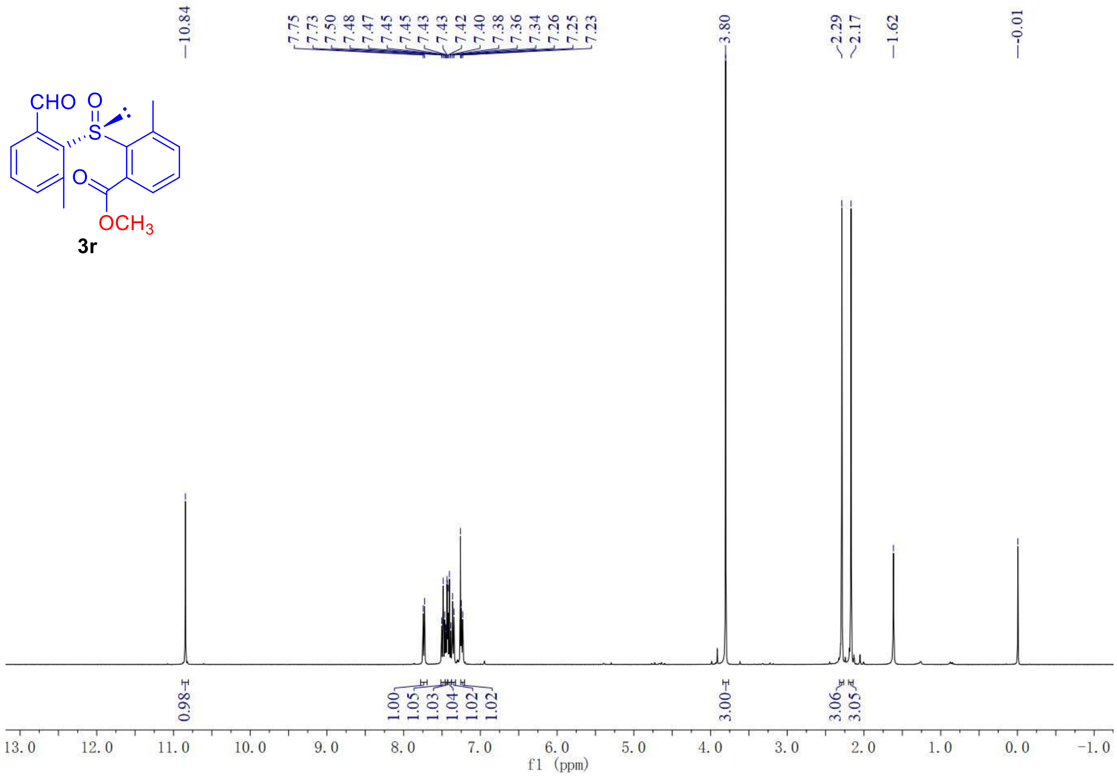

Supplementary Figure 90 ^{1}H NMR (400 MHz, $CDCl_3$) of **3r**

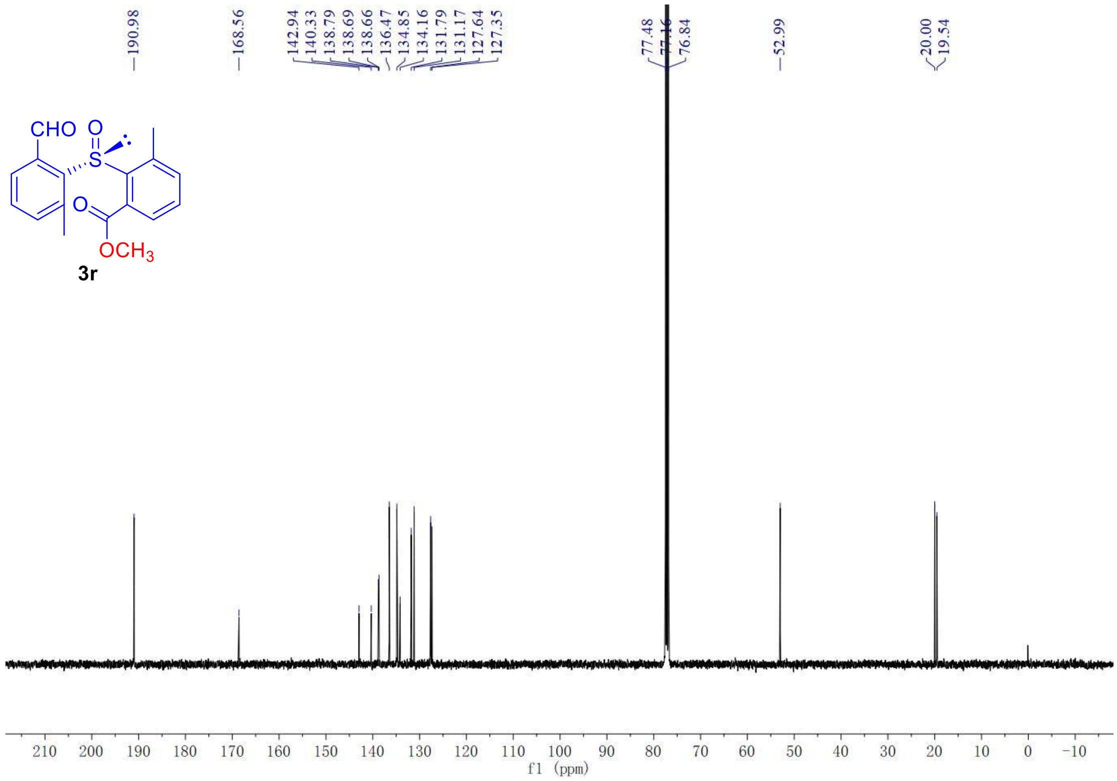

Supplementary Figure 91 ^{13}C NMR (101 MHz, $CDCl_3$) of **3r**

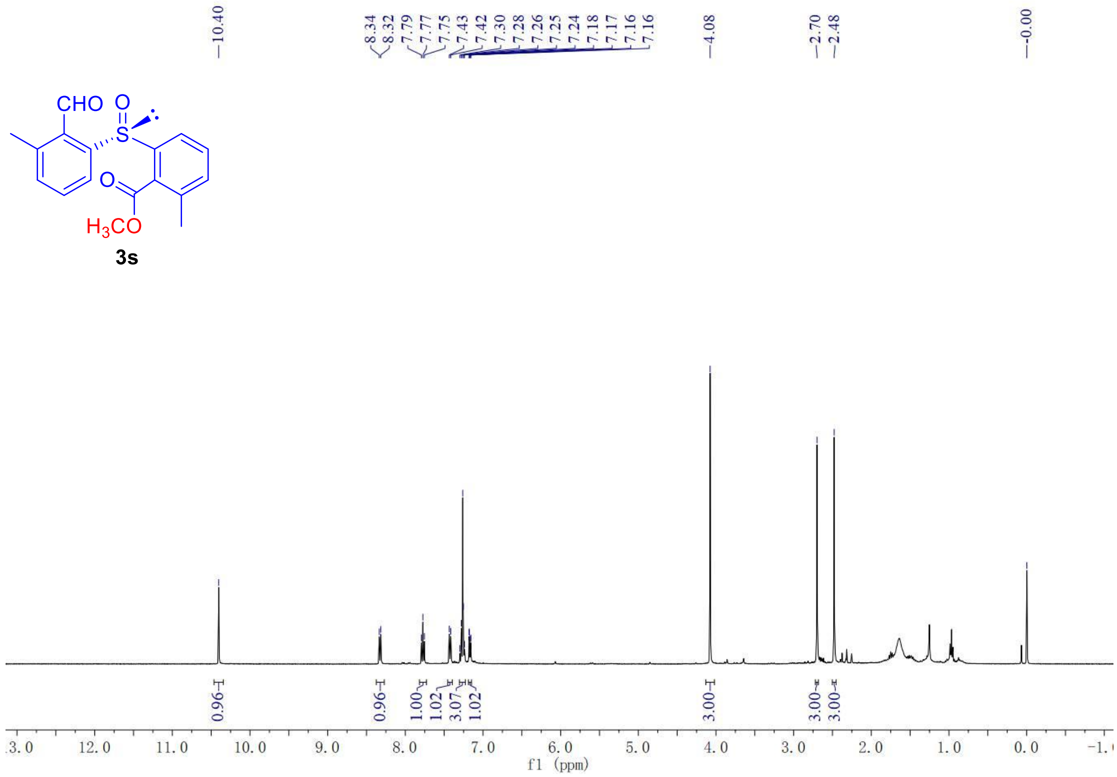

Supplementary Figure 92 ^{1}H NMR (400 MHz, $CDCl_3$) of **3s**

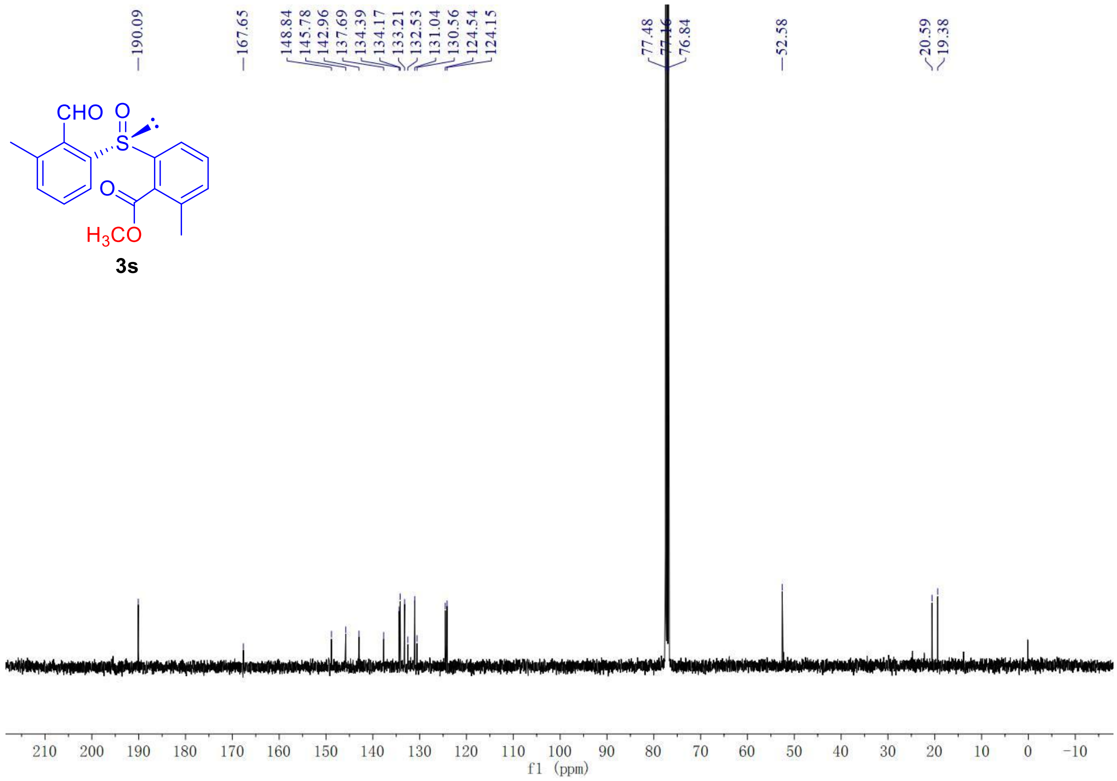

Supplementary Figure 93 ^{13}C NMR (101 MHz, $CDCl_3$) of **3s**

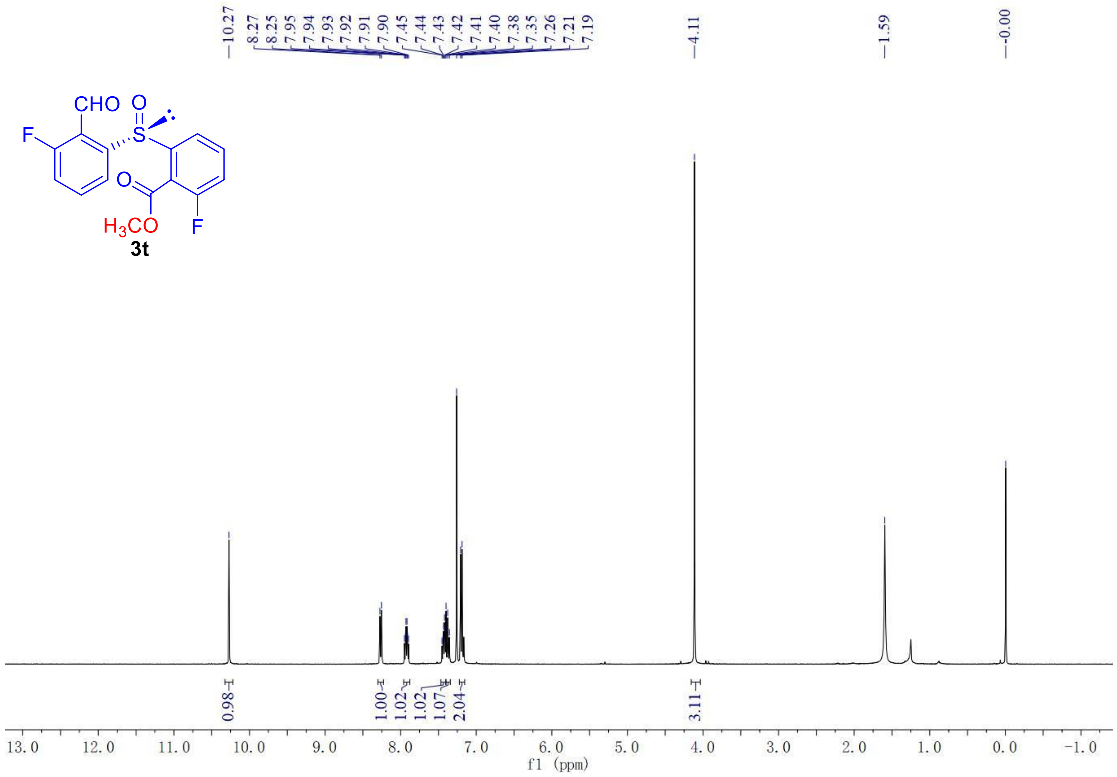

Supplementary Figure 94 ^{1}H NMR (400 MHz, $CDCl_3$) of **3s**

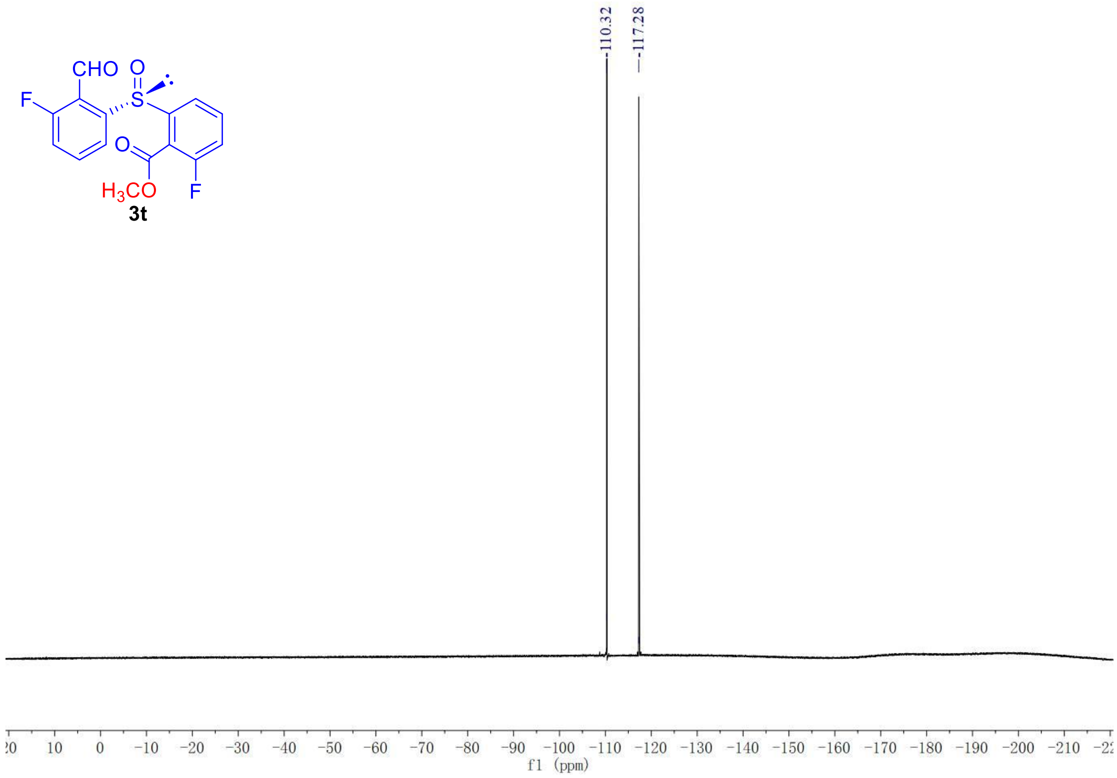

Supplementary Figure 95 ^{19}F NMR (101 MHz, $CDCl_3$) of **3s**

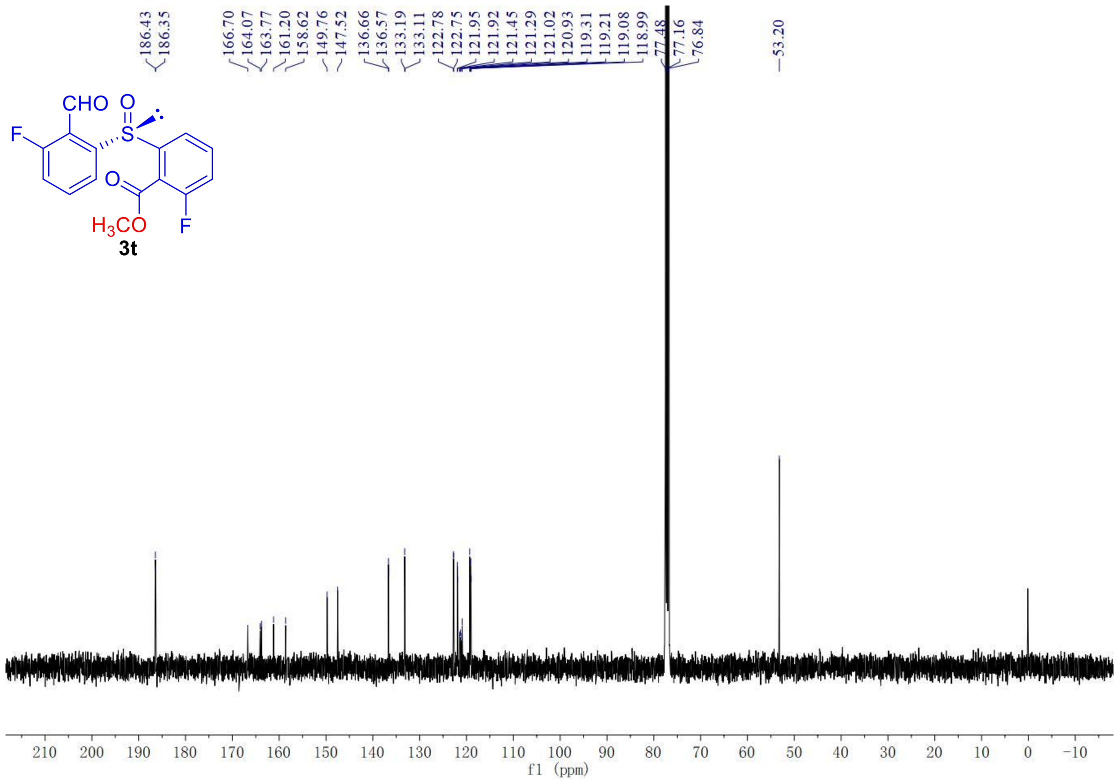

Supplementary Figure 96 ^{13}C NMR (101 MHz, $CDCl_3$) of **3t**

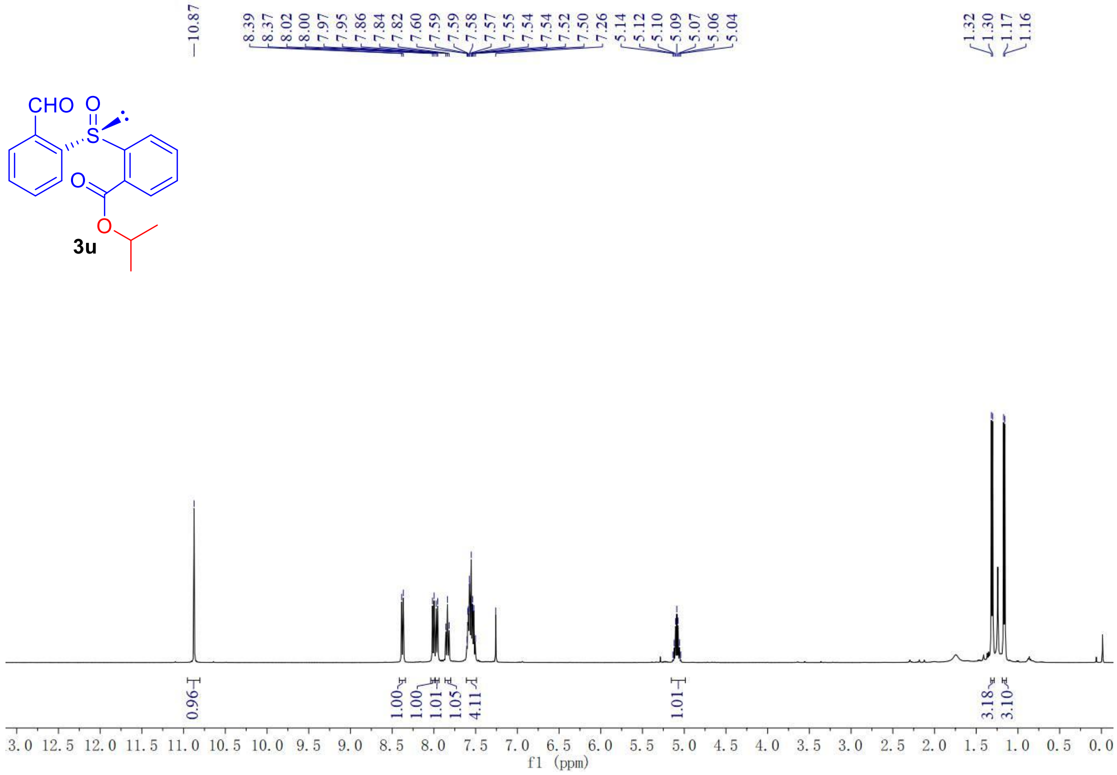

Supplementary Figure 97 ^{1}H NMR (400 MHz, $CDCl_3$) of **3u**

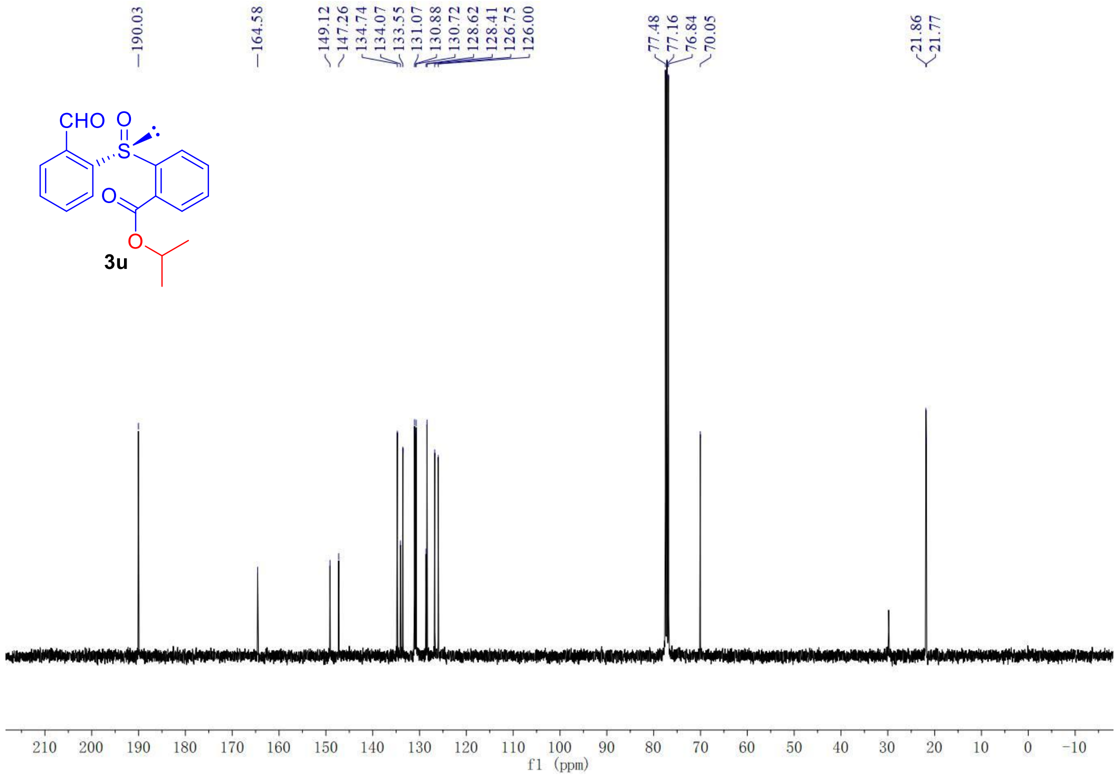

Supplementary Figure 99 ^{13}C NMR (101 MHz, $CDCl_3$) of **3u**

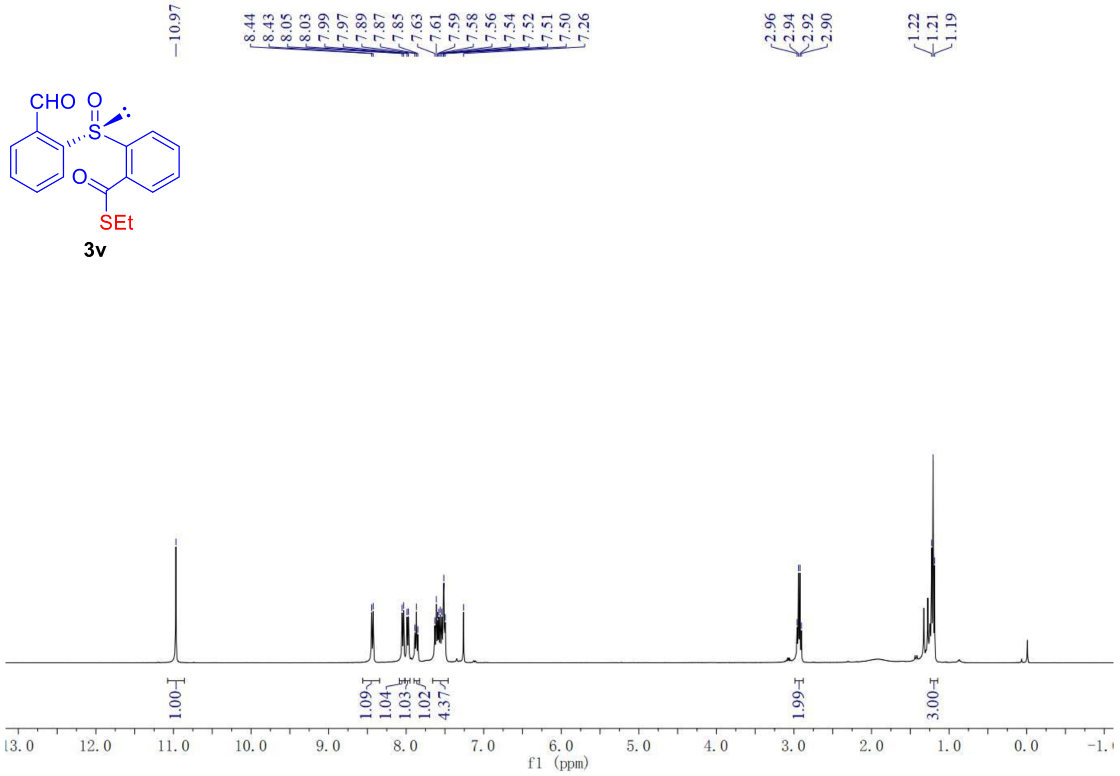

Supplementary Figure 99 ^{1}H NMR (400 MHz, $CDCl_3$) of **3v**

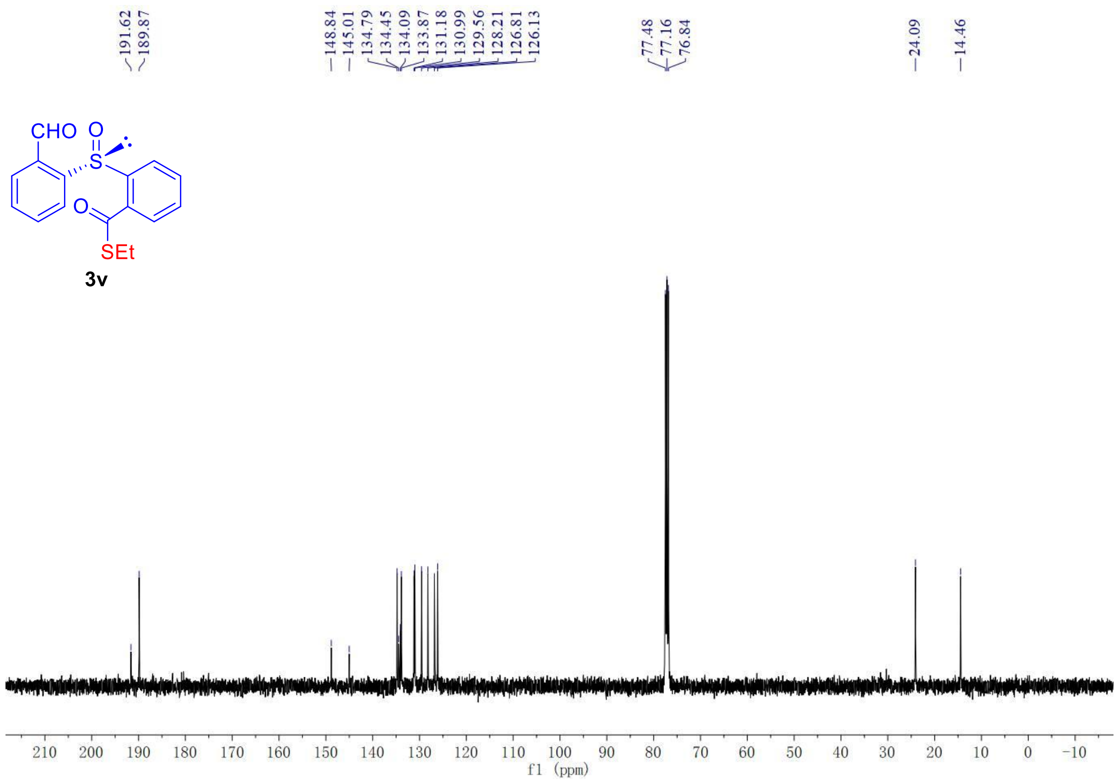

Supplementary Figure 100 ^{13}C NMR (101 MHz, $CDCl_3$) of **3v**

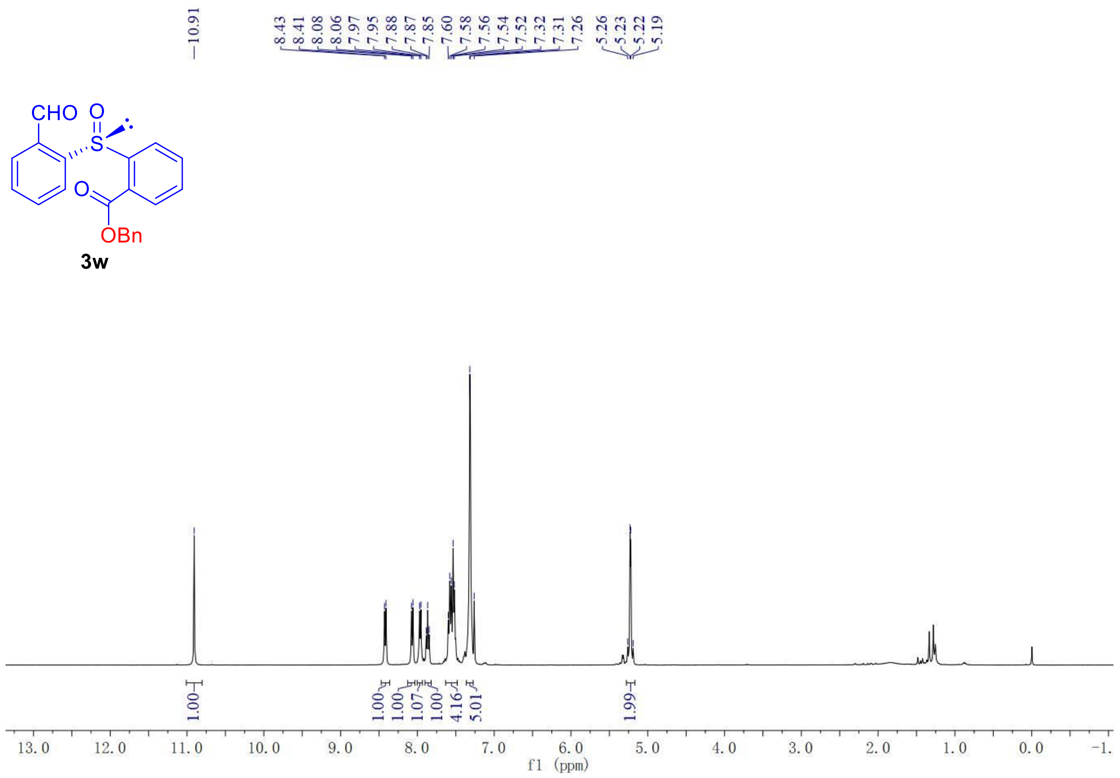

Supplementary Figure 101 ^{1}H NMR (400 MHz, $CDCl_3$) of **3w**

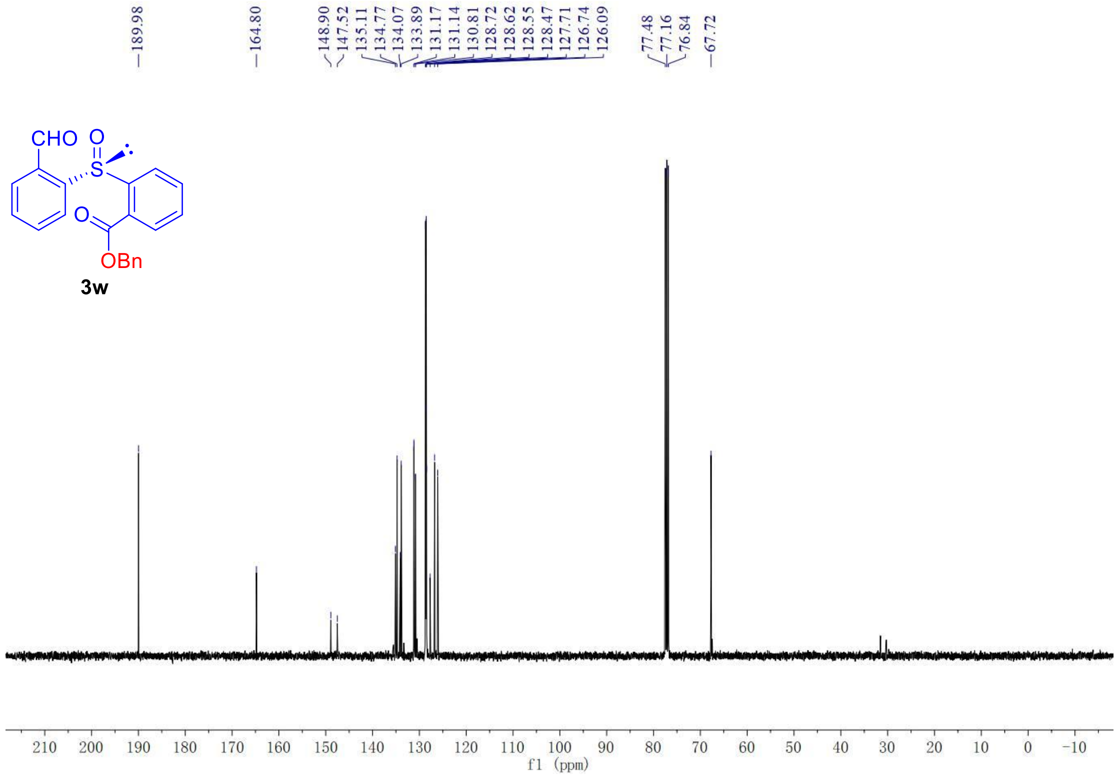

Supplementary Figure 102 ^{13}C NMR (101 MHz, $CDCl_3$) of **3w**

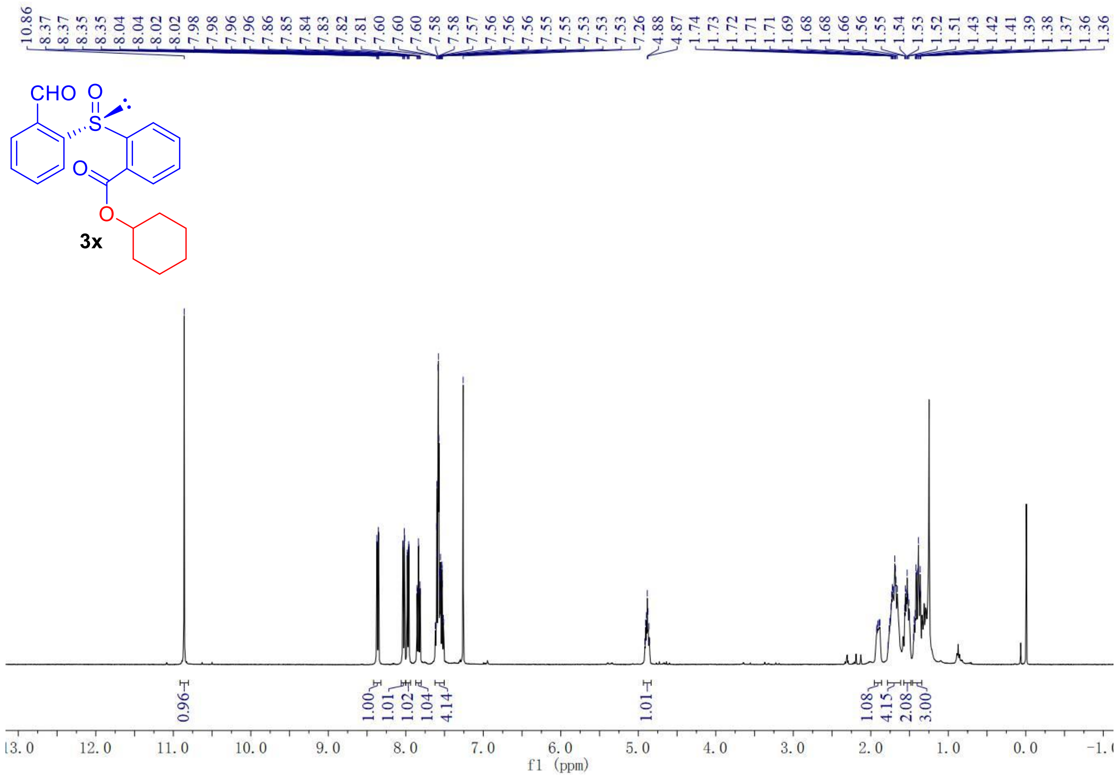

Supplementary Figure 103 ^{1}H NMR (400 MHz, $CDCl_3$) of **3x**

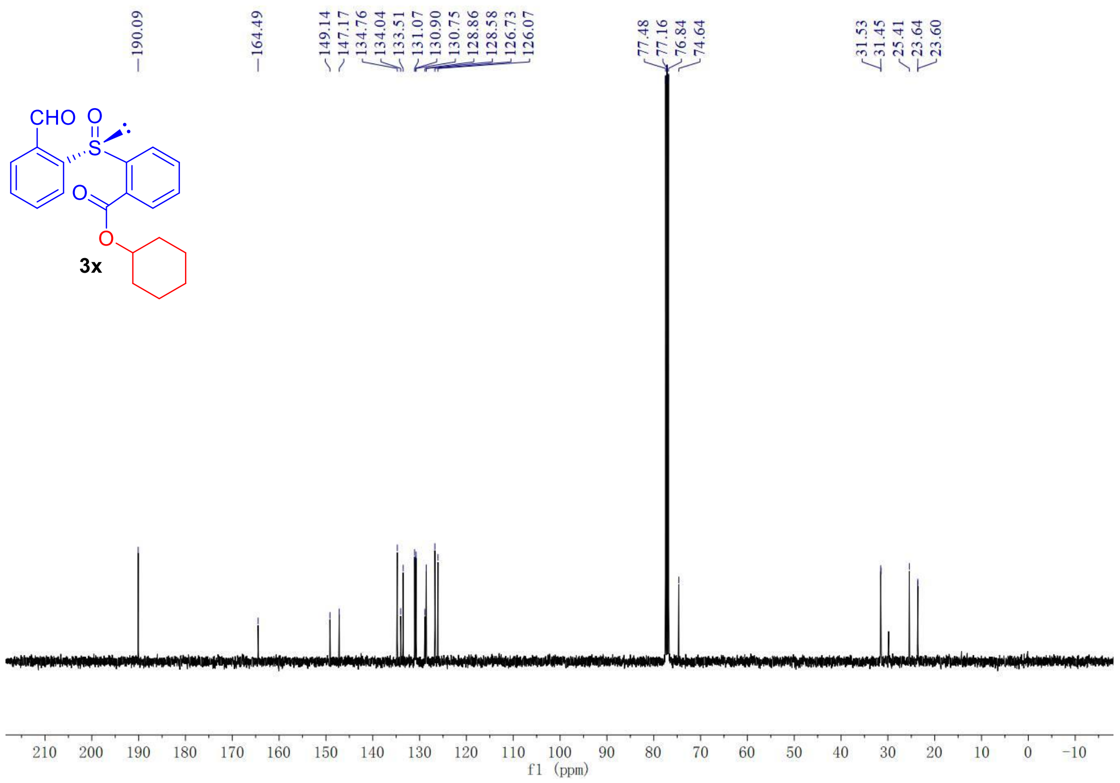

Supplementary Figure 104 ^{13}C NMR (101 MHz, $CDCl_3$) of **3x**

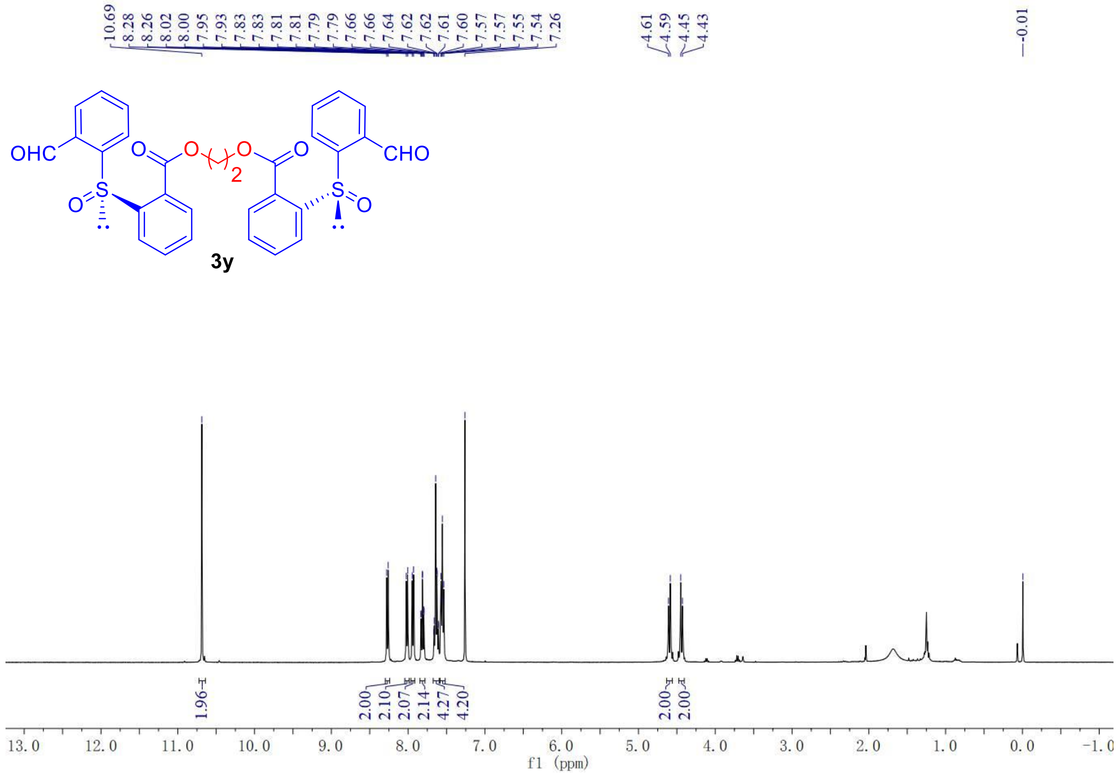

Supplementary Figure 105 ^{1}H NMR (400 MHz, $CDCl_3$) of **3y**

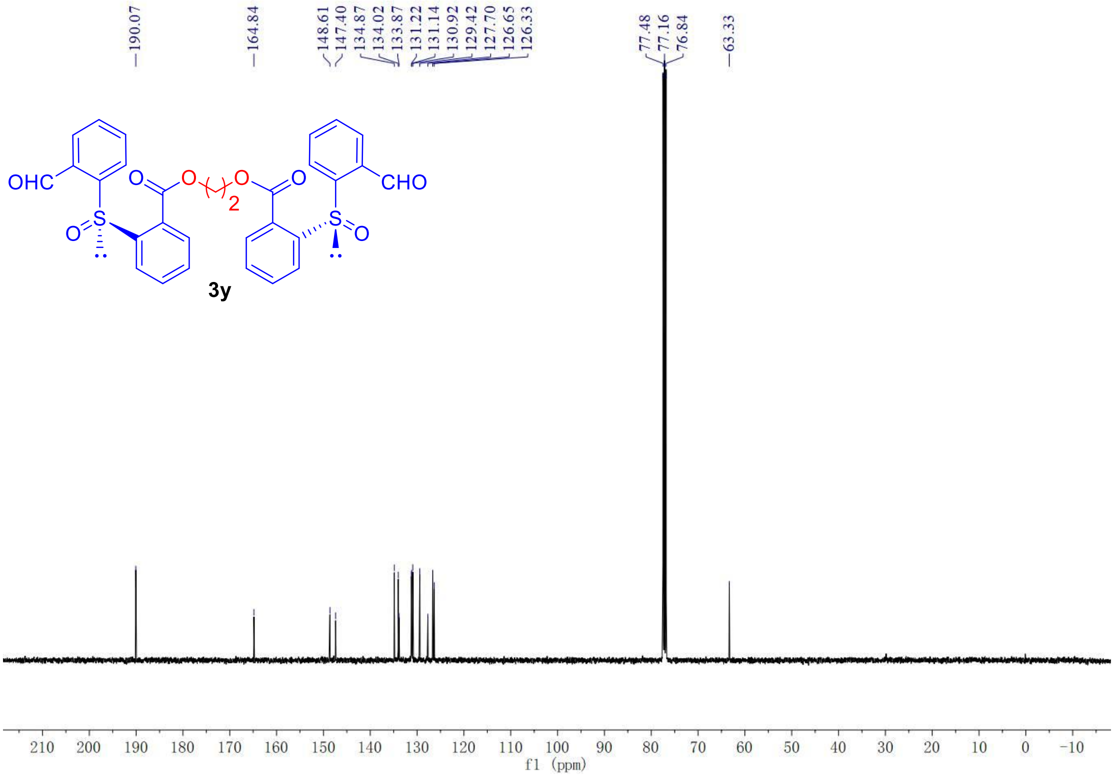

Supplementary Figure 106 ^{13}C NMR (101 MHz, $CDCl_3$) of **3y**

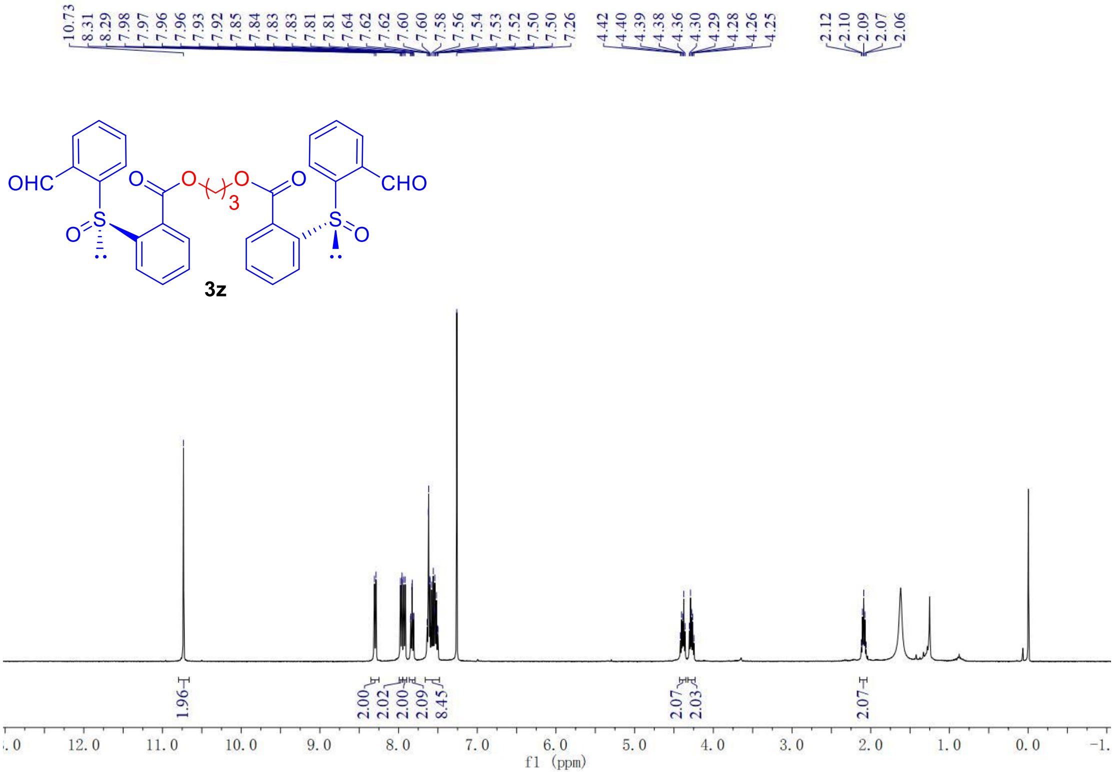

Supplementary Figure 107 ^{1}H NMR (400 MHz, $CDCl_3$) of **3z**

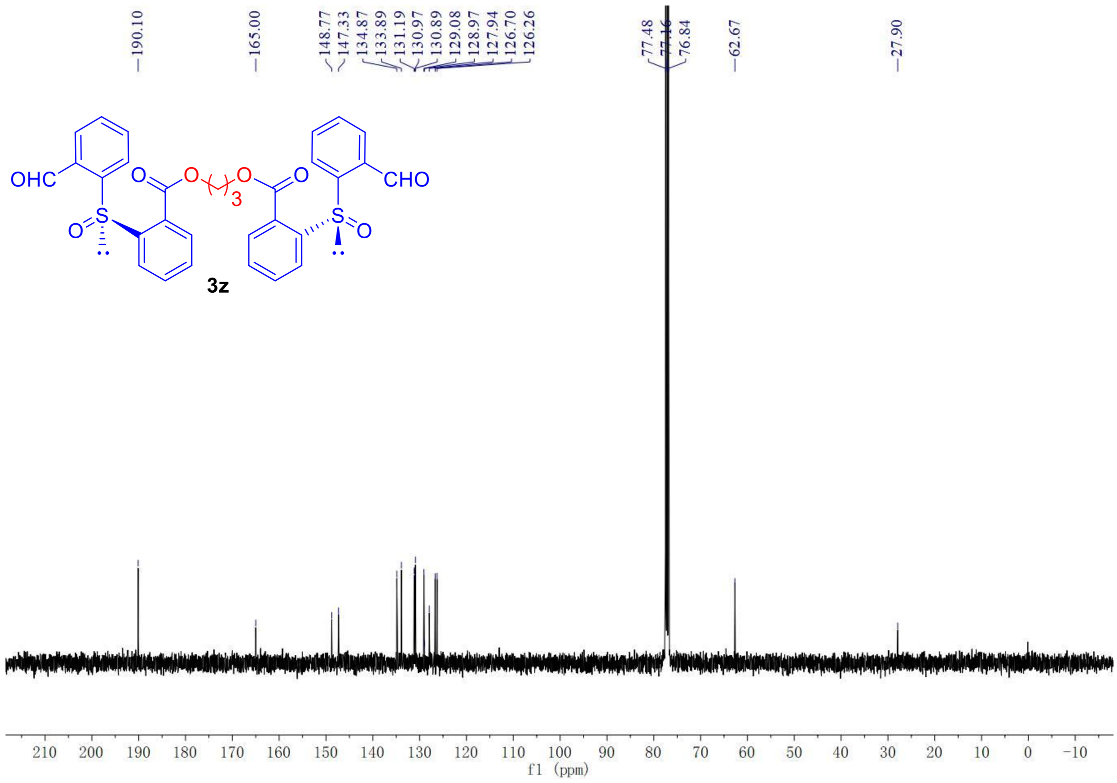

Supplementary Figure 108 ^{13}C NMR (101 MHz, $CDCl_3$) of **3z**

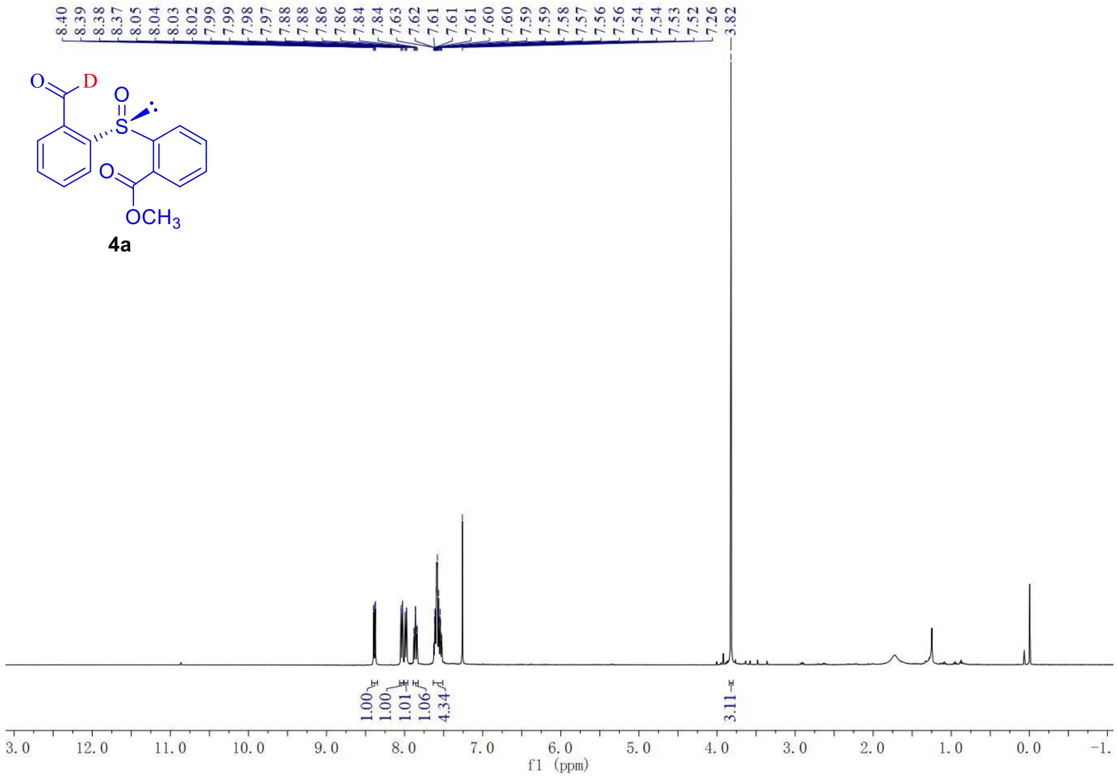

Supplementary Figure 109 ^{1}H NMR (400 MHz, $CDCl_3$) of **4a**

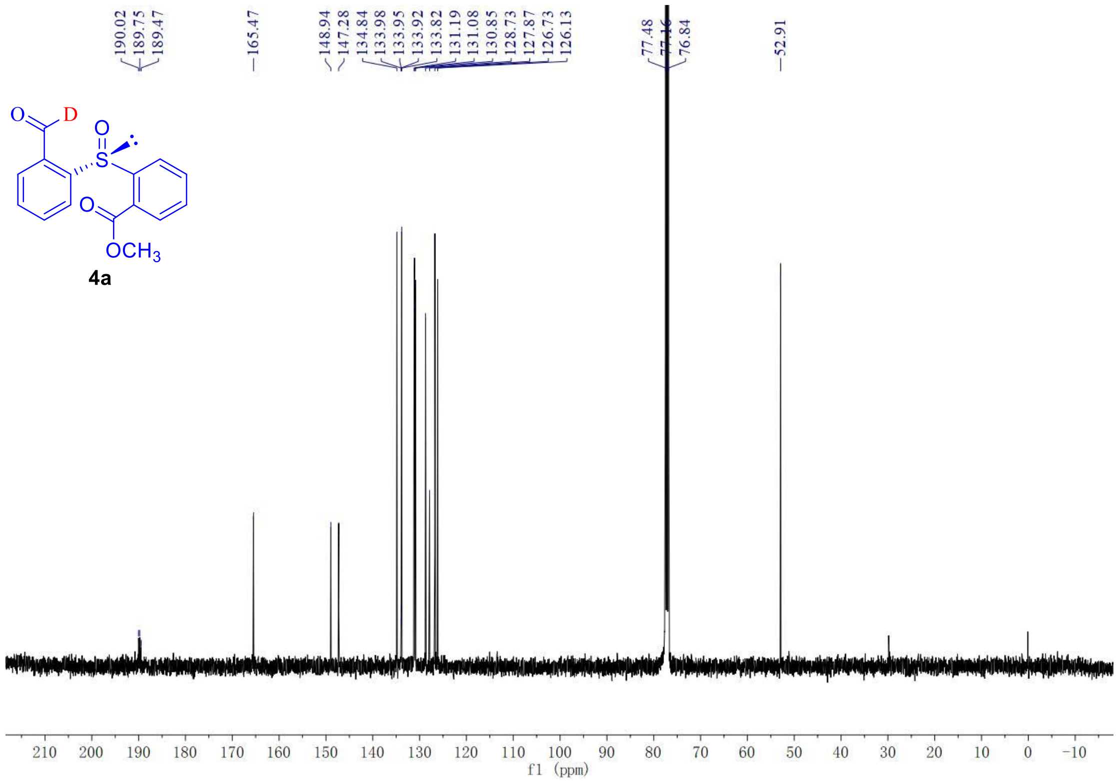

Supplementary Figure 110 ^{13}C NMR (101 MHz, $CDCl_3$) of **4a**

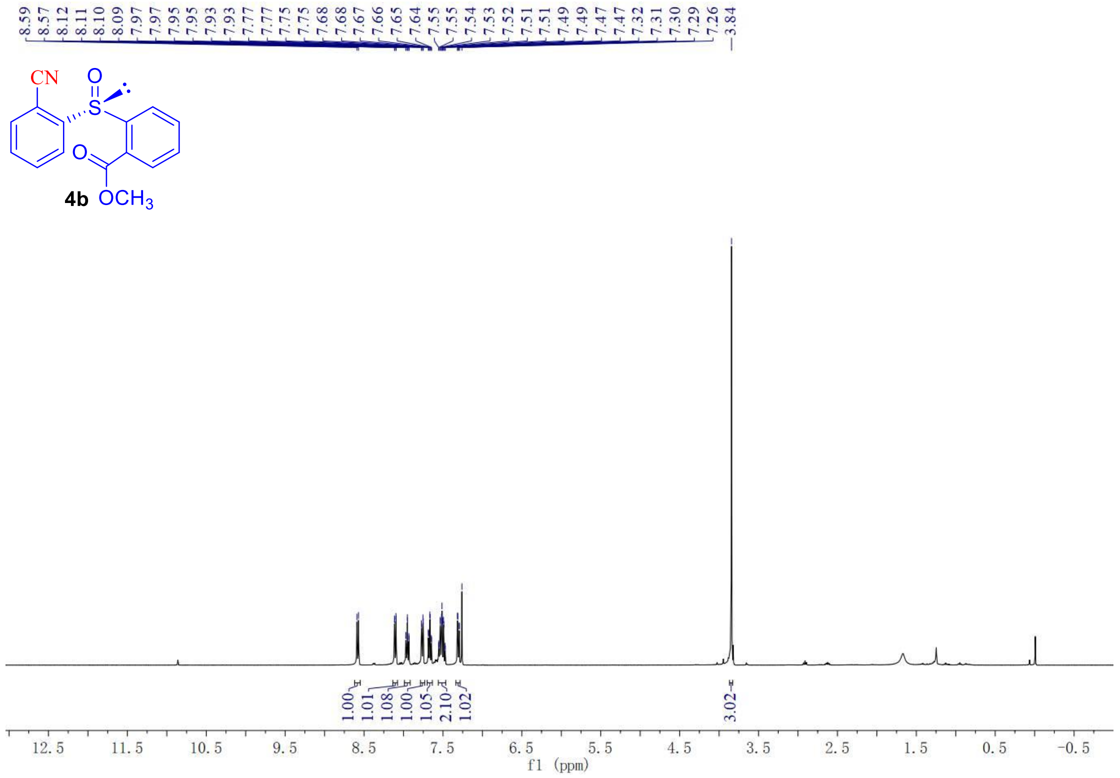

Supplementary Figure 111 ^{1}H NMR (400 MHz, $CDCl_3$) of **4b**

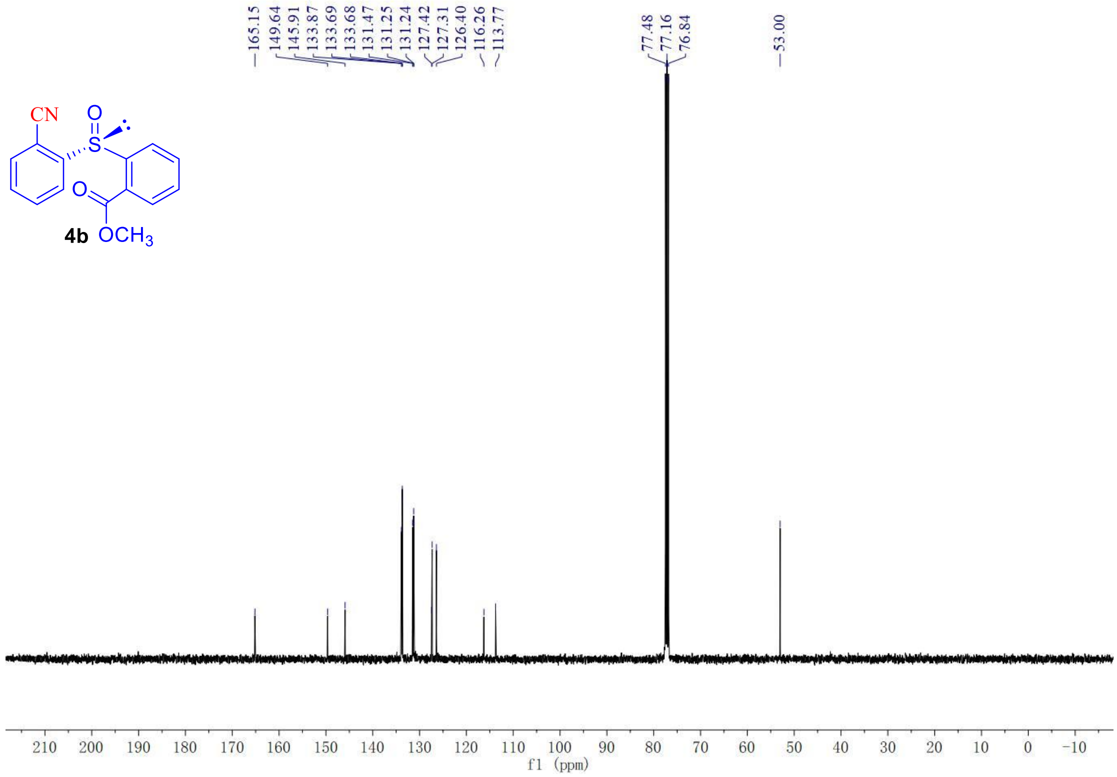

Supplementary Figure 112 ^{13}C NMR (101 MHz, $CDCl_3$) of **4b**

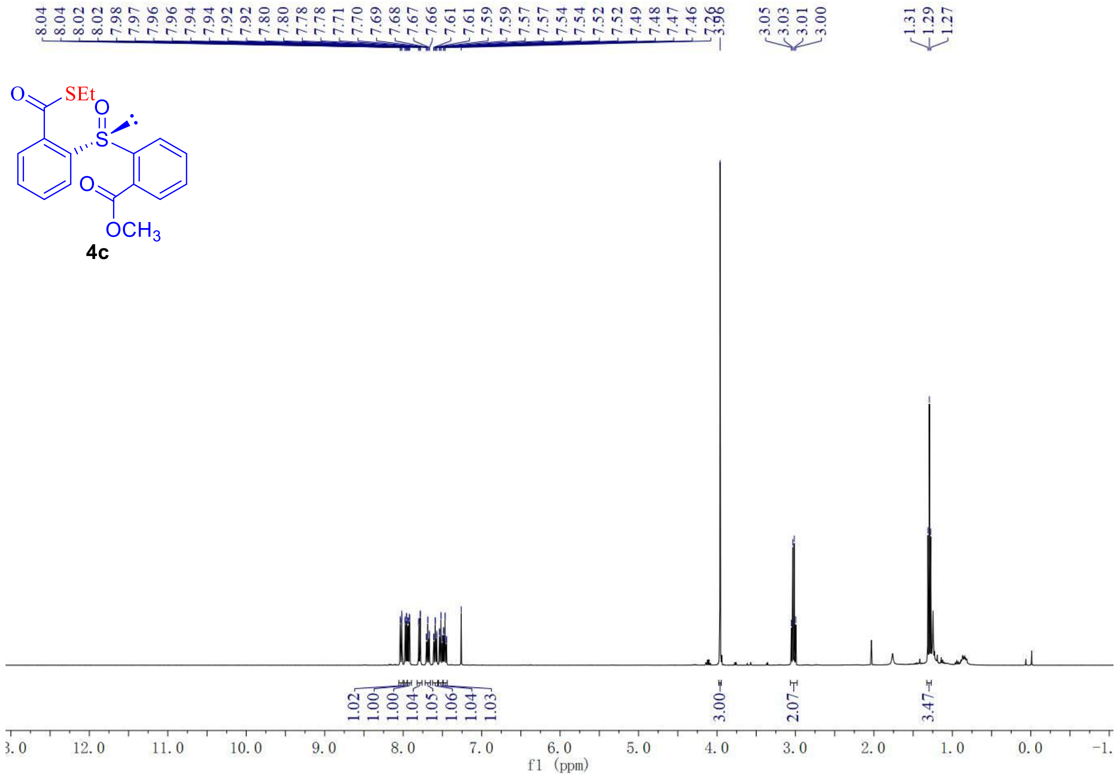

Supplementary Figure 113 ^{1}H NMR (400 MHz, $CDCl_3$) of **4c**

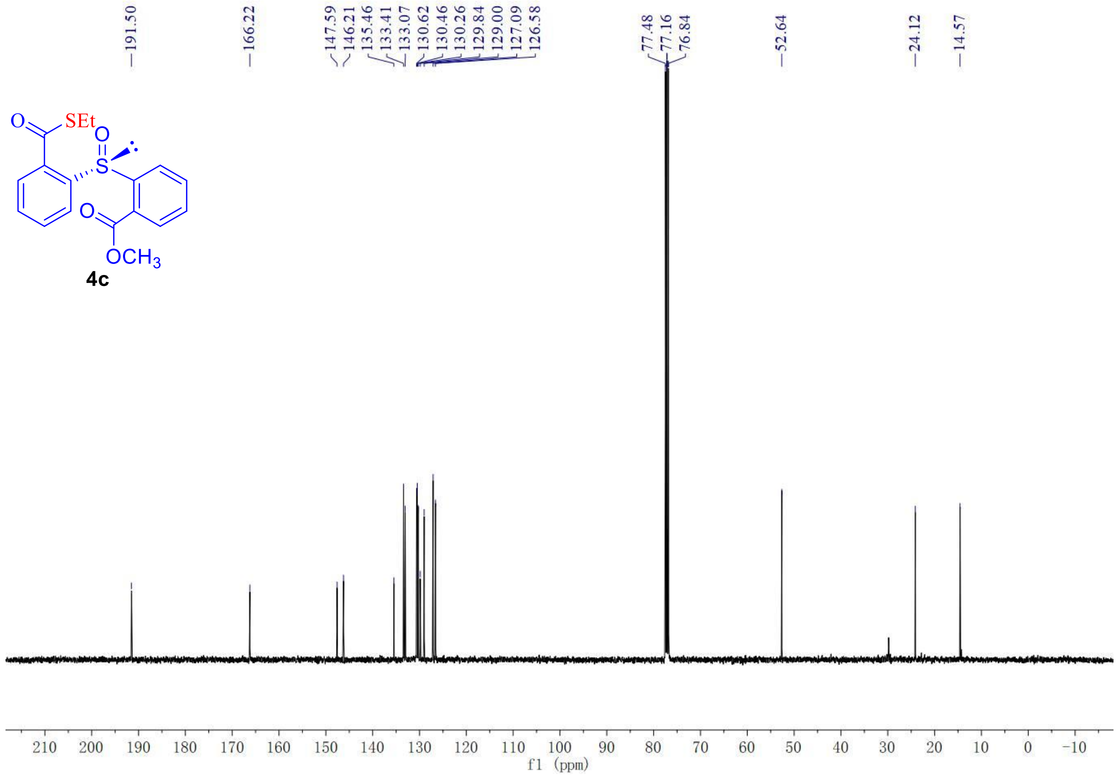

Supplementary Figure 114 ^{13}C NMR (101 MHz, $CDCl_3$) of **4c**

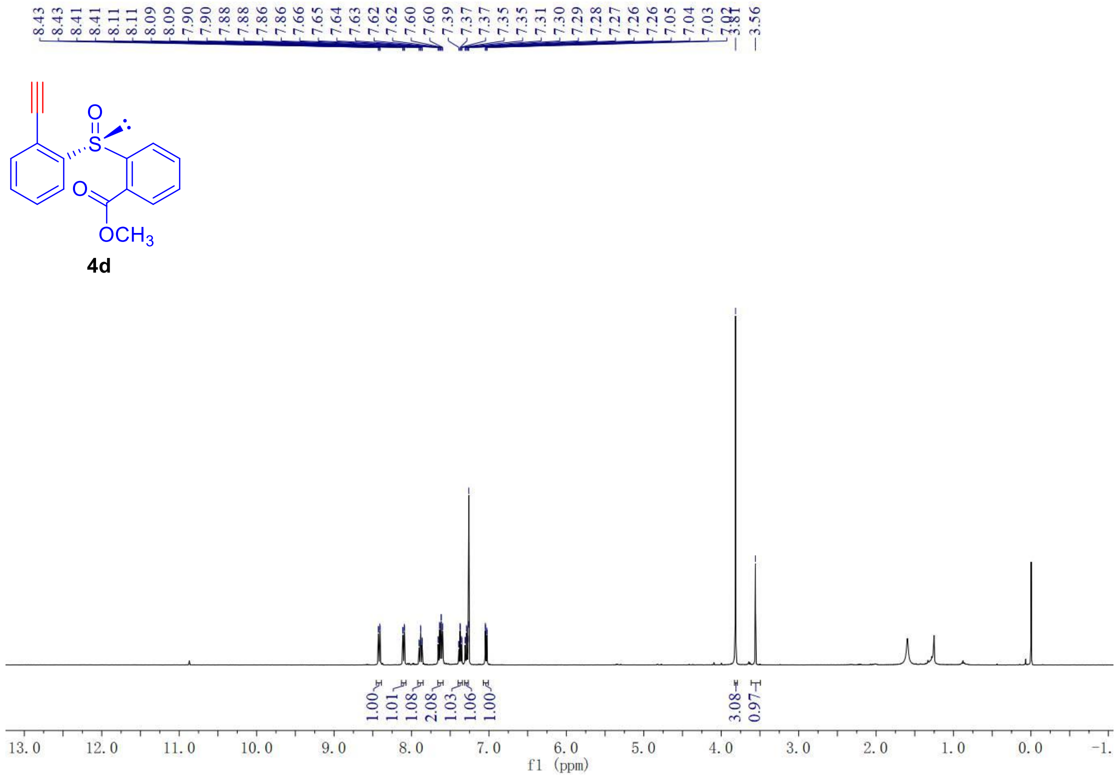

Supplementary Figure 115 ^{1}H NMR (400 MHz, $CDCl_3$) of **4d**

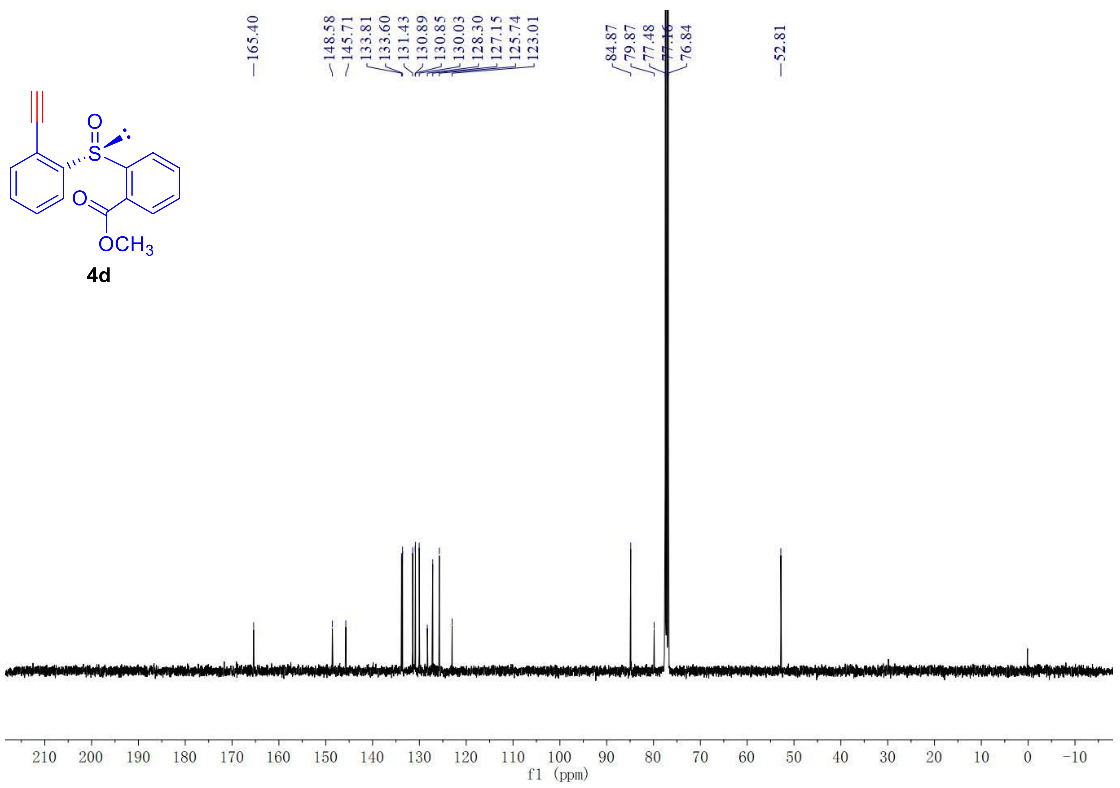

Supplementary Figure 116 ^{13}C NMR (101 MHz, $CDCl_3$) of **4d**

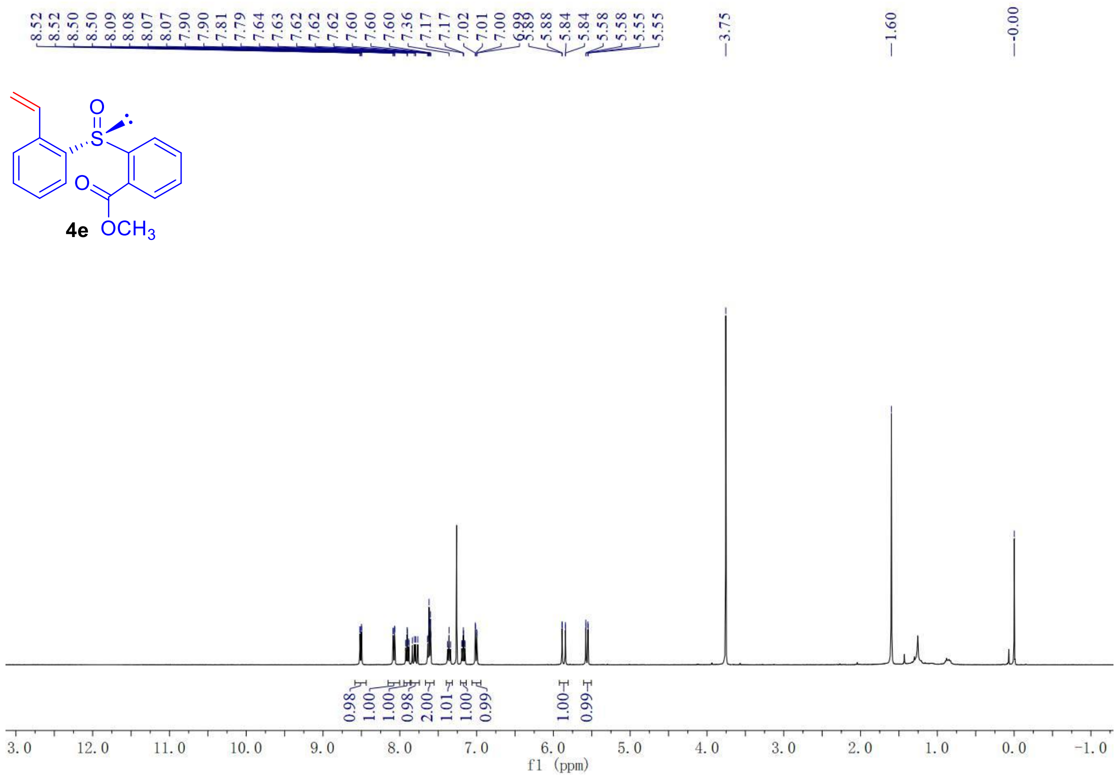

Supplementary Figure 117 ^{1}H NMR (400 MHz, $CDCl_3$) of **4e**

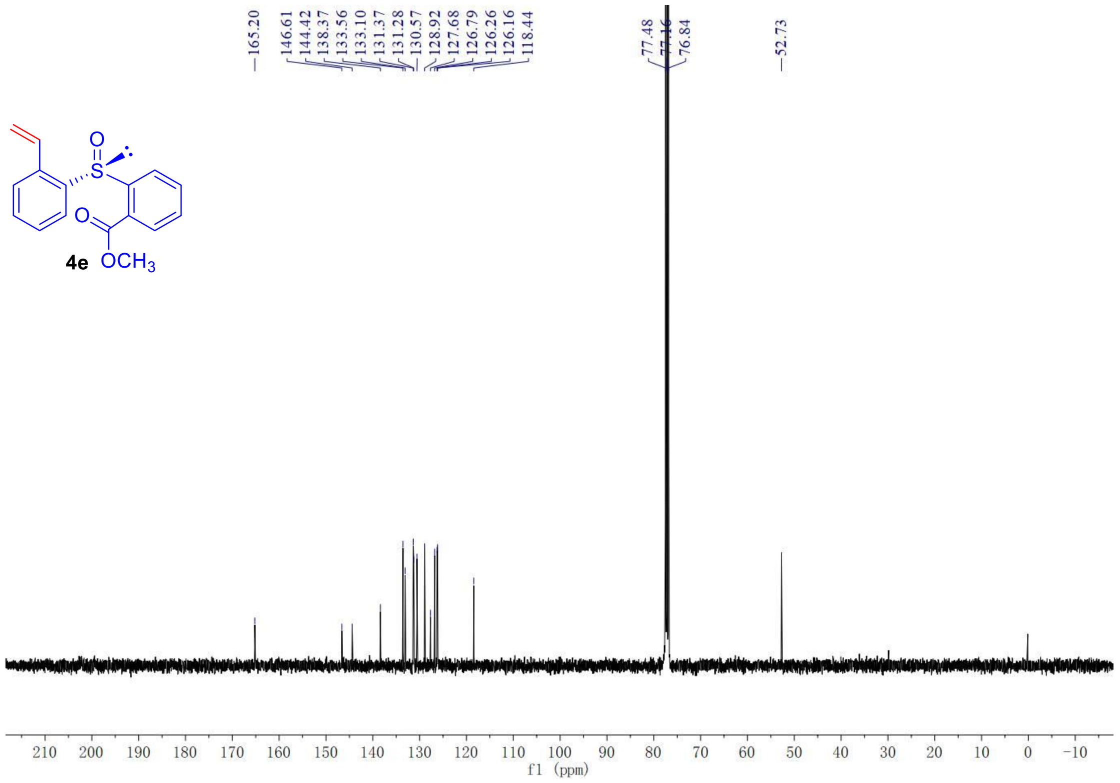

Supplementary Figure 118 ^{13}C NMR (101 MHz, $CDCl_3$) of **4e**

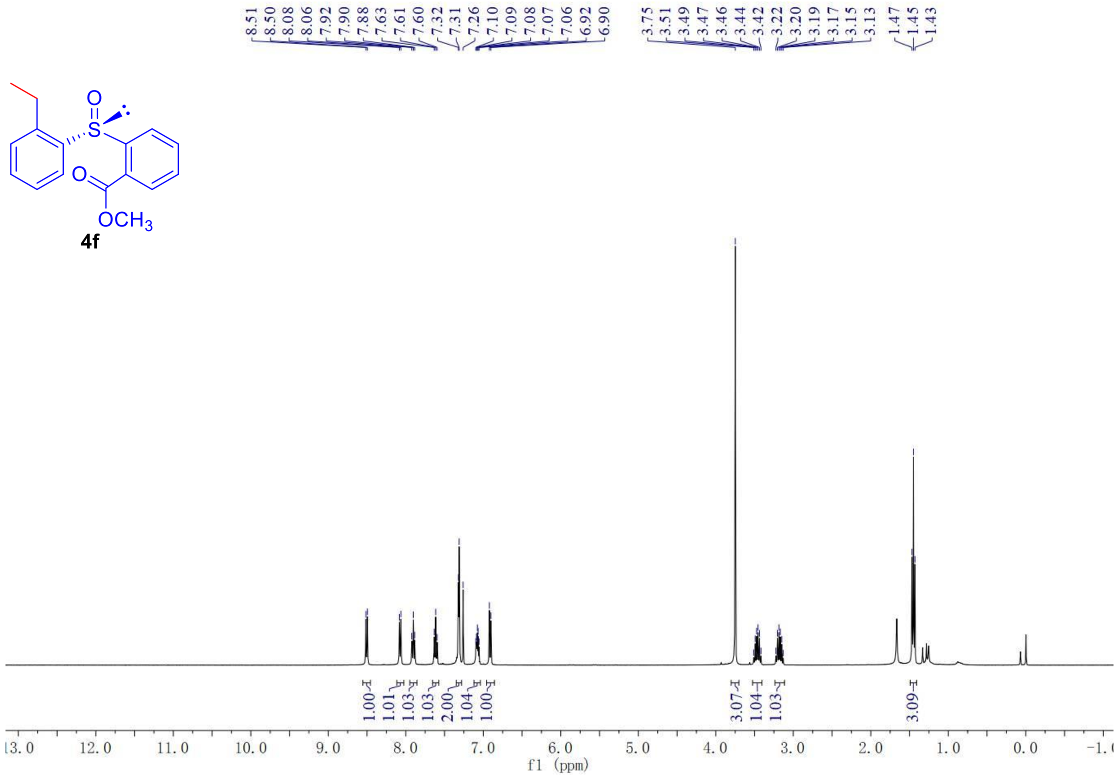

Supplementary Figure 119 ^{1}H NMR (400 MHz, $CDCl_3$) of **4f**

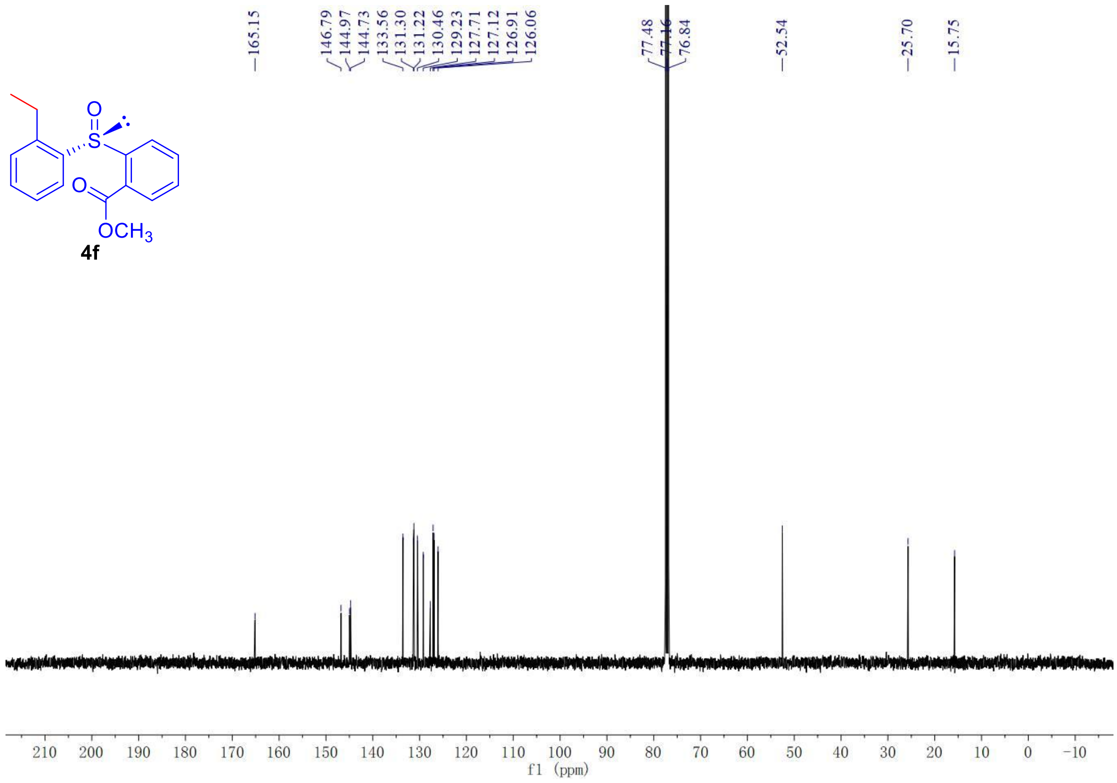

Supplementary Figure 120 ^{13}C NMR (101 MHz, $CDCl_3$) of **4f**

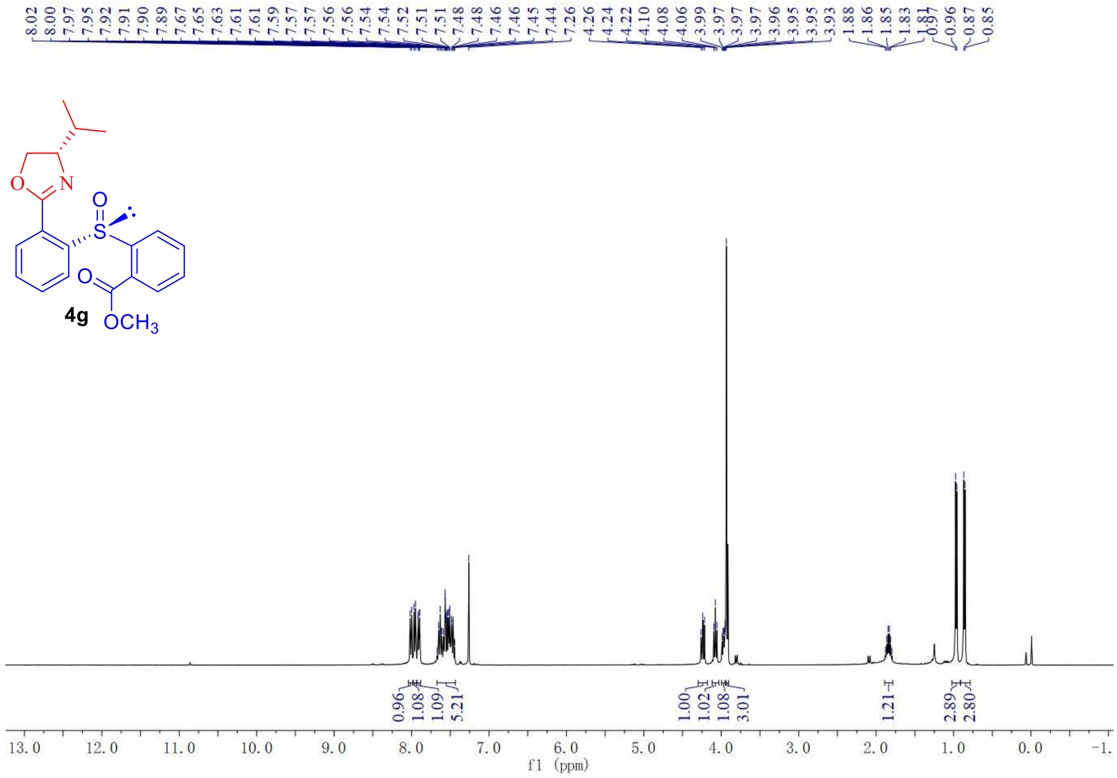

Supplementary Figure 121 ^{1}H NMR (400 MHz, $CDCl_3$) of **4g**

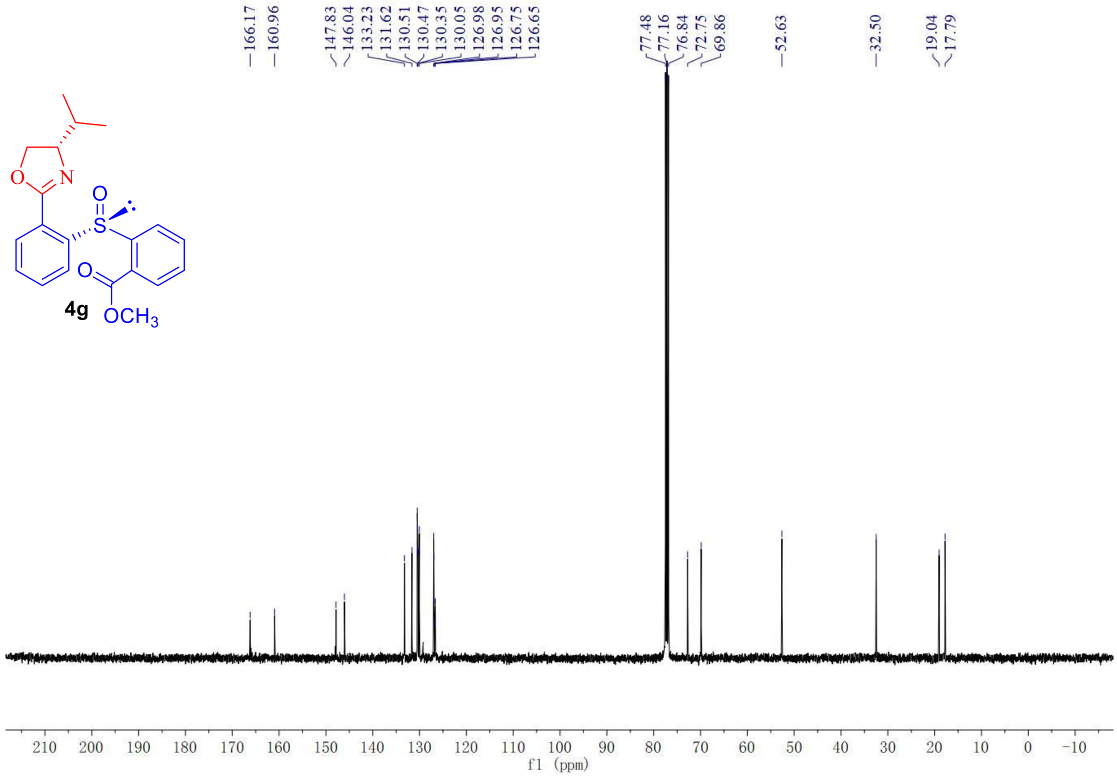

Supplementary Figure 122 ^{13}C NMR (101 MHz, $CDCl_3$) of **4g**

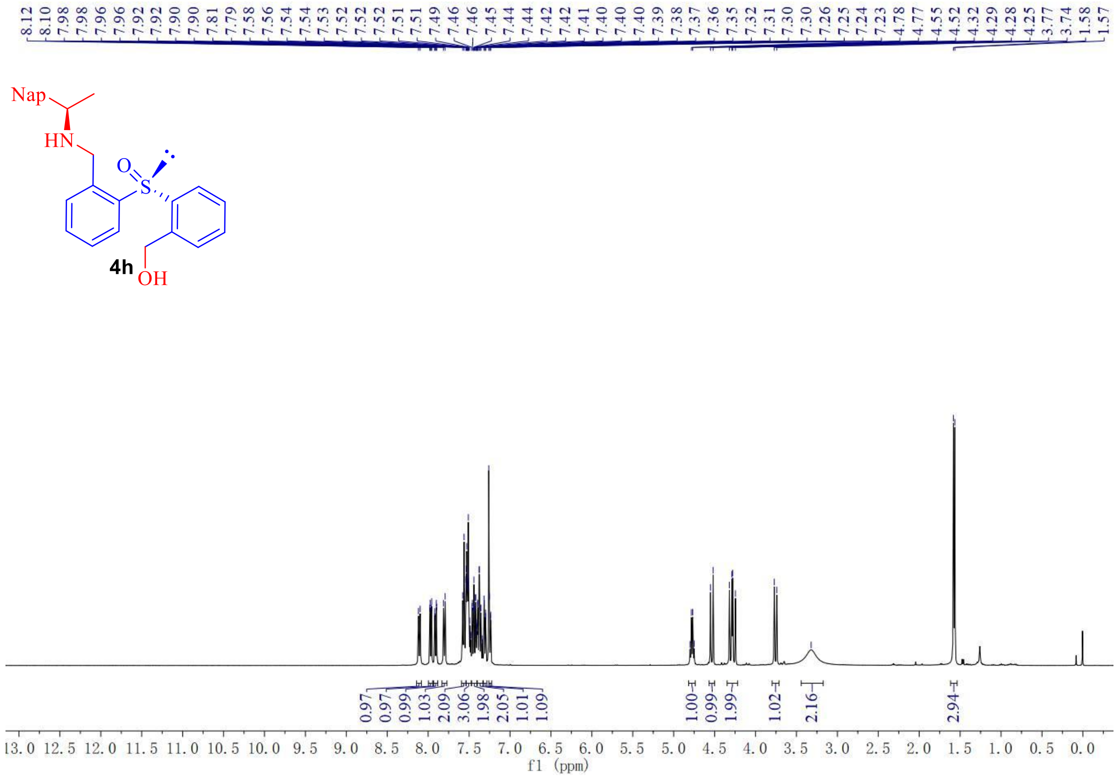

Supplementary Figure 123 ^{1}H NMR (400 MHz, $CDCl_3$) of **4h**

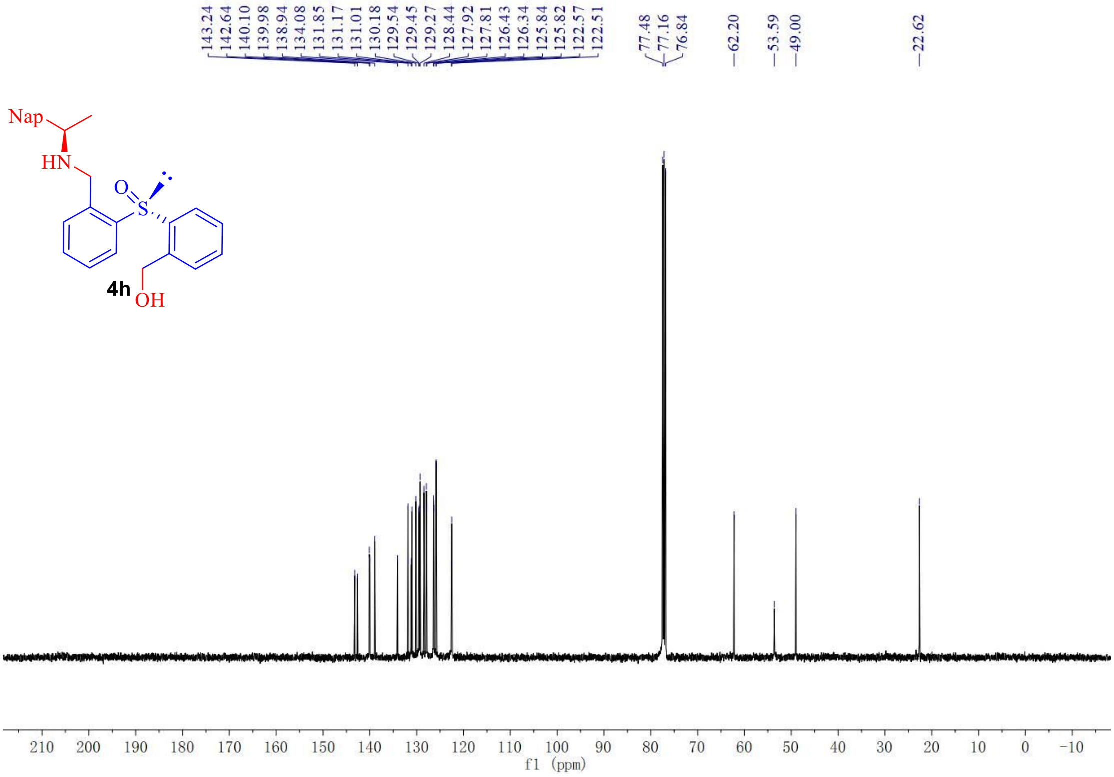

Supplementary Figure 124 ^{13}C NMR (101 MHz, $CDCl_3$) of **4h**

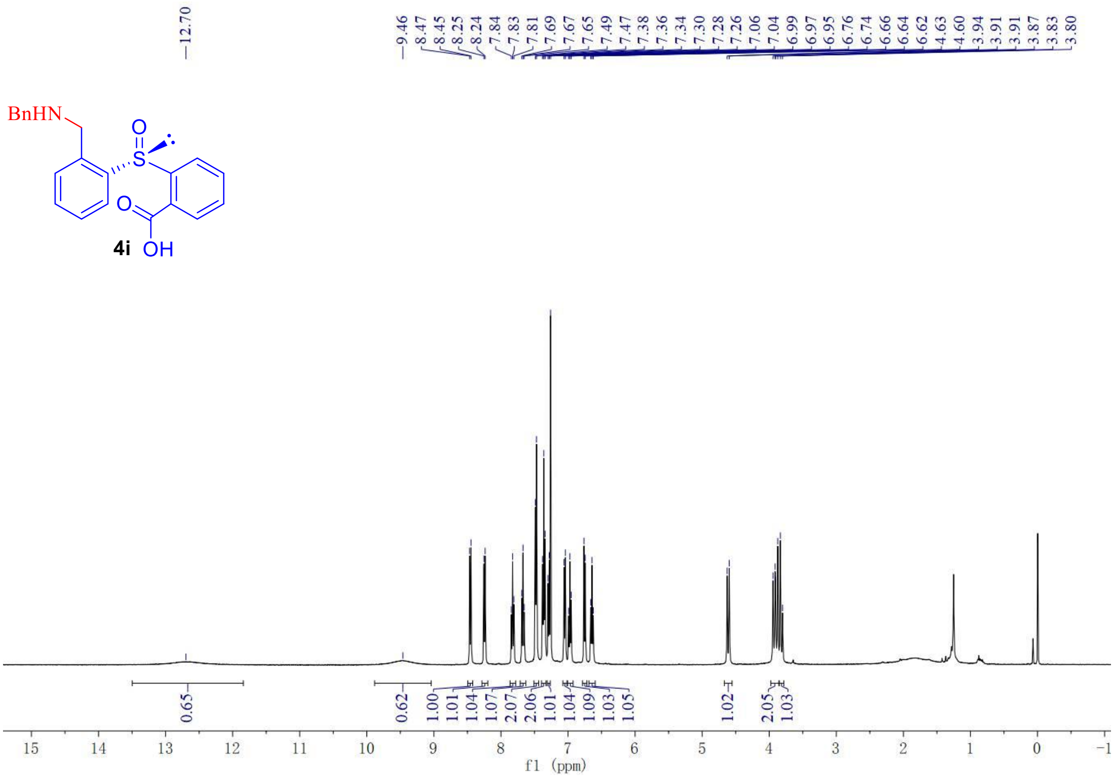

Supplementary Figure 125 ^{1}H NMR (400 MHz, $CDCl_3$) of **4i**

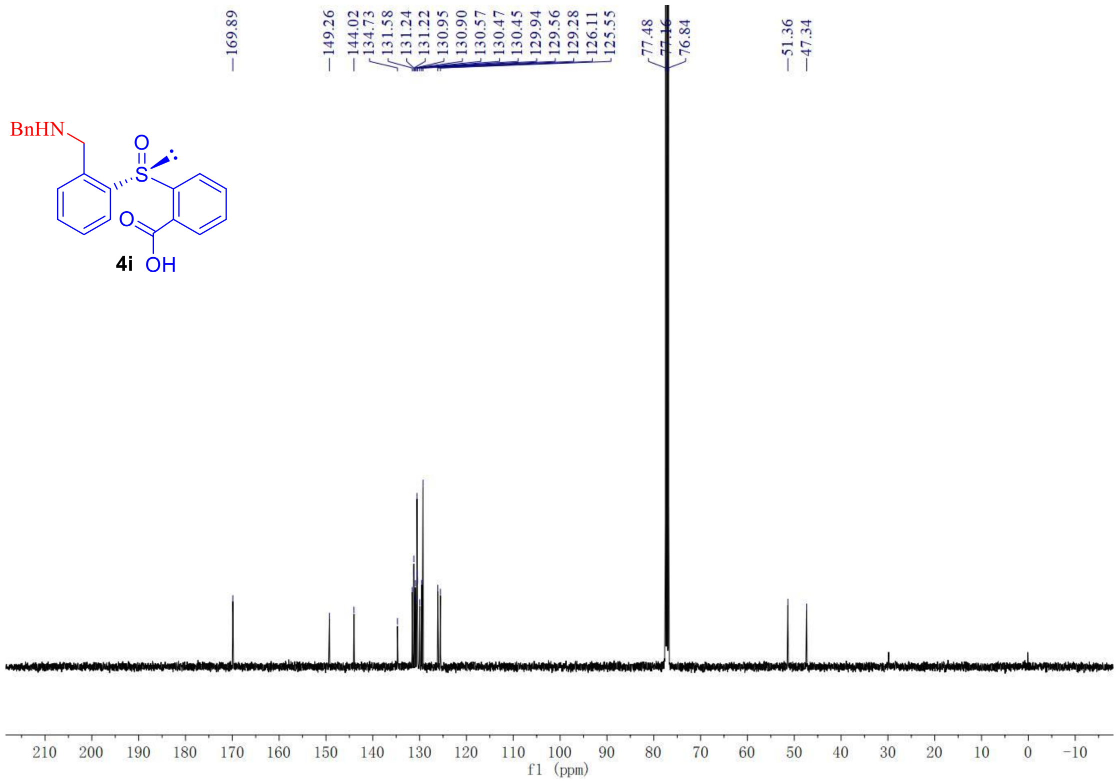

Supplementary Figure 126 ^{13}C NMR (101 MHz, $CDCl_3$) of **4i**

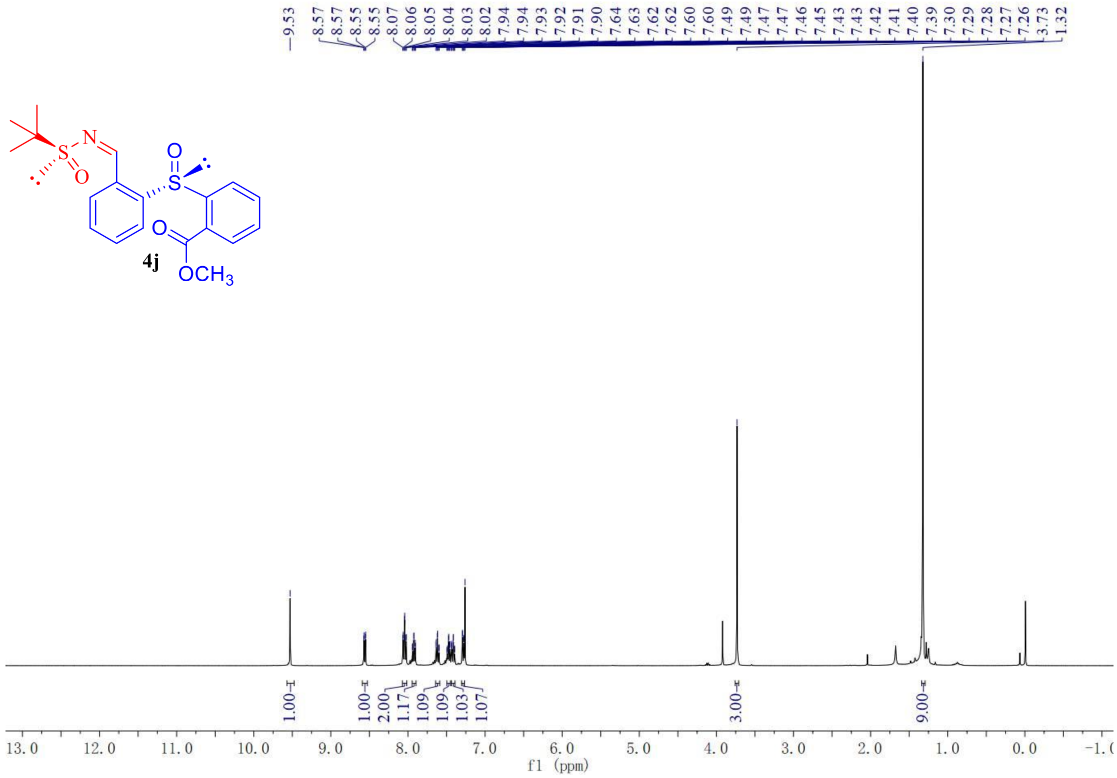

Supplementary Figure 127 ^{1}H NMR (400 MHz, $CDCl_3$) of **4j**

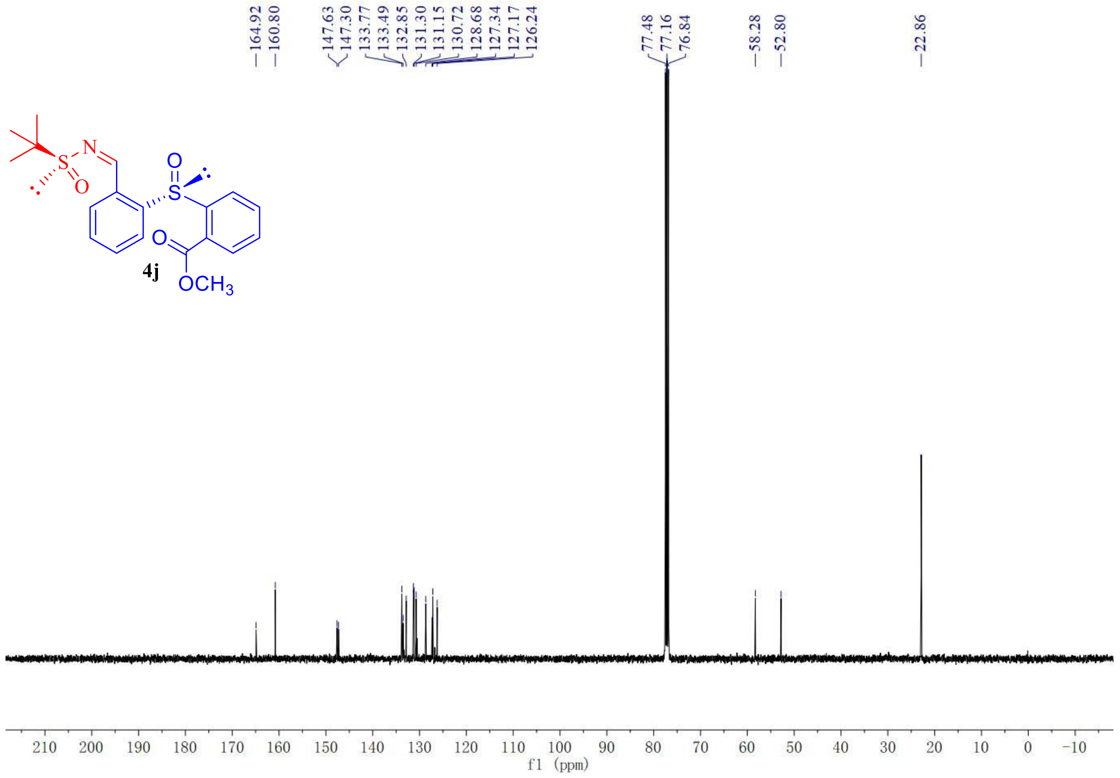

Supplementary Figure 128 ^{13}C NMR (101 MHz, $CDCl_3$) of **4j**

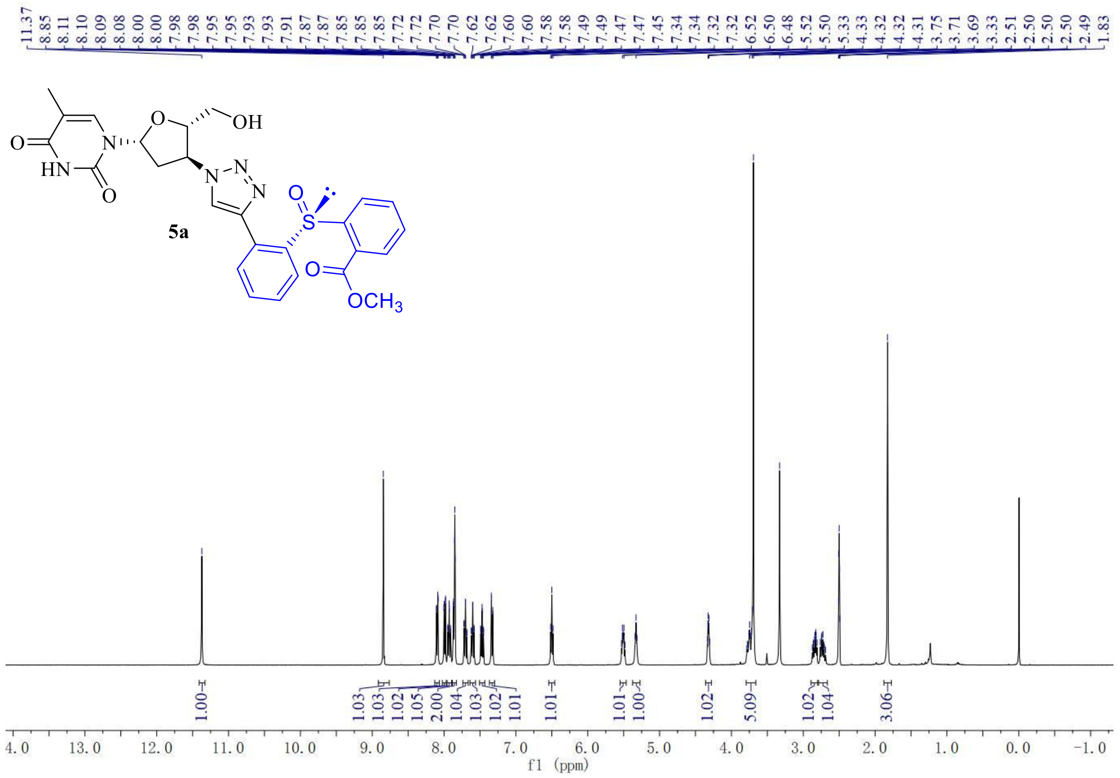

Supplementary Figure 129 ^{1}H NMR (400 MHz, DMSO-d_6) of **5a**

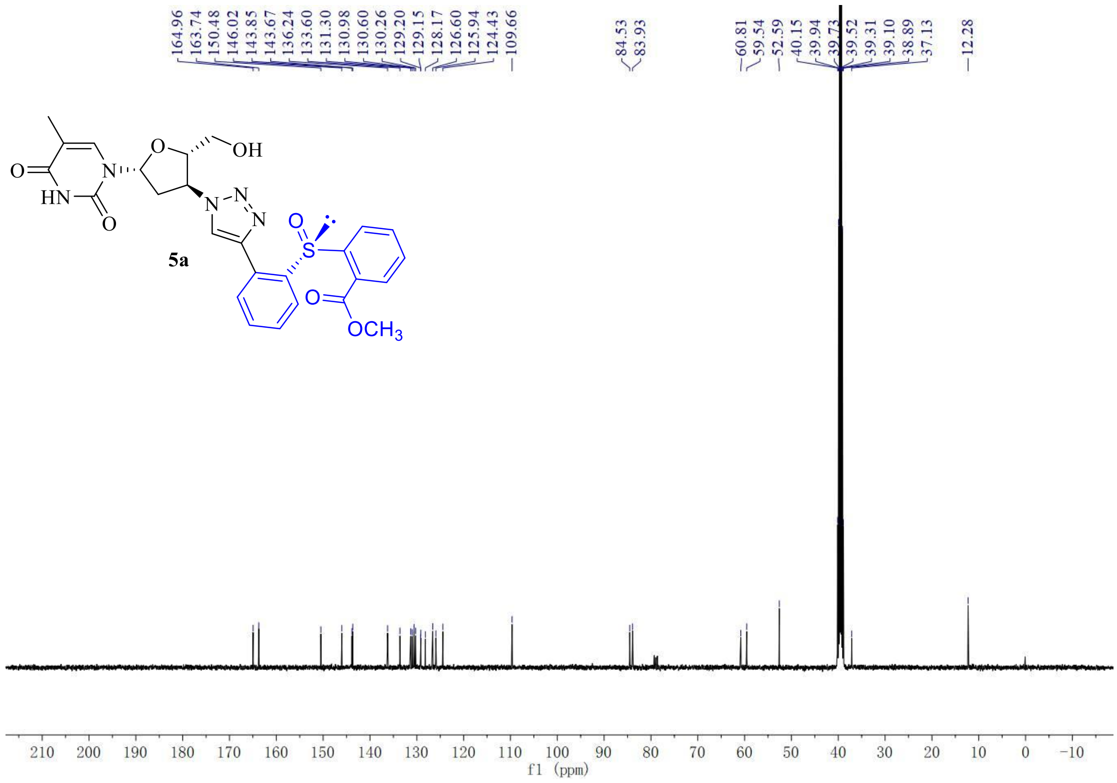

Supplementary Figure 130 ^{13}C NMR (101 MHz, DMSO-d_6) of **5a**

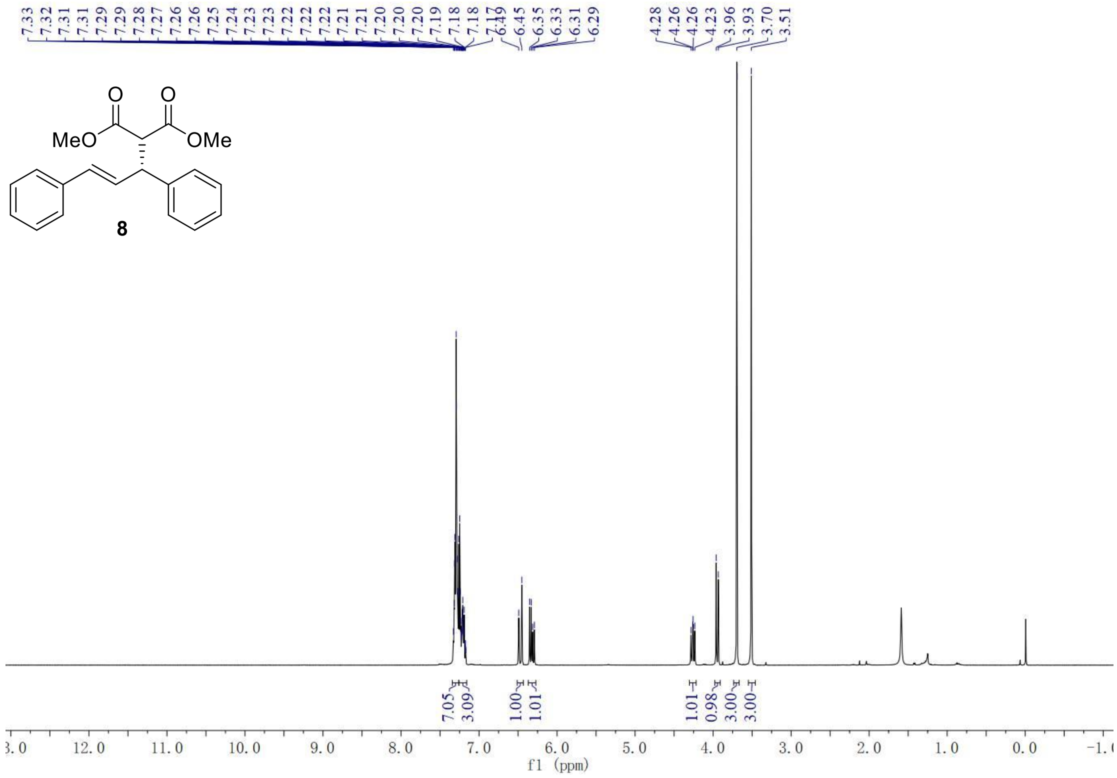

Supplementary Figure 131 ^{1}H NMR (400 MHz, $CDCl_3$) of **8**

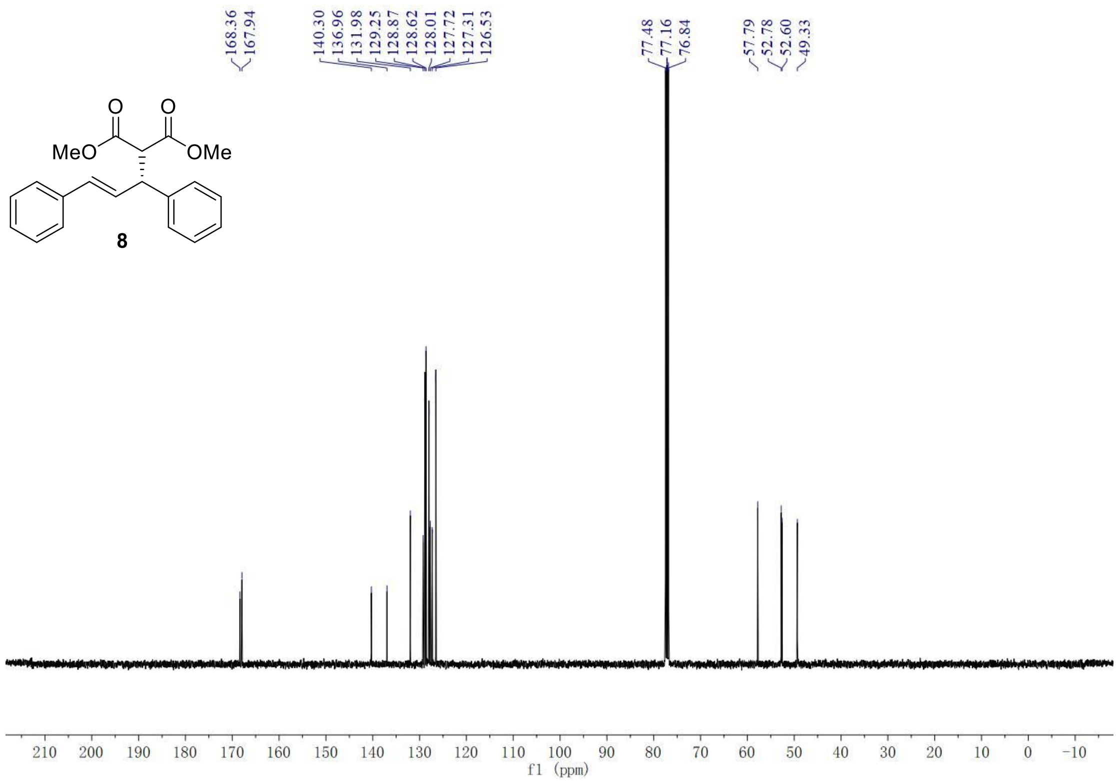

Supplementary Figure 132 ^{13}C NMR (101 MHz, $CDCl_3$) of **8**

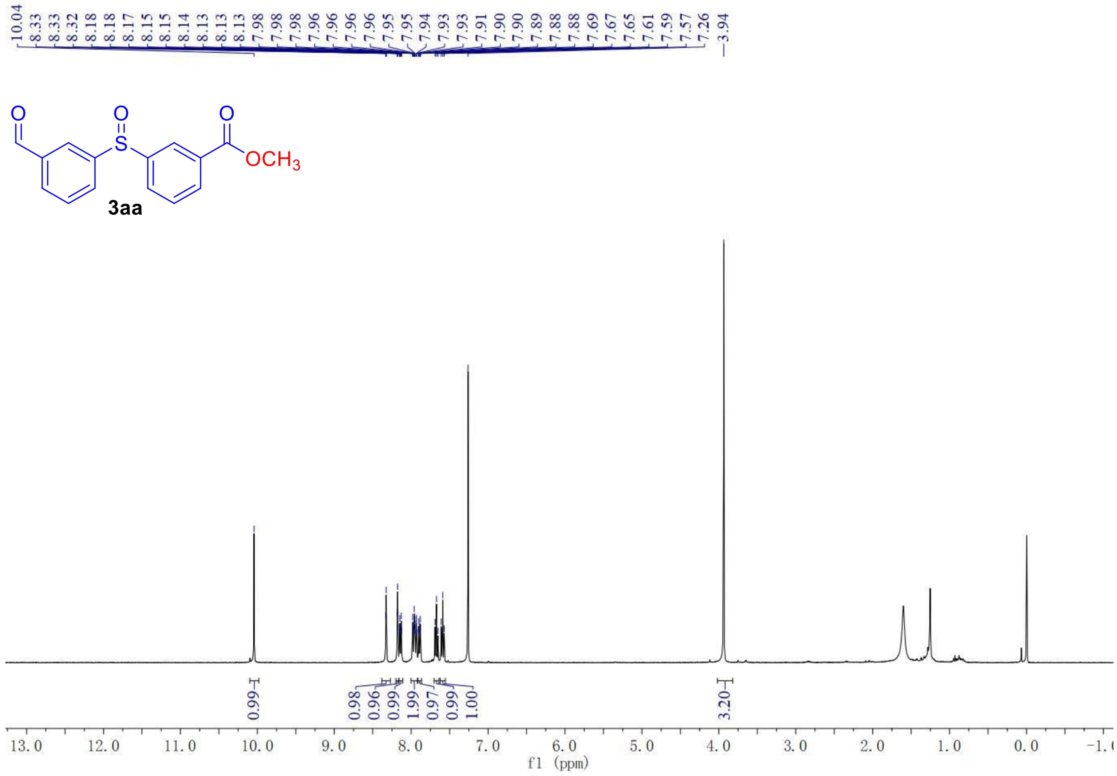

Supplementary Figure 133 ^{1}H NMR (400 MHz, $CDCl_3$) of **3aa**

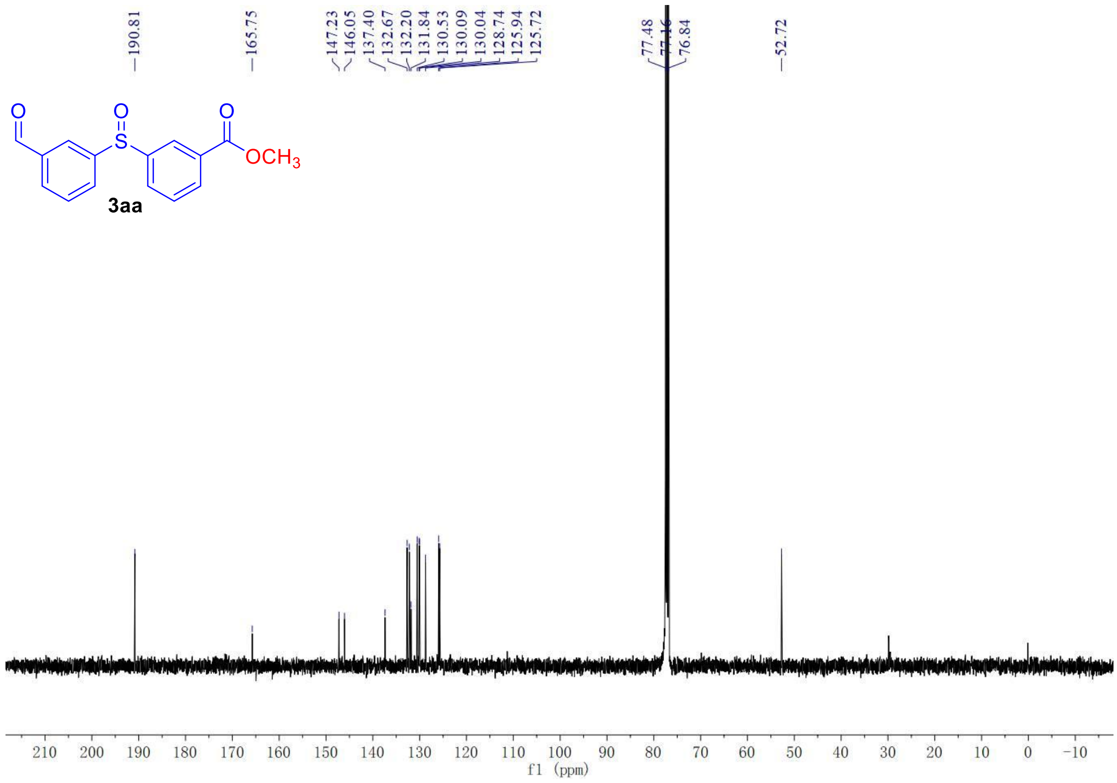

Supplementary Figure 134 ^{13}C NMR (101 MHz, $CDCl_3$) of **3aa**

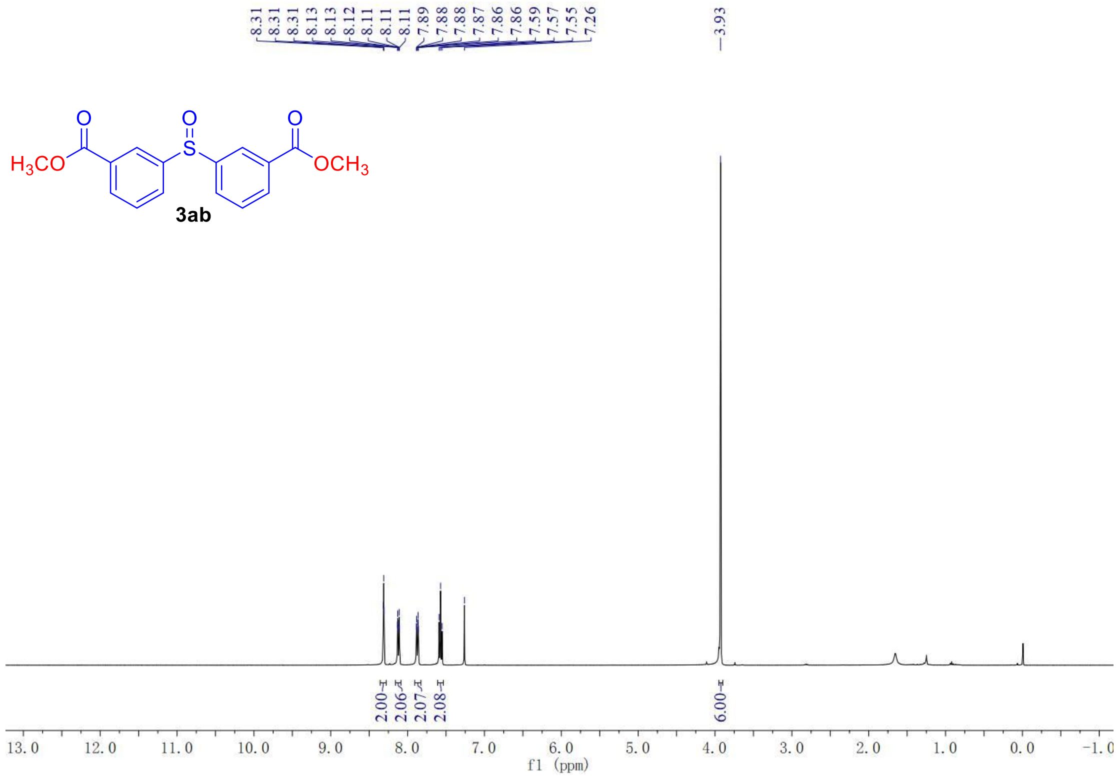

Supplementary Figure 135 ^{1}H NMR (400 MHz, $CDCl_3$) of **3ab**

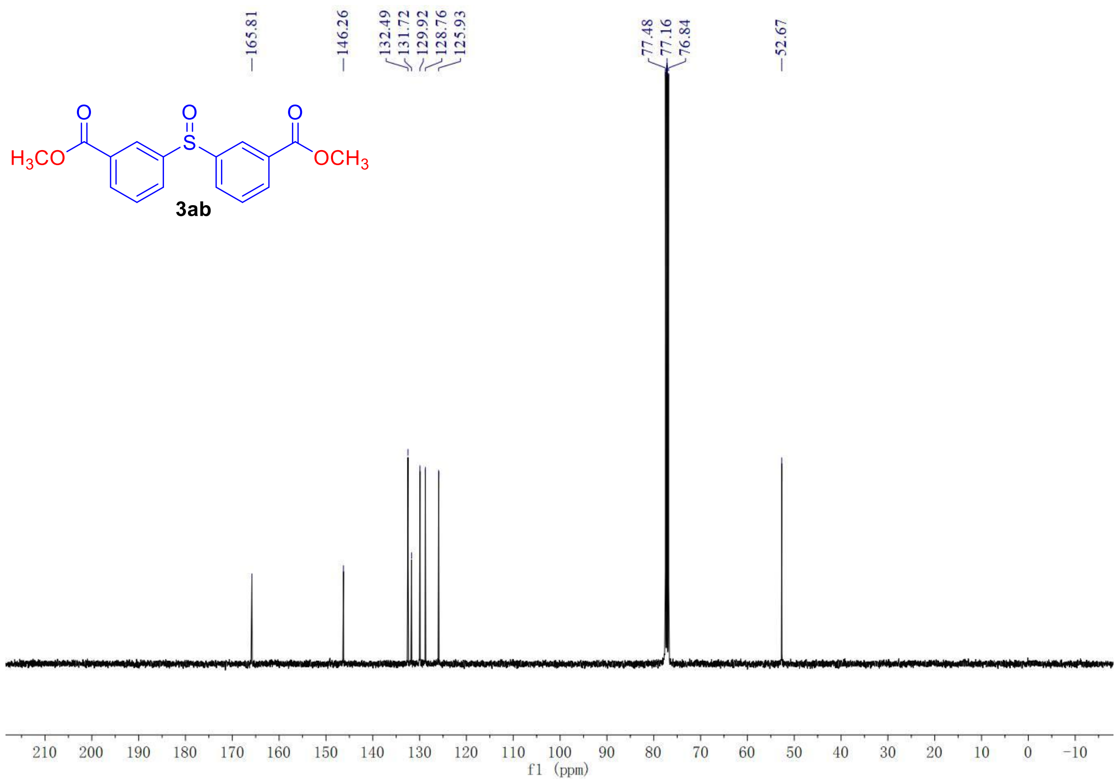

Supplementary Figure 136 ^{13}C NMR (101 MHz, $CDCl_3$) of **3ab**

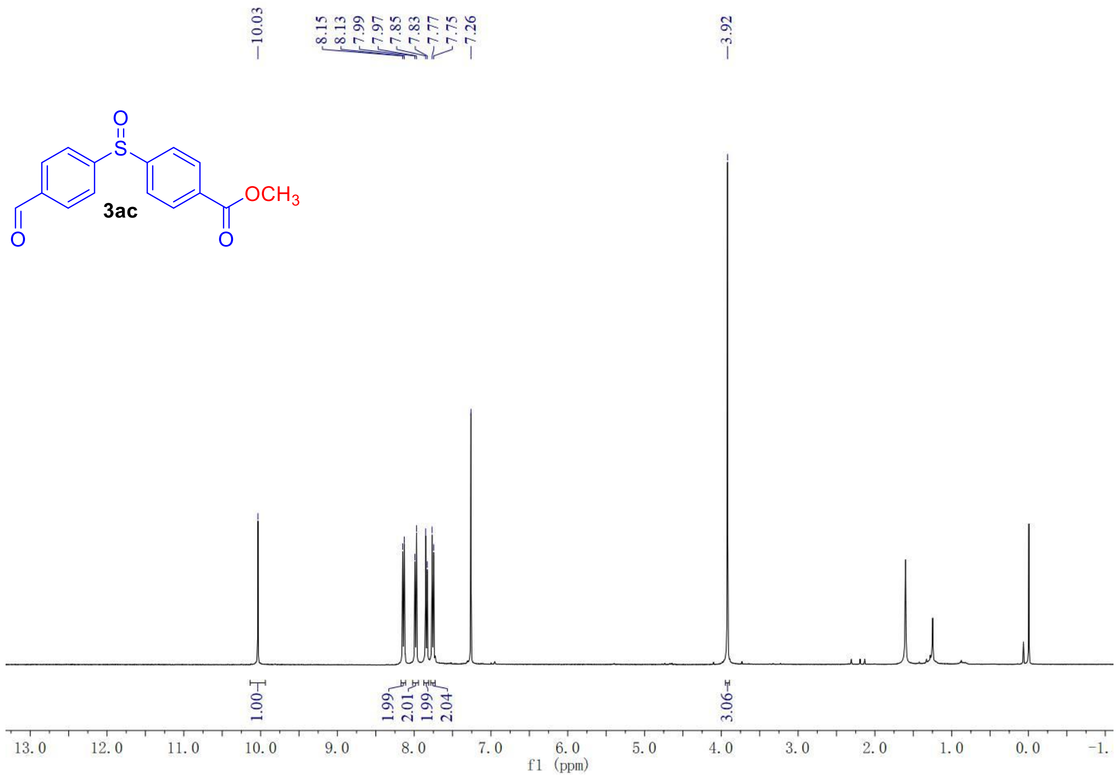

Supplementary Figure 137 ^{1}H NMR (400 MHz, $CDCl_3$) of **3ac**

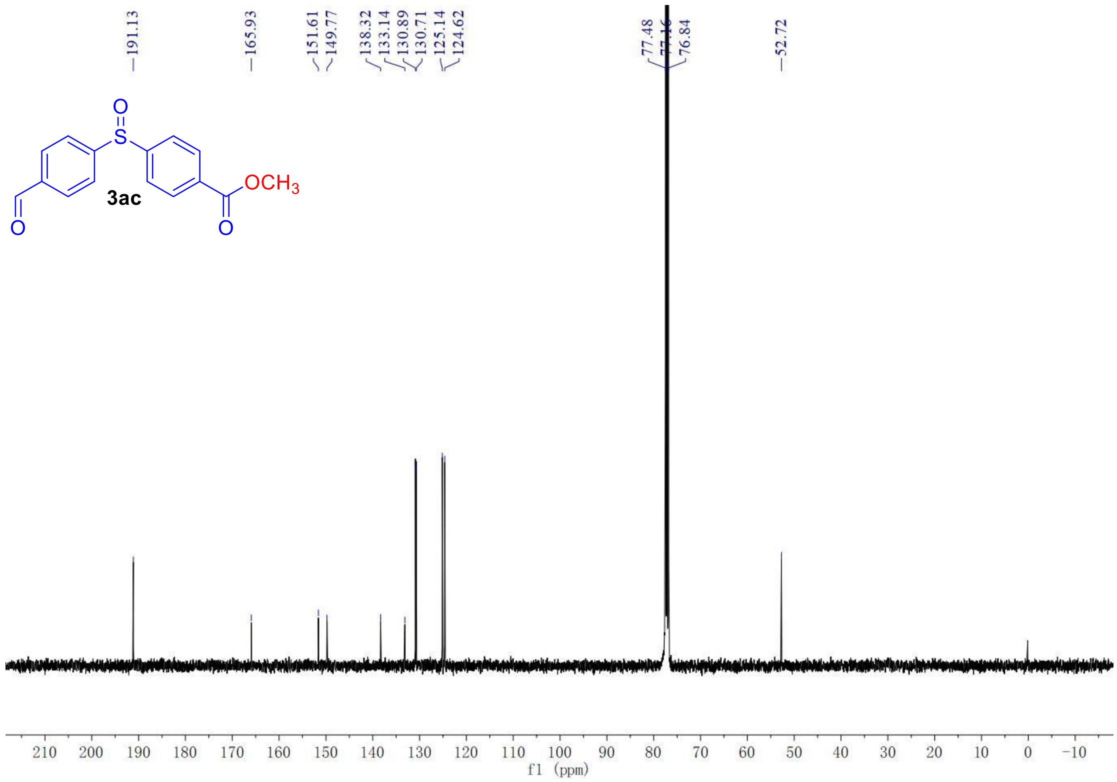

Supplementary Figure 138 ^{13}C NMR (101 MHz, $CDCl_3$) of **3ac**

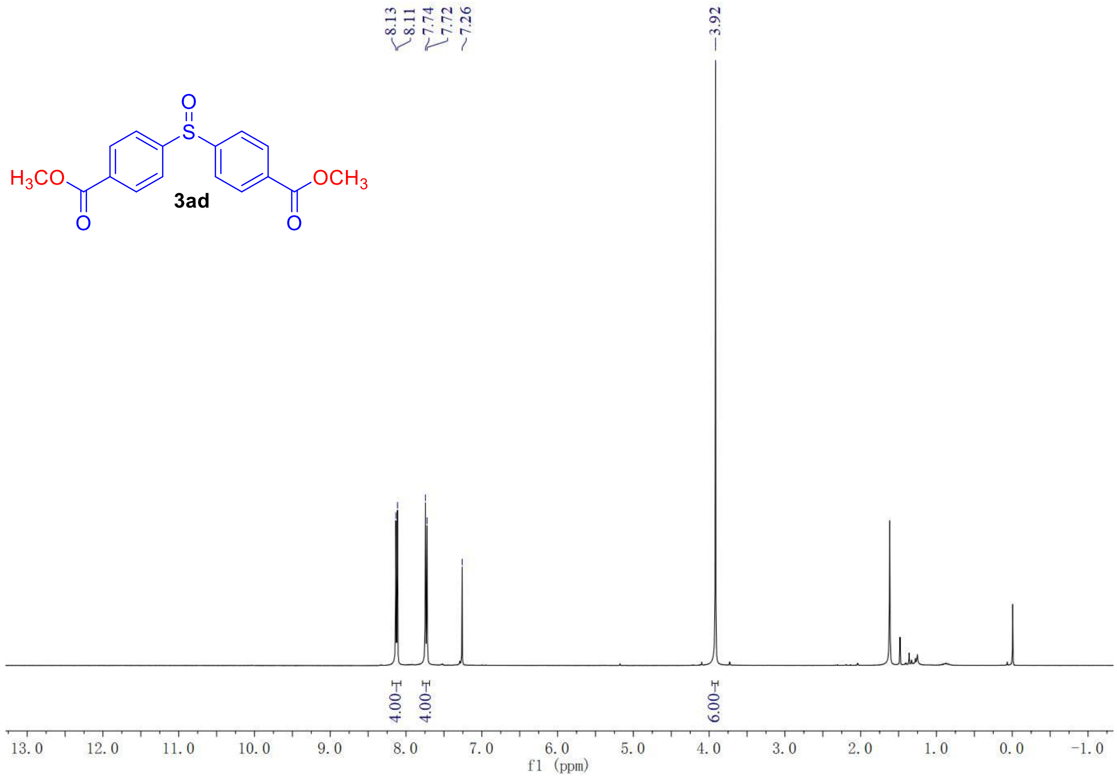

Supplementary Figure 139 ^{1}H NMR (400 MHz, $CDCl_3$) of **3ad**

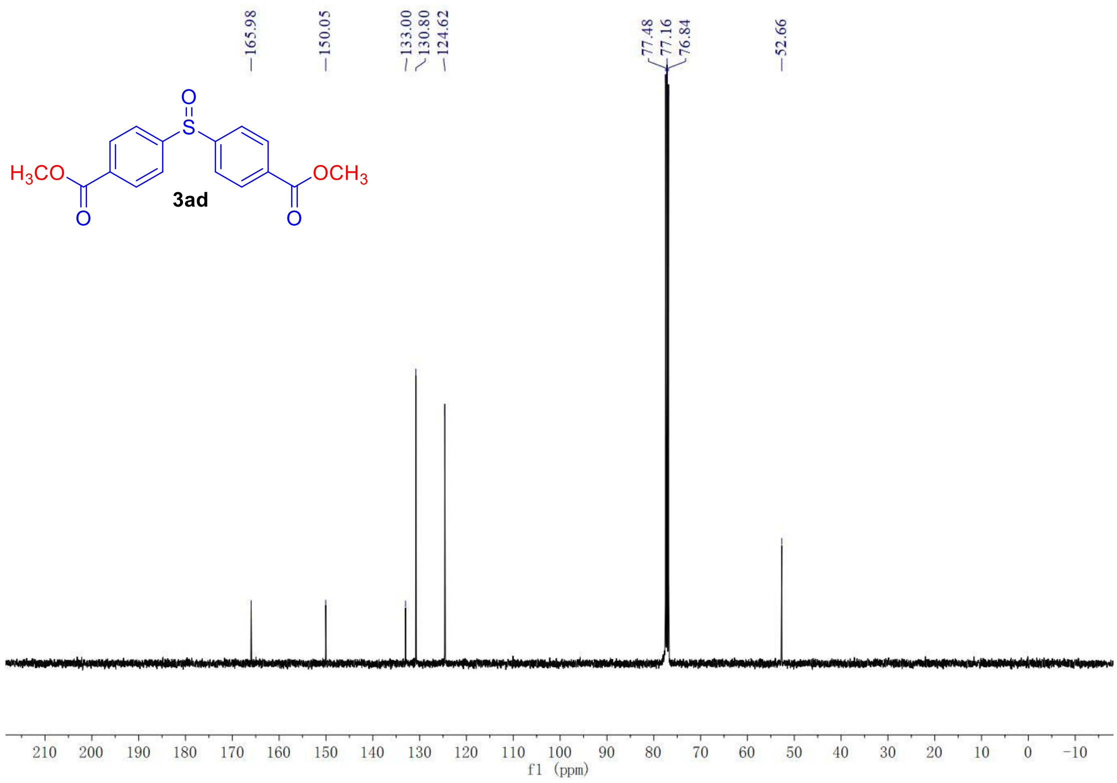

Supplementary Figure 140 ^{13}C NMR (101 MHz, $CDCl_3$) of **3ad**

HPLC and UPLC spectra

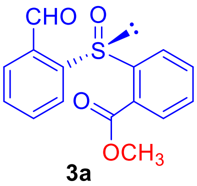

3a

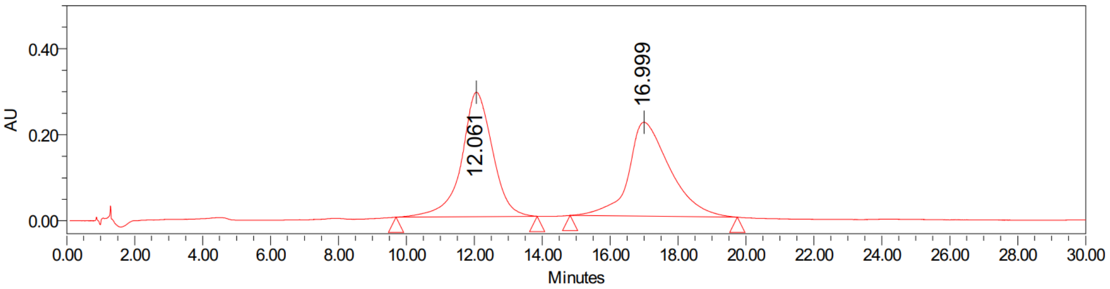

Peak Results

	RT	Height	Area	% Height	% Area
1	12.061	289626	18303403	56.98	49.63
2	16.999	218683	18573706	43.02	50.37

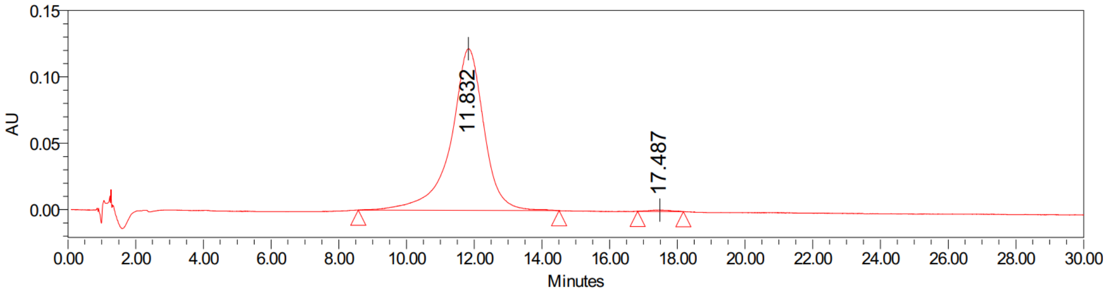

Peak Results

	RT	Height	Area	% Height	% Area
1	11.832	121742	8254363	99.12	99.44
2	17.487	1079	46664	0.88	0.56

Supplementary Figure 141 UPLC spectra of **3a**

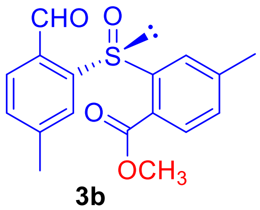

3b

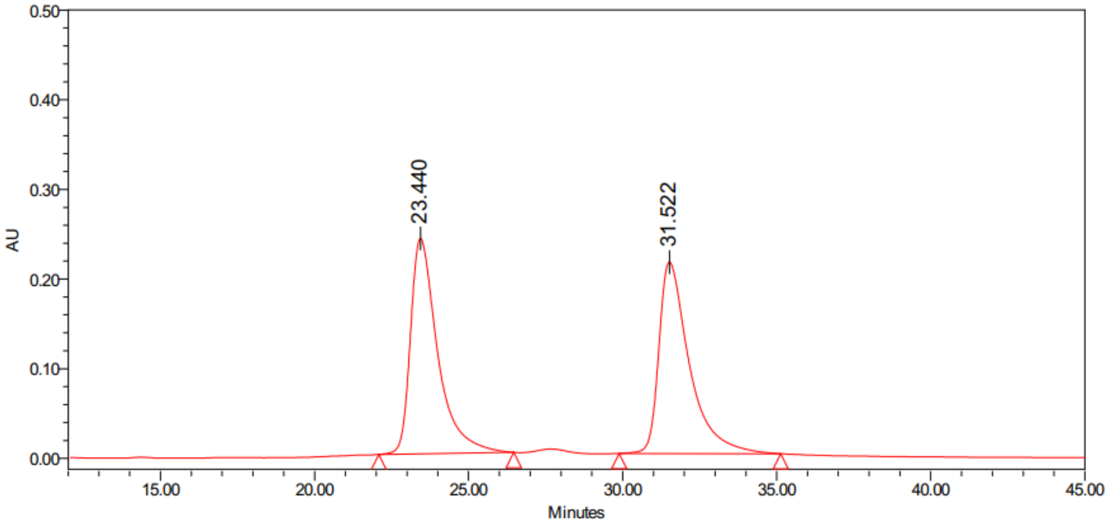

Peak Results

	RT	Area	Height	% Height	% Area
1	23.440	14746366	240264	52.95	50.09
2	31.522	14694398	213501	47.05	49.91

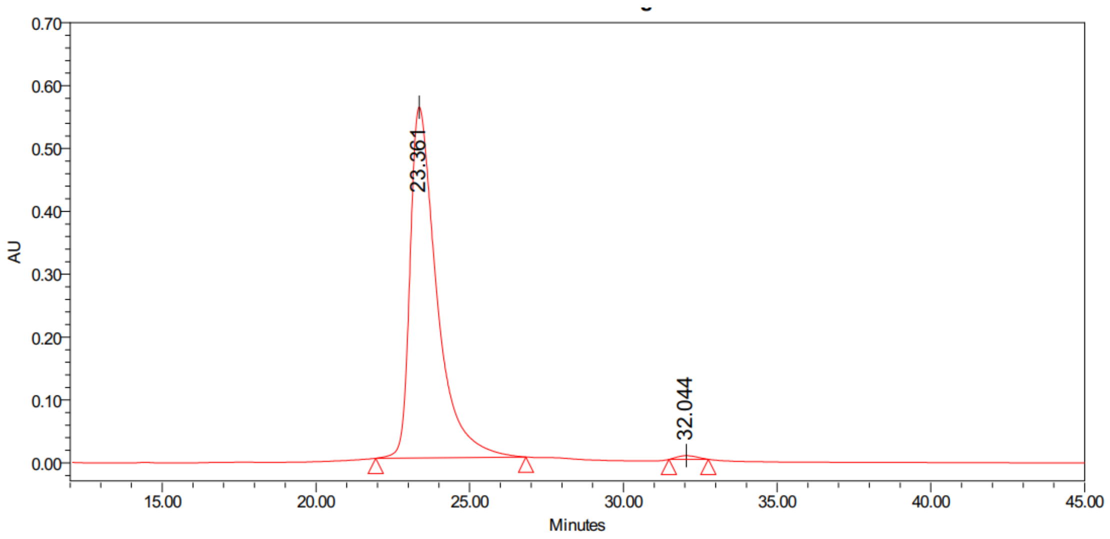

Peak Results

	RT	Area	Height	% Height	% Area
1	23.361	33727055	558104	98.96	99.24
2	32.044	259993	5841	1.04	0.76

Supplementary Figure 142 HPLC spectra of **3b**

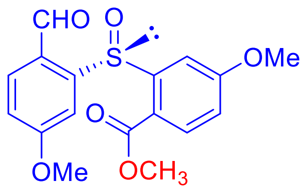

3c

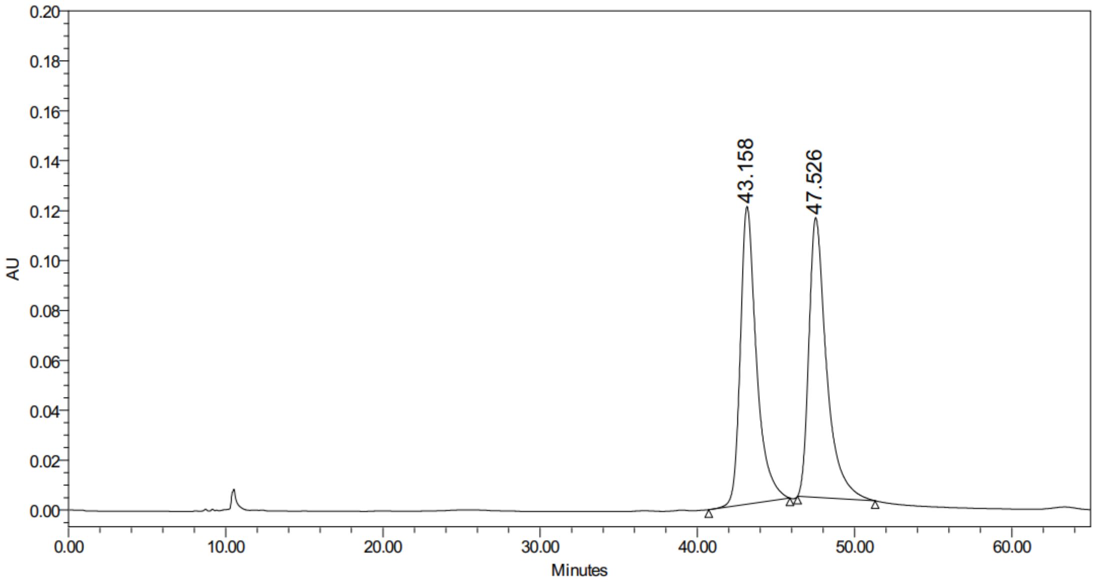

	RT	Height	Area	% Height	% Area
1	43.158	119277	8542909	51.57	49.44
2	47.526	112015	8735394	48.43	50.56

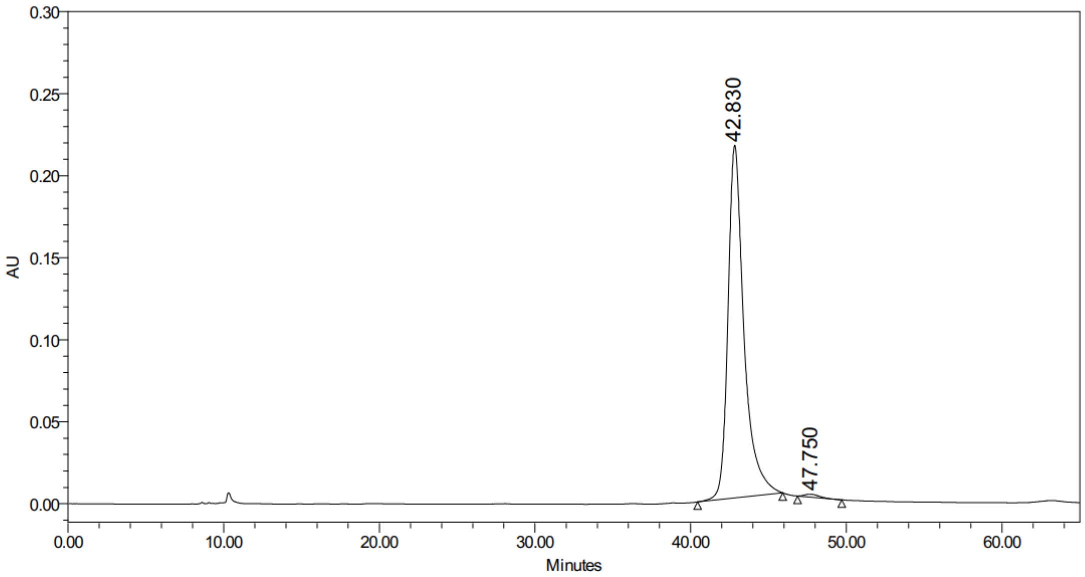

	RT	Height	Area	% Height	% Area
1	42.830	215096	15394168	99.16	99.25
2	47.750	1817	115914	0.84	0.75

Supplementary Figure 143 HPLC spectra of **3c**

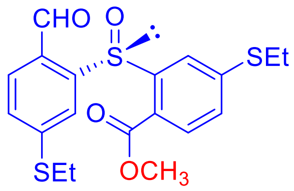

3d

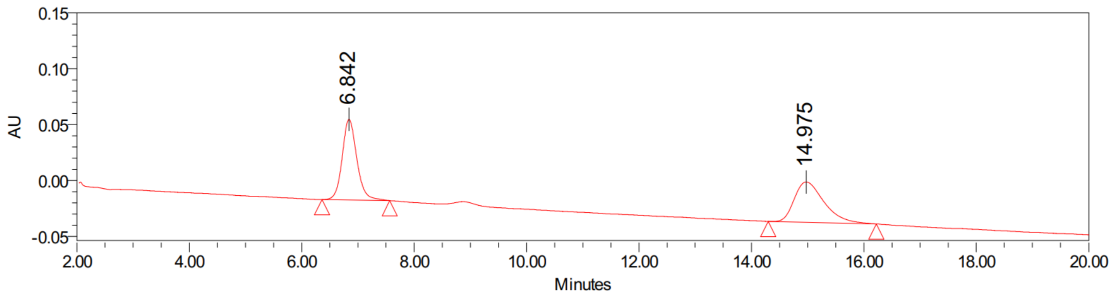

Peak Results

	RT	Height	Area	% Height	% Area
1	6.842	72185	1310623	66.66	49.67
2	14.975	36103	1327942	33.34	50.33

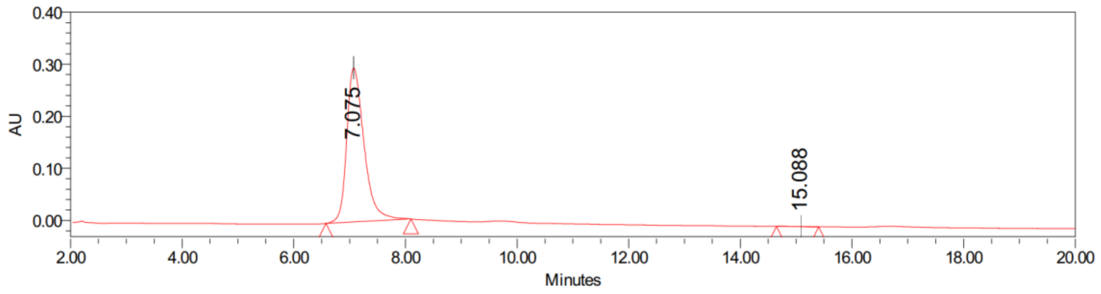

Peak Results

	RT	Height	Area	% Height	% Area
1	7.075	296105	6377178	99.97	99.98
2	15.088	91	1375	0.03	0.02

Supplementary Figure 144 UPLC spectra of **3d**

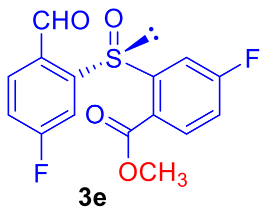

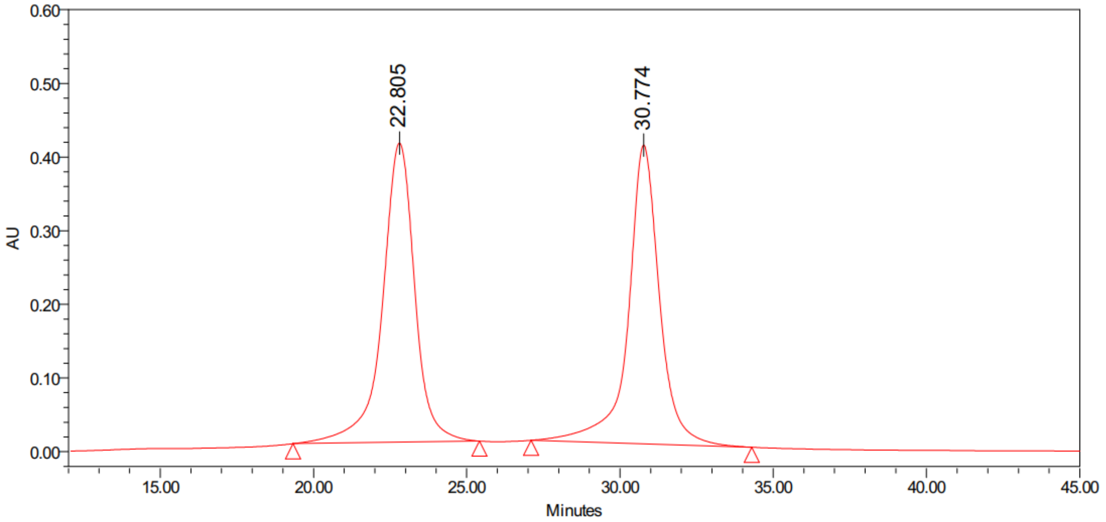

Peak Results

	RT	Area	Height	% Height	% Area
1	22.805	29919997	406380	50.04	50.77
2	30.774	29008849	405705	49.96	49.23

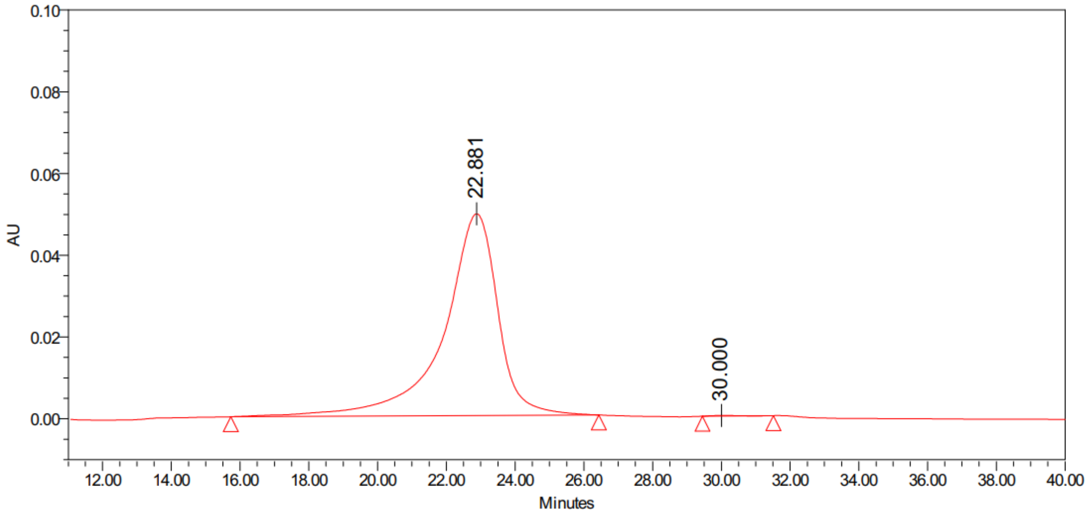

Peak Results

	RT	Area	Height	% Height	% Area
1	22.881	5441027	49406	99.71	99.85
2	30.000	8052	146	0.29	0.15

Supplementary Figure 145 HPLC spectra of **3e**

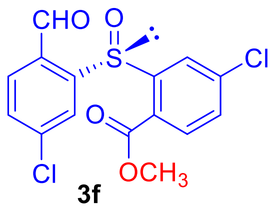

3f

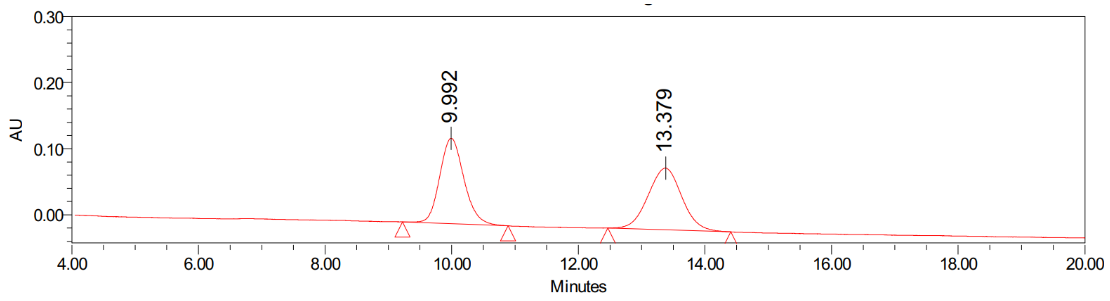

Peak Results

	RT	Height	Area	% Height	% Area
1	9.992	129437	3438648	58.06	49.81
2	13.379	93509	3464384	41.94	50.19

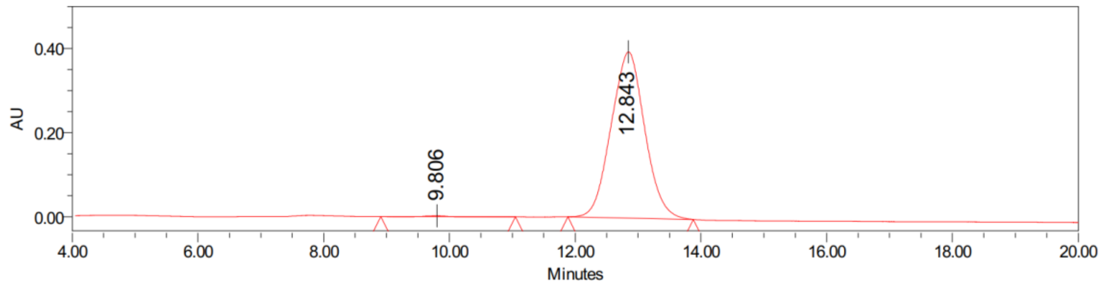

Peak Results

	RT	Height	Area	% Height	% Area
1	9.806	2028	61390	0.51	0.42
2	12.843	395157	14676809	99.49	99.58

Supplementary Figure 146 UPLC spectra of **3f**

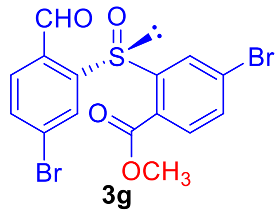

3g

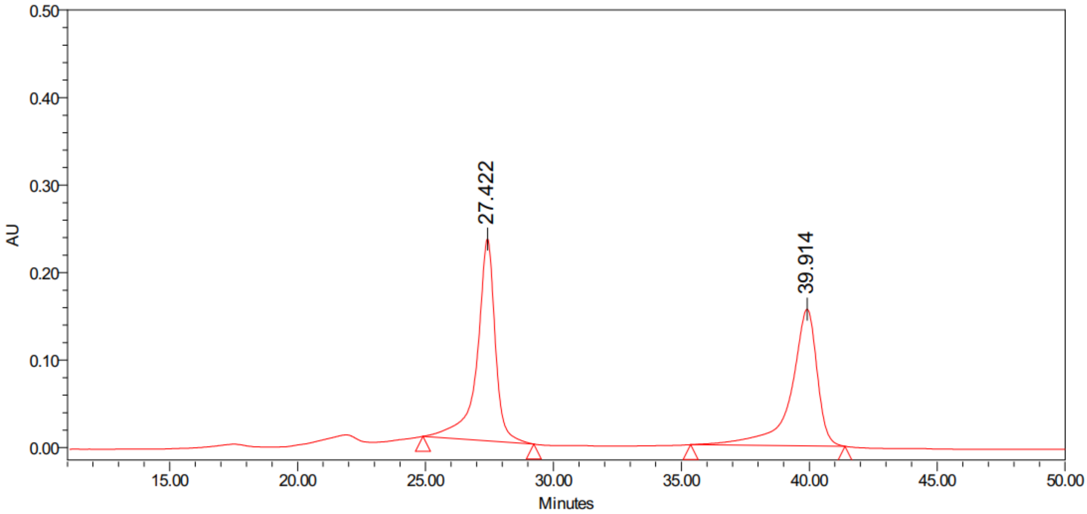

Peak Results

	RT	Area	Height	% Height	% Area
1	27.422	11462908	230635	59.62	50.90
2	39.914	11057467	156206	40.38	49.10

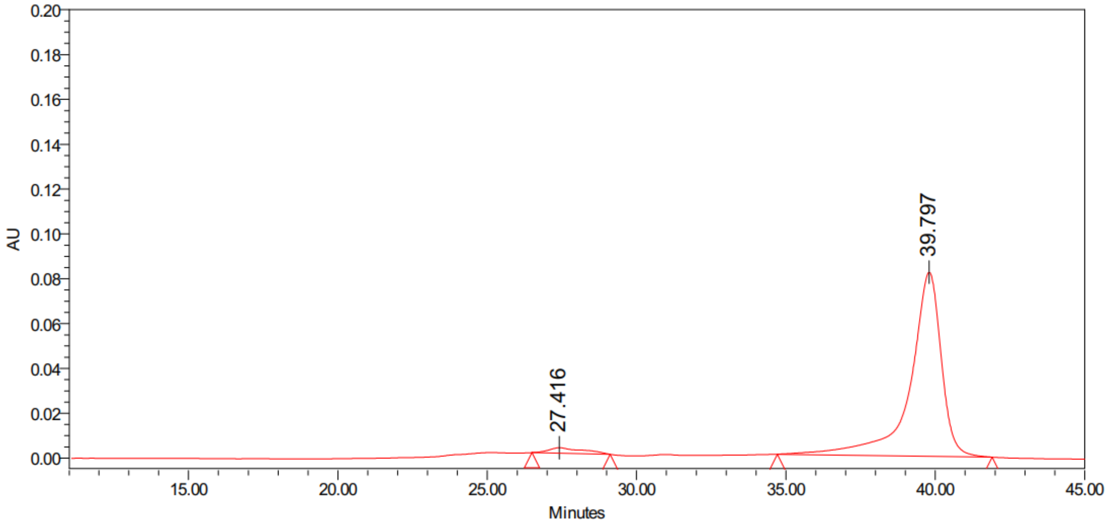

Peak Results

	RT	Area	Height	% Height	% Area
1	27.416	200598	2447	2.89	3.25
2	39.797	5979157	82181	97.11	96.75

Supplementary Figure 147 HPLC spectra of **3g**

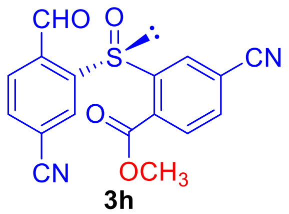

3h

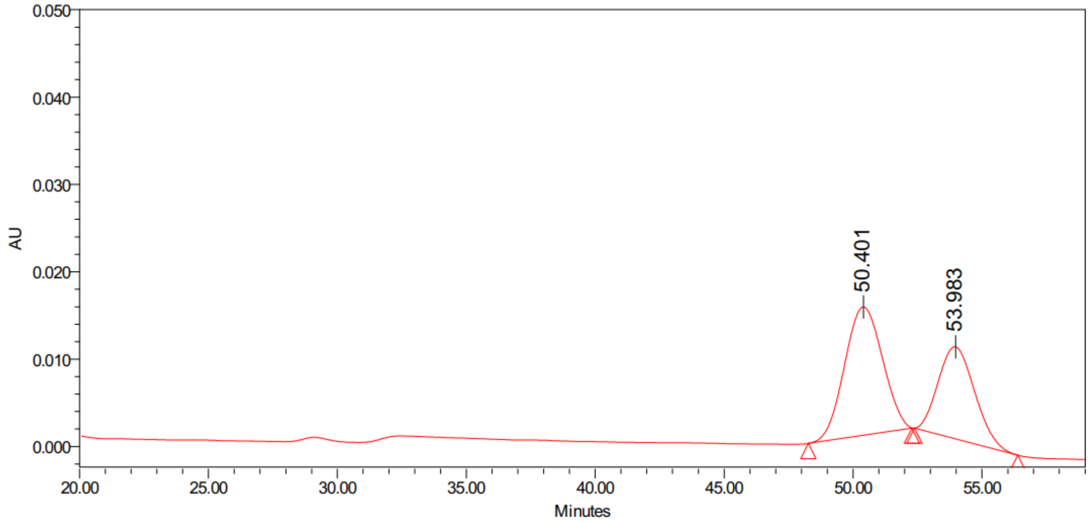

Peak Results

	RT	Area	Height	% Height	% Area
1	50.401	1497357	14683	58.18	59.01
2	53.983	1039947	10553	41.82	40.99

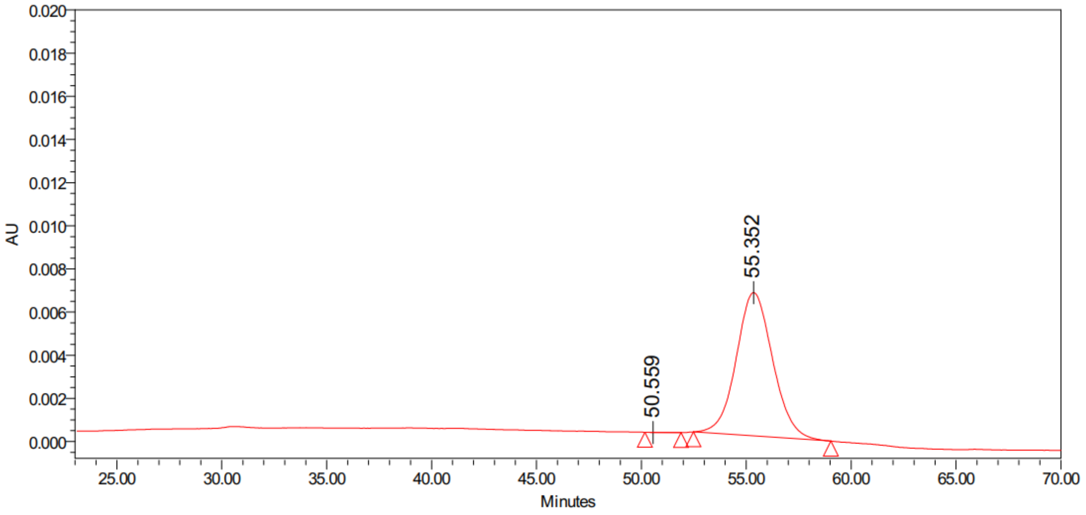

Peak Results

	RT	Area	Height	% Height	% Area
1	50.559	661	-13	0.19	0.08
2	55.352	803888	6648	99.81	99.92

Supplementary Figure 148 HPLC spectra of **3h**

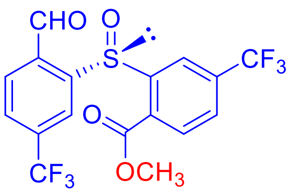

3i

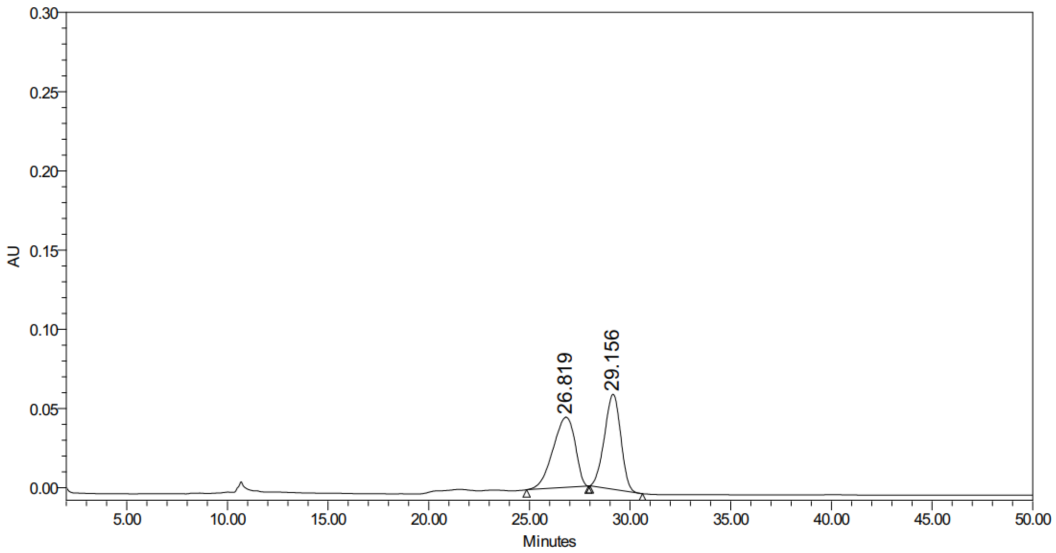

	RT	Height	Area	% Height	% Area
1	26.819	44332	3383994	42.51	50.05
2	29.156	59944	3377640	57.49	49.95

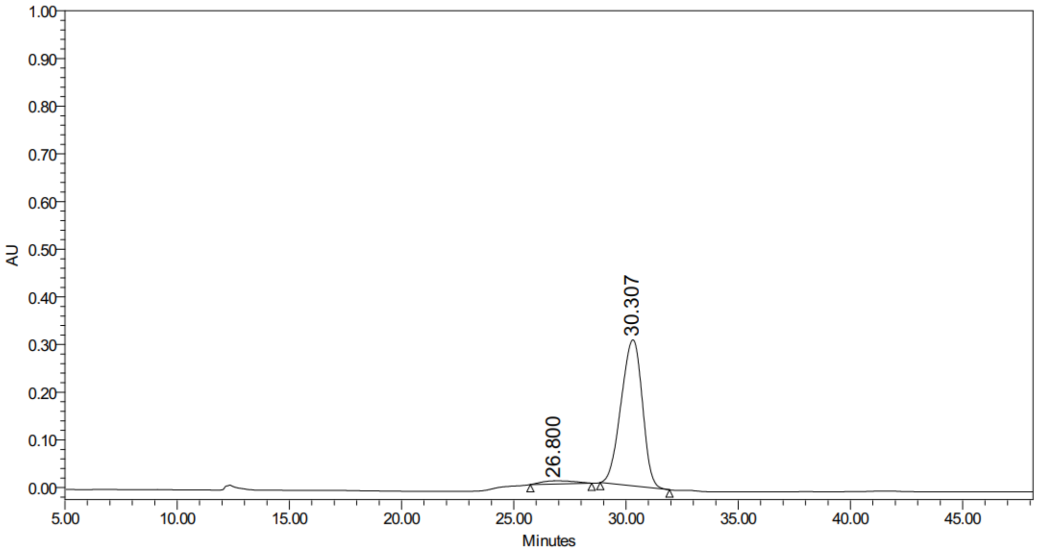

	RT	Height	Area	% Height	% Area
1	26.800	6608	662021	2.11	3.17
2	30.307	306613	20213598	97.89	96.83

Supplementary Figure 149 HPLC spectra of **3i**

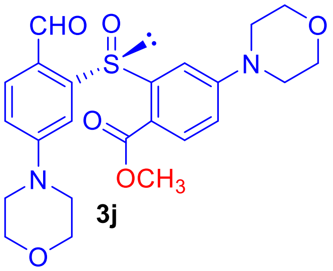

3j

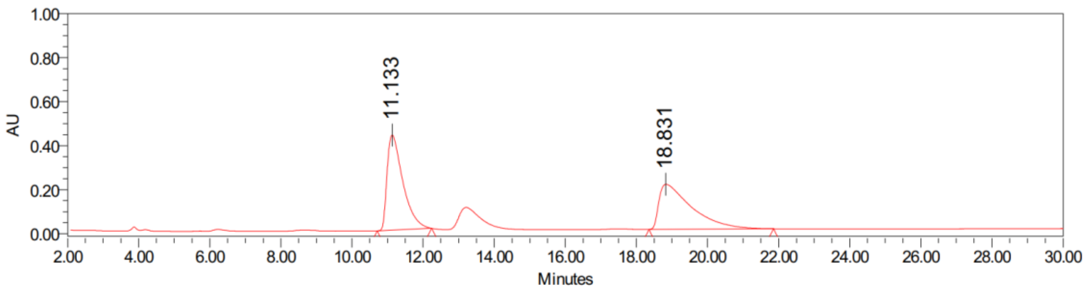

Peak Results

	RT	Height	% Height	Area (μV*sec)	% Area
1	11.133	432472	67.87	13377359	50.58
2	18.831	204709	32.13	13069500	49.42

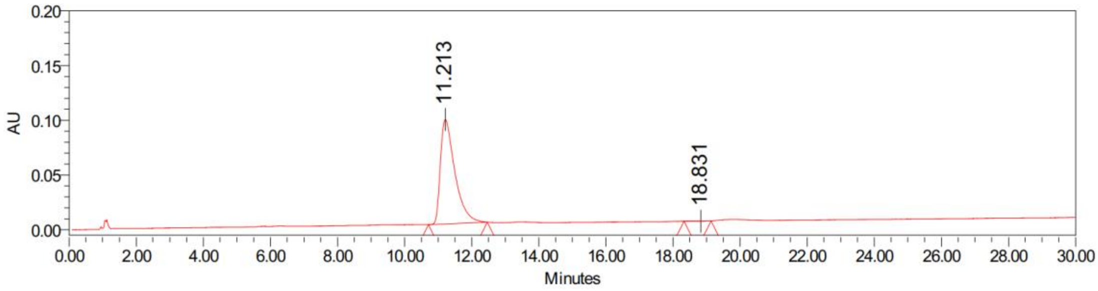

Peak Results

	RT	Height	% Height	Area (μV*sec)	% Area
1	11.213	95376	99.85	2870009	99.90
2	18.831	-139	0.15	2874	0.10

Supplementary Figure 150 UPLC spectra of **3j**

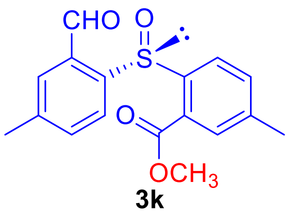

3k

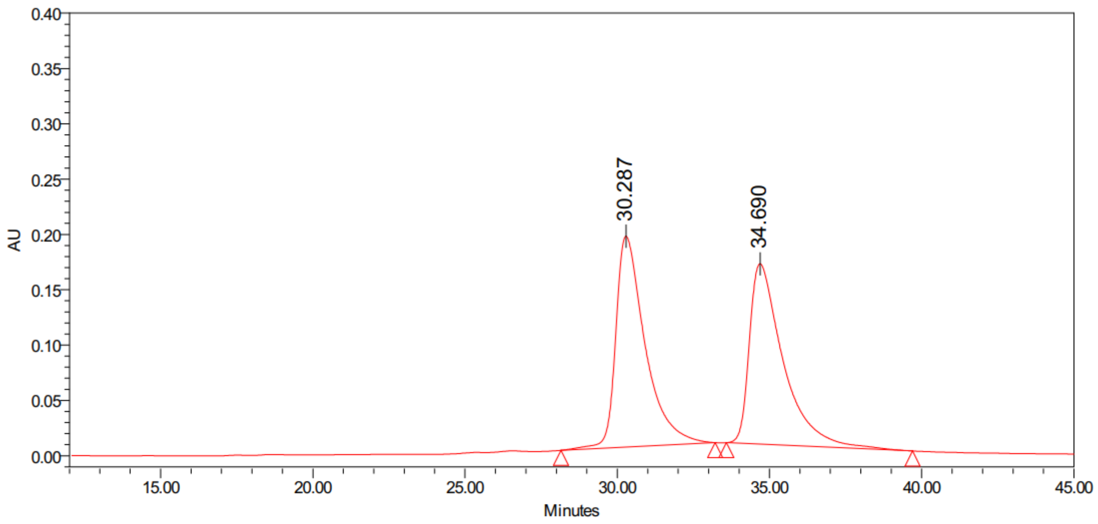

Peak Results

	RT	Area	Height	% Height	% Area
1	30.287	12814631	190662	53.90	50.41
2	34.690	12604768	163078	46.10	49.59

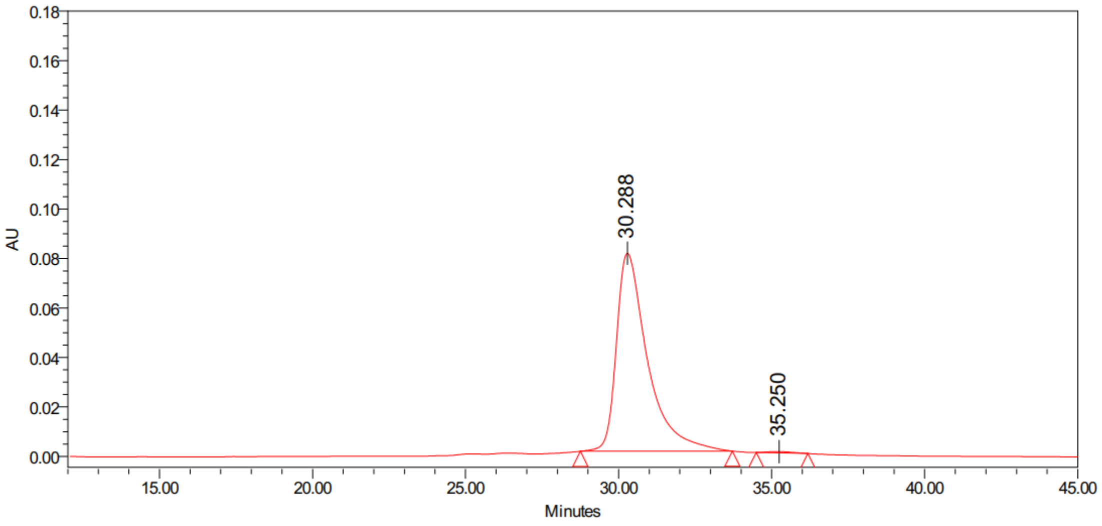

Peak Results

	RT	Area	Height	% Height	% Area
1	30.288	5654852	80037	99.31	99.45
2	35.250	31336	556	0.69	0.55

Supplementary Figure 151 HPLC spectra of **3k**

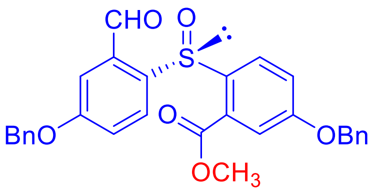

3l

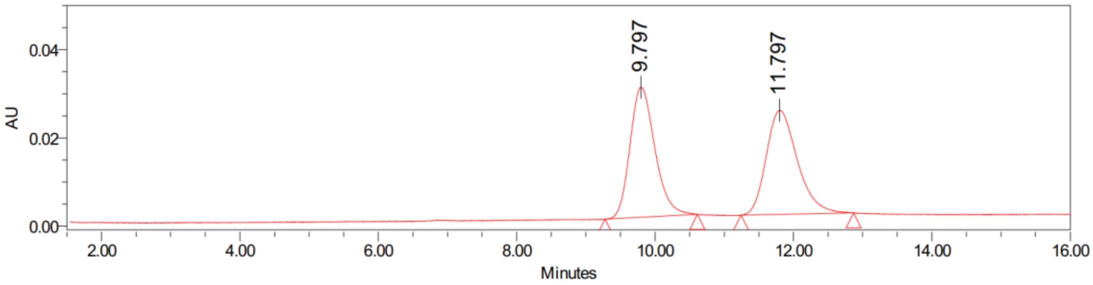

Peak Results

	RT	Height	Area	% Height	% Area
1	9.797	29518	738611	55.57	50.11
2	11.797	23602	735474	44.43	49.89

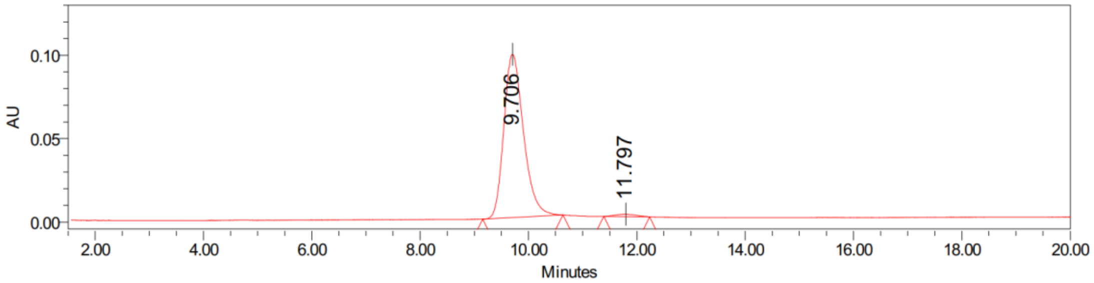

Peak Results

	RT	Height	Area	% Height	% Area
1	9.706	97973	2466383	98.56	98.51
2	11.797	1435	37215	1.44	1.49

Supplementary Figure 152 UPLC spectra of **3l**

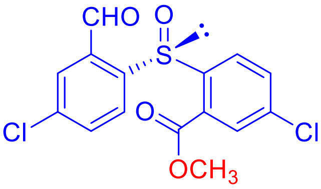

3m

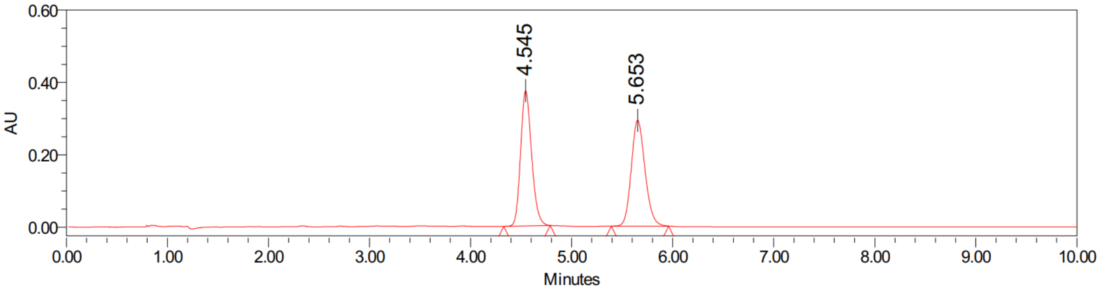

Peak Results

	RT	Height	Area	% Height	% Area
1	4.545	373859	2826983	56.09	50.60
2	5.653	292679	2759467	43.91	49.40

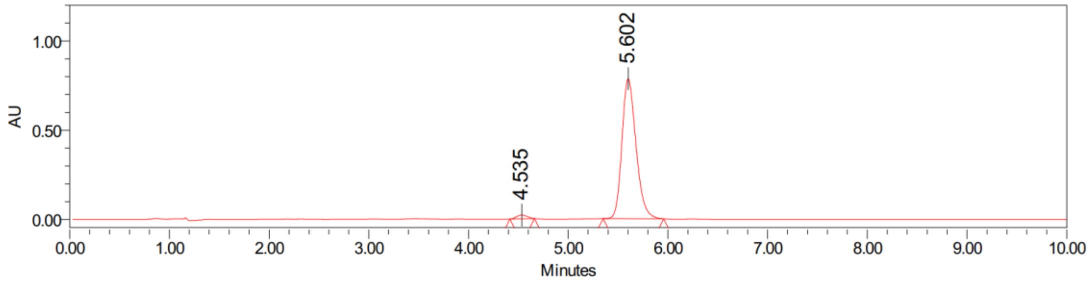

Peak Results

	RT	Height	Area	% Height	% Area
1	4.535	20779	154944	2.58	1.98
2	5.602	784135	7675681	97.42	98.02

Supplementary Figure 153 UPLC spectra of **3m**

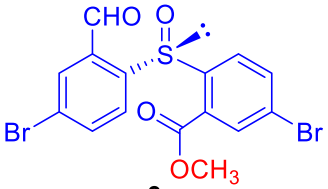

3n

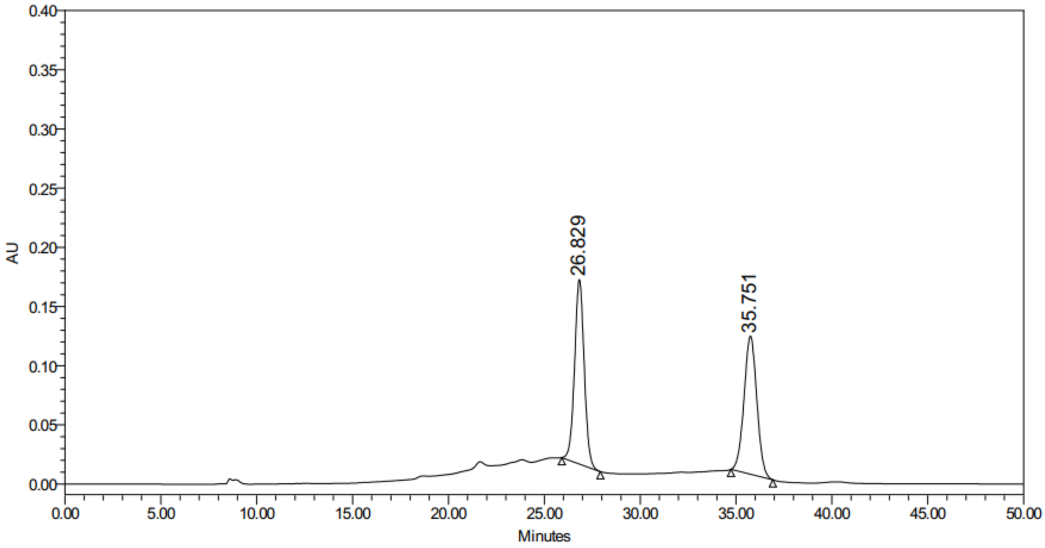

	RT	Height	Area	% Height	% Area
1	26.829	155814	5448524	57.17	50.09
2	35.751	116736	5429481	42.83	49.91

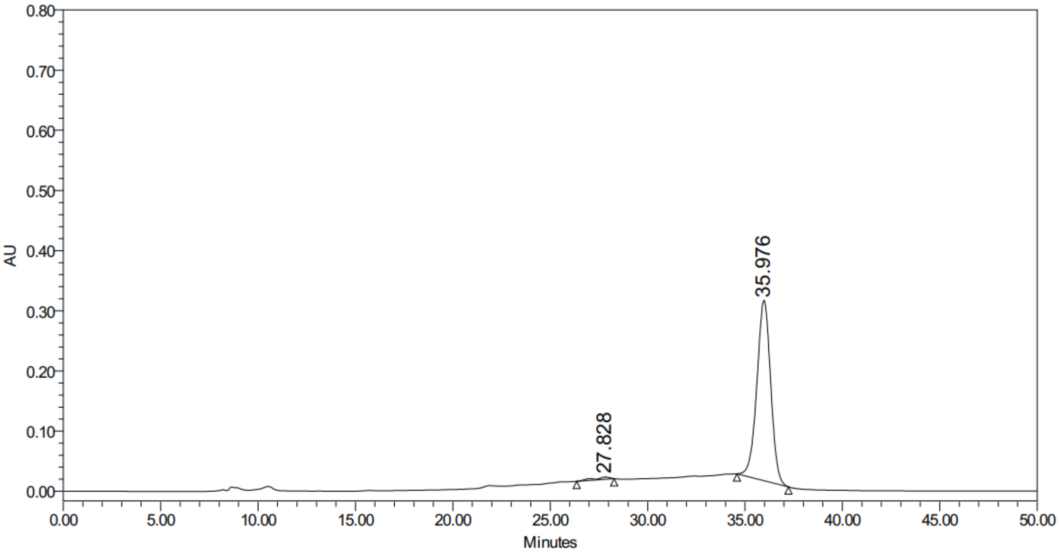

	RT	Height	Area	% Height	% Area
1	27.828	3522	215472	1.16	1.44
2	35.976	299449	14772330	98.84	98.56

Supplementary Figure 154 HPLC spectra of **3n**

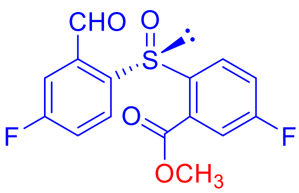

3o

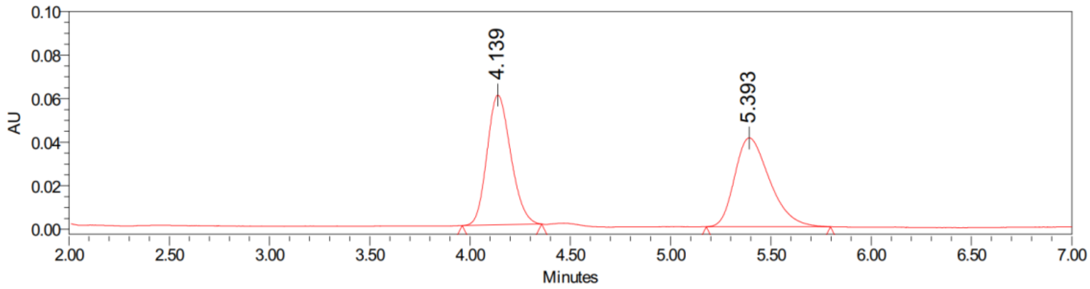

Peak Results

	RT	Height	Area	% Height	% Area
1	4.139	59635	490164	59.39	49.75
2	5.393	40781	495009	40.61	50.25

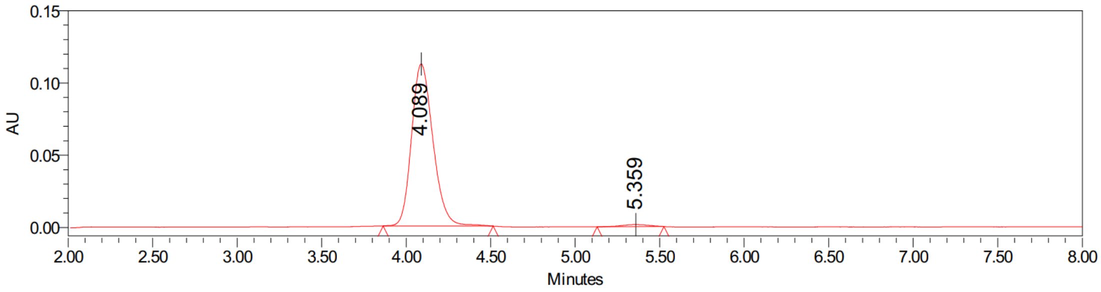

Peak Results

	RT	Height	Area	% Height	% Area
1	4.089	112170	988571	98.78	98.53
2	5.359	1386	14704	1.22	1.47

Supplementary Figure 155 UPLC spectra of **3o**

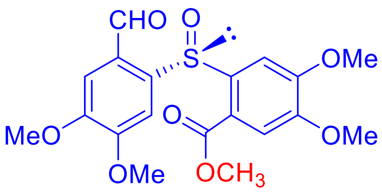

3p

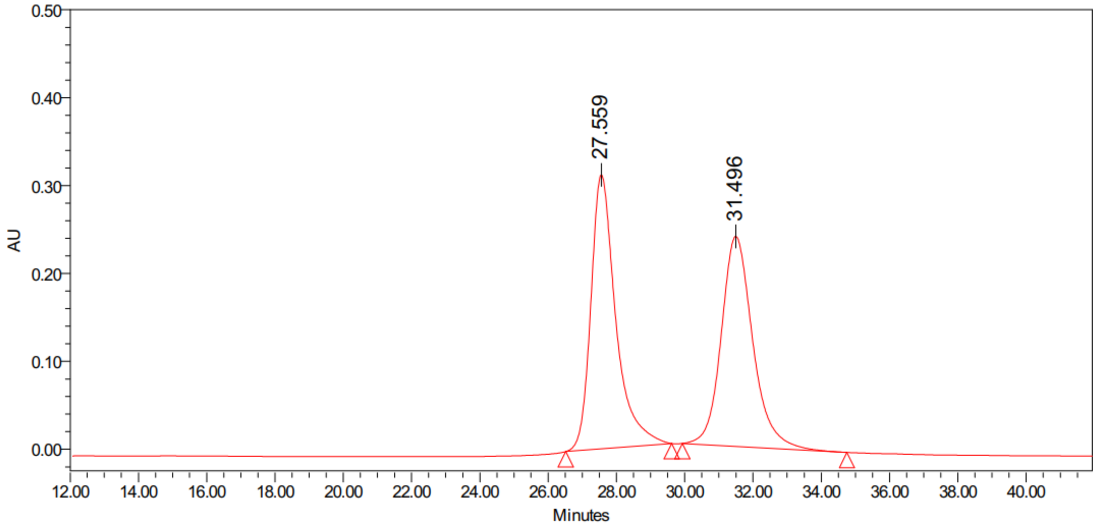

Peak Results

	RT	Area	Height	% Height	% Area
1	27.559	15819662	311947	56.63	50.80
2	31.496	15323566	238912	43.37	49.20

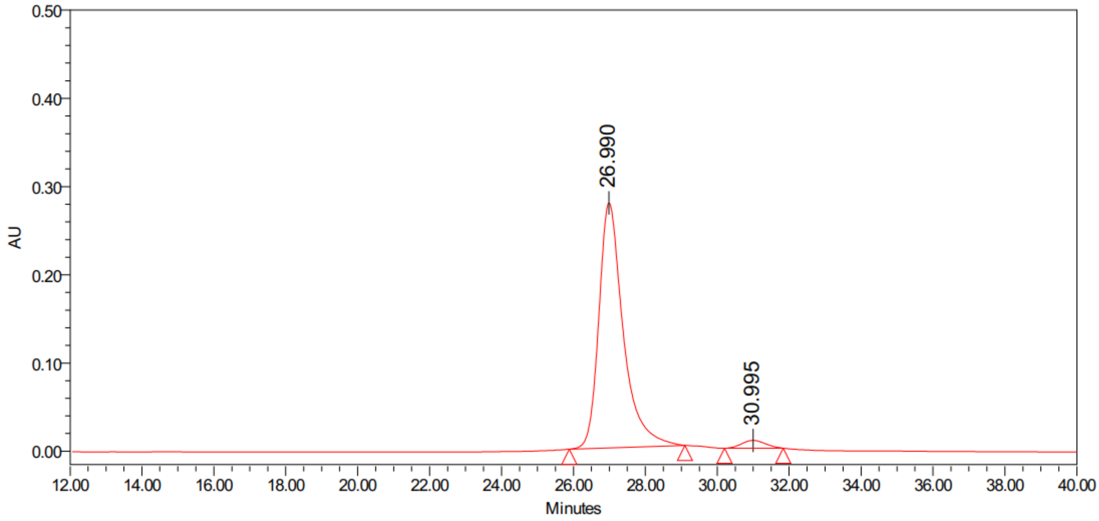

Peak Results

	RT	Area	Height	% Height	% Area
1	26.990	13085105	277942	96.91	97.00
2	30.995	405266	8866	3.09	3.00

Supplementary Figure 156 HPLC spectra of **3p**

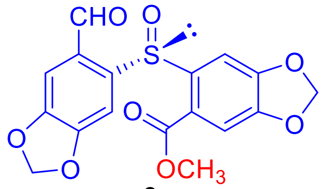

3q

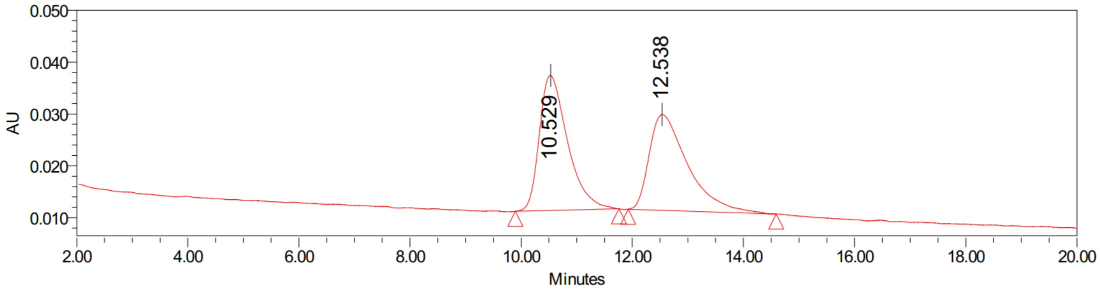

Peak Results

	RT	Height	Area	% Height	% Area
1	10.529	26050	918809	58.54	50.88
2	12.538	18448	886904	41.46	49.12

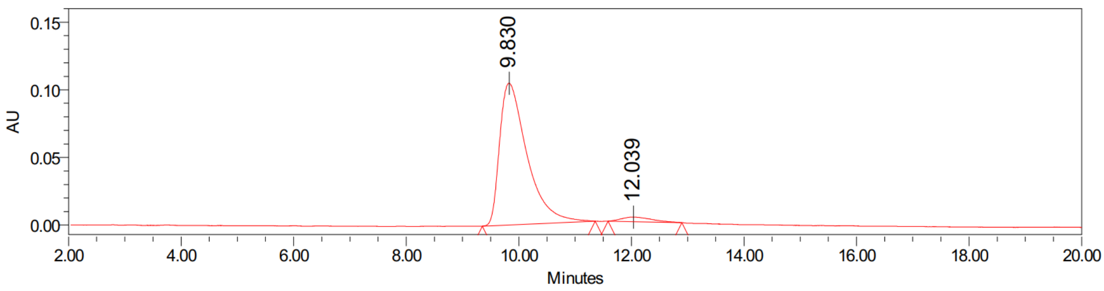

Peak Results

	RT	Height	Area	% Height	% Area
1	9.830	104808	3540830	96.91	96.50
2	12.039	3339	128408	3.09	3.50

Supplementary Figure 157 UPLC spectra of **3q**

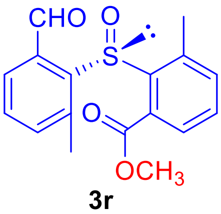

3r

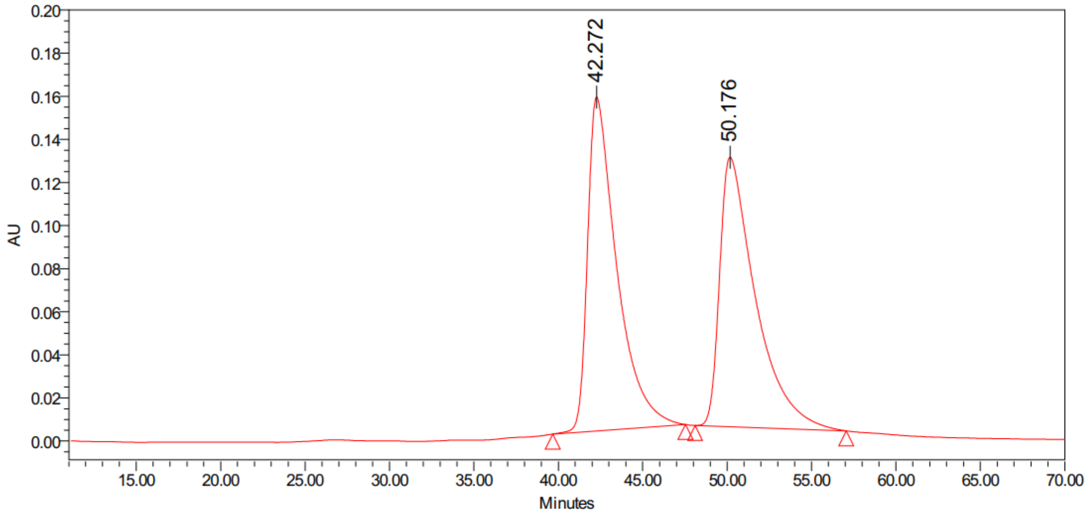

Peak Results

	RT	Area	Height	% Height	% Area
1	42.272	18621204	154960	55.34	50.24
2	50.176	18443111	125069	44.66	49.76

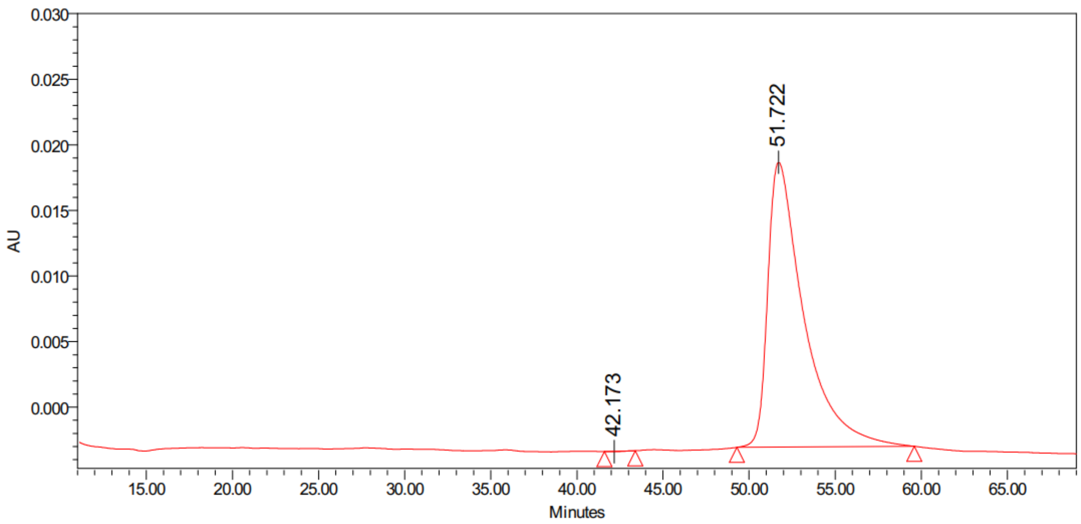

Peak Results

	RT	Area	Height	% Height	% Area
1	42.173	1494	-27	0.13	0.05
2	51.722	3168999	21730	99.87	99.95

Supplementary Figure 158 HPLC spectra of **3r**

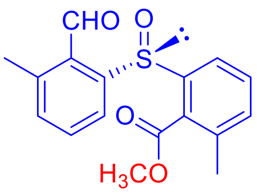

3s

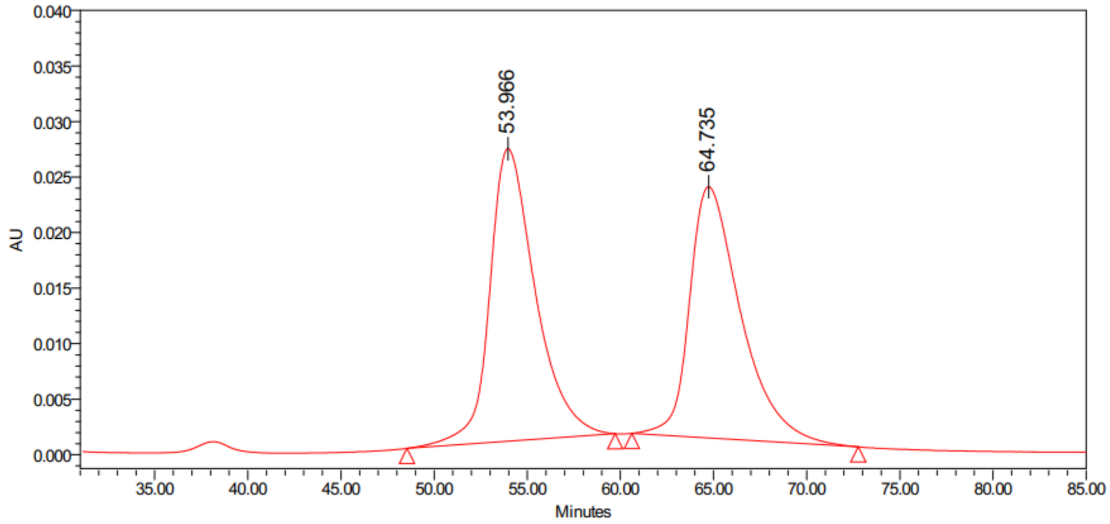

Peak Results

	RT	Area	Height	% Height	% Area
1	53.966	4500286	26341	53.80	50.91
2	64.735	4339653	22618	46.20	49.09

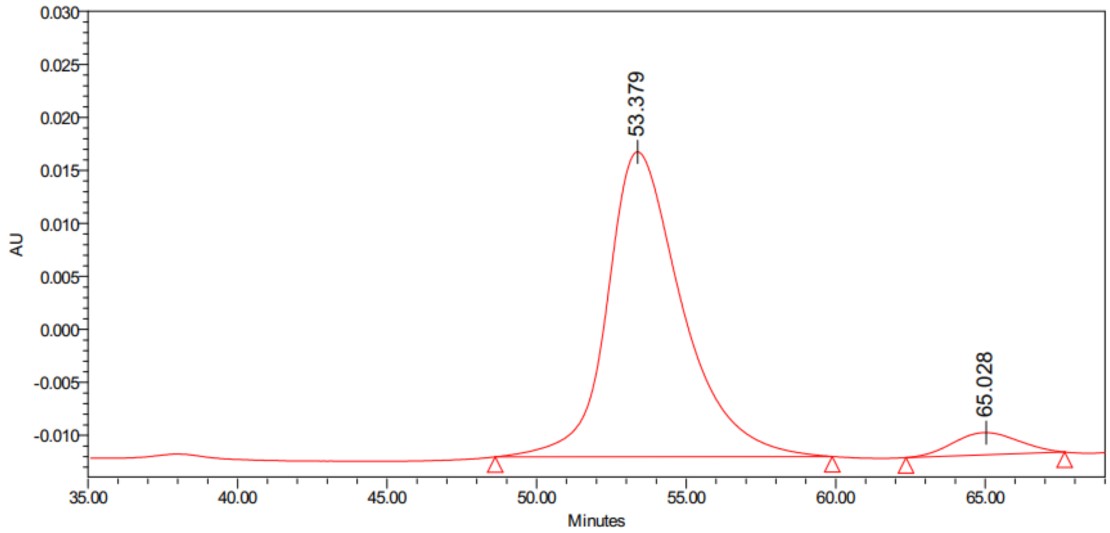

Peak Results

	RT	Area	Height	% Height	% Area
1	53.379	5020229	28756	93.20	94.00
2	65.028	320451	2097	6.80	6.00

Supplementary Figure 159 HPLC spectra of **3s**

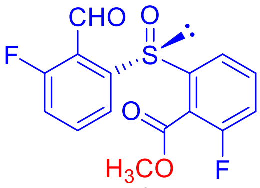

3t

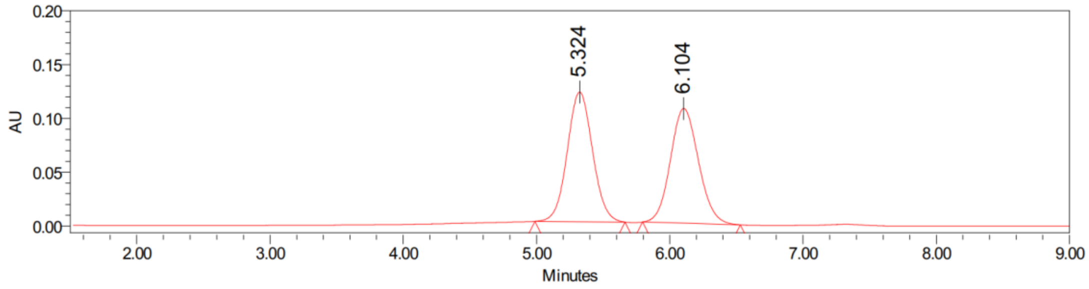

Peak Results

	RT	Height	Area	% Height	% Area
1	5.324	120444	1588993	53.06	50.43
2	6.104	106568	1561748	46.94	49.57

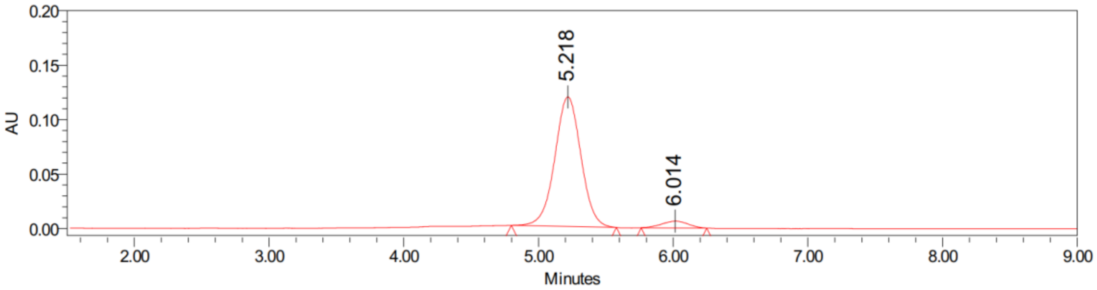

Peak Results

	RT	Height	Area	% Height	% Area
1	5.218	118864	1605895	95.05	95.03
2	6.014	6187	83945	4.95	4.97

Supplementary Figure 160 UPLC spectra of **3t**

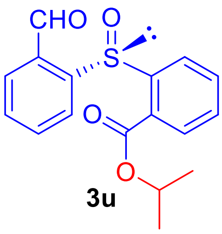

3u

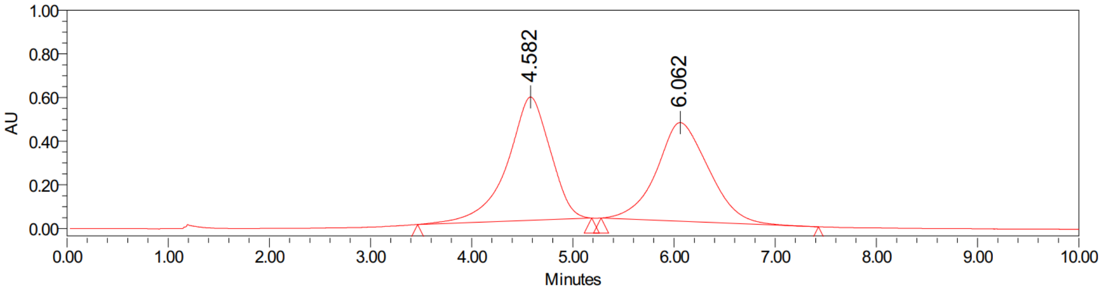

Peak Results

	RT	Height	Area	% Height	% Area
1	4.582	565284	16707084	55.57	50.79
2	6.062	452042	16184544	44.43	49.21

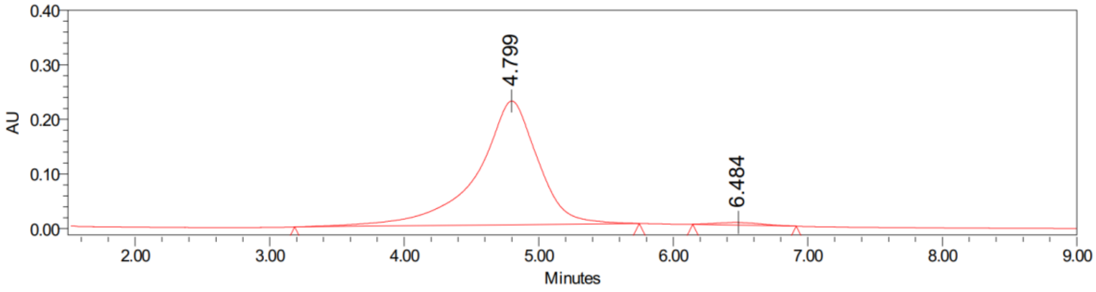

Peak Results

	RT	Height	Area	% Height	% Area
1	4.799	226717	7124019	97.94	98.50
2	6.484	4766	108220	2.06	1.50

Supplementary Figure 161 UPLC spectra of **3u**

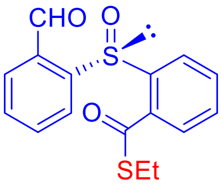

3v

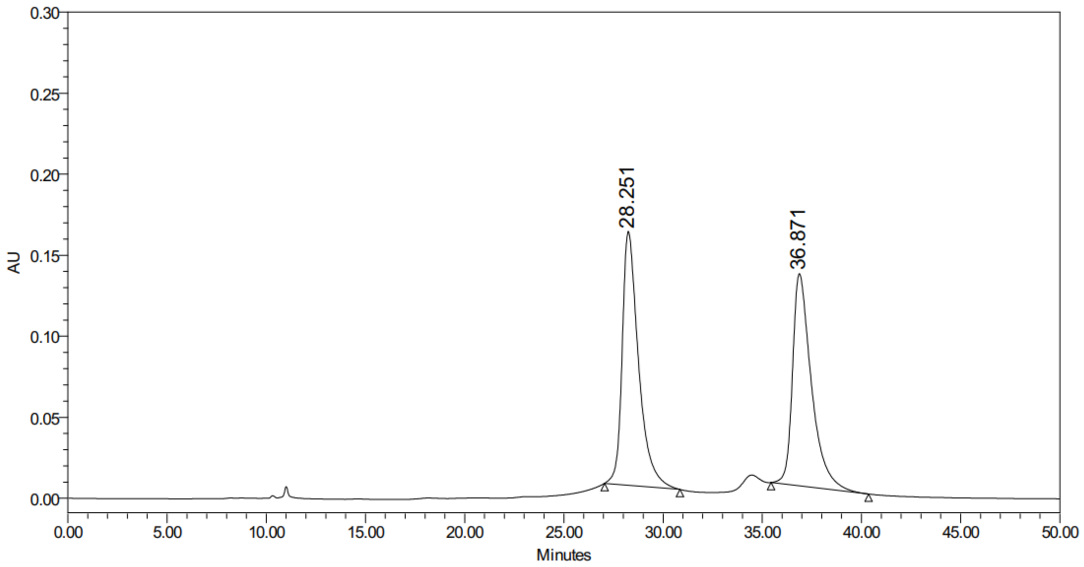

	RT	Height	Area	% Height	% Area
1	28.251	156486	8992543	54.45	50.89
2	36.871	130905	8679180	45.55	49.11

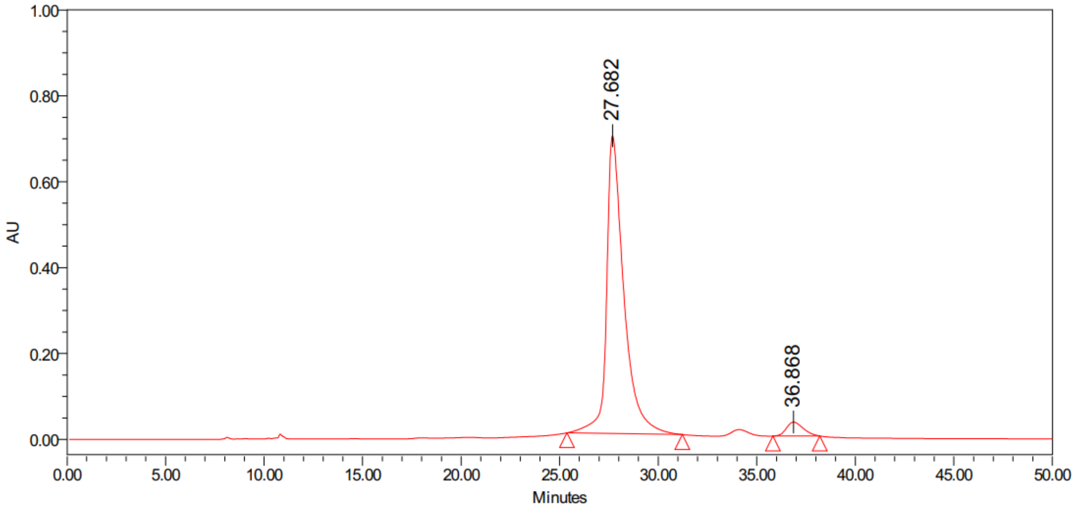

Peak Results

	RT	Area	Height	% Height	% Area
1	27.682	42275248	693996	95.52	95.62
2	36.868	1938764	32579	4.48	4.38

Supplementary Figure 162 HPLC spectra of **3v**

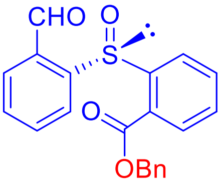

3w

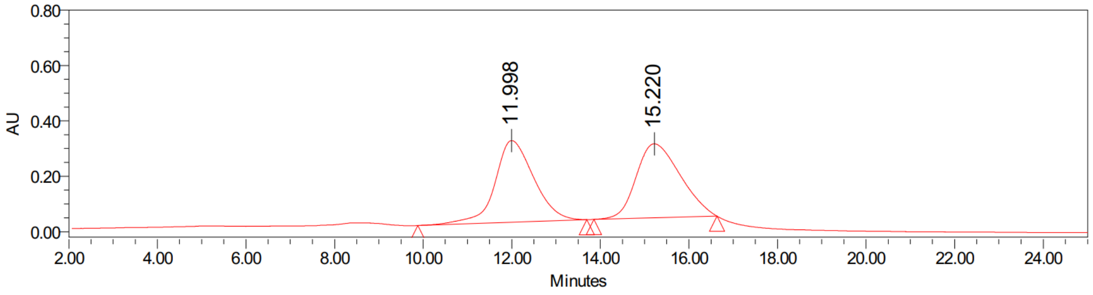

Peak Results

	RT	Height	Area	% Height	% Area
1	11.998	295194	17937644	52.47	49.33
2	15.220	267392	18423822	47.53	50.67

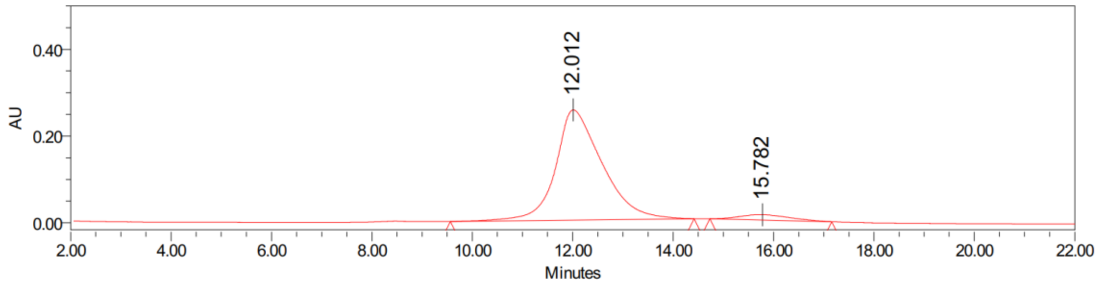

Peak Results

	RT	Height	Area	% Height	% Area
1	12.012	254254	16441154	95.32	95.03
2	15.782	12475	860438	4.68	4.97

Supplementary Figure 163 UPLC spectra of **3w**

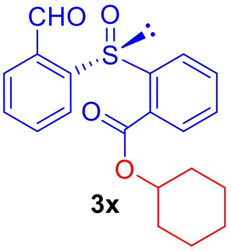

3x

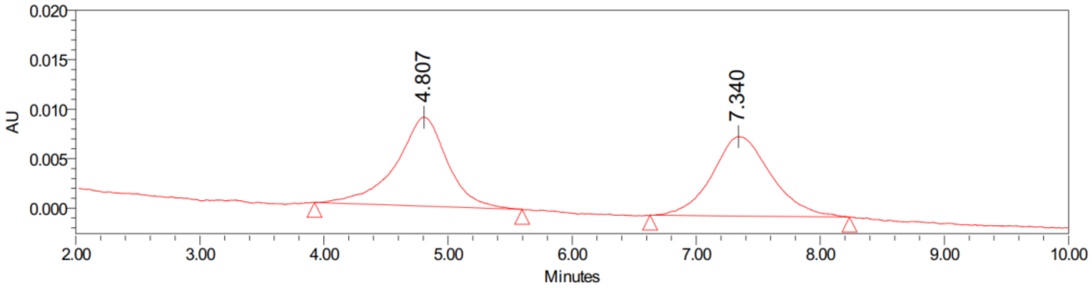

Peak Results

	RT	Height	Area	% Height	% Area
1	4.807	8990	259808	52.80	49.44
2	7.340	8036	265669	47.20	50.56

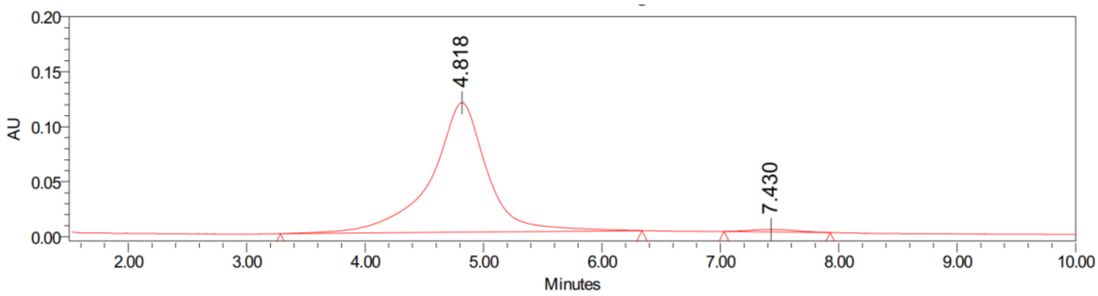

Peak Results

	RT	Height	Area	% Height	% Area
1	4.818	117708	3880183	98.16	98.50
2	7.430	2212	59159	1.84	1.50

Supplementary Figure 164 UPLC spectra of **3x**

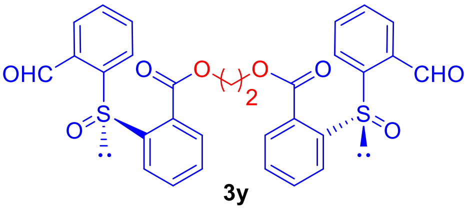

3y

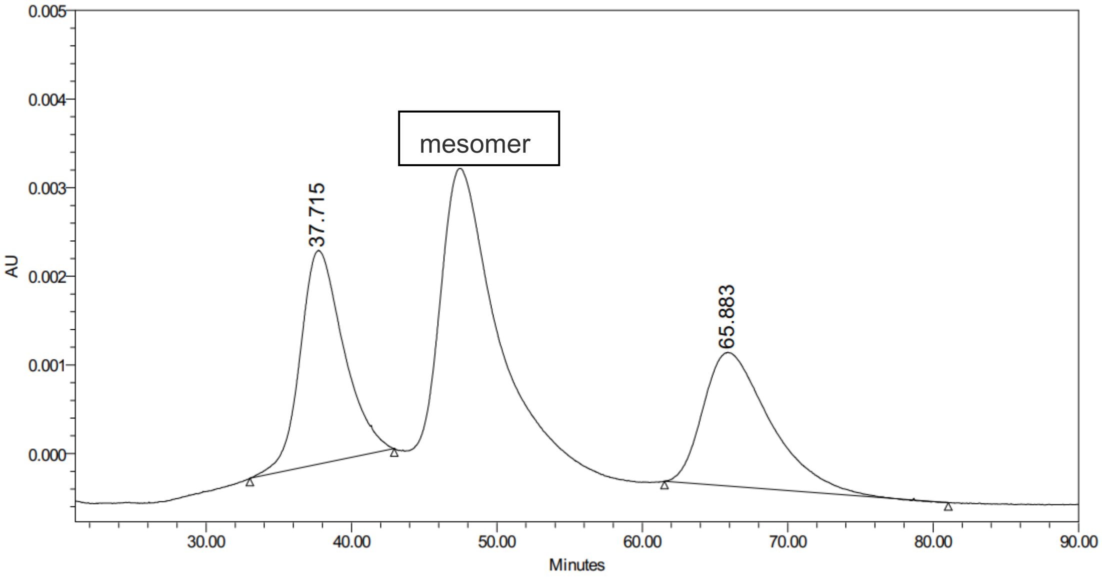

	RT	Height	Area	% Height	% Area
1	37.715	2412	499841	61.53	50.77
2	65.883	1508	484716	38.47	49.23

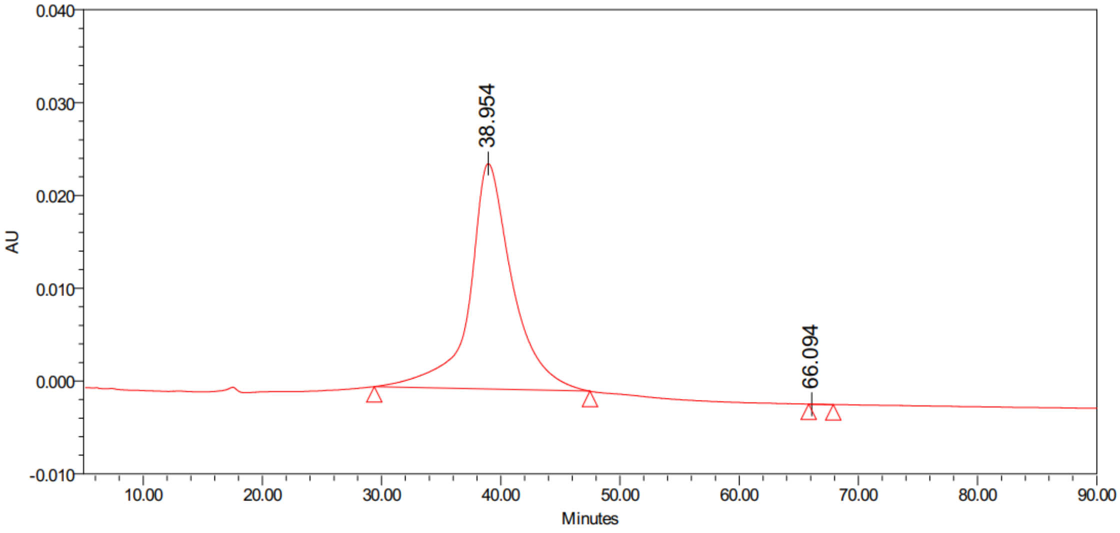

Peak Results

	RT	Area	Height	% Height	% Area
1	38.954	6099023	24312	99.97	100.00
2	66.094	296	-6	0.03	0.00

Supplementary Figure 165 HPLC spectra of **3y**

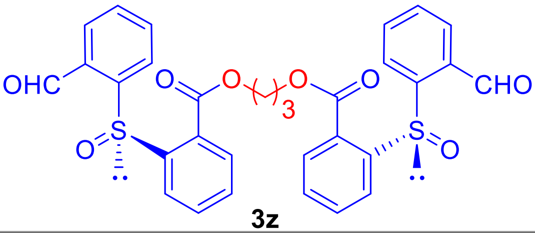

3z

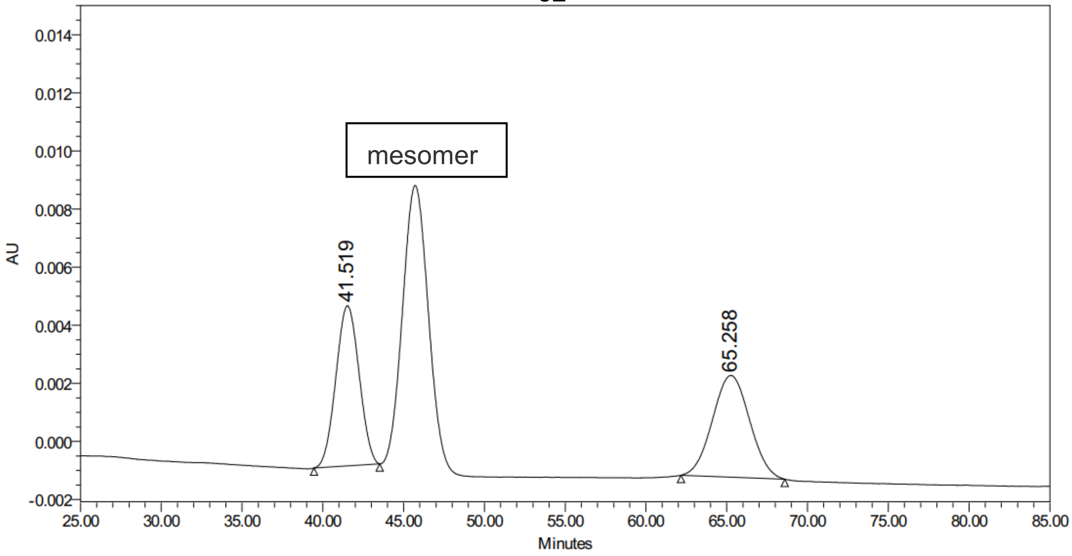

	RT	Height	Area	% Height	% Area
1	41.519	5510	549506	61.15	49.54
2	65.258	3501	559666	38.85	50.46

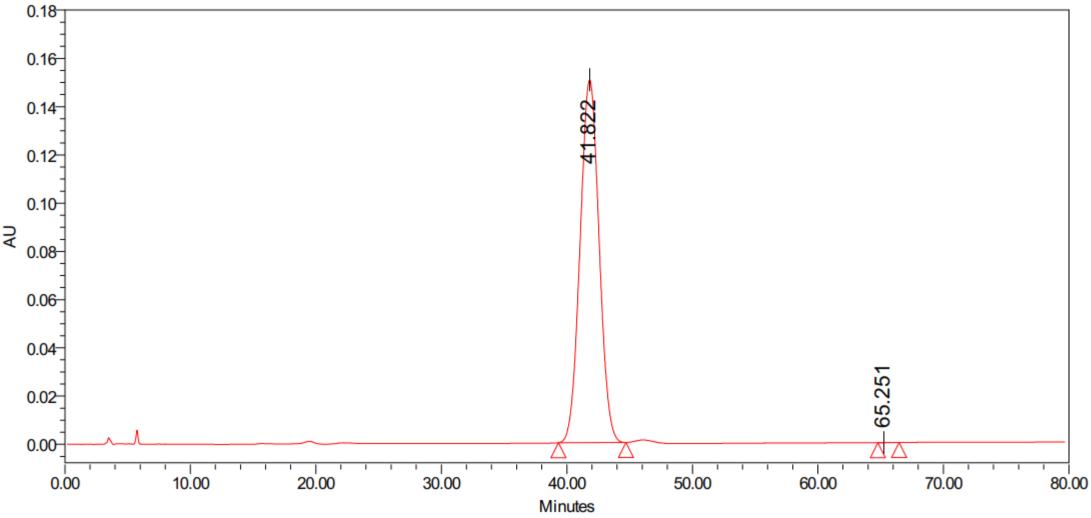

Peak Results

	RT	Height	Area	% Height	% Area
1	41.822	150450	15792960	100.00	100.00
2	65.251	2	104	0.00	0.00

Supplementary Figure 166 HPLC spectra of **3z**

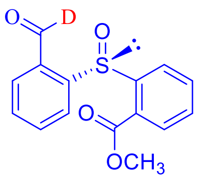

4a

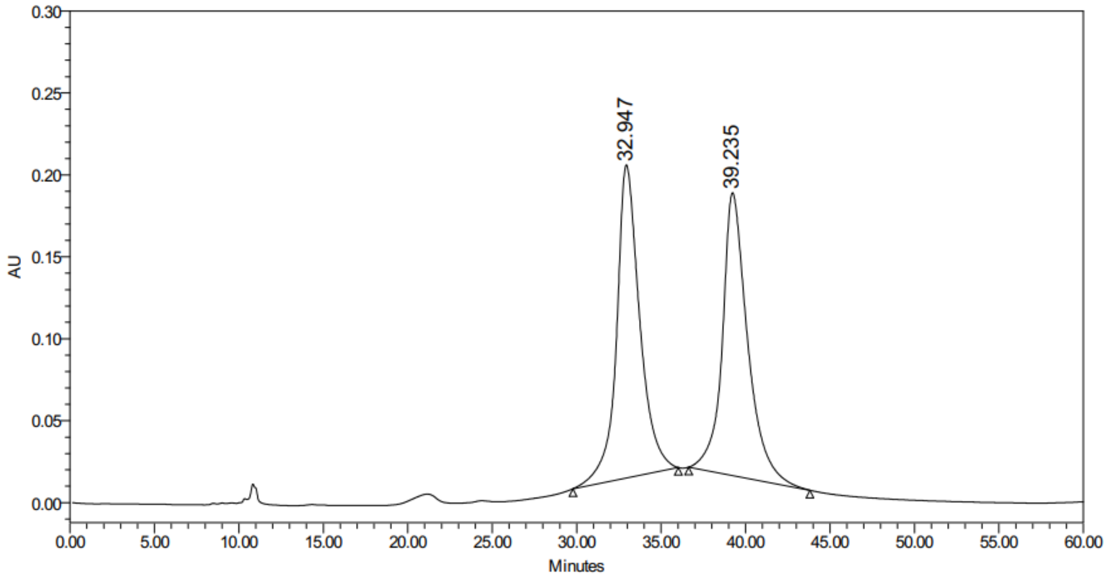

	RT	Height	Area	% Height	% Area
1	32.947	191006	18298144	52.54	50.43
2	39.235	172539	17988065	47.46	49.57

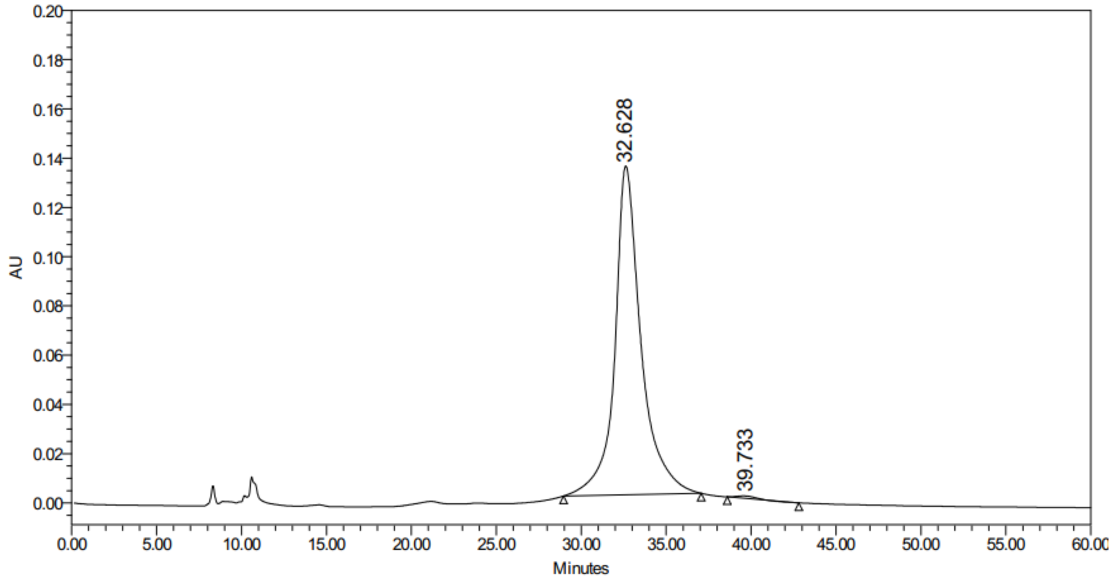

	RT	Height	Area	% Height	% Area
1	32.628	133519	14367937	99.25	99.42
2	39.733	1003	84442	0.75	0.58

Supplementary Figure 167 HPLC spectra of **4a**

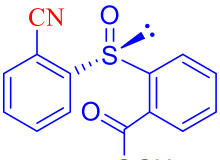

4b

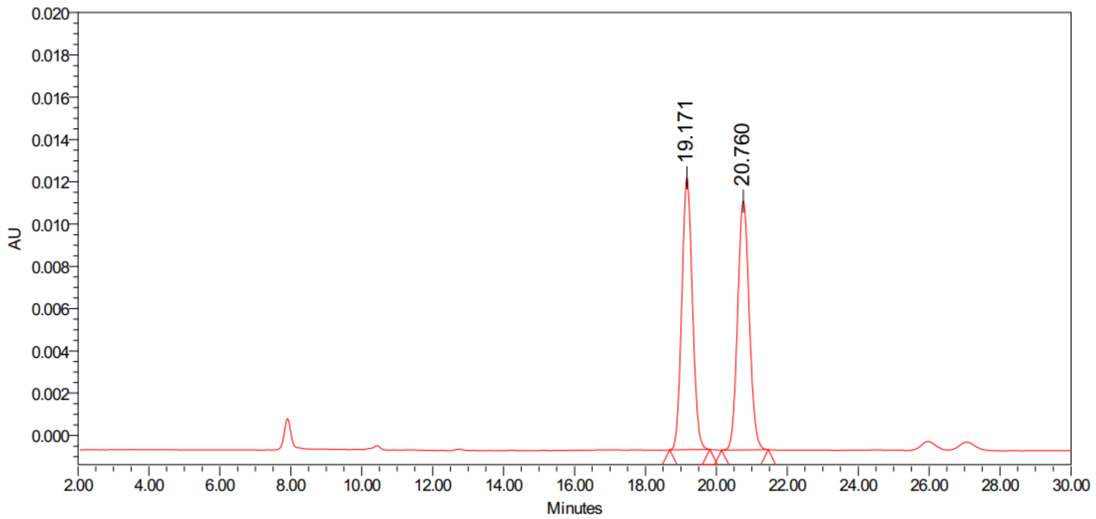

Peak Results

	RT	Area	Height	% Height	% Area
1	19.171	267737	12880	52.22	50.05
2	20.760	267225	11787	47.78	49.95

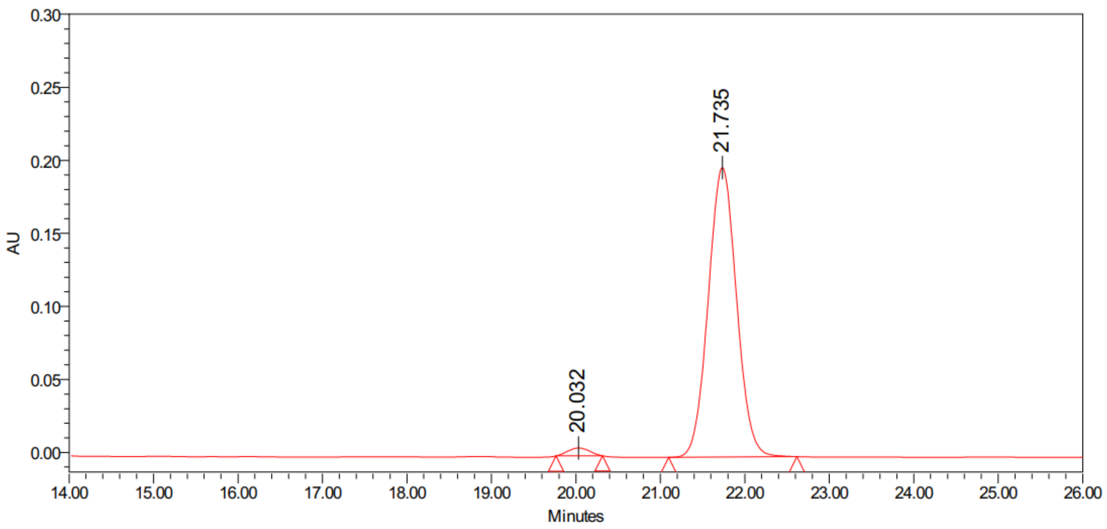

Peak Results

	RT	Area	Height	% Height	% Area
1	20.032	95730	5402	2.65	2.03
2	21.735	4618055	198281	97.35	97.97

Supplementary Figure 168 HPLC spectra of **4b**

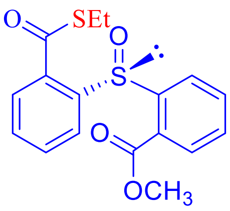

4c

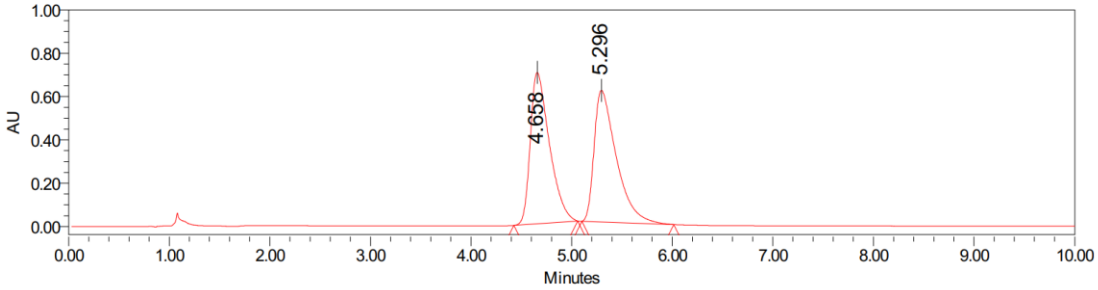

Peak Results

	RT	Height	Area	% Height	% Area
1	4.658	698601	9222846	53.49	49.81
2	5.296	607448	9291763	46.51	50.19

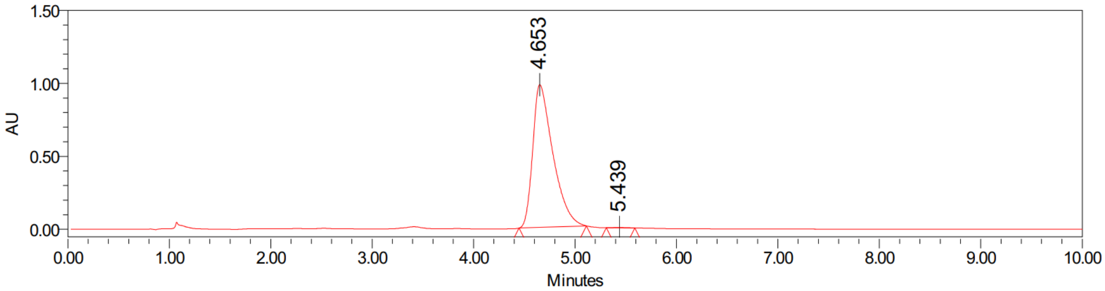

Peak Results

	RT	Height	Area	% Height	% Area
1	4.653	978214	13522568	99.70	99.80
2	5.439	2905	27045	0.30	0.20

Supplementary Figure 169 UPLC spectra of **4c**

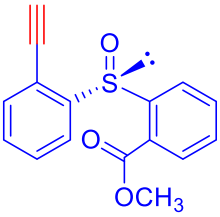

4d

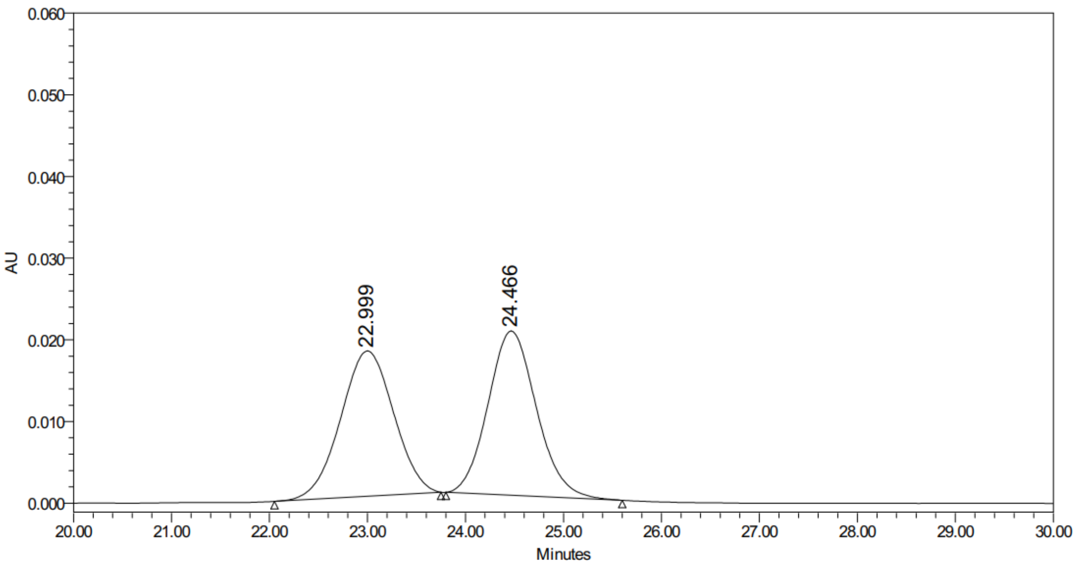

	RT	Height	Area	% Height	% Area
1	22.999	17789	669197	46.91	49.17
2	24.466	20130	691769	53.09	50.83

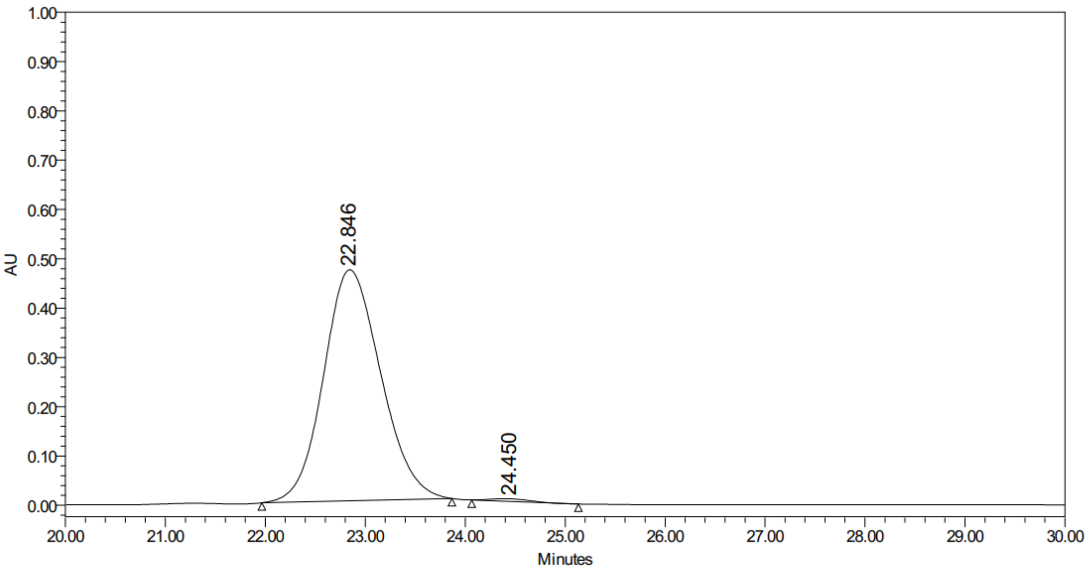

	RT	Height	Area	% Height	% Area
1	22.846	468764	18650173	98.87	99.24
2	24.450	5350	142008	1.13	0.76

Supplementary Figure 170 HPLC spectra of **4d**

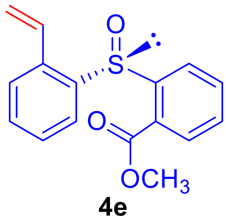

4e

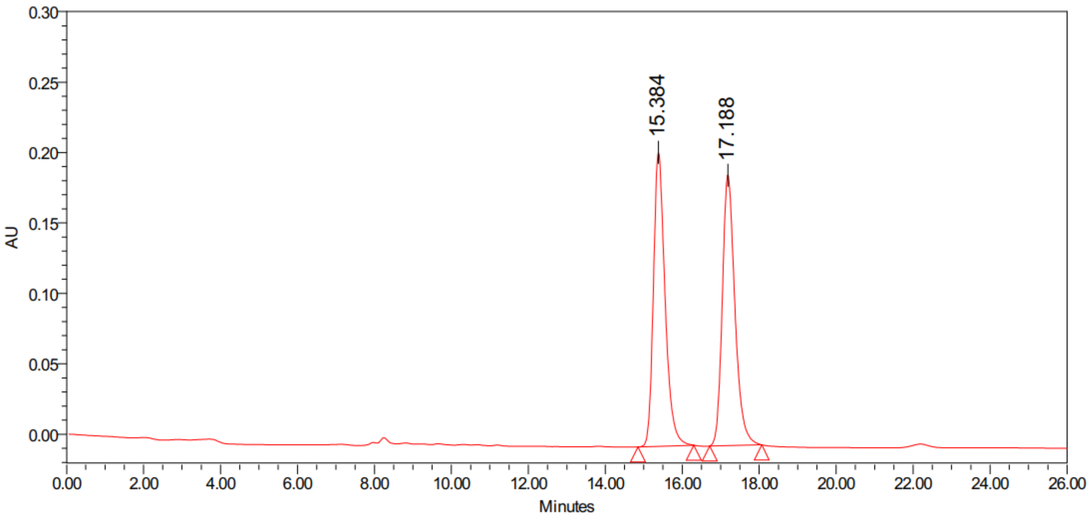

Peak Results

	RT	Area	Height	% Height	% Area
1	15.384	4379029	208760	52.08	50.07
2	17.188	4366229	192055	47.92	49.93

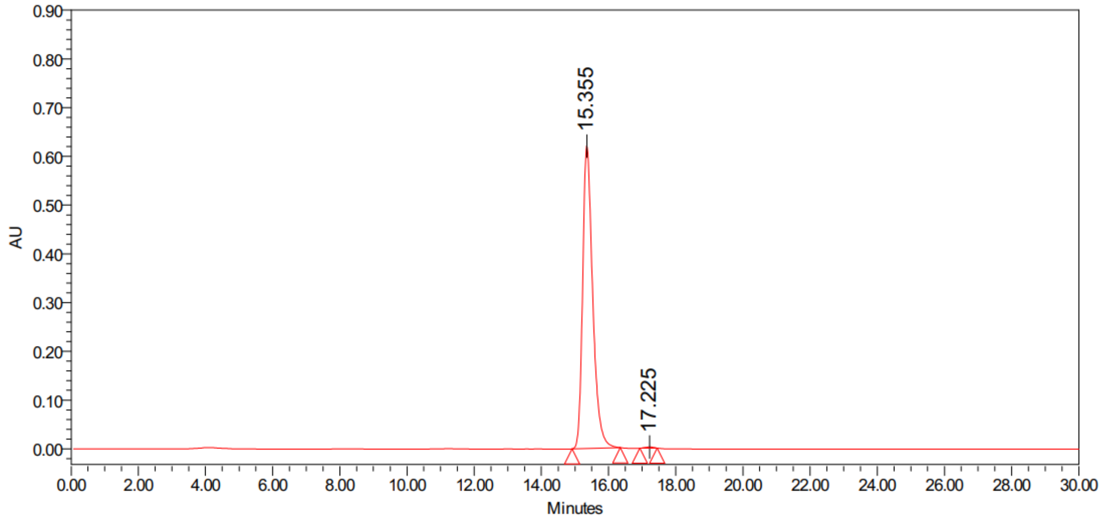

Peak Results

	RT	Area	Height	% Height	% Area
1	15.355	12954644	621741	99.61	99.67
2	17.225	42559	2449	0.39	0.33

Supplementary Figure 171 HPLC spectra of **4e**

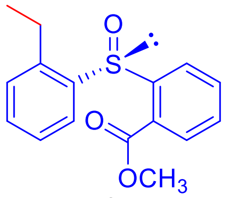

4f

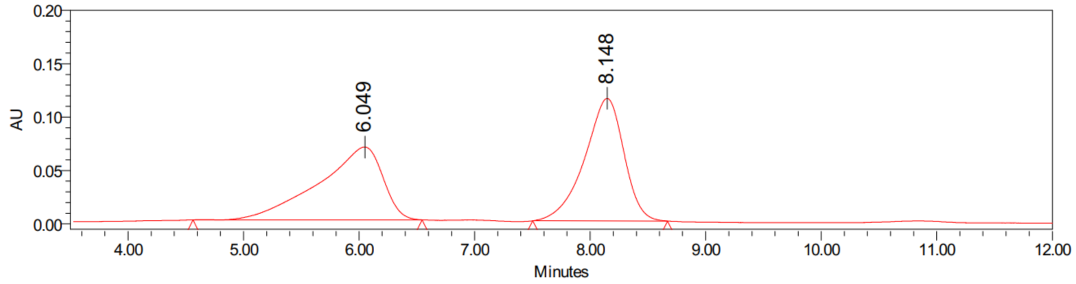

Peak Results

	RT	Height	% Height	Area (μV*sec)	% Area
1	6.049	68235	37.30	2624264	49.13
2	8.148	114722	62.70	2717174	50.87

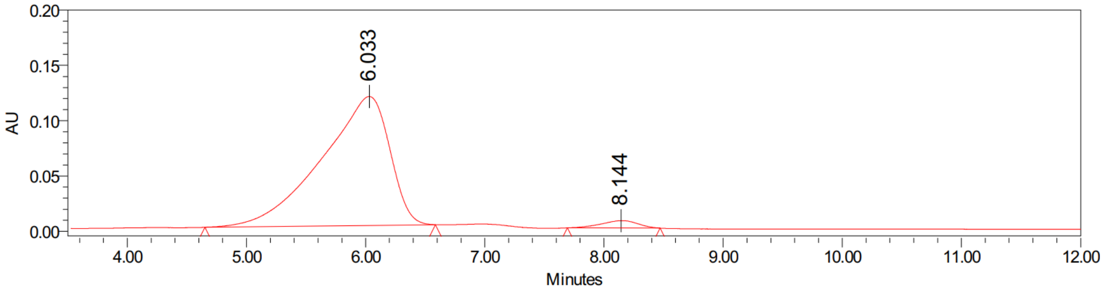

Peak Results

	RT	Height	Area	% Height	% Area
1	6.033	116643	4629999	94.58	97.03
2	8.144	6684	141843	5.42	2.97

Supplementary Figure 172 UPLC spectra of **4f**

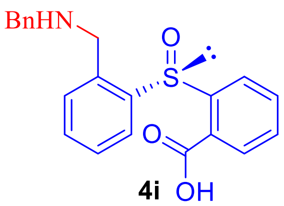

4i

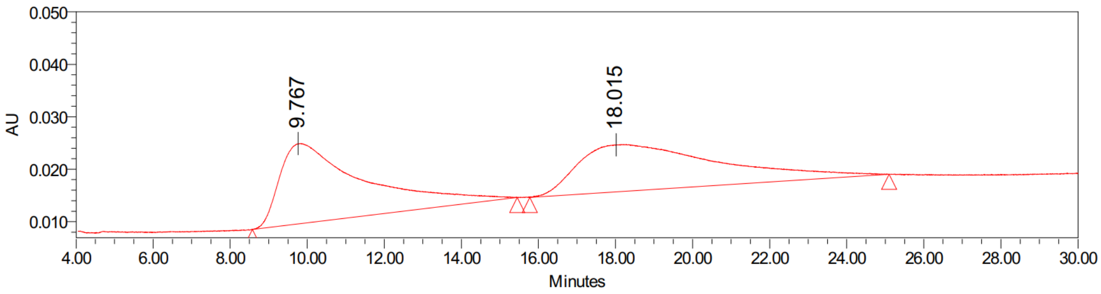

Peak Results

	RT	Height	Area	% Height	% Area
1	9.767	15339	2304582	63.10	50.80
2	18.015	8971	2232274	36.90	49.20

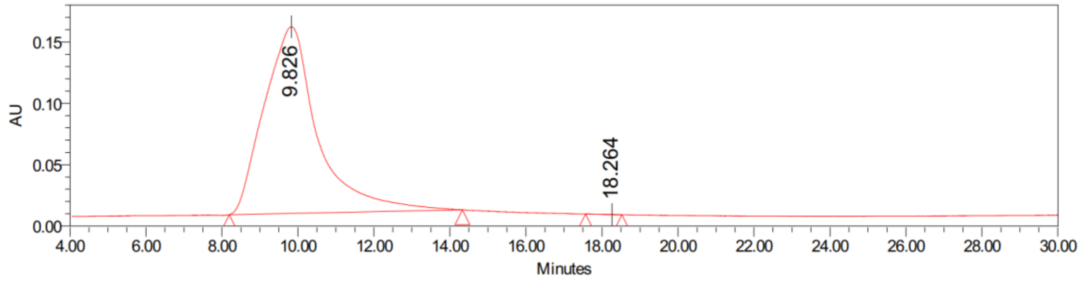

Peak Results

	RT	Height	Area	% Height	% Area
1	9.826	152055	14928157	99.92	99.98
2	18.264	121	2571	0.08	0.02

Supplementary Figure 173 UPLC spectra of **4i**

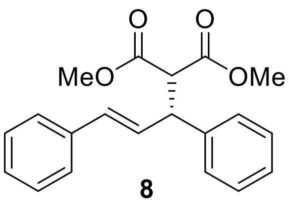

8

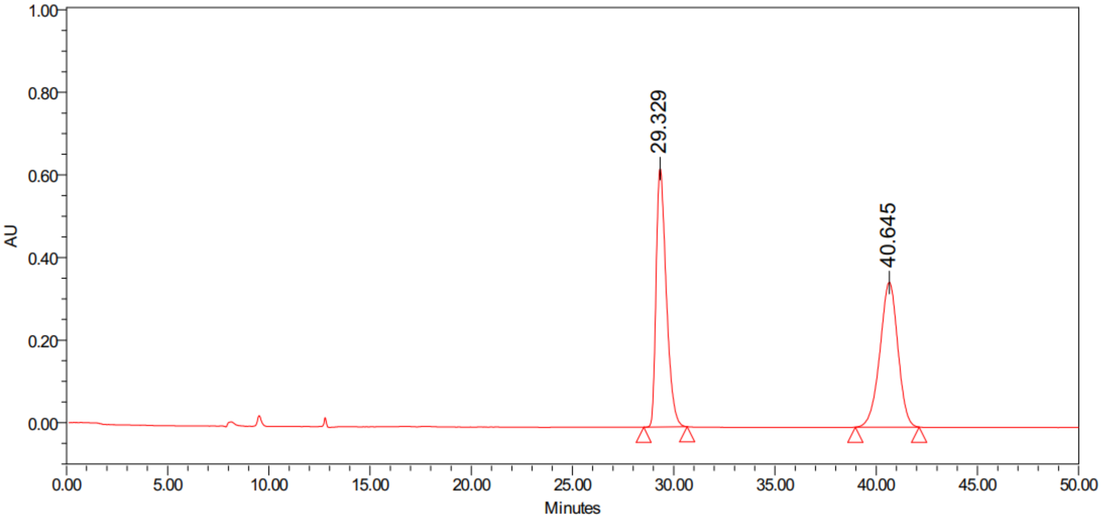

Peak Results

	RT	Area	Height	% Height	% Area
1	29.329	21815227	625687	64.09	50.17
2	40.645	21663843	350648	35.91	49.83

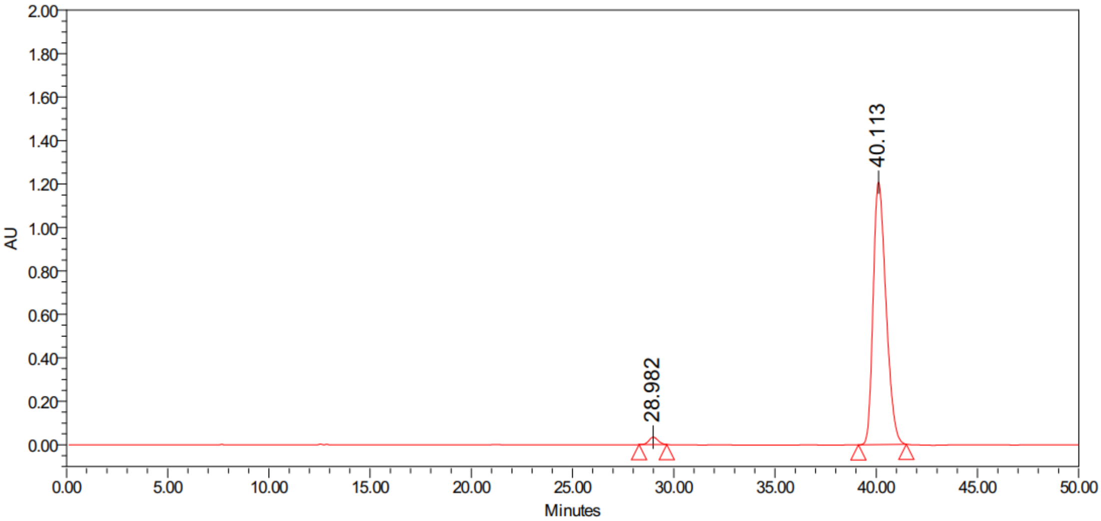

Peak Results

	RT	Area	Height	% Height	% Area
1	28.982	1074580	34300	2.76	1.98
2	40.113	53219251	1207723	97.24	98.02

Supplementary Figure 174 HPLC spectra of **8**

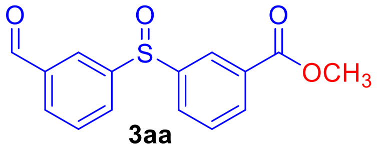

3aa

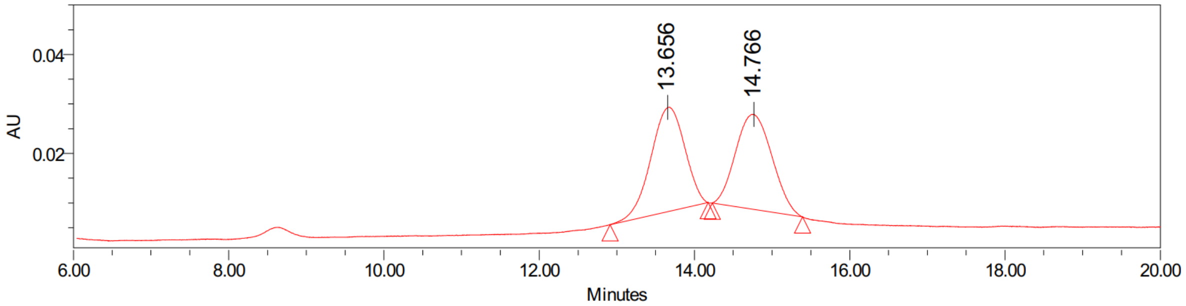

Peak Results

	RT	Height	Area	% Height	% Area
1	13.656	21048	640508	52.35	50.68
2	14.766	19155	623382	47.65	49.32

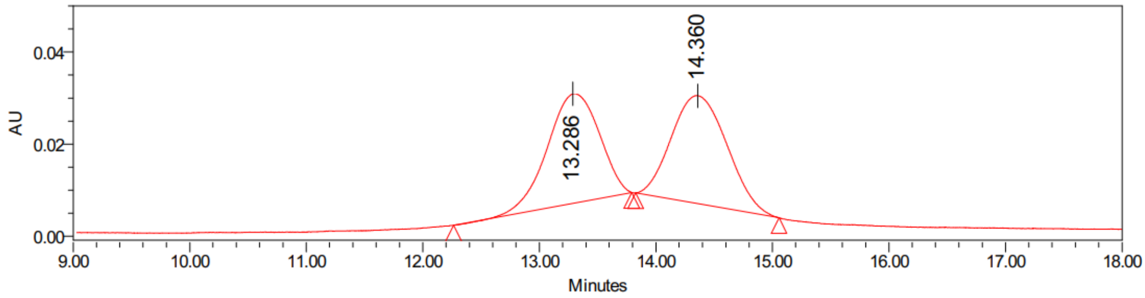

Peak Results

	RT	Height	Area	% Height	% Area
1	13.286	23772	739439	50.35	48.72
2	14.360	23440	778169	49.65	51.28

Supplementary Figure 175 UPLC spectra of **3aa**

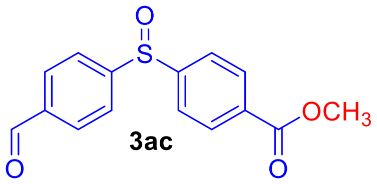

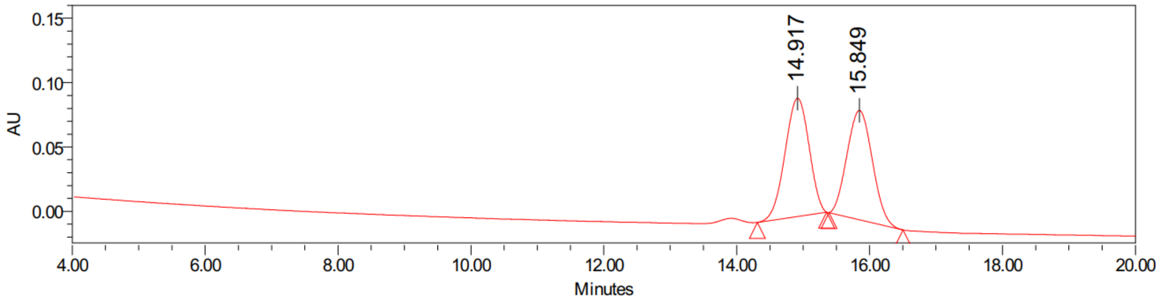

Peak Results

	RT	Height	Area	% Height	% Area
1	14.917	92063	2311811	52.01	50.66
2	15.849	84962	2251443	47.99	49.34

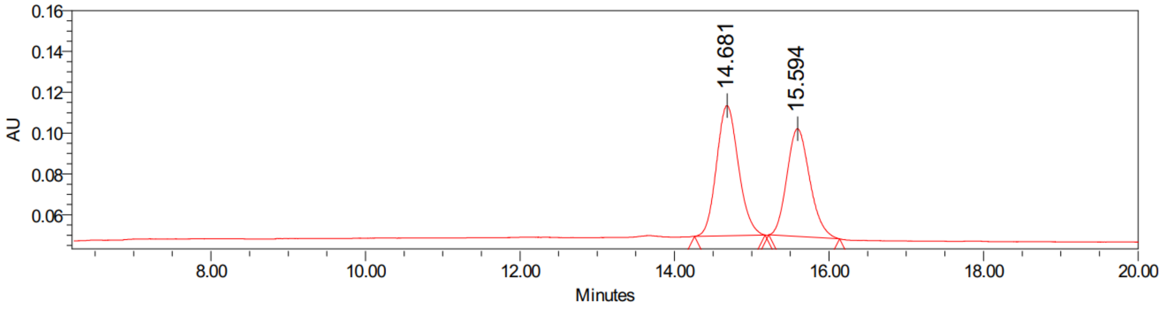

Peak Results

	RT	Height	Area	% Height	% Area
1	14.681	63751	1253414	54.66	53.50
2	15.594	52888	1089421	45.34	46.50

Supplementary Figure 176 UPLC spectra of **3ac**

X-ray crystallography

Colorless block crystal of **1a** and **1aa** was obtained by vaporization of an ethyl acetate / petroleum ether solution. A colorless block crystal of compound **1c**, **1i** and **1ac** was obtained by vaporization of a petroleum ether / dichloromethane solution. A colorless needle-like crystal of **3a** was obtained by vaporization of ethyl acetate / petroleum ether solution. The corresponding structures and CCDC numbers were listed in supplementary figure 177-182. These data can be obtained free of charge from The Cambridge Crystallographic Data Centre *via* www.ccdc.cam.ac.uk/data_request/cif.

Supplementary Figure 177. X-ray crystallography of **1a**

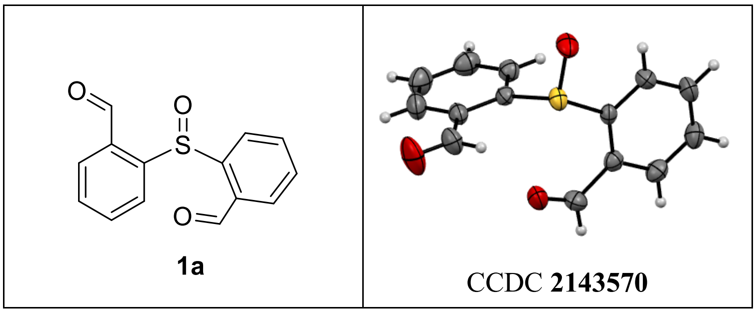

Crystal Data: $C_{14}H_{10}O_3S$, M_r = 258.28, Monoclinic, $P2_1/c$ (No. 14), a = 9.3038(11) Å, b = 14.9489(12) Å, c = 8.6150(9) Å, *a* = 90.00°, *b* = 102.217(3)°, *g* = 90.00°, *V* = 1171.1(2) Å^3, *T* = 298(2) K, *Z* = 4, *Z'* = 1, *m*(MoK$_a$) = 0.261, 5741 reflections measured, 2062 unique (R_{int} = 0.0450) which were used in all calculations. The final wR_2 was 0.1176 (all data) and R_1 was 0.0456 (I≥2 *s*(I)).

Supplementary Figure 178. X-ray crystallography of **1c**

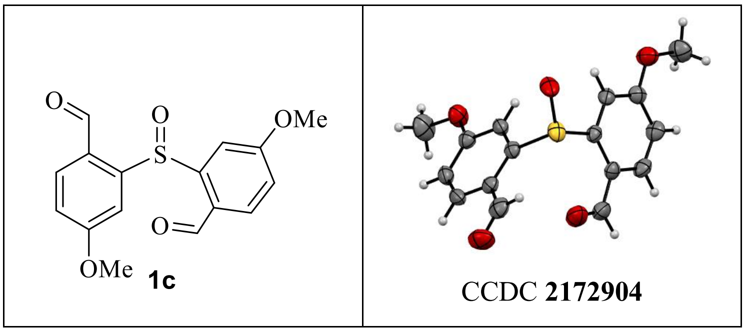

Crystal Data: $C_{16}H_{14}O_5S$, M_r = 318.33, monoclinic, $P2_1/n$ (No. 14), a = 10.4572(11)

Å, b = 11.2772(12) Å, c = 12.1814(14) Å, *b* = 94.720(4)°, *a* = *g* = 90°, *V* = 1431.7(3) Å^3, *T* = 298(2) K, *Z* = 4, *Z'* = 1, *m*(MoK_a) = 0.248, 6753 reflections measured, 2531 unique (R_{int} = 0.0519) which were used in all calculations. The final wR_2 was 0.1388 (all data) and R_1 was 0.0523 (I≥2 *s*(I)).

Supplementary Figure 179. X-ray crystallography of **1i**

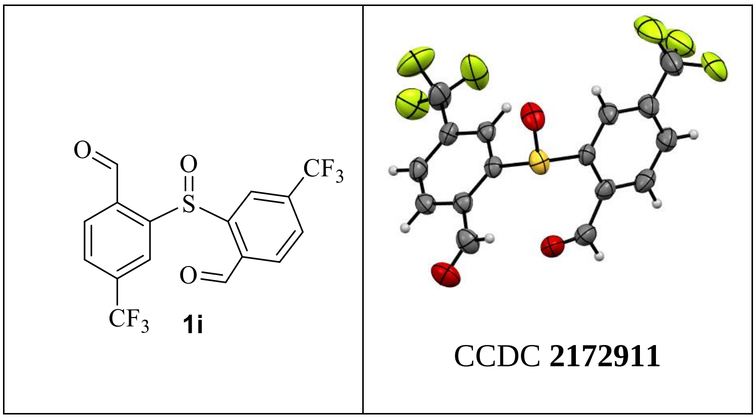

Crystal Data: $C_{16}H_8F_6O_3S$, M_r = 394.28, monoclinic, $P2_1/c$ (No. 14), a = 9.5510(9) Å, b = 14.2339(14) Å, c = 11.3070(11) Å, *b* = 101.140(3)°, *a* = *g* = 90°, *V* = 1508.2(3) Å^3, *T* = 298(2) K, *Z* = 4, *Z'* = 1, *m*(MoK_a) = 0.299, 7369 reflections measured, 2641 unique (R_{int} = 0.0510) which were used in all calculations. The final wR_2 was 0.2120 (all data) and R_1 was 0.0719 (I≥2 *s*(I)).

Supplementary Figure 180. X-ray crystallography of **1aa**

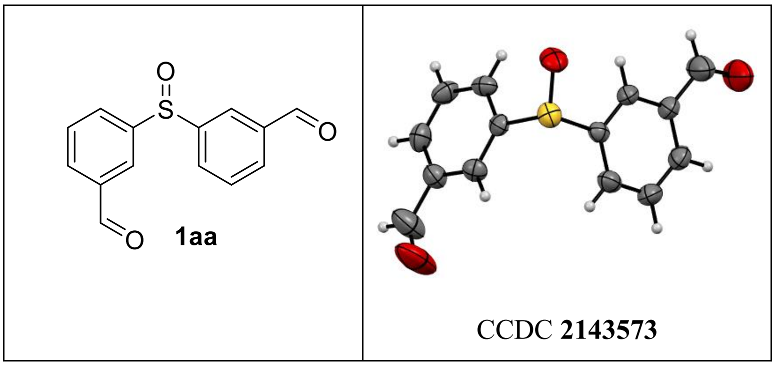

Crystal Data: $C_{14}H_{10}O_3S$, M_r = 258.28, Monoclinic, $P2_1/n$ (No. 14), a = 14.6195(13) Å, b = 13.5854(12) Å, c = 18.1911(16) Å, *a* = 90.00°, *b* = 94.119(2)°, *g* = 90.00°, *V* = 3603.6(6) Å^3, *T* = 298(2) K, *Z* = 12, *Z'* = 3, *m*(MoK_a) = 0.265, 17489 reflections measured, 6347 unique (R_{int} = 0.0746) which were used in all calculations. The final

wR_2 was 0.1508 (all data) and R_1 was 0.0597 (I≥2 *s*(I)).

Supplementary Figure 181. X-ray crystallography of **1ac**

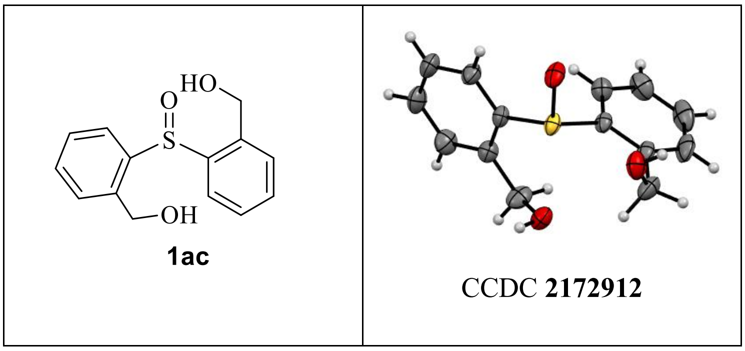

Crystal Data: $C_{14}H_{14}O_3S$, M_r = 262.31, triclinic, *P*-1 (No. 2), a = 7.3198(6) Å, b = 8.5308(12) Å, c = 11.1512(18) Å, *a* = 69.860(9)°, *b* = 73.143(10)°, *g* = 79.416(11)°, *V* = 622.93(15) Å^3, *T* = 298 K, *Z* = 2, *Z'* = 1, *m*(CuK$_a$) = 2.296, 8505 reflections measured, 2397 unique (R_{int} = 0.1184) which were used in all calculations. The final wR_2 was 0.1503 (all data) and R_1 was 0.0516 (I≥2 *s*(I)).

Supplementary Figure 182. X-ray crystallography of **3a**

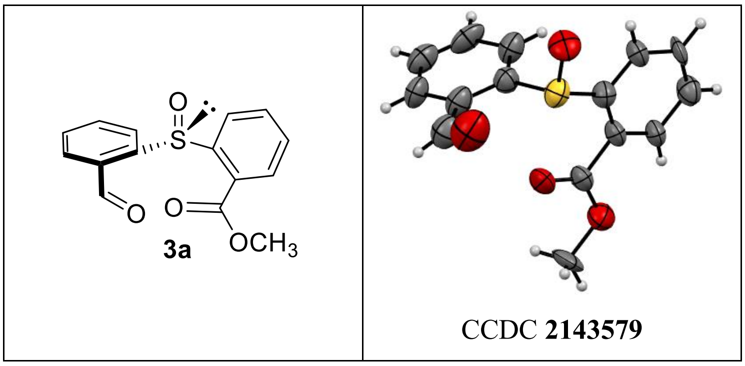

Crystal Data: $C_{15}H_{12}O_4S$, M_r = 288.31, monoclinic, *C*2 (No. 5), a = 19.9512(18) Å, b = 7.8209(9) Å, c = 19.8489(19) Å, *b* = 118.666(5)°, *a* = *g* = 90°, *V* = 2717.5(5) Å^3, *T* = 293(2) K, *Z* = 8, *Z'* = 2, *m*(MoK$_a$) = 0.248, 6599 reflections measured, 4412 unique (R_{int} = 0.0744) which were used in all calculations. The final wR_2 was 0.2597 (all data) and R_1 was 0.0990 (I≥2 *s*(I)).

VI. Supplementary References

1. Boger, D. L. & Wolkenberg, S. E. Total synthesis of amaryllidaceae alkaloids utilizing sequential intramolecular heterocyclic azadiene diels–alder reactions of an unsymmetrical 1,2,4,5-tetrazine. *J. Org. Chem.* **65**, 9120–9124 (2000).
2. Rachwalski, M & Kiełbasinski, P. et al. Enzyme-promoted desymmetrization of bis(2-hydroxymethylphenyl) sulfoxide as a route to tridentate chiral catalysts. *Tetrahedron: Asymmetry*. **19**, 2096-2101 (2008).
3. Geng, H. Chen, X. Gui, J. & Wang, W. *et al.* Practical synthesis of C1 deuterated aldehydes enabled by NHC catalysis. *Nat. Catal.* **2,** 1071–1077 (2019).
4. Lv, Y. Luo, G. Liu, Q. & Y. R. Chi. *et al.* Catalytic atroposelective synthesis of axially chiral benzonitriles *via* chirality control during bond dissociation and CN group formation. *Nat. Commun.* **13,** 36 (2022).
5. Orbe, M. E. Zanini, M. Quinonero, O. & Echavarren A. M. Gold- or indium-catalyzed cross-coupling of bromoalkynes with allylsilanes through a concealed rearrangement. *ACS Catal*. **9**, 7817–7822 (2019).
6. Schwekendiek, K. Glorius, F. Efficient oxidative synthesis of 2-Oxazolines. *Synthesis* **18**, 2996-3002 (2006).
7. Morales, S. Guijarro, F. G. & Cid M. B. A General Aminocatalytic method for the synthesis of aldimines. *J. Am. Chem. Soc*. **136**, 1082-1089 (2014).
8. Peng, X. Xu, J. Chi, Y. R. & Jin, Z. Chemo-selective cross reaction of two enals *via* carbene-catalyzed dual activation. *Chem. Sci*. **11**, 12533-12539 (2020).
9. Hiroi, K. Suzuki, Y. & Abe, I. (*S*)-Proline-derived new chiral ligands with phosphino, organosulfur or organoselenenyl functionality as an enantiocontrollable coordinating element. *Tetrahedron Asymmetry* **10,** 1173–1188 (1999).
10. Frisch, M. J.; Trucks, G. W.; Schlegel, H. B.; Scuseria, G. E.; Robb, M. A.; Cheeseman, J. R.; Scalmani, G.; Barone, V.; Mennucci, B.; Petersson, G. A.; Nakatsuji, H.; Caricato, M.; Li, X.; Hratchian, H. P.; Izmaylov, A. F.; Bloino, J.; Zheng, G.; Sonnenberg, J. L.; Hada, M.; Ehara, M.; Toyota, K.; Fukuda, R.;

Hasegawa, J.; Ishida, M.; Nakajima, T.; Honda, Y.; Kitao, O.; Nakai, H.; Vreven, T.; Montgomery, J. A., Jr.; Peralta, J. E.; Ogliaro, F.; Bearpark, M.; Heyd, J. J.; Brothers, E.; Kudin, K. N.; Staroverov, V. N.; Keith, T.; Kobayashi, R.; Normand, J.; Raghavachari, K.; Rendell, A.; Burant, J. C.; Iyengar, S. S.; Tomasi, J.; Cossi, M.; Rega, N.; Millam, J. M.; Klene, M.; Knox, J. E.; Cross, J. B.; Bakken, V.; Adamo, C.; Jaramillo, J.; Gomperts, R.; Stratmann, R. E.; Yazyev, O.; Austin, A. J.; Cammi, R.; Pomelli, C.; Ochterski, J. W.; Martin, R. L.; Morokuma, K.; Krzewski, V. G., Voth, G. A.; Salvador, P.; Dannenberg, J. J.; Dapprich, S.; Daniels, A. D.; Farkas, O.; Foresman, J. B.; Ortiz, J. V.; Cioslowski, J.; Fox, D. J.Gaussian 16, rev. A.03.; Gaussian, Inc.: Wallingford, CT, 2016.

11. T Lu, Q Chen, Shermo: A general code for calculating molecular thermodynamic properties, *Comput. Theor. Chem*. **1200**, 113249 (2021).
12. CYLview20; Legault, C. Y., Université de Sherbrooke, 2020 (http://www.cylview.org)
13. Peng, Q. Duarte, F. & Paton R. S. Computing organic stereoselectivity – from concepts to quantitative calculations and predictions. *Chem. Soc. Rev.* **45**, 6093-6107 (2016).
