# Supplementary material for: Chalcogen bond-guided conformational isomerization enables catalytic dynamic kinetic resolution of sulfoxides
Source: Nat Commun. 2022 Aug 15;13:4793. doi: 10.1038/s41467-022-32428-4 (PMC9378665; doi:10.1038/s41467-022-32428-4)
Supplement: Supplementary file 3 — Description of Additional Supplementary Files [file 41467_2022_32428_MOESM3_ESM.pdf]

Supplementary Data File 1: Cartesian coordinates of all the optimized structures
