# Supplementary material for: Chalcogen bond-guided conformational isomerization enables catalytic dynamic kinetic resolution of sulfoxides
Source: Nat Commun. 2022 Aug 15;13:4793. doi: 10.1038/s41467-022-32428-4 (PMC9378665; doi:10.1038/s41467-022-32428-4)
Supplement: Supplementary file 4 — Supplementary Data 1 [file 41467_2022_32428_MOESM4_ESM.pdf]

XI. Cartesian coordinates of all the optimized structures

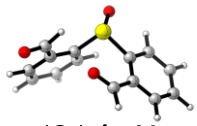

(S_p)-**1a**-M_0

O	0.19079700	2.22241100	-0.19624700
O	3.58392100	1.99862500	0.34778500
O	-0.40061400	-1.25664700	2.35179400
S	-0.14944800	-0.03780000	1.50160800
C	-0.85528200	2.12881600	-0.79514000
H	-1.14731200	2.87473100	-1.56600600
C	-1.83027800	1.04625700	-0.55746900
C	-1.61354600	0.04276100	0.40433500
C	-2.57647600	-0.93018900	0.63462400
H	-2.39331200	-1.67868400	1.40731500
C	-3.75343500	-0.93123700	-0.12149800
H	-4.50357400	-1.70361500	0.05654200
C	-3.97399800	0.04541200	-1.08984800
H	-4.89427300	0.04222400	-1.67500200
C	-3.01383200	1.03391600	-1.30097100
H	-3.17571300	1.81470800	-2.04783900
C	2.61271700	1.35837300	0.66185600
H	1.96432900	1.67744900	1.50280700
C	2.23849600	0.08178100	-0.02020100
C	3.13786500	-0.46209500	-0.94667800
H	4.05796900	0.08741400	-1.15266600
C	2.86580700	-1.67441800	-1.57044200
H	3.57430400	-2.08952500	-2.28877900
C	1.68979700	-2.36665700	-1.26760600
H	1.47843800	-3.32473800	-1.74521500
C	0.78337300	-1.83697200	-0.35250200
H	-0.13288800	-2.37555000	-0.09950200
C	1.05052300	-0.60820100	0.24753400

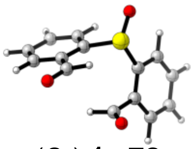

(S_p)-**1a**-TS

O	0.71113400	3.05782400	-0.03068600
O	-3.18128900	2.27418300	-0.60985400
O	0.39185900	-1.80890700	-2.09587000
S	0.16479400	-0.45356100	-1.48138700
C	0.63200300	1.99962200	0.53484500
H	-0.23529900	1.76400300	1.19450000
C	1.69873800	0.94215500	0.44422300
C	1.56654300	-0.20748200	-0.34456100
C	2.57404900	-1.16340100	-0.40517100
H	2.44143000	-2.02734400	-1.05875900
C	3.72886200	-0.98906600	0.35916400
H	4.51947600	-1.74008000	0.32250200
C	3.87568600	0.14640900	1.15270100
H	4.78263000	0.29063400	1.74158700
C	2.86688400	1.11036800	1.19182400
H	2.98057400	2.00224900	1.81151100
C	-2.31070600	1.46860000	-0.81920800
H	-1.55846100	1.64224700	-1.61941300
C	-2.18675500	0.19583700	-0.04770300
C	-3.17469700	-0.10620200	0.90058500
H	-3.98912200	0.60643500	1.04112400
C	-3.11664700	-1.28921900	1.62691300
H	-3.89192000	-1.51680300	2.35981500
C	-2.07055700	-2.19349100	1.41267000
H	-2.03093500	-3.12760200	1.97514900
C	-1.07872900	-1.90758600	0.47908400
H	-0.26426500	-2.60949200	0.28567300
C	-1.13208200	-0.70855600	-0.22873400

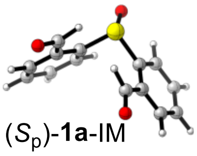

(S_p)-**1a**-IM

O	-0.99843700	3.19762400	-0.67376500
O	3.41617000	2.08630100	0.75975400
O	-0.26886900	-1.89836100	2.08016800
S	-0.07065900	-0.50727500	1.54111800
C	-0.87729400	2.18897400	-0.02763900
H	-0.08233000	2.11135400	0.74909100
C	-1.77463700	1.01150900	-0.16995900
C	-1.53332600	-0.20509800	0.48607600
C	-2.43243000	-1.26052300	0.39268200
H	-2.22532200	-2.18205400	0.93936500
C	-3.57471400	-1.11713600	-0.39787900
H	-4.28119600	-1.94521800	-0.47600100
C	-3.81515700	0.07447000	-1.08243000
H	-4.70783200	0.18081200	-1.70016200
C	-2.91909700	1.13354600	-0.96525500
H	-3.09340600	2.08251200	-1.47547400
C	2.51772300	1.30609300	0.94572200
H	1.87198200	1.39075800	1.84760800
C	2.22650500	0.17103800	0.02144200
C	3.12052700	-0.06689800	-1.03316400
H	3.96659200	0.61184600	-1.15276800
C	2.93905800	-1.15065500	-1.88278000
H	3.64249100	-1.32849700	-2.69734700
C	1.86224200	-2.02142100	-1.68553700
H	1.72558700	-2.88155800	-2.34278100
C	0.96063200	-1.79549000	-0.64971200
H	0.12134600	-2.47385600	-0.48030700
C	1.13358900	-0.69077900	0.18203700

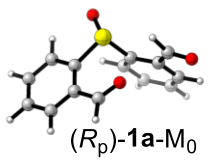

(R_p)-**1a**-M_0

O	0.19079700	2.22241100	0.19624700
O	3.58392100	1.99862500	-0.34778500
O	-0.40061400	-1.25664700	-2.35179400
S	-0.14944800	-0.03780000	-1.50160800
C	-0.85528200	2.12881600	0.79514000
H	-1.14731200	2.87473100	1.56600600
C	-1.83027800	1.04625700	0.55746900
C	-1.61354600	0.04276100	-0.40433500
C	-2.57647600	-0.93018900	-0.63462400
H	-2.39331200	-1.67868400	-1.40731500
C	-3.75343500	-0.93123700	0.12149800
H	-4.50357400	-1.70361500	-0.05654200
C	-3.97399800	0.04541200	1.08984800
H	-4.89427300	0.04222400	1.67500200
C	-3.01383200	1.03391600	1.30097100
H	-3.17571300	1.81470800	2.04783900
C	2.61271700	1.35837300	-0.66185600
H	1.96432900	1.67744900	-1.50280700
C	2.23849600	0.08178100	0.02020100
C	3.13786500	-0.46209500	0.94667800
H	4.05796900	0.08741400	1.15266600
C	2.86580700	-1.67441800	1.57044200
H	3.57430400	-2.08952500	2.28877900
C	1.68979700	-2.36665700	1.26760600
H	1.47843800	-3.32473800	1.74521500
C	0.78337300	-1.83697200	0.35250200
H	-0.13288800	-2.37555000	0.09950200
C	1.05052300	-0.60820100	-0.24753400

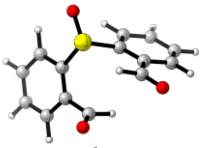

(R_p)-**1a**-TS

O	0.71113400	3.05782400	0.03068600
O	-3.18128900	2.27418300	0.60985400
O	0.39185900	-1.80890700	2.09587000
S	0.16479400	-0.45356100	1.48138700
C	0.63200300	1.99962200	-0.53484500
H	-0.23529900	1.76400300	-1.19450000
C	1.69873800	0.94215500	-0.44422300
C	1.56654300	-0.20748200	0.34456100
C	2.57404900	-1.16340100	0.40517100
H	2.44143000	-2.02734400	1.05875900
C	3.72886200	-0.98906600	-0.35916400
H	4.51947600	-1.74008000	-0.32250200
C	3.87568600	0.14640900	-1.15270100
H	4.78263000	0.29063400	-1.74158700
C	2.86688400	1.11036800	-1.19182400
H	2.98057400	2.00224900	-1.81151100
C	-2.31070600	1.46860000	0.81920800
H	-1.55846100	1.64224700	1.61941300
C	-2.18675500	0.19583700	0.04770300
C	-3.17469700	-0.10620200	-0.90058500
H	-3.98912200	0.60643500	-1.04112400
C	-3.11664700	-1.28921900	-1.62691300
H	-3.89192000	-1.51680300	-2.35981500
C	-2.07055700	-2.19349100	-1.41267000
H	-2.03093500	-3.12760200	-1.97514900
C	-1.07872900	-1.90758600	-0.47908400
H	-0.26426500	-2.60949200	-0.28567300
C	-1.13208200	-0.70855600	0.22873400

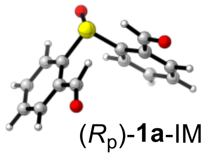

(R_p)-**1a**-IM

O	-0.99843700	3.19762400	0.67376500
O	3.41617000	2.08630100	-0.75975400
O	-0.26886900	-1.89836100	-2.08016800
S	-0.07065900	-0.50727500	-1.54111800
C	-0.87729400	2.18897400	0.02763900
H	-0.08233000	2.11135400	-0.74909100
C	-1.77463700	1.01150900	0.16995900
C	-1.53332600	-0.20509800	-0.48607600
C	-2.43243000	-1.26052300	-0.39268200
H	-2.22532200	-2.18205400	-0.93936500
C	-3.57471400	-1.11713600	0.39787900
H	-4.28119600	-1.94521800	0.47600100
C	-3.81515700	0.07447000	1.08243000
H	-4.70783200	0.18081200	1.70016200
C	-2.91909700	1.13354600	0.96525500
H	-3.09340600	2.08251200	1.47547400
C	2.51772300	1.30609300	-0.94572200
H	1.87198200	1.39075800	-1.84760800
C	2.22650500	0.17103800	-0.02144200
C	3.12052700	-0.06689800	1.03316400
H	3.96659200	0.61184600	1.15276800
C	2.93905800	-1.15065500	1.88278000
H	3.64249100	-1.32849700	2.69734700
C	1.86224200	-2.02142100	1.68553700
H	1.72558700	-2.88155800	2.34278100
C	0.96063200	-1.79549000	0.64971200
H	0.12134600	-2.47385600	0.48030700
C	1.13358900	-0.69077900	-0.18203700

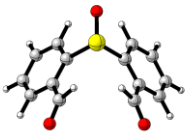

INT-**1a**-TS

O	-3.01827900	-0.09994242	2.16042700
O	-3.01827900	-0.09994242	-2.16042700
O	1.79668176	2.24292506	0.00000000
S	0.43483883	1.60314192	0.00000000
C	-2.01872206	0.40392468	1.71706300
H	-2.07622515	1.32343567	1.09294900
C	-0.64951000	-0.10956618	1.99874400
C	0.49654195	0.37894993	1.35725100
C	1.76753899	-0.03314794	1.74592900
H	2.64045094	0.40875614	1.26248000
C	1.89826808	-0.98824293	2.75236000
H	2.89282112	-1.31618683	3.05762900
C	0.76437914	-1.52156304	3.37106600
H	0.87167521	-2.27112003	4.15563500
C	-0.49977991	-1.07864017	3.00002400
H	-1.40022487	-1.45246726	3.48953600
C	-2.01872206	0.40392468	-1.71706300
H	-2.07622515	1.32343567	-1.09294900
C	-0.64951000	-0.10956618	-1.99874400
C	-0.49977991	-1.07864017	-3.00002400
H	-1.40022487	-1.45246726	-3.48953600
C	0.76437914	-1.52156304	-3.37106600
H	0.87167521	-2.27112003	-4.15563500
C	1.89826808	-0.98824293	-2.75236000
H	2.89282112	-1.31618683	-3.05762900
C	1.76753899	-0.03314794	-1.74592900
H	2.64045094	0.40875614	-1.26248000
C	0.49654195	0.37894993	-1.35725100

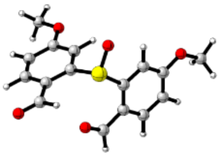

1c-M_0

S	0.00000000	0.00000000	0.00000000
O	0.00000000	0.00000000	5.05374885
O	4.94782179	0.00000000	0.85858357
O	-0.64455404	2.78450782	-0.35271646
O	0.44248717	-1.41523790	0.22588384
O	-3.96552552	1.92682963	0.47439259
C	-0.54241755	0.21285640	6.33241320
H	-0.71193500	1.28491941	6.52429253
H	0.19081534	-0.16931951	7.05036323
H	-1.49234078	-0.33079535	6.46129991
C	-0.71524516	0.37467080	3.97847894
C	-0.13591929	0.11711511	2.72871915
H	0.83224308	-0.38554169	2.68478939
C	-0.80148335	0.48794159	1.57303391
C	1.54142119	0.97299823	0.23589954
C	2.66171476	0.20359502	0.46439111
H	2.57199923	-0.88342500	0.51917932
C	3.91693666	0.82239019	0.62684481
C	6.23245031	0.54839058	1.02492032
H	6.55451426	1.09214419	0.12234772
H	6.90995773	-0.29345492	1.19998539
H	6.26731504	1.22900169	1.89072309
C	-1.97550433	0.98839203	4.04552731
H	-2.44726832	1.19622666	5.00453220
C	-2.63789928	1.32073133	2.86961175
H	-3.62836642	1.77857345	2.89439602
C	-2.07592227	1.07191066	1.61450717
C	1.63483019	2.37632296	0.12564996
C	2.88653096	2.97085337	0.28522982
H	2.96825068	4.05765919	0.20333606
C	4.02914514	2.21586781	0.53839551
H	4.98980185	2.71332098	0.65626567
C	0.46795939	3.21716024	-0.17666789
H	0.67533267	4.31078946	-0.24693913
C	-2.88663794	1.39876094	0.40456594
H	-2.45037105	1.10371963	-0.57201264

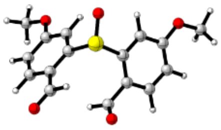

1c-TS

S	0.00000000	0.00000000	0.00000000
O	0.00000000	0.00000000	5.04250133
O	5.02780634	0.00000000	0.19477938
O	-0.06549230	3.57967840	-1.32181859
O	0.40894870	-1.41193571	0.29408292
O	-3.42165105	2.78374180	0.46249124
C	-0.46224759	0.34553030	6.32533326
H	-0.36750904	1.42810704	6.50754856
H	0.16707454	-0.19612895	7.03886353
H	-1.51257442	0.04439390	6.46688435
C	-0.60966179	0.52459558	3.96973559
C	-0.11638860	0.12781598	2.71897760
H	0.70159423	-0.59315162	2.65922101
C	-0.68735970	0.63941302	1.56741928
C	1.55797244	0.95275760	0.03555641
C	2.70324759	0.18644672	0.13665078
H	2.62599973	-0.89604987	0.25452511
C	3.96051311	0.80863444	0.07294213
C	6.31176565	0.56418516	0.11494763
H	6.47824177	1.04732280	-0.86172501
H	7.02333890	-0.25943332	0.23453401
H	6.47639670	1.30370761	0.91571699
C	-1.68970126	1.42193415	4.03884740
H	-2.09088868	1.74035954	4.99957576
C	-2.26515096	1.89570996	2.86721383
H	-3.11938696	2.57453003	2.89704699
C	-1.78826463	1.50991990	1.60911668
C	1.60761995	2.34164074	-0.15718351
C	2.86282100	2.93900843	-0.23586595
H	2.93208492	4.01740537	-0.39285779
C	4.03697784	2.19129979	-0.12008691
H	4.99800452	2.69783129	-0.18974614
C	0.37845447	3.20160211	-0.27330841
H	-0.10133653	3.50057400	0.69006220
C	-2.49453613	2.01976554	0.39990012
H	-2.12462315	1.64895370	-0.58247050

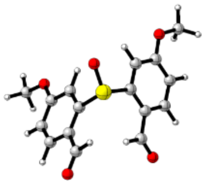

1c-IM

S	0.00000000	0.00000000	0.00000000
O	0.00000000	0.00000000	5.04845975
O	4.95326560	0.00000000	0.84585523
O	0.53030963	4.40579400	-0.40818316
O	0.40848021	-1.42229661	0.23701893
O	-3.70589104	2.38403597	0.46226797
C	-0.48406306	0.31471886	6.33145304
H	-0.48842617	1.40322166	6.50200091
H	0.19713635	-0.15923299	7.04550978
H	-1.50109104	-0.08070644	6.48345935
C	-0.66022547	0.45913845	3.97580906
C	-0.13330175	0.10710689	2.72528425
H	0.75725397	-0.52265285	2.67456835
C	-0.75167454	0.55561476	1.57214027
C	1.55244193	0.95860201	0.17782605
C	2.66811189	0.18883647	0.43800561
H	2.57800748	-0.89522918	0.52888650
C	3.91963844	0.81276985	0.58667031
C	6.23458573	0.55867065	1.00312624
H	6.55938618	1.07855416	0.08759606
H	6.91483740	-0.27498800	1.20514276
H	6.26262252	1.26356649	1.84950863
C	-1.81960480	1.25086388	4.04430959
H	-2.24538986	1.53704520	5.00451457
C	-2.44090885	1.65933363	2.87214167
H	-3.35775284	2.25076818	2.90076192
C	-1.93329778	1.31225916	1.61466607
C	1.63802936	2.35348089	0.02209492
C	2.88898546	2.95391120	0.17796686
H	2.94674981	4.03761615	0.06060286
C	4.02686611	2.20520863	0.45959493
H	4.98665523	2.70693154	0.56874347
C	0.47760080	3.20759529	-0.32371207
H	-0.47680825	2.66752631	-0.53040028
C	-2.71039020	1.71120238	0.41020862
H	-2.33305548	1.32763760	-0.56619042

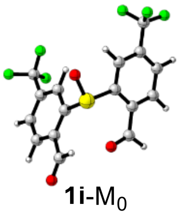

1i-M_0

F	0.00000000	0.00000000	0.00000000
F	0.00000000	0.00000000	2.14444375
F	1.86572365	0.00000000	1.07243366
O	-0.54160480	6.89192187	1.03287371
O	-3.07661740	8.88637940	2.24947508
O	-3.26001340	3.50796221	0.97916838
S	-2.37336077	4.72396716	0.97125471
C	0.48264671	6.25348735	1.03971455
H	1.47582460	6.75070228	1.01576469
C	0.50357564	4.77348968	1.07044977
C	-0.68369089	4.01848540	1.06500498
C	-0.63414799	2.63465659	1.04661899
H	-1.57067320	2.07239515	1.01156561
C	0.61042296	1.99549463	1.07300863
C	1.79474283	2.72224965	1.09593351
H	2.75536404	2.20876988	1.11199452
C	1.73353218	4.11590259	1.08889012
H	2.65318105	4.70407303	1.09491274
C	0.62697782	0.48727222	1.07202515
C	-2.93806327	7.72630388	1.96277956
H	-2.99182647	7.37860424	0.91205825
C	-2.72182016	6.66926001	3.00416042
C	-2.45090283	5.33461310	2.69233784
C	-2.34985431	4.36938067	3.69152056
H	-2.18703800	3.32187810	3.42908401
C	-2.47039866	4.75846598	5.02023227
C	-2.70927907	6.09491000	5.35748422
H	-2.80803156	6.38520212	6.40450639
C	-2.83817212	7.04020734	4.35036695
H	-3.04625014	8.08601844	4.57901144
C	-2.31824987	3.74613486	6.12626361
F	-1.14559690	3.88542902	6.75092429
F	-3.26883740	3.89108325	7.05102027
F	-2.38081250	2.49586645	5.67172780

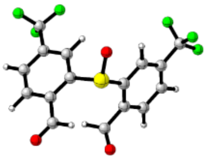

1i-TS

F	0.00000000	0.00000000	0.00000000
F	0.00000000	0.00000000	2.14390684
F	1.86574690	0.00000000	1.07408496
O	0.89903416	6.96852303	0.22467460
O	-2.28734113	9.11486176	1.38189759
O	-3.22680531	3.62165313	0.57954039
S	-2.26920590	4.78026030	0.58947988
C	0.52434938	6.29137637	1.14253799
H	0.18051166	6.75220233	2.09769475
C	0.52526579	4.78547157	1.10062968
C	-0.64609395	4.03106415	0.94664088
C	-0.61231772	2.64540545	0.91601731
H	-1.54354635	2.09514216	0.76184852
C	0.61425174	1.99623260	1.07210396
C	1.79010885	2.72234295	1.22389804
H	2.74377534	2.20546686	1.32809085
C	1.74257647	4.11730404	1.23282901
H	2.66224115	4.69252798	1.34898765
C	0.62720186	0.48956143	1.07132529
C	-2.31801946	7.92330471	1.22435152
H	-2.18401987	7.47431455	0.21642098
C	-2.54422679	6.97164900	2.35941248
C	-2.51793429	5.58225184	2.20634947
C	-2.78448133	4.73078123	3.27718934
H	-2.80318032	3.65009349	3.12147927
C	-3.03495127	5.28400848	4.52555919
C	-3.03722539	6.67244995	4.71068188
H	-3.23485950	7.09191486	5.69848175
C	-2.79731343	7.50794750	3.63081500
H	-2.80844619	8.59286469	3.74051938
C	-3.31539438	4.39982788	5.71474908
F	-2.42917000	4.61355861	6.68953878
F	-4.52242185	4.64877587	6.22552298
F	-3.26736510	3.10780949	5.40032608

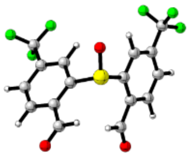

1i-IM

F	0.00000000	0.00000000	0.00000000
F	0.00000000	0.00000000	2.14443650
F	1.86566820	0.00000000	1.07439463
O	1.50351050	6.92955979	1.28004075
O	-2.89230754	8.92646076	1.97625340
O	-3.21875717	3.57234888	0.51609942
S	-2.30797905	4.75882062	0.66136107
C	0.52174780	6.26901661	1.07300457
H	-0.45003660	6.75646215	0.83087214
C	0.51931467	4.77792629	1.09529921
C	-0.65268595	4.02554363	0.92472338
C	-0.61368589	2.64020687	0.89305797
H	-1.53966857	2.08422387	0.72805305
C	0.61261343	1.99579675	1.07354571
C	1.78572009	2.72016368	1.26638117
H	2.73421348	2.20090757	1.40038446
C	1.73328327	4.11203742	1.27347500
H	2.63560208	4.70981378	1.40787353
C	0.62696056	0.48766943	1.07154187
C	-2.77825059	7.76755754	1.67768459
H	-2.70766654	7.45531318	0.61258348
C	-2.75094799	6.67370788	2.70046892
C	-2.55150209	5.32832881	2.37813805
C	-2.63033002	4.33440877	3.35263484
H	-2.51535134	3.28416843	3.07646123
C	-2.86134874	4.70315802	4.67069729
C	-3.02636380	6.04835524	5.02356896
H	-3.20440526	6.32303004	6.06444931
C	-2.97572397	7.02378455	4.04055923
H	-3.12370304	8.07734231	4.27985812
C	-2.94146154	3.66002203	5.75705626
F	-2.01330579	3.87005083	6.69259578
F	-4.12589818	3.69479012	6.37000899
F	-2.76687529	2.42888733	5.28312068

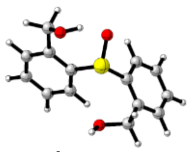

1ac-M_0

S	0.00000000	0.00000000	0.00000000
O	0.00000000	0.00000000	1.52973454
O	0.43902284	0.00000000	-2.85982400
H	0.40401232	-0.89193773	-3.22595538
O	-1.73508135	-1.97296768	1.22882443
H	-0.93224008	-1.57901158	1.61468916
C	0.96307858	1.46610646	-0.42761824
C	-1.66841490	0.56443846	-0.47294011
C	1.75998873	1.48302716	-1.57801374
C	-2.77978235	0.01289073	0.18963143
C	0.96199129	2.51509525	0.48719556
H	0.35394061	2.42842291	1.38936956
C	-1.83041794	1.49334230	-1.50356988
H	-0.95836690	1.88513630	-2.02433192
C	2.54558015	2.61577034	-1.80833750
H	3.16784805	2.65721736	-2.70446534
C	1.75229618	3.63509023	0.23848621
H	1.75455847	4.46734141	0.94335465
C	2.54282291	3.68313500	-0.91046122
H	3.16351481	4.55776973	-1.10942086
C	-4.21976123	1.39281482	-1.22196988
H	-5.22396934	1.71112160	-1.50517400
C	-3.10490925	1.91201200	-1.87637287
H	-3.22083042	2.64162460	-2.67859706
C	1.77194271	0.30843270	-2.52338253
H	2.26007332	-0.54775440	-2.02362237
H	2.37222586	0.56267395	-3.41233194
C	-2.71809615	-0.98173614	1.34187058
H	-3.69811332	-1.47875156	1.39129201
H	-2.60041268	-0.41082027	2.27912476
C	-4.04849567	0.45372862	-0.20920759
H	-4.92464639	0.03676309	0.29280496

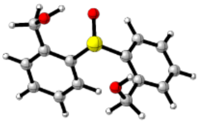

1ac-TS

S	0.00000000	0.00000000	0.00000000
O	0.00000000	0.00000000	1.52861599
O	1.53443602	0.00000000	-2.55617993
H	2.40556459	0.25352025	-2.22589599
O	-0.57258316	-2.57778071	1.07954399
H	-0.07613617	-1.88204458	1.54353925
C	0.23271264	1.73701707	-0.45838007
C	-1.75006134	-0.21527631	-0.46934540
C	0.63576668	2.13125059	-1.74530147
C	-2.45305775	-1.29562353	0.09762414
C	0.05300467	2.65877123	0.56967466
H	-0.23093939	2.28848072	1.55572780
C	-2.36523086	0.63182744	-1.39417644
H	-1.82699173	1.48411096	-1.80787505
C	0.82634719	3.49975761	-1.96521968
H	1.14404254	3.83321991	-2.95544449
C	0.24675154	4.01620182	0.32170848
H	0.10305350	4.74163740	1.12332414
C	0.63098003	4.43602816	-0.95035616
H	0.79206468	5.49552929	-1.15348733
C	-4.39615364	-0.65055108	-1.23595868
H	-5.43017681	-0.83361491	-1.53082056
C	-3.68675222	0.41601000	-1.78011694
H	-4.15524112	1.08701673	-2.50074980
C	0.83025460	1.16342714	-2.89714792
H	1.31068469	1.70943479	-3.72663735
H	-0.15206063	0.82767162	-3.26764017
C	-1.92164625	-2.21932400	1.18646195
H	-2.52145804	-3.14054123	1.15092361
H	-2.12975854	-1.74099699	2.15954231
C	-3.77886705	-1.48642625	-0.30879435
H	-4.33859565	-2.32111532	0.11944043

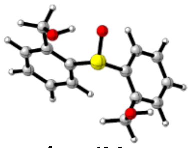

1ac-IM

S	0.00000000	0.00000000	0.00000000
O	0.00000000	0.00000000	1.52747620
O	2.47154785	0.00000000	-1.99877699
H	3.24913498	0.52998846	-1.78421523
O	-1.03297586	-2.42990005	1.20829306
H	-0.39586648	-1.81605439	1.61271758
C	0.42421181	1.69472761	-0.44929958
C	-1.75264424	-0.00624582	-0.50397913
C	1.08815090	1.96124898	-1.65340841
C	-2.63753235	-0.89903230	0.12863772
C	0.10313452	2.69478066	0.46198613
H	-0.38649186	2.41997514	1.39794003
C	-2.17845355	0.79896181	-1.56358481
H	-1.48689887	1.48991403	-2.04708706
C	1.39916440	3.29466171	-1.93501891
H	1.91716566	3.53302449	-2.86651514
C	0.42440473	4.01710284	0.15897418
H	0.17522712	4.81274058	0.86205198
C	1.06612437	4.31521852	-1.04289881
H	1.31998009	5.34820574	-1.28430600
C	-4.39621971	-0.11831073	-1.37733687
H	-5.43407170	-0.17007992	-1.70873510
C	-3.50015791	0.74509507	-2.00140818
H	-3.82268962	1.38190186	-2.82571714
C	1.51406151	0.84517577	-2.58654832
H	1.87462803	1.28926272	-3.52959106
H	0.65262966	0.20771338	-2.83918241
C	-2.27767093	-1.79414408	1.30672833
H	-3.05407433	-2.57033222	1.37341226
H	-2.34395884	-1.19192807	2.22873956
C	-3.95998356	-0.92567213	-0.32959692
H	-4.66252626	-1.61021221	0.15113518

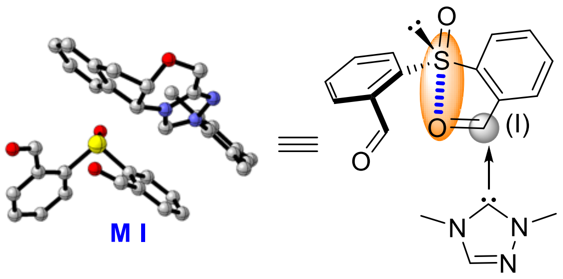

C	-2.69635200	0.77938900	-4.31540300
C	-2.46653600	0.46100500	-2.87053400
C	-0.06997500	-0.02067900	-3.31859100
C	-0.47173900	0.17296000	-4.80313100
H	-3.12473800	-0.10307600	-4.82582100
H	-3.40259300	1.61195500	-4.41269600
H	0.41072200	-1.00186700	-3.18100600
H	-0.82007900	-0.76771700	-5.26307000
O	-1.48063000	1.15896700	-4.90795700
N	-3.29516500	0.49359000	-1.87585400
N	-1.23273300	0.03820900	-2.44901200
C	0.78907000	0.74525200	-5.45596000
H	1.44434300	-0.07036800	-5.79925600
H	0.52451400	1.36120200	-6.32568800
C	0.93309000	1.09085600	-3.09874700
C	1.36297100	1.65054300	-1.90168900
C	1.43527400	1.51987900	-4.33009600
C	2.32134000	2.66748200	-1.95270900
H	0.95917100	1.29200200	-0.95181300
C	2.39479600	2.52715900	-4.37800300
C	2.83385000	3.09820700	-3.17951700
H	2.79166900	2.87469000	-5.33370100
H	3.57961900	3.89442100	-3.20276300
C	-1.24795700	-0.21158600	-1.10790700
N	-2.52875900	0.07759900	-0.81617600
C	-3.13313700	0.02739400	0.48162200
C	-4.02242100	-1.02418500	0.75774400
C	-2.86688600	1.04003500	1.40854100
C	-4.65930400	-1.03764500	1.99645400
C	-3.52905900	0.98141300	2.64234700
C	-4.42540300	-0.04044600	2.95215500
H	-5.35418600	-1.85002800	2.22575400
H	-3.33701700	1.76770900	3.37719000
C	-1.89941300	2.15671600	1.12238300
H	-2.23309800	3.08329100	1.60825900
H	-0.90376100	1.92108700	1.53231200

H	-1.78876700	2.34292900	0.04662600
C	-4.25526800	-2.10392900	-0.26275900
H	-4.72757400	-1.69495600	-1.16784800
H	-3.29741500	-2.55026800	-0.57298500
H	-4.89778500	-2.89500400	0.14310800
C	-5.11221200	-0.09632300	4.29153700
H	-4.98188100	0.84098800	4.84732400
H	-6.18855000	-0.28652400	4.17668200
H	-4.70210400	-0.91354400	4.90523800
H	2.67075000	3.12931300	-1.02713000
O	1.82707700	-1.33847300	-0.33536000
O	5.07846700	-0.17143500	-0.69001800
O	1.22880900	1.50450900	2.88577500
S	1.48526800	0.52471200	1.76180600
C	0.82942700	-1.97701900	-0.09391400
H	0.43215600	-2.72353000	-0.81620700
C	0.07253800	-1.87008300	1.17182400
C	0.29419600	-0.81880800	2.07250900
C	-0.48714100	-0.68602000	3.21210100
H	-0.32818700	0.17635600	3.86185500
C	-1.47047700	-1.64047400	3.48774700
H	-2.08325600	-1.53625000	4.38450100
C	-1.68430300	-2.70468500	2.61549100
H	-2.46144700	-3.44000100	2.82762300
C	-0.91839000	-2.81036400	1.45477300
H	-1.09368800	-3.62455800	0.74739700
C	4.14819500	0.02872300	0.04986900
H	3.28576300	0.64550300	-0.26920100
C	4.10536000	-0.50311500	1.44650000
C	5.24798300	-1.14172400	1.94551800
H	6.10407200	-1.25075400	1.27807300
C	5.28625800	-1.60549400	3.25524800
H	6.18165200	-2.09970200	3.63398200
C	4.17908400	-1.42727300	4.08976900
H	4.20806800	-1.77846600	5.12206800
C	3.03391000	-0.79934000	3.60661300
H	2.16643800	-0.64728100	4.25212700
C	2.99433200	-0.35988300	2.28490100

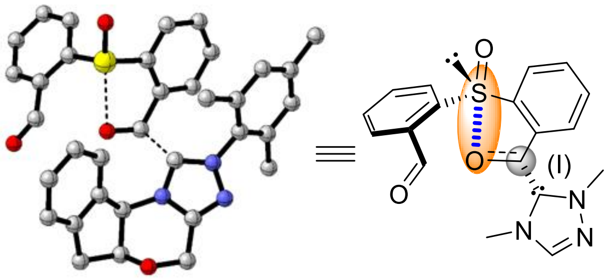

TS I

C	-0.30418000	0.36426000	-0.04067300
C	-0.23948300	0.15770500	1.44162300
C	2.23394000	-0.08713600	1.33142000
C	2.01845800	-0.04277800	-0.20006900
H	-0.58960900	-0.58778700	-0.52512700
H	-1.06270600	1.11717700	-0.28350900
H	2.82941700	-0.96226100	1.62928200
H	1.83946500	-1.04370200	-0.62775500
O	0.93738700	0.81744100	-0.51028400
N	-1.19514900	0.16490600	2.31800600
N	0.95870000	-0.13970200	2.03804800
C	3.28997600	0.63571500	-0.71834000
H	4.08417000	-0.11153600	-0.87043700
H	3.09672600	1.13401800	-1.67738600
C	3.01141200	1.18355100	1.58448900
C	3.13567500	1.90432100	2.76325000
C	3.64344100	1.58461900	0.40522700
C	3.93367700	3.05216500	2.75682200
H	2.63814500	1.56325000	3.67343800
C	4.44425200	2.72259900	0.40109700
C	4.58585600	3.45153100	1.58697800
H	4.94420100	3.05061700	-0.51206900
H	5.20567200	4.34955700	1.59586200
C	0.75797600	-0.33253300	3.36249800
N	-0.55969900	-0.14032000	3.48891700
C	-1.28811100	-0.15589900	4.72238900
C	-2.20614400	-1.18800900	4.94689700
C	-1.03507000	0.85362700	5.66050700
C	-2.87038600	-1.20625800	6.17480100
C	-1.72266400	0.78823700	6.87411100
C	-2.63196400	-0.23604200	7.15301200
H	-3.58465400	-2.00850200	6.37596600
H	-1.53838400	1.56093500	7.62484900
C	-0.06245800	1.96883600	5.38033000
H	-0.25054500	2.81711300	6.04998800

H	0.97529200	1.63922400	5.54562100
H	-0.13559600	2.31798300	4.34056300
C	-2.44317800	-2.24876200	3.90628000
H	-2.93294300	-1.82555800	3.01758400
H	-1.49187000	-2.68877700	3.56850000
H	-3.07530100	-3.05063500	4.30630600
C	-3.30497500	-0.31161100	8.49726100
H	-3.55275000	0.68911900	8.87582600
H	-4.22465900	-0.90908000	8.45339900
H	-2.63263600	-0.78347300	9.23163600
H	4.05081100	3.63544700	3.67187200
O	3.33378400	-0.95193300	4.02572300
O	6.44392800	-0.52543500	2.98565500
O	3.84215900	1.67231100	7.39062400
S	3.70100400	0.77160800	6.18428800
C	2.22114700	-1.39429600	4.35230500
H	1.80463600	-2.29783800	3.84977800
C	1.75376300	-1.27590100	5.77703600
C	2.34947600	-0.36949800	6.66037400
C	1.88252000	-0.22789400	7.96509000
H	2.34072200	0.52886900	8.60373100
C	0.84922900	-1.04713700	8.42059000
H	0.48937300	-0.94428000	9.44571500
C	0.27807400	-1.99077600	7.56734700
H	-0.52899200	-2.63465300	7.92022700
C	0.72651700	-2.09391100	6.25226500
H	0.26840500	-2.81569800	5.57047300
C	5.80350100	-0.13474100	3.93029300
H	5.09841300	0.71232200	3.83607100
C	5.94947600	-0.72107900	5.29814000
C	7.01823500	-1.59383100	5.53506500
H	7.67489700	-1.83536700	4.69776200
C	7.23815200	-2.11926200	6.80385600
H	8.07521300	-2.79654200	6.97784100
C	6.39142500	-1.76744000	7.85829200
H	6.56638900	-2.16571500	8.85873100
C	5.32075800	-0.90402200	7.63619100
H	4.65827200	-0.61173000	8.45356900
C	5.09088100	-0.40521500	6.35632700

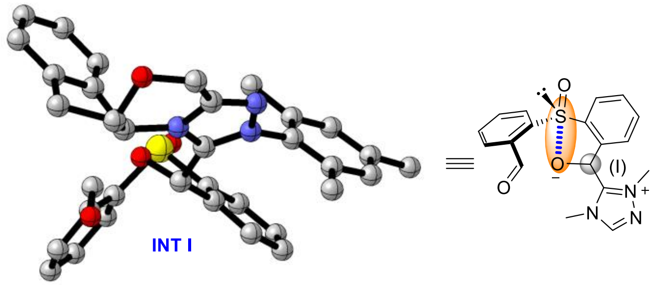

C	-2.07831800	0.48246100	-4.24822100
C	-1.96660700	0.32988400	-2.75976200
C	0.49596700	0.03042700	-2.92372600
C	0.20573700	-0.17039200	-4.42608500
H	-2.54358400	-0.44167200	-4.63885300
H	-2.73559400	1.32572700	-4.49164500
H	1.14787500	-0.76440500	-2.51080700
H	-0.05316800	-1.21291100	-4.67575800
O	-0.82930200	0.71342100	-4.82900500
N	-2.92341500	0.30870500	-1.88299800
N	-0.76971900	0.01109600	-2.18207000
C	1.48233000	0.34530200	-5.08637600
H	2.25665000	-0.43723100	-5.07595900
H	1.29437500	0.63869100	-6.12724200
C	1.23870300	1.35749100	-2.93367600
C	1.37923800	2.33018900	-1.95037400
C	1.85209900	1.50154400	-4.18461700
C	2.16303900	3.45383300	-2.23226100
H	0.90576100	2.21039200	-0.97606200
C	2.64241000	2.61240400	-4.45701700
C	2.79597800	3.58977700	-3.46905200
H	3.12044400	2.72860600	-5.43142100
H	3.40580700	4.47183500	-3.67078700
C	-1.01493400	-0.27385700	-0.88825400
N	-2.32213400	-0.05414100	-0.72219500
C	-3.10807700	-0.12498200	0.48101200
C	-4.08978900	-1.12025200	0.55977400
C	-2.85363700	0.78819200	1.51300100
C	-4.81541300	-1.20959700	1.74839700
C	-3.59763900	0.64093200	2.68463700
C	-4.57075300	-0.35297200	2.82529500
H	-5.57871700	-1.98558400	1.84095900
H	-3.40350600	1.32334000	3.51571000
C	-1.85172700	1.90618400	1.38601600
H	-1.50916000	2.22201300	2.38068800
H	-0.96169300	1.62782700	0.80562100

H	-2.31080900	2.77596200	0.89075500
C	-4.33908200	-2.07475200	-0.57686000
H	-4.82231600	-1.56243700	-1.42086700
H	-3.40055500	-2.50651500	-0.95702800
H	-4.98790100	-2.89604400	-0.25047300
C	-5.30408900	-0.51874000	4.12825500
H	-5.54381200	0.45453100	4.57683600
H	-6.23415800	-1.08588200	3.99622600
H	-4.67397700	-1.06584500	4.84726500
H	2.28207000	4.22977500	-1.47470200
O	1.10949500	0.09023100	-0.01520700
O	2.36136800	-1.61225100	-1.09383000
O	1.46349600	1.51346700	3.80748100
S	1.36199800	0.98244500	2.39520000
C	0.06633500	-0.80696000	0.03394400
H	0.40652500	-1.74975400	-0.45449700
C	-0.36181000	-1.11913900	1.45423900
C	0.12456100	-0.37706000	2.53602000
C	-0.30775800	-0.63949300	3.83340100
H	0.08418500	-0.02206300	4.64283500
C	-1.21387000	-1.67216000	4.06793100
H	-1.55254900	-1.87612800	5.08493600
C	-1.67182900	-2.45097600	3.00551700
H	-2.36834100	-3.27169900	3.18243700
C	-1.23710500	-2.17742500	1.71121500
H	-1.59778200	-2.78481500	0.87682600
C	2.48400700	-0.57591900	-0.34521000
H	2.96660300	0.32847900	-0.79550900
C	3.13779700	-0.80603400	1.02870300
C	4.20541700	-1.70827100	1.06847000
H	4.44431600	-2.23944200	0.14576300
C	4.93372800	-1.92453000	2.23685100
H	5.76582100	-2.63068500	2.23793600
C	4.59802100	-1.23613200	3.40260000
H	5.16612600	-1.39300700	4.32085700
C	3.52108600	-0.35112900	3.39522200
H	3.21751900	0.19223500	4.29145000
C	2.79750900	-0.15465700	2.22071600

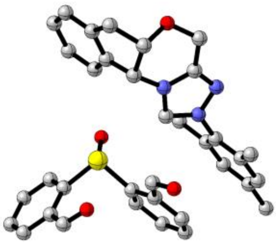

M II

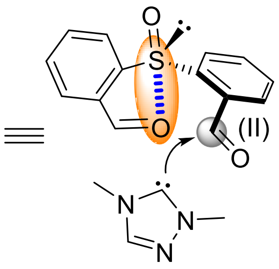

O	-1.08925000	2.50857700	2.74252800
O	-0.33675300	-0.98948500	3.26034200
O	-0.64978700	1.65557200	-1.53548900
S	-0.73951200	1.88293500	-0.03879300
C	-1.73178000	3.51919800	2.58779900
H	-2.07216100	4.12307300	3.45789700
C	-2.11243700	4.04986200	1.26371300
C	-1.74871700	3.41133000	0.06332700
C	-2.09284900	3.97846100	-1.15717700
H	-1.77716200	3.47754700	-2.07285100
C	-2.82822300	5.16767900	-1.19134200
H	-3.10004600	5.60113800	-2.15489200
C	-3.20840700	5.80001600	-0.01031000
H	-3.77956800	6.72804000	-0.04042200
C	-2.84468100	5.23979100	1.21268500
H	-3.12473200	5.72773900	2.14895800
C	-0.52728000	-0.18430300	2.37962800
H	0.21638300	0.59262500	2.13495200
C	-1.79282400	-0.16788100	1.57945300
C	-2.78708400	-1.09933200	1.89613500
H	-2.59625300	-1.79041400	2.71870400
C	-3.97211400	-1.14809500	1.17024900
H	-4.73764300	-1.88187300	1.42714700
C	-4.17251200	-0.27261400	0.10067900
H	-5.09399600	-0.31817000	-0.48192400
C	-3.18963100	0.65695700	-0.23191700
H	-3.32435200	1.33496800	-1.07753200
C	-2.01966400	0.71745800	0.52096900
C	4.20847900	-2.80121600	-0.10247800
C	2.75594900	-2.43625500	-0.12699700
C	3.28712800	-0.07466200	0.42120600
C	4.71293600	-0.63180400	0.65975600
H	4.44447200	-3.29358000	0.85923600
H	4.43759000	-3.50257600	-0.91349000
H	2.93222500	0.44207700	1.32703600
H	4.83934300	-1.02267800	1.68404200

O	4.99156200	-1.65035200	-0.28127100
N	1.72925200	-3.19980800	-0.31991300
N	2.35325800	-1.15208400	0.13724900
C	5.63021000	0.54729500	0.33287900
H	5.77870900	1.17337700	1.22653400
H	6.61269900	0.18869000	-0.00173700
C	3.50719900	0.92303700	-0.69678100
C	2.58056700	1.47368800	-1.57428500
C	4.85542500	1.29390500	-0.72873200
C	3.03190700	2.41830200	-2.50207200
H	1.52550400	1.19012400	-1.54440900
C	5.29976400	2.23550800	-1.65198200
C	4.37651300	2.79569000	-2.54051500
H	6.35130200	2.52667800	-1.68940700
H	4.71128900	3.53109500	-3.27412900
C	0.99023200	-1.06728500	0.11116900
N	0.66816900	-2.34581100	-0.16413100
C	-0.65656400	-2.88301500	-0.25548100
C	-1.08148500	-3.78011800	0.73269400
C	-1.47159400	-2.51753300	-1.33662700
C	-2.36540800	-4.32177700	0.61564900
C	-2.74741500	-3.08062200	-1.40411300
C	-3.21120000	-3.98308800	-0.44202900
H	-2.71397600	-5.01987900	1.38109700
H	-3.39834300	-2.80612000	-2.23883600
C	-0.98697600	-1.55833200	-2.38761300
H	-1.65476400	-1.57614700	-3.25876900
H	-0.94451100	-0.52923200	-1.99730800
H	0.02944200	-1.81884200	-2.71869900
C	-0.18938500	-4.13646800	1.89174900
H	0.66523900	-4.74284700	1.55905400
H	0.21171000	-3.22750100	2.36403800
H	-0.74861700	-4.70340800	2.64677000
C	-4.60267600	-4.55046000	-0.54479800
H	-5.35522000	-3.75104600	-0.46138300
H	-4.75889200	-5.04088500	-1.51654300
H	-4.79571800	-5.28517700	0.24738700
H	2.32218500	2.86343700	-3.20144500

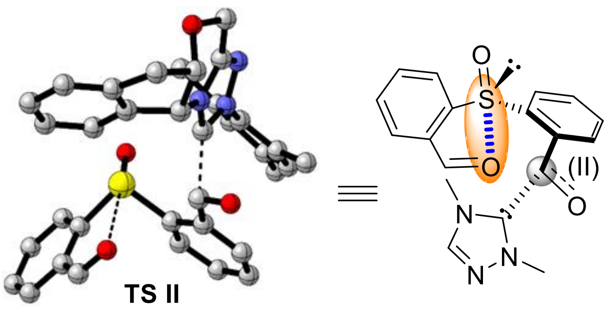

O	-0.22274100	2.81681800	1.62132600
O	1.02231800	-0.69916500	2.74776600
O	-1.65459800	0.58960500	-1.85961600
S	-1.11840100	1.29209100	-0.63384900
C	-0.97082100	3.75349300	1.48043500
H	-0.93780100	4.62632300	2.16971000
C	-1.97032400	3.84060700	0.39680600
C	-2.12092300	2.82559400	-0.56533200
C	-3.03287300	2.97776600	-1.60177000
H	-3.10855600	2.18622700	-2.34839400
C	-3.82845400	4.12625900	-1.66625600
H	-4.54919000	4.23364600	-2.47820400
C	-3.70370800	5.12906700	-0.70770800
H	-4.32449000	6.02357300	-0.76145400
C	-2.77065000	4.98394100	0.31740800
H	-2.65071400	5.76591100	1.07063900
C	0.43447500	-0.11772100	1.81349700
H	0.84180100	0.82352700	1.40093300
C	-1.08871800	-0.16694600	1.76192100
C	-1.74540700	-0.83128200	2.80201600
H	-1.12651600	-1.25822300	3.59174900
C	-3.13229600	-0.95702100	2.81624000
H	-3.62163900	-1.48642700	3.63558300
C	-3.89556800	-0.41512900	1.78109500
H	-4.98099300	-0.52518100	1.77593700
C	-3.26337100	0.28443400	0.75555700
H	-3.84431500	0.72445200	-0.05805000
C	-1.87731200	0.43045000	0.77364000
C	4.09298500	-2.69716200	-0.91457600
C	2.65971400	-2.32747700	-0.68004400
C	3.33727600	-0.35808600	0.68927300
C	4.73819800	-1.01119300	0.59643500
H	4.36247200	-3.53627700	-0.24721700
H	4.23406700	-3.02256900	-1.95208900
H	3.02805000	-0.25289100	1.74195100

H	4.88971000	-1.77804200	1.37473100
O	4.91354800	-1.58387100	-0.68559500
N	1.58726800	-2.96489100	-1.03264400
N	2.34072500	-1.21637800	0.05505300
C	5.70261500	0.17036800	0.70034700
H	5.92399600	0.38773700	1.75671400
H	6.64786400	-0.05803700	0.19056500
C	3.55870000	0.99194000	0.04016200
C	2.63089400	1.87882000	-0.49287900
C	4.92286300	1.29999700	0.06900100
C	3.08962700	3.09488900	-1.00820900
H	1.56694300	1.63616500	-0.51261000
C	5.37732000	2.51302900	-0.43879400
C	4.45014900	3.40915700	-0.97946100
H	6.44085100	2.75859400	-0.42492000
H	4.79366200	4.36085300	-1.38803800
C	0.99142300	-1.14098000	0.17649900
N	0.57661400	-2.22019600	-0.49647800
C	-0.78231000	-2.65757400	-0.63187000
C	-1.39282500	-3.26633700	0.47305500
C	-1.42648600	-2.48550200	-1.86057000
C	-2.73704000	-3.61915000	0.34774700
C	-2.76937300	-2.86608900	-1.93802800
C	-3.44501200	-3.40651600	-0.84074100
H	-3.24316000	-4.07849400	1.20086500
H	-3.30101400	-2.72782900	-2.88262500
C	-0.68092600	-1.95016100	-3.05037700
H	-1.37009800	-1.76144100	-3.88268000
H	-0.19002600	-1.00248200	-2.79555100
H	0.08458600	-2.66874900	-3.37920200
C	-0.60360000	-3.59989900	1.71160500
H	0.07983000	-4.43830600	1.50043000
H	0.00714100	-2.75786600	2.06729000
H	-1.27586600	-3.90526800	2.52360800
C	-4.91038900	-3.74375600	-0.92207800
H	-5.51123300	-2.95132300	-0.44863700
H	-5.24290300	-3.83873700	-1.96382800
H	-5.13316500	-4.68183700	-0.39545100
H	2.37754100	3.80167700	-1.43703500

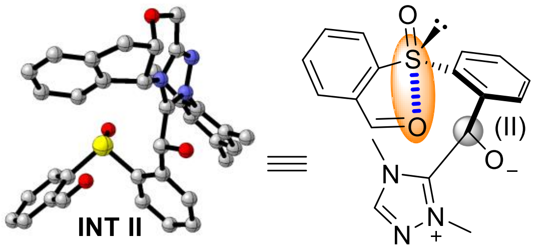

O	-0.09213000	2.64717600	1.40137300
O	0.98957900	-1.00512400	2.60686300
O	-1.88295600	0.48182000	-1.97816800
S	-1.21508800	1.14773900	-0.79682400
C	-0.79772600	3.62056600	1.29712800
H	-0.69621400	4.48311000	1.99234800
C	-1.83037300	3.76669100	0.25199700
C	-2.09014900	2.75787800	-0.69328000
C	-3.03433500	2.96620100	-1.69047500
H	-3.20075200	2.17556000	-2.42279400
C	-3.74959200	4.16732700	-1.73450100
H	-4.49509700	4.31774600	-2.51666000
C	-3.51463000	5.16617300	-0.79337400
H	-4.07261500	6.10192800	-0.83070600
C	-2.55272300	4.96215400	0.19441600
H	-2.34737000	5.73862000	0.93479000
C	0.43283600	-0.41497500	1.55385500
H	0.78850700	0.62066800	1.34834500
C	-1.10981800	-0.31545700	1.59584000
C	-1.74050500	-0.88763800	2.70470300
H	-1.08739500	-1.34953500	3.44512600
C	-3.12601700	-0.87530200	2.84021200
H	-3.59042200	-1.33879100	3.71256600
C	-3.92300600	-0.27724100	1.86201400
H	-5.01019300	-0.27858500	1.95186600
C	-3.31503400	0.34395300	0.77485500
H	-3.91650900	0.83590100	0.00714000
C	-1.92291400	0.35179100	0.67244200
C	4.02321500	-2.52005200	-1.11272700
C	2.59764800	-2.22429900	-0.75255300
C	3.30403100	-0.33713500	0.72315900
C	4.70220300	-0.97205300	0.53942800
H	4.33901800	-3.42917700	-0.56937500
H	4.10922500	-2.71384300	-2.18860800
H	3.00652700	-0.30580200	1.78504500
H	4.88042800	-1.80208900	1.24315800

O	4.83676200	-1.42539800	-0.79607200
N	1.52851100	-2.89877300	-1.04324700
N	2.30195300	-1.17883200	0.07477100
C	5.65884600	0.20708900	0.70997000
H	5.88977800	0.35573300	1.77605500
H	6.59946300	0.02328700	0.17452700
C	3.49593000	1.04581300	0.13490700
C	2.55526300	1.94400400	-0.35493300
C	4.85830400	1.36487000	0.15987700
C	2.99804200	3.18683700	-0.81802800
H	1.49320900	1.69539400	-0.38291800
C	5.29596300	2.60445600	-0.29452700
C	4.35466900	3.51557400	-0.78347000
H	6.35755700	2.85811900	-0.28230500
H	4.68468000	4.48878700	-1.15030600
C	0.97761600	-1.22063500	0.33667000
N	0.53064800	-2.26439600	-0.36750900
C	-0.82285700	-2.72486300	-0.51135100
C	-1.42858300	-3.38919800	0.56363200
C	-1.45530100	-2.51528600	-1.74153600
C	-2.76669300	-3.75281500	0.40957200
C	-2.79259400	-2.90728100	-1.84401000
C	-3.46930000	-3.49841300	-0.77433700
H	-3.27317900	-4.25388100	1.23842600
H	-3.31823900	-2.73606100	-2.78625800
C	-0.71132500	-1.95856400	-2.92341200
H	-1.41672200	-1.57376300	-3.66805400
H	-0.05442100	-1.12935700	-2.63214100
H	-0.09220800	-2.74473200	-3.38207300
C	-0.64856600	-3.75950700	1.79637600
H	0.00412900	-4.61912000	1.57220600
H	-0.01133000	-2.93773900	2.15895500
H	-1.33126200	-4.05612000	2.60320600
C	-4.93018700	-3.84643400	-0.88008700
H	-5.54227700	-3.06643000	-0.40067900
H	-5.24874200	-3.92523200	-1.92745300
H	-5.15137200	-4.79495600	-0.37218700
H	2.27487000	3.90320000	-1.21121200

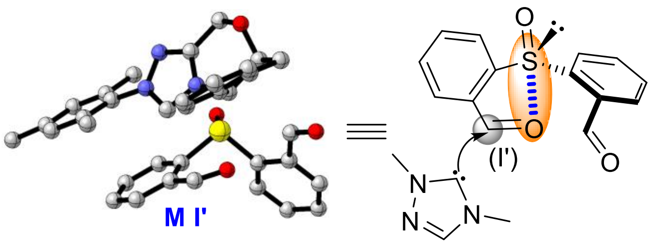

O	-1.07141900	-1.79587300	0.53478400
O	-1.07834200	-1.77169000	3.96162600
O	3.26133600	-1.30973200	0.78284100
S	1.75201900	-1.37880700	0.72533600
C	-1.11545400	-1.67120300	-0.66949200
H	-2.07803400	-1.53300100	-1.20429500
C	0.07946800	-1.77489200	-1.53737700
C	1.38125500	-1.78834000	-1.00862200
C	2.48494800	-1.89695700	-1.84447700
H	3.48245900	-1.85641400	-1.40356300
C	2.29636700	-2.00644000	-3.22543200
H	3.16432700	-2.08830700	-3.88155200
C	1.01323600	-1.97685000	-3.76555600
H	0.87040500	-2.03448200	-4.84527300
C	-0.09006100	-1.86228400	-2.92001100
H	-1.10167000	-1.83445200	-3.33170300
C	-0.22631200	-1.77062300	3.10872000
H	0.08420100	-0.82298300	2.62135700
C	0.49476700	-2.98926800	2.64682300
C	0.30016800	-4.20009400	3.31895800
H	-0.38774400	-4.21667500	4.16578700
C	0.97756900	-5.34533600	2.91109600
H	0.82321000	-6.28738600	3.43827400
C	1.85982400	-5.28787000	1.82884000
H	2.39538200	-6.18389300	1.51236400
C	2.06275600	-4.08704600	1.14932400
H	2.75871400	-4.02865700	0.30969600
C	1.36606200	-2.95248200	1.55128100
C	0.43991200	2.91883100	2.36166100
C	0.27436800	2.34420400	0.98845800
C	-1.75815000	1.10716200	1.71533300
C	-1.37531600	1.62186500	3.12483400
H	1.19072200	2.32608500	2.91722600
H	0.79290600	3.95487900	2.30207600
H	-1.86506900	0.01078000	1.72182700
H	-0.68596100	0.93771900	3.64932700
O	-0.79654800	2.90954800	3.02823500

N	0.98946000	2.52526200	-0.07467100
N	-0.73640600	1.45012300	0.73893200
C	-2.72037800	1.77304600	3.83632400
H	-2.99940900	0.81486400	4.30181500
H	-2.65974500	2.53643600	4.62315400
C	-3.10591300	1.75772700	1.48106500
C	-3.77929600	1.97390000	0.28433900
C	-3.66483200	2.12331900	2.70950400
C	-5.04035800	2.57633800	0.33144900
H	-3.32442200	1.68398100	-0.66551000
C	-4.92263900	2.71676100	2.75456300
C	-5.60613700	2.94187200	1.55548700
H	-5.36583800	3.01212000	3.70745900
H	-6.58969900	3.41407100	1.57542500
C	-0.67958000	1.02428900	-0.55221000
N	0.39617000	1.70391100	-1.00066500
C	0.91597000	1.62230700	-2.33238300
C	2.29791700	1.44826400	-2.52961200
C	0.02560900	1.71297900	-3.41835500
C	2.76277600	1.33347200	-3.84220500
C	0.54752500	1.57916900	-4.70864100
C	1.90783600	1.37811100	-4.94432500
H	3.83438700	1.18784000	-4.00229800
H	-0.13814700	1.64733500	-5.55717500
C	-1.45129300	1.96184100	-3.25130100
H	-1.87588300	2.33125400	-4.19357400
H	-1.97907300	1.04196400	-2.96228300
H	-1.64852400	2.69862100	-2.46025200
C	3.28669900	1.38003400	-1.39608800
H	3.46113500	2.37512500	-0.96500600
H	2.94584600	0.73502200	-0.57605800
H	4.24292900	0.98173900	-1.76038100
C	2.43618900	1.18341800	-6.34082400
H	3.44551500	1.60285400	-6.44815900
H	2.49811500	0.11026600	-6.58249700
H	1.78042600	1.65325900	-7.08566600
H	-5.58610300	2.76422900	-0.59442400

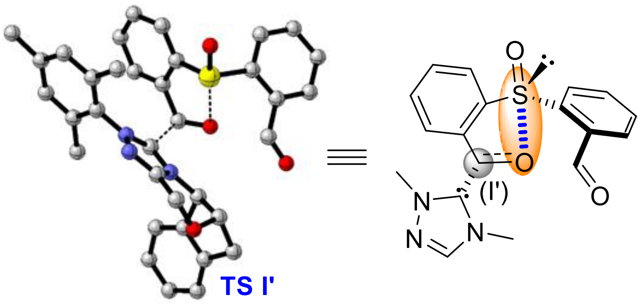

O	-0.03678900	-0.05529800	-0.00474400
O	-0.06928800	-0.09899100	3.35704000
O	4.31046200	0.03103700	0.15820100
S	2.79740700	0.09837800	0.13820200
C	-0.05308300	0.36307800	-1.17185900
H	-1.00880800	0.56225100	-1.69911300
C	1.07448100	-0.00067900	-2.09727400
C	2.35731800	-0.25017000	-1.59234800
C	3.41382100	-0.57490000	-2.43610100
H	4.40961300	-0.69947200	-2.00691600
C	3.18369300	-0.69273900	-3.80836400
H	4.00812700	-0.95010100	-4.47499700
C	1.91121200	-0.45862500	-4.32543300
H	1.73383900	-0.53405100	-5.39934000
C	0.86343100	-0.11350100	-3.47054000
H	-0.13410400	0.08031600	-3.87290300
C	0.81154800	-0.15338700	2.53442600
H	1.23153800	0.77466600	2.09320200
C	1.43076500	-1.42091300	2.05932800
C	1.14113100	-2.61766200	2.72170300
H	0.45655900	-2.58616300	3.57082800
C	1.72301300	-3.81056400	2.30238200
H	1.49478700	-4.74155000	2.82243600
C	2.60356400	-3.81430600	1.21755700
H	3.06464300	-4.74747800	0.89114400
C	2.89938800	-2.62739100	0.54727000
H	3.59360300	-2.61814900	-0.29564000
C	2.29672900	-1.44426500	0.95969300
C	1.31856900	4.60724800	1.77289900
C	1.22080300	3.91790100	0.44601000
C	-0.70839300	2.56845000	1.25470200
C	-0.38213600	3.20475100	2.62432500
H	2.12287100	4.13106700	2.36407900
H	1.57108100	5.66468600	1.63348800
H	-0.75558000	1.47277200	1.32614400

H	0.35336600	2.61387700	3.19720700
O	0.08768700	4.52849100	2.44135800
N	1.92795100	4.08702800	-0.62655800
N	0.31280800	2.90500900	0.27023200
C	-1.74702400	3.29707500	3.30725400
H	-1.96521700	2.34526800	3.81599600
H	-1.75657200	4.10284300	4.05285800
C	-2.08430500	3.12096300	0.95149500
C	-2.74437600	3.22053900	-0.26665300
C	-2.69108500	3.51917100	2.14694900
C	-4.04346300	3.73878000	-0.27842400
H	-2.25701600	2.90868200	-1.19353800
C	-3.98572500	4.02791900	2.13414100
C	-4.65714200	4.13603600	0.91167500
H	-4.46815400	4.34754600	3.05970000
H	-5.67003200	4.54102100	0.88668400
C	0.45785600	2.39036300	-0.97277000
N	1.44671700	3.13763200	-1.48602000
C	1.92912800	3.06672800	-2.83460200
C	3.30312500	2.89596400	-3.06898100
C	1.00189100	3.17971600	-3.88242800
C	3.72493800	2.79925000	-4.39663900
C	1.48000900	3.07390200	-5.19130500
C	2.83243500	2.86988500	-5.46927400
H	4.79003100	2.65528000	-4.59506700
H	0.76888900	3.15817800	-6.01681600
C	-0.46799400	3.40242800	-3.63929400
H	-0.95154500	3.76416900	-4.55512500
H	-0.96670400	2.46843800	-3.33691900
H	-0.63974500	4.13596200	-2.83867800
C	4.30954500	2.82845900	-1.95248800
H	4.39514200	3.79743900	-1.44237100
H	4.04233100	2.08305200	-1.19094500
H	5.29390900	2.55255500	-2.35141300
C	3.31720000	2.69968100	-6.88423400
H	4.31563900	3.13657200	-7.01944600
H	3.38886100	1.62990200	-7.13701100
H	2.62930000	3.16582600	-7.60156400
H	-4.58021200	3.83456800	-1.22330100

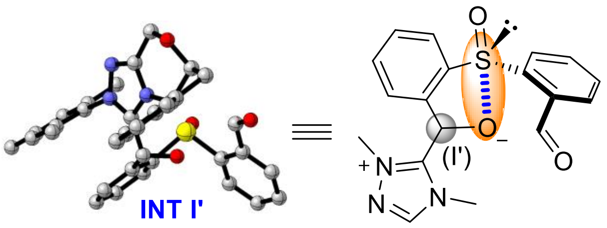

O	-0.71258900	-1.28286400	0.57589200
O	-0.71688500	-1.29547800	3.89768300
O	3.50759200	-1.79245500	0.46345800
S	2.02214200	-1.48359900	0.54622100
C	-0.72954700	-0.61759400	-0.57255700
H	-1.72993900	-0.51699400	-1.07245200
C	0.21174400	-1.25162900	-1.60086000
C	1.43798800	-1.75805700	-1.15776400
C	2.34863600	-2.33625800	-2.03628200
H	3.32111500	-2.65125800	-1.65341600
C	2.00813400	-2.47127000	-3.38320800
H	2.71161900	-2.93287600	-4.07769100
C	0.77319100	-2.01055000	-3.83596500
H	0.50167000	-2.11436100	-4.88783600
C	-0.11722500	-1.40611400	-2.94643600
H	-1.08094400	-1.03740900	-3.30659400
C	0.12958400	-1.45027000	3.04880700
H	0.66405100	-0.58064900	2.61504200
C	0.55935600	-2.78775800	2.55505700
C	0.14977700	-3.93049600	3.24770100
H	-0.48288800	-3.80098200	4.12727100
C	0.54813800	-5.19465100	2.82065500
H	0.22513300	-6.08249900	3.36546800
C	1.36698600	-5.32289900	1.69693500
H	1.68673800	-6.31092800	1.36265200
C	1.78411500	-4.18958400	0.99782800
H	2.43547500	-4.27878800	0.12620400
C	1.35896600	-2.93413800	1.41595600
C	0.05567400	3.49070300	2.15252700
C	0.08605900	2.65581100	0.90601900
C	-1.60370200	1.10316400	1.86465200
C	-1.33491000	1.86198600	3.17702900
H	0.96560600	3.25862900	2.73658600
H	0.07491300	4.55570600	1.89340600
H	-1.51792100	0.01366300	1.96910800
H	-0.49409500	1.43361600	3.74838600
O	-1.10418600	3.23333300	2.89123000

N	0.81052000	2.80609800	-0.16112700
N	-0.62524700	1.48992200	0.84973900
C	-2.67943800	1.78022300	3.89781600
H	-2.74611000	0.81829400	4.42899400
H	-2.78818800	2.59541500	4.62476800
C	-3.03495700	1.48427000	1.55588800
C	-3.72106400	1.47832100	0.34896500
C	-3.66719700	1.83997800	2.75312700
C	-5.07378700	1.83590000	0.34940800
H	-3.22189100	1.21164700	-0.58521400
C	-5.01326200	2.18689800	2.75301300
C	-5.71287300	2.18206300	1.54106300
H	-5.51506200	2.47114000	3.67976500
H	-6.76770700	2.46044500	1.52582800
C	-0.29012900	0.86015100	-0.29185900
N	0.56532400	1.68913700	-0.89828000
C	1.07550900	1.58867500	-2.24006500
C	2.43424400	1.32520300	-2.44824200
C	0.16607200	1.79065200	-3.28662700
C	2.85749300	1.19791000	-3.77394500
C	0.64306500	1.65965400	-4.59035500
C	1.97982400	1.34231700	-4.85172900
H	3.91169000	0.98322700	-3.96620200
H	-0.04866500	1.80802800	-5.42287800
C	-1.27974000	2.10837000	-3.01306800
H	-1.76935300	2.48594100	-3.91856100
H	-1.82888200	1.20782800	-2.69130600
H	-1.38578800	2.86317400	-2.22010200
C	3.41236400	1.18301900	-1.31390600
H	3.11783200	1.78507200	-0.44523300
H	3.51018500	0.13674000	-0.98462300
H	4.40836700	1.51200000	-1.63755200
C	2.46145000	1.13750700	-6.26256400
H	3.50031600	1.47203200	-6.38231900
H	2.42723300	0.06732900	-6.52099900
H	1.83167100	1.67500600	-6.98309200
H	-5.63090500	1.84568100	-0.58827100

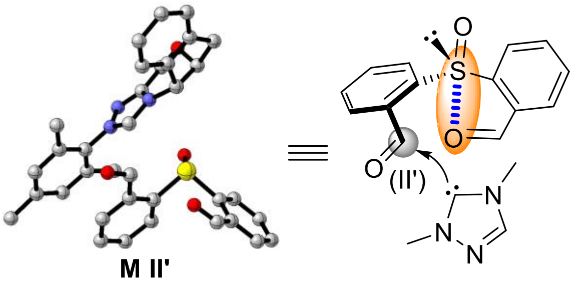

M II'

C	2.56773300	-0.49983000	3.23835800
C	1.64352600	-0.99960700	2.17284200
C	2.77394900	0.40760800	0.46888700
C	3.52720900	0.98812800	1.69109000
H	2.06367700	0.30412900	3.80628500
H	2.82644400	-1.30726200	3.93338500
H	2.25676400	1.21855500	-0.06930800
H	2.96539800	1.81800200	2.15283600
O	3.75252900	-0.02772800	2.64872600
N	0.63911200	-1.80945000	2.27204900
N	1.77384500	-0.55541900	0.88478300
C	4.89110200	1.39789900	1.13006400
H	4.85474400	2.43304800	0.75640300
H	5.65934600	1.34418500	1.91291500
C	3.89029600	-0.15715600	-0.38197900
C	3.81637500	-1.08458500	-1.41578000
C	5.10500100	0.42305900	-0.00573300
C	4.99698800	-1.43511200	-2.07719300
H	2.85639700	-1.52590600	-1.69480800
C	6.27868700	0.07512800	-0.66852600
C	6.21549000	-0.85967800	-1.70627100
H	7.23442900	0.51628600	-0.37920900
H	7.12885200	-1.14720300	-2.22967000
C	0.79209400	-1.07996400	0.10315900
N	0.13397100	-1.83863900	0.99918700
C	-1.03648100	-2.61080900	0.72402100
C	-2.20310200	-2.35109600	1.46046900
C	-0.98752700	-3.59665000	-0.27336400
C	-3.33648100	-3.11617500	1.17576700
C	-2.15521400	-4.32312700	-0.52691000
C	-3.33674900	-4.09942600	0.18343600
H	-4.25422400	-2.92050300	1.73738100
H	-2.13250600	-5.09748000	-1.29821400
C	0.26782500	-3.88407200	-1.05505000
H	0.20099200	-4.87311200	-1.52645900

H	0.41438300	-3.13081600	-1.84275200
H	1.15685700	-3.85841100	-0.40961100
C	-2.24739500	-1.27984400	2.51805700
H	-1.80594500	-1.64063600	3.45874000
H	-1.67416400	-0.39305500	2.20904700
H	-3.28806500	-0.98595000	2.71125600
C	-4.58995000	-4.87198600	-0.13598200
H	-4.35455000	-5.85453700	-0.56600700
H	-5.20626100	-5.02132500	0.76072700
H	-5.20412000	-4.32816100	-0.87126900
H	4.96771000	-2.16470100	-2.88785700
O	-0.86874800	2.43452100	-2.77332400
O	-0.36144000	-1.26997500	-3.21509100
O	-0.35241300	1.44763000	1.43794800
S	-0.49993100	1.74356000	-0.04225200
C	-1.38386400	3.51115400	-2.58888200
H	-1.67939900	4.16159400	-3.44162300
C	-1.65701100	4.06565700	-1.24656600
C	-1.32779500	3.37066800	-0.06804700
C	-1.55904300	3.94819300	1.17353900
H	-1.27132500	3.39565500	2.06866200
C	-2.15111300	5.21306800	1.25112000
H	-2.33824500	5.65776100	2.22957500
C	-2.49705000	5.90531300	0.09310800
H	-2.95624500	6.89189100	0.15739800
C	-2.24460700	5.33026400	-1.15143000
H	-2.50024600	5.86493700	-2.06894000
C	-0.48532000	-0.39360900	-2.39276200
H	0.31152600	0.34735100	-2.21078500
C	-1.75399000	-0.21671200	-1.61111400
C	-2.83690300	-1.04585800	-1.92007400
H	-2.70141800	-1.78999500	-2.70628500
C	-4.04351600	-0.92268300	-1.23831500
H	-4.88044100	-1.57609800	-1.48946300
C	-4.18017700	0.02883000	-0.22578500
H	-5.12130000	0.12242800	0.31828500
C	-3.10812600	0.85652800	0.10125000
H	-3.19870700	1.58962100	0.90596200
C	-1.91065600	0.73871900	-0.59986100

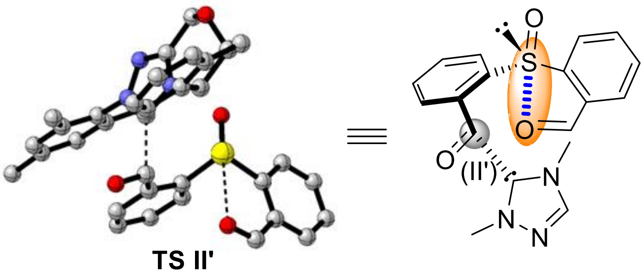

TS II'

C	2.57564400	-0.33409600	3.14035000
C	1.61058200	-0.85204800	2.12276300
C	2.70734000	0.50261200	0.33976400
C	3.54451500	1.06314900	1.51955300
H	2.11240300	0.51490100	3.67576100
H	2.82069100	-1.11709000	3.86693500
H	2.18897200	1.33007200	-0.17103000
H	3.04993400	1.93341700	1.98353500
O	3.75701400	0.06135200	2.49309000
N	0.61835200	-1.67151400	2.27093200
N	1.69536900	-0.42692200	0.82296400
C	4.90249100	1.38496800	0.89166700
H	4.91058600	2.41668400	0.50746100
H	5.69914100	1.29398600	1.64187300
C	3.74806400	-0.11833800	-0.56546600
C	3.56967700	-1.02609000	-1.60536600
C	5.00875200	0.39121600	-0.24185000
C	4.70119900	-1.43953400	-2.31554700
H	2.57485300	-1.39318800	-1.87936700
C	6.13071000	-0.02137400	-0.95488400
C	5.96750200	-0.94529000	-1.99102100
H	7.12154400	0.36390700	-0.70716300
H	6.83894100	-1.28362200	-2.55399900
C	0.69067500	-0.98626200	0.11361800
N	0.06440300	-1.73596900	1.02719400
C	-1.07157800	-2.57327800	0.78064600
C	-2.28043900	-2.28901000	1.43100600
C	-0.92538900	-3.63841600	-0.11808300
C	-3.37961200	-3.09230800	1.12016200
C	-2.05898600	-4.40706900	-0.39627500
C	-3.29408700	-4.14197300	0.20099700
H	-4.33624400	-2.88056200	1.60517000
H	-1.96904000	-5.24078700	-1.09710500
C	0.39812600	-3.94235900	-0.76833300
H	0.37881200	-4.94190100	-1.22075400
H	0.60930800	-3.20156300	-1.55592400

H	1.21792400	-3.90570600	-0.03582900
C	-2.39785100	-1.17734700	2.43829000
H	-2.02647600	-1.51126800	3.41958000
H	-1.80637300	-0.29695900	2.15001400
H	-3.44920600	-0.88129700	2.55236600
C	-4.51394200	-4.95199800	-0.15160500
H	-4.23819000	-5.93483900	-0.55541500
H	-5.15994500	-5.10116000	0.72408700
H	-5.11211000	-4.43293400	-0.91693400
H	4.59284800	-2.15389300	-3.13298800
O	-0.53583300	2.58120800	-2.48722500
O	0.37471300	-1.36297900	-2.62995500
O	-0.48372400	1.47639700	1.70071200
S	-0.51074300	1.83281100	0.22444500
C	-1.13220900	3.62199700	-2.35040200
H	-1.34978300	4.28105300	-3.22001900
C	-1.61166300	4.12432500	-1.04362400
C	-1.40291400	3.41229200	0.15147800
C	-1.81765600	3.93592400	1.36833500
H	-1.61788900	3.36800500	2.27789100
C	-2.47880000	5.16815800	1.39943900
H	-2.81288800	5.57352500	2.35553900
C	-2.70882000	5.87774300	0.22289700
H	-3.22272100	6.83870400	0.25198100
C	-2.27016700	5.35567900	-0.99348700
H	-2.43244600	5.90710800	-1.92227600
C	0.10484600	-0.49039800	-1.79063000
H	0.71931900	0.43430300	-1.73499500
C	-1.35191100	-0.24613200	-1.40777100
C	-2.31862700	-1.09941100	-1.94246000
H	-1.97582900	-1.88231300	-2.62048400
C	-3.66194800	-0.95753500	-1.60239800
H	-4.40367500	-1.64016600	-2.02113700
C	-4.06084700	0.04926300	-0.72129700
H	-5.11057100	0.15813000	-0.44507900
C	-3.11196300	0.92315300	-0.19308000
H	-3.41454200	1.71549800	0.49498600
C	-1.77232500	0.77611800	-0.54878200

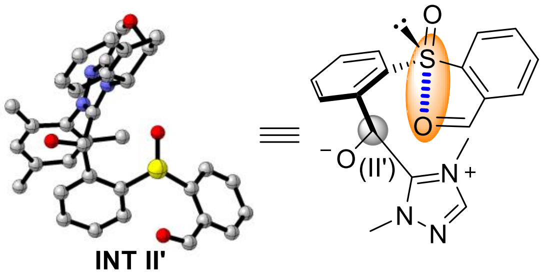

C	2.92329300	-0.91535700	3.06968700
C	1.84589900	-1.21649400	2.07234100
C	2.89867100	0.29967700	0.39135800
C	3.73579500	0.74295600	1.61561200
H	2.52365500	-0.21185200	3.82267000
H	3.22683500	-1.83576800	3.58263200
H	2.34096900	1.16274300	-0.00122300
H	3.20758800	1.50254700	2.21663500
O	4.04078300	-0.38231000	2.41673500
N	0.76314500	-1.90923200	2.24176100
N	1.89094200	-0.67247500	0.81659800
C	5.04629800	1.23395900	1.00295300
H	4.96512600	2.29926900	0.73704000
H	5.87090300	1.12057300	1.71905600
C	3.95222100	-0.18792700	-0.58192300
C	3.81347300	-1.00948000	-1.69735300
C	5.18457300	0.37644200	-0.23340300
C	4.95617100	-1.26619800	-2.46339500
H	2.83962500	-1.44799400	-1.95737500
C	6.31392900	0.12112100	-1.00450700
C	6.19078500	-0.70830100	-2.12307000
H	7.28020700	0.55307400	-0.73716200
H	7.06909500	-0.92476300	-2.73366600
C	0.75555400	-1.02931100	0.18668000
N	0.09827900	-1.79087400	1.06099900
C	-1.17824500	-2.42978000	0.88786900
C	-2.32628400	-1.75706100	1.32687900
C	-1.21018400	-3.69843400	0.30003800
C	-3.55458800	-2.37793600	1.09796400
C	-2.46595800	-4.27694200	0.09733500
C	-3.64461600	-3.62585900	0.47204400
H	-4.46833800	-1.86711300	1.41247800
H	-2.52103200	-5.26231500	-0.37160400
C	0.06275100	-4.38032600	-0.11459000
H	-0.14625600	-5.37090700	-0.53699500
H	0.57275000	-3.75095400	-0.86341400

H	0.73620900	-4.50268500	0.74767100
C	-2.23803000	-0.43603500	2.04092800
H	-1.97604500	-0.59500300	3.09940000
H	-1.46643800	0.21573900	1.60622600
H	-3.20395200	0.08492100	2.00442200
C	-4.99086100	-4.23842300	0.19025800
H	-4.90766500	-5.31413000	-0.01074000
H	-5.67934400	-4.09053300	1.03341500
H	-5.44782400	-3.76526100	-0.69299200
H	4.88059800	-1.91314600	-3.33902400
O	-1.49016100	3.21952700	-2.93456200
O	0.85752800	-1.91605300	-1.95612800
O	0.12410700	1.54444600	0.71875500
S	-0.49276800	2.05148800	-0.58058000
C	-2.07732700	4.13073600	-2.40483700
H	-2.51700500	4.96149600	-3.00011200
C	-2.26916500	4.23317800	-0.93970900
C	-1.66598200	3.32820200	-0.04777600
C	-1.84701500	3.46186500	1.32242200
H	-1.32905900	2.77304200	1.99132000
C	-2.67099400	4.47919500	1.81337800
H	-2.82279300	4.57388100	2.88941700
C	-3.28635600	5.37497900	0.94107600
H	-3.92208100	6.17095000	1.32929900
C	-3.07606900	5.25475600	-0.43183500
H	-3.54122800	5.95719500	-1.12705500
C	0.45783300	-0.82872400	-1.31318900
H	1.03003600	0.11177900	-1.56226200
C	-1.03815100	-0.47194900	-1.49917200
C	-1.84860700	-1.45798800	-2.06539700
H	-1.34753600	-2.38013100	-2.36259300
C	-3.21705000	-1.26556400	-2.22126400
H	-3.83433500	-2.05594000	-2.65242100
C	-3.80049100	-0.06440100	-1.81587000
H	-4.87482100	0.09376300	-1.92043300
C	-3.00506300	0.95069300	-1.28970400
H	-3.46432000	1.89545400	-0.99834000
C	-1.63019000	0.74988500	-1.14847400

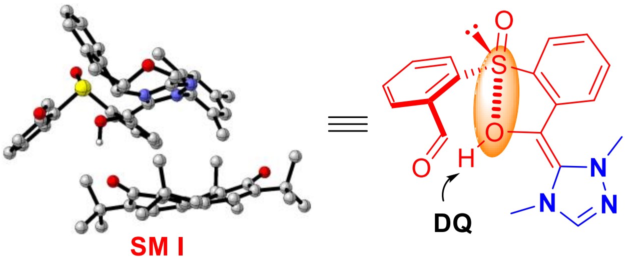

C	-0.70391700	2.39986300	-5.03657300
C	-0.90633700	1.97081200	-3.61584500
C	1.11121500	3.21311300	-2.93347200
C	1.30675500	3.46753800	-4.44486200
H	-0.19287100	1.58425200	-5.58280700
H	-1.67367200	2.58372500	-5.51244500
H	2.06135300	2.87518000	-2.49917900
H	1.90880400	2.67520600	-4.92295200
O	0.05369700	3.58000500	-5.09092700
N	-1.89228000	1.31079600	-3.12509200
N	0.08647000	2.21110400	-2.69406600
C	1.96098100	4.85127700	-4.48027100
H	3.04883800	4.75688100	-4.33668200
H	1.77884600	5.34536200	-5.44370300
C	0.78344100	4.60126100	-2.42432700
C	0.07060400	4.98250500	-1.29482000
C	1.31573000	5.54942000	-3.30349400
C	-0.07799300	6.34819000	-1.02914600
H	-0.35204500	4.23315100	-0.62323900
C	1.17367500	6.90678700	-3.03629100
C	0.47695200	7.29963900	-1.88771200
H	1.58220300	7.65563600	-3.71752600
H	0.35218300	8.36145700	-1.66935500
C	-0.30031600	1.60622200	-1.49666700
N	-1.55584700	1.09428900	-1.80255900
C	-2.50854300	0.52278300	-0.91698500
C	-2.80792500	-0.84180800	-1.04693400
C	-3.10240300	1.31693400	0.07636500
C	-3.68051800	-1.41997800	-0.12402600
C	-3.96696700	0.69552900	0.98171400
C	-4.26065900	-0.66920700	0.90401800
H	-3.90737100	-2.48663100	-0.20275100
H	-4.42893100	1.29937400	1.76742800
C	-2.81221100	2.79013400	0.17195000
H	-3.63937000	3.31495600	0.66626000
H	-1.90161200	2.97757200	0.76408700

H	-2.65655000	3.23063100	-0.82312700
C	-2.15192500	-1.65562400	-2.12666200
H	-2.35085600	-1.22997000	-3.12037500
H	-1.05696400	-1.65616700	-1.99428500
H	-2.50439700	-2.69437900	-2.10113700
C	-5.20743200	-1.31131400	1.88318500
H	-5.15985800	-2.40674400	1.81751800
H	-4.97301300	-1.01290300	2.91495400
H	-6.24464400	-1.00310100	1.68082200
H	-0.63136700	6.66961300	-0.14519500
O	1.68587300	2.16146300	-0.34167600
O	3.94847700	4.27672500	-0.60882000
O	-0.07086300	4.09229300	3.31047300
S	0.56051600	3.49988000	2.07129400
C	0.44173000	1.53893600	-0.34600600
C	0.01772300	0.97123400	0.94198700
C	0.08396600	1.74195200	2.11992800
C	-0.38326400	1.26940100	3.34154800
H	-0.39337500	1.94582800	4.19807600
C	-0.87892400	-0.03166700	3.43324800
H	-1.25168000	-0.40923500	4.38677300
C	-0.90661800	-0.83701000	2.29418600
H	-1.29319400	-1.85670300	2.34996200
C	-0.45363000	-0.34408000	1.07272200
H	-0.46066100	-0.98555200	0.18974300
C	3.09904000	4.21809100	0.24673800
H	2.08613500	4.62946200	0.07714800
C	3.35530100	3.65883800	1.60926100
C	4.68413500	3.50929500	2.02525000
H	5.47384000	3.74059600	1.30858300
C	4.97891200	3.11678800	3.32679700
H	6.01728700	3.01591600	3.64502700
C	3.94219700	2.87568300	4.23224500
H	4.16826800	2.59045700	5.26076700
C	2.61535900	2.99011300	3.82359000
H	1.79606400	2.80668000	4.52088400
C	2.32814100	3.34965400	2.50930600
H	2.36032800	1.45857900	-0.40677500
C	-0.44778500	-4.19793600	0.48038800
C	-1.36435100	-4.71620100	-0.52411100
C	-2.60633600	-5.16588500	-0.23105400
C	-3.08530800	-5.06293600	1.18502700
C	-2.07220900	-4.77733800	2.24884400
C	-0.83647500	-4.37976300	1.87004300

C	0.70225800	-3.50107400	0.14266700
C	1.00802900	-3.15235000	-1.23643600
C	1.87824300	-2.16998600	-1.56788000
C	2.51958600	-1.40140900	-0.45814400
C	2.52849700	-2.02433300	0.89715500
C	1.62483000	-2.99835600	1.14914000
H	-1.02139400	-4.75605800	-1.55453200
H	-0.10821500	-4.13558800	2.63766000
H	0.48607200	-3.68118600	-2.02973500
H	1.57513600	-3.42070600	2.14911100
C	-2.47854400	-4.90653200	3.71657300
C	-1.28640100	-4.65600300	4.64694300
C	-3.56508100	-3.86852000	4.05391500
C	-3.00453600	-6.32796900	3.98721100
H	-0.46133800	-5.35756900	4.45305200
H	-0.90217600	-3.62844100	4.55332900
H	-1.60961400	-4.79453200	5.68816700
H	-4.48460700	-4.05323200	3.48794000
H	-3.79672700	-3.92006200	5.12873700
H	-3.21033900	-2.84982700	3.83071000
H	-3.27076000	-6.42259900	5.05068300
H	-3.89537500	-6.54713600	3.38686300
H	-2.23016400	-7.07692100	3.76207700
C	-3.53889500	-5.77121300	-1.28147300
C	-3.97199800	-7.17618000	-0.82038200
C	-4.78122800	-4.88449900	-1.48941300
C	-2.83246800	-5.91772400	-2.63361200
H	-3.09294800	-7.81770700	-0.65567800
H	-4.55780200	-7.13319100	0.10528200
H	-4.59137300	-7.64115400	-1.60183800
H	-4.49862500	-3.90633600	-1.90710600
H	-5.45609000	-5.37075800	-2.21018700
H	-5.32560600	-4.72696800	-0.55136000
H	-3.52232700	-6.38603600	-3.34970700
H	-2.53537300	-4.94402900	-3.05199800
H	-1.93775300	-6.55354900	-2.55896800
C	3.48904500	-1.49248000	1.95747300
C	3.50445100	-2.40269800	3.19084800
C	4.91965500	-1.43315000	1.39231500
C	3.03889600	-0.09385700	2.41277100
H	2.53255700	-2.40967400	3.70634500
H	3.77143700	-3.43787000	2.92959900
H	4.25127600	-2.02714000	3.90457400
H	4.99419800	-0.72890400	0.55547700

H	5.60926000	-1.10290000	2.18345800
H	5.24572300	-2.42751000	1.05018800
H	3.74213700	0.28693000	3.16884000
H	3.00595200	0.62359100	1.58258100
H	2.03550900	-0.13960300	2.86460500
C	2.18859900	-1.79439800	-3.01607300
C	1.57950400	-2.80547600	-3.99346100
C	1.59605500	-0.41105500	-3.33273900
C	3.71297100	-1.78034200	-3.22935300
H	1.93736900	-3.82729200	-3.79757900
H	0.47955400	-2.80488500	-3.95305900
H	1.87042000	-2.53392300	-5.01801900
H	2.01152300	0.36264400	-2.67637500
H	1.81530100	-0.14649900	-4.37971600
H	0.50145400	-0.42540600	-3.20488600
H	3.93262600	-1.54884000	-4.28225500
H	4.20049100	-1.02505200	-2.60132200
H	4.14585300	-2.76595500	-2.99997500
O	-4.26342700	-5.22142400	1.46316900
O	3.03451000	-0.30668600	-0.65879900

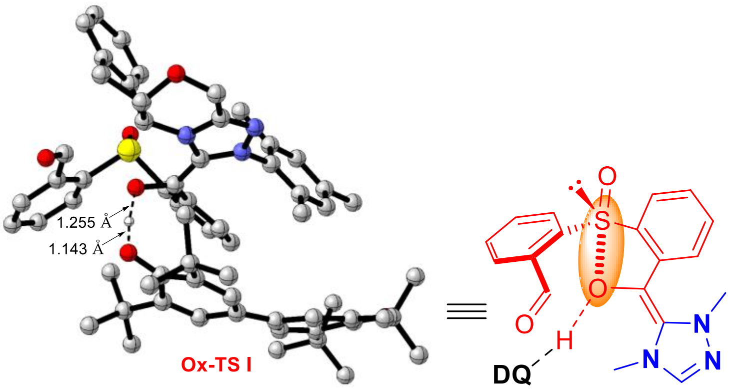

C	1.26355000	0.04285500	-0.00131000
C	1.06471300	-0.43425000	1.40678500
C	3.14041900	0.67630500	2.10575900
C	3.36241900	0.94223600	0.60218000
H	1.67751700	-0.79851200	-0.58877700
H	0.29929600	0.32832100	-0.43658100
H	4.06411500	0.29903400	2.56328700
H	3.91846200	0.12778200	0.10813200
O	2.11832600	1.15153500	-0.03907800
N	0.05983600	-1.08821100	1.89451300
N	2.06506200	-0.28498500	2.32558700
C	4.09597900	2.28682900	0.61134900
H	5.16997400	2.12618100	0.79310600
H	3.97627400	2.80543700	-0.34863000
C	2.82858900	2.06783700	2.61560600
C	2.06443600	2.46166000	3.70450100
C	3.44408000	3.00066400	1.77568700
C	1.96596700	3.82768800	3.98947400
H	1.56035500	1.72503500	4.33241000
C	3.34952100	4.35806000	2.06078400
C	2.61415900	4.76359000	3.18109200
H	3.82273400	5.09857800	1.41349200
H	2.52852900	5.82626200	3.41293900
C	1.67994700	-0.95189000	3.46603700
N	0.41493300	-1.38462700	3.17561800
C	-0.54499800	-1.99905700	4.03933500
C	-0.85194200	-3.35008600	3.83139800
C	-1.14453400	-1.23264900	5.04871600
C	-1.73215800	-3.96033700	4.72596600
C	-2.03112100	-1.88446300	5.90827100
C	-2.32421900	-3.24582600	5.77300800
H	-1.96239700	-5.02124500	4.60186700
H	-2.50803400	-1.30937200	6.70593600
C	-0.85187600	0.23602200	5.20384900

H	-1.67419700	0.73459300	5.73144300
H	0.06172500	0.40528900	5.79667200
H	-0.71991400	0.72487000	4.22785100
C	-0.23278800	-4.10590300	2.69045600
H	-0.56066800	-3.69274700	1.72527300
H	0.86588000	-4.02906300	2.72056100
H	-0.50937700	-5.16631800	2.73607900
C	-3.29719100	-3.92224600	6.70057100
H	-3.17698700	-5.01361300	6.66984700
H	-3.16278500	-3.58143000	7.73642000
H	-4.33219800	-3.68671000	6.40811600
H	1.37769900	4.15985200	4.84604900
O	3.73852900	-0.69157900	4.53702200
O	6.04446100	1.36142100	4.11612400
O	2.05713600	1.69725500	8.05931000
S	2.68537300	0.99290800	6.88012500
C	2.51532800	-1.13693600	4.61203500
C	2.00603300	-1.58249400	5.92966500
C	2.13302900	-0.73675600	7.04897400
C	1.62773100	-1.09578400	8.29294600
H	1.66249900	-0.36862400	9.10606900
C	1.03574700	-2.34907300	8.46237200
H	0.63922900	-2.63591400	9.43756400
C	0.93258800	-3.21814000	7.37683000
H	0.45807000	-4.19480700	7.48975800
C	1.41651600	-2.83790500	6.12677700
H	1.34458400	-3.52423500	5.28343700
C	5.20828600	1.41275200	4.98554900
H	4.19267500	1.79883200	4.77786500
C	5.47198500	1.03148300	6.40354600
C	6.79970700	0.91511000	6.83184900
H	7.59121600	1.05144000	6.09323000
C	7.09084100	0.66771300	8.16940000
H	8.12827700	0.59109600	8.49711500
C	6.05310800	0.53675600	9.09656300
H	6.27868300	0.36246300	10.14953800
C	4.72624400	0.62422800	8.68057700
H	3.90569100	0.53316200	9.39453000
C	4.44342200	0.84301300	7.33521400
H	4.53256300	-1.63842900	4.31938000
C	1.45625900	-6.50469100	5.32468700
C	0.58058100	-7.08843900	4.34586500
C	-0.63240000	-7.63074100	4.65266800
C	-1.10767500	-7.57330300	6.05978800

C	-0.13088900	-7.15683900	7.09724200
C	1.07151100	-6.65192900	6.70057100
C	2.59923100	-5.74649300	4.96118200
C	2.88849200	-5.40603800	3.60269300
C	3.77006100	-4.41079700	3.25839100
C	4.43077600	-3.67295900	4.32479400
C	4.43217100	-4.24904200	5.65513000
C	3.50420800	-5.21816400	5.93902600
H	0.91271500	-7.10728800	3.31041900
H	1.76392100	-6.30603000	7.46332900
H	2.35988000	-5.93106400	2.81263800
H	3.46225000	-5.61682700	6.94828400
C	-0.50747900	-7.29132100	8.57464800
C	0.65182700	-6.88447100	9.49179200
C	-1.70734400	-6.38652400	8.90682000
C	-0.85922200	-8.75824900	8.88390900
H	1.54944400	-7.49455100	9.31116600
H	0.92217300	-5.82403300	9.36935600
H	0.34984800	-7.02975900	10.53889100
H	-2.59037400	-6.67286200	8.32449400
H	-1.94616600	-6.46851000	9.97865700
H	-1.46774200	-5.33293900	8.69215600
H	-1.11427700	-8.86063700	9.94992400
H	-1.71435900	-9.09409200	8.28598900
H	0.00048500	-9.41292600	8.67446100
C	-1.52584300	-8.28791600	3.59715300
C	-1.86130800	-9.72682100	4.03309000
C	-2.82542200	-7.48200900	3.41284600
C	-0.82664900	-8.36829000	2.23534800
H	-0.93886300	-10.31240900	4.16704200
H	-2.42558200	-9.73626200	4.97238900
H	-2.46583200	-10.21664000	3.25442800
H	-2.60966100	-6.48826400	2.99026700
H	-3.48770000	-8.00579800	2.70609500
H	-3.35406300	-7.35810000	4.36522000
H	-1.49120000	-8.87199700	1.51871200
H	-0.59794100	-7.37183100	1.82746600
H	0.10976800	-8.94318700	2.29191200
C	5.45349400	-3.80088200	6.70802000
C	5.44629500	-4.73273500	7.92674100
C	6.86992100	-3.84092800	6.10508300
C	5.12671500	-2.38874400	7.20413900
H	4.49216700	-4.69001200	8.47262600
H	5.64320900	-5.77798200	7.64485600

H	6.23552900	-4.41297200	8.62215100
H	6.97623200	-3.12872200	5.27927100
H	7.60472800	-3.58596800	6.88373400
H	7.10363400	-4.85072700	5.73347200
H	5.90698200	-2.04799800	7.90246700
H	5.06518400	-1.66765800	6.38091300
H	4.16327700	-2.38335400	7.73690800
C	4.11118600	-4.12934200	1.78893100
C	3.46677900	-5.15658600	0.84991900
C	3.60236300	-2.74350100	1.37881300
C	5.63686900	-4.21469000	1.59921100
H	3.78134200	-6.18266500	1.08998200
H	2.36742800	-5.11193900	0.87925200
H	3.77761900	-4.93935500	-0.18194200
H	4.02129700	-1.96161000	2.02418100
H	3.88482000	-2.53276800	0.33482700
H	2.50241700	-2.71805700	1.44707800
H	5.88414900	-4.06483600	0.53735600
H	6.15713200	-3.45262900	2.19085000
H	6.00510000	-5.20776100	1.89838500
O	-2.26595100	-7.87489800	6.35661900
O	5.11019400	-2.59189500	4.06729900

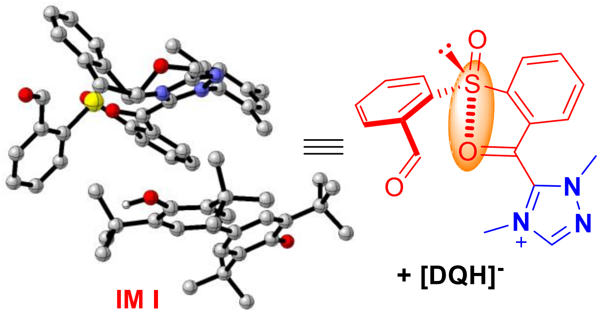

IM I

C	0.33858500	2.47916500	-5.02092400
C	-0.26992900	2.14444600	-3.69225000
C	1.70701200	3.06181700	-2.50290600
C	2.35296100	3.05404700	-3.90805000
H	0.69050900	1.53572200	-5.47639100
H	-0.42574400	2.91451400	-5.67522700
H	2.35558800	2.55758100	-1.77235000
H	2.82077000	2.08825100	-4.15853400
O	1.37538100	3.40311600	-4.86984900
N	-1.41897900	1.57511300	-3.44771300
N	0.42792300	2.33692700	-2.53733900
C	3.33094900	4.22592000	-3.82888100
H	4.27331100	3.90276300	-3.36081500
H	3.55509000	4.60927900	-4.83244900
C	1.59061700	4.54947200	-2.22191200
C	0.69810200	5.25399400	-1.42346300
C	2.58190800	5.20804300	-2.95740300
C	0.84093600	6.64061900	-1.32921900
H	-0.10070900	4.74883000	-0.87858600
C	2.73058800	6.58642000	-2.85195600
C	1.85731200	7.29764600	-2.02455600
H	3.50234600	7.10677600	-3.42159700
H	1.95789000	8.38039000	-1.93723000
C	-0.31456400	1.81459600	-1.54288400
N	-1.43970600	1.37933000	-2.11885100
C	-2.62503900	0.88462300	-1.46380900
C	-3.02126900	-0.43593400	-1.71555300
C	-3.29242600	1.73975300	-0.57921000
C	-4.10531900	-0.91722900	-0.98522500
C	-4.36268500	1.19741100	0.13638300
C	-4.76880600	-0.12824600	-0.03680500
H	-4.43266100	-1.94650600	-1.14814300
H	-4.89795000	1.83654400	0.84199500
C	-2.89507700	3.18118100	-0.39128300
H	-3.75255100	3.76095200	-0.02968000

H	-2.09911100	3.29878800	0.36308300
H	-2.54977600	3.63886100	-1.32922500
C	-2.27348200	-1.31350400	-2.67968200
H	-2.23304700	-0.86867400	-3.68368800
H	-1.23515300	-1.47495200	-2.34258900
H	-2.75316100	-2.29673400	-2.75401300
C	-5.88185600	-0.71052700	0.78883400
H	-5.46234900	-1.29862800	1.62016500
H	-6.51641800	0.07479200	1.21786000
H	-6.50737400	-1.38690200	0.19127700
H	0.15072800	7.21217000	-0.70726300
O	0.85555500	2.78380900	0.19786400
O	3.06055800	5.35539300	0.96776800
O	-1.13322100	3.43521500	4.01437300
S	-0.40392200	3.26673600	2.70995400
C	0.08082300	1.89703900	-0.08247800
C	-0.52521600	1.01669100	0.92774700
C	-0.76208200	1.53943600	2.21686500
C	-1.45162700	0.78773000	3.15660400
H	-1.69006400	1.24429900	4.11884000
C	-1.84566900	-0.51766500	2.84479600
H	-2.38133700	-1.10922000	3.58869400
C	-1.56298000	-1.06856000	1.59661100
H	-1.85134100	-2.09530300	1.36307600
C	-0.90358700	-0.29876300	0.63920700
H	-0.66378700	-0.73082700	-0.33578800
C	2.15211100	4.81990700	1.54933200
H	1.09731000	5.05714700	1.29900500
C	2.36896800	3.83063100	2.64761800
C	3.66759200	3.68215200	3.15126800
H	4.46008500	4.28637400	2.70725600
C	3.92853400	2.79579100	4.19004900
H	4.94322700	2.68927700	4.57536800
C	2.88770600	2.04492700	4.74309500
H	3.08561500	1.35080500	5.56095700
C	1.58960300	2.18407900	4.25687700
H	0.76895900	1.61319300	4.69603700
C	1.34051900	3.05996300	3.20302600
H	3.86040300	-0.11025900	-1.07029200
C	-0.45335000	-4.07459600	0.63389000
C	-1.62438700	-4.42589100	-0.06251600
C	-2.65947400	-5.15901200	0.50639100
C	-2.57772900	-5.58116900	1.89963200
C	-1.35588000	-5.22935200	2.61205000

C	-0.35841000	-4.51163800	1.96763300
C	0.57620100	-3.20452100	0.02960500
C	0.70844700	-3.06240000	-1.36048100
C	1.59634100	-2.16200700	-1.95529500
C	2.39532400	-1.36282300	-1.10477000
C	2.36408500	-1.52888900	0.29830000
C	1.44189300	-2.43769200	0.82284600
H	-1.73341100	-4.07509400	-1.09046200
H	0.55521000	-4.28160200	2.51604800
H	0.09944900	-3.69910200	-1.99888500
H	1.35838700	-2.52880400	1.90264200
C	-1.19845500	-5.66746500	4.07469500
C	0.14318400	-5.23338200	4.67563400
C	-2.31400300	-5.03469800	4.92616300
C	-1.27947600	-7.20106000	4.17447200
H	0.99557800	-5.67221500	4.13491500
H	0.25961600	-4.13846800	4.66864700
H	0.19921300	-5.57047000	5.72209800
H	-3.29503500	-5.31890000	4.52887800
H	-2.23350500	-5.36845900	5.97393800
H	-2.22627000	-3.93638500	4.91121500
H	-1.20218800	-7.52151800	5.22658600
H	-2.22986900	-7.55230700	3.75667500
H	-0.45024400	-7.66309800	3.61561500
C	-3.90779500	-5.53779900	-0.30218400
C	-4.06077000	-7.06960000	-0.33450800
C	-5.15566100	-4.90567800	0.33867100
C	-3.83524800	-5.05800500	-1.75493300
H	-3.20248900	-7.52565200	-0.85262600
H	-4.11076300	-7.46238500	0.68712000
H	-4.97678100	-7.35065200	-0.87987600
H	-5.07572100	-3.80645600	0.32212900
H	-6.06428800	-5.19186900	-0.21647100
H	-5.24507700	-5.23537100	1.37999400
H	-4.74263400	-5.37385800	-2.29176600
H	-3.77639300	-3.96124000	-1.81708100
H	-2.96442400	-5.47572300	-2.28307000
C	3.33190700	-0.77298200	1.22871200
C	3.09084000	-1.11925000	2.70439100
C	4.78443100	-1.19058500	0.91472700
C	3.14879500	0.75250700	1.12737800
H	2.07368600	-0.84103700	3.02081000
H	3.24195100	-2.18916900	2.90562300
H	3.80023900	-0.55138100	3.32443600

H	5.10982000	-0.98920200	-0.11689700
H	5.47902400	-0.66255100	1.58491300
H	4.90324400	-2.27153400	1.07680900
H	3.91886800	1.26257600	1.72506100
H	3.19406000	1.16019800	0.10781500
H	2.17007500	1.03385500	1.54183000
C	1.74242000	-2.09479600	-3.48535100
C	0.82671600	-3.10081800	-4.19530400
C	1.36198900	-0.69585500	-3.99620300
C	3.18834800	-2.43478600	-3.89196800
H	1.06247900	-4.13613600	-3.91069900
H	-0.23681300	-2.91751700	-3.98234700
H	0.97003600	-3.00878700	-5.28170200
H	1.96837300	0.08151700	-3.51408700
H	1.51578000	-0.64148100	-5.08594200
H	0.29591300	-0.50207600	-3.79518900
H	3.27765100	-2.42321100	-4.98928100
H	3.90671800	-1.72016000	-3.47562000
H	3.45149900	-3.44349800	-3.54013900
O	-3.51676500	-6.22167400	2.45973800
O	3.21579900	-0.44148600	-1.70289400

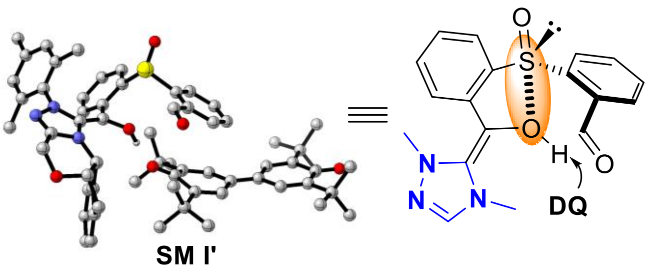

O	-1.99904300	0.48652900	-0.87440500
O	-0.39671200	2.01793600	-3.44816000
O	-1.46660600	-3.34911000	-3.20763000
S	-1.72403300	-2.03316400	-2.50574300
C	-3.13547200	-0.19491000	-0.46029000
C	-2.96236200	-1.58817100	-0.03933800
C	-2.24326100	-2.49839100	-0.83555800
C	-2.09928600	-3.83799100	-0.48807200
H	-1.59911700	-4.51049400	-1.18769300
C	-2.64615100	-4.29820300	0.70901600
H	-2.53777000	-5.34658300	0.99067500
C	-3.34201500	-3.41228300	1.53453400
H	-3.77665300	-3.76465600	2.47173600
C	-3.48564100	-2.07645800	1.17122600
H	-4.02360900	-1.38617400	1.82382800
C	-0.67693200	0.87251900	-3.19057500
H	-1.68195900	0.46609500	-3.41811000
C	0.30158100	-0.08204900	-2.58708400
C	1.63388000	0.32387900	-2.44539200
H	1.90738500	1.32568100	-2.78092000
C	2.58499800	-0.54112300	-1.91339800
H	3.62258000	-0.21826200	-1.81477700
C	2.21261200	-1.82681900	-1.51312200
H	2.95744300	-2.50570600	-1.09311600
C	0.89293200	-2.25118900	-1.66470500
H	0.59458200	-3.26348600	-1.38404100
C	-0.05587900	-1.37557400	-2.18335500
C	-6.36563800	3.41974300	-1.18542500
C	-5.87197700	2.03811500	-0.89681800
C	-3.56722900	2.89841400	-0.58913900
C	-4.17654400	4.25874300	-1.01069200
H	-6.39927000	3.57451000	-2.28016100
H	-7.37691700	3.54566200	-0.78297400
H	-2.66195600	2.70991000	-1.18395400
H	-4.12030200	4.41018400	-2.10235300
O	-5.51912800	4.36247900	-0.57959900

N	-6.56104300	0.96420300	-0.75121400
N	-4.51382300	1.81699000	-0.82046200
C	-3.37130800	5.27567200	-0.20150600
H	-2.43759800	5.52643600	-0.72938800
H	-3.94554700	6.20040000	-0.05803000
C	-3.20469700	3.14670300	0.86631300
C	-3.02440000	2.24580400	1.91308200
C	-3.08921100	4.52602100	1.08006700
C	-2.74202500	2.74803400	3.18823000
H	-3.11375300	1.17021300	1.75250300
C	-2.79554800	5.02233700	2.34582600
C	-2.62643300	4.12232900	3.40258000
H	-2.71815300	6.09791200	2.51685700
H	-2.40907700	4.49785600	4.40388200
C	-4.33344800	0.45733100	-0.57170700
N	-5.63135500	-0.03909600	-0.58355400
C	-6.04729700	-1.37626200	-0.33685400
C	-5.75871000	-2.38660300	-1.26748800
C	-6.68874700	-1.65749200	0.88054800
C	-6.08994400	-3.70117000	-0.92983900
C	-7.02902600	-2.98196500	1.16011700
C	-6.71882800	-4.02005900	0.27639400
H	-5.84758800	-4.50037400	-1.63575600
H	-7.52392500	-3.21204600	2.10718300
C	-6.95937700	-0.55651900	1.87007700
H	-7.29048200	-0.96849000	2.83134300
H	-6.05540500	0.05101900	2.03772100
H	-7.73026000	0.13176500	1.49507500
C	-5.12915500	-2.09142000	-2.60357000
H	-4.55238100	-1.15861000	-2.59604500
H	-4.45844300	-2.90988500	-2.90459800
H	-5.90779200	-1.99871400	-3.37693200
C	-7.01414300	-5.45408300	0.62886500
H	-7.36186900	-6.01672900	-0.24823800
H	-6.10349600	-5.95213400	0.99752600
H	-7.77683100	-5.52484700	1.41516300
H	-2.60919500	2.05614800	4.02167900
H	-1.34885100	0.51646700	-0.14611800
C	5.29303100	0.10193700	0.62148500
C	5.95060300	-1.16311800	0.90635300
C	7.21185400	-1.45744300	0.51468600
C	7.98974800	-0.43021800	-0.24716300
C	7.32204700	0.87066400	-0.56888700
C	6.05623300	1.07900500	-0.13815500

C	3.98326000	0.34583500	1.01183700
C	3.20563100	-0.63648600	1.75196700
C	1.86872900	-0.53517900	1.93164600
C	1.16225400	0.59934300	1.26915500
C	1.97822800	1.78338800	0.86640700
C	3.31068600	1.60217800	0.71436100
H	5.39209800	-1.91479700	1.45462600
H	5.57709900	2.02243600	-0.37966100
H	3.72530500	-1.48526600	2.18677800
H	3.91276900	2.43891300	0.37263500
C	8.09820400	1.91680100	-1.36950300
C	7.25452600	3.17236600	-1.61567100
C	9.35777000	2.34150100	-0.59067700
C	8.49292300	1.33891400	-2.74189300
H	6.34487000	2.94759500	-2.19248800
H	6.96130800	3.66012600	-0.67406700
H	7.84781600	3.89394700	-2.19468400
H	10.03621700	1.49607400	-0.43199900
H	9.89198700	3.11809000	-1.15882400
H	9.08144900	2.76504200	0.38681300
H	9.01638700	2.11177200	-3.32482300
H	9.15497800	0.47222500	-2.63788700
H	7.59679400	1.03729100	-3.30523700
C	7.87347300	-2.80091900	0.82367900
C	8.25999100	-3.50849400	-0.48895500
C	9.12438900	-2.57934700	1.69519300
C	6.92552600	-3.72783500	1.59267000
H	7.36934800	-3.67524900	-1.11373700
H	8.99002000	-2.92520700	-1.06085100
H	8.70044500	-4.48996200	-0.25687800
H	8.85303500	-2.08199200	2.63871100
H	9.57346400	-3.55351900	1.94136800
H	9.87310600	-1.97091000	1.17612700
H	7.43846700	-4.67953600	1.79035200
H	6.63038200	-3.29952400	2.56217900
H	6.01531200	-3.95180700	1.01625500
C	1.29715700	3.13957200	0.68221700
C	2.32663000	4.23834100	0.39831200
C	0.28943300	3.12735000	-0.47866500
C	0.56536800	3.48807500	1.98964600
H	3.07204100	4.31899300	1.20347900
H	2.85322700	4.06863700	-0.55292600
H	1.80660500	5.20362200	0.32211800
H	-0.52583600	2.41632200	-0.30505300

H	-0.15245800	4.13237000	-0.57721400
H	0.77386000	2.87676100	-1.43247700
H	0.09263600	4.47763400	1.89515100
H	-0.22858200	2.76232900	2.20781500
H	1.26750000	3.51957900	2.83729200
C	1.05927900	-1.54973600	2.73842500
C	1.98091000	-2.46240300	3.55587900
C	0.12842100	-0.81551300	3.72218700
C	0.23076800	-2.43224900	1.79012700
H	2.61370900	-3.09158500	2.91289200
H	2.63109500	-1.88471900	4.23018800
H	1.36588100	-3.13645400	4.16874000
H	-0.38636300	-1.55466900	4.35389300
H	0.70498700	-0.14759100	4.38106700
H	-0.63037500	-0.22343800	3.19637000
H	-0.37755200	-3.13752300	2.37741400
H	-0.44359500	-1.83471800	1.16354900
H	0.89390700	-3.01561300	1.13344600
O	9.13879900	-0.64753500	-0.59755100
O	-0.04898700	0.56067600	1.08589700

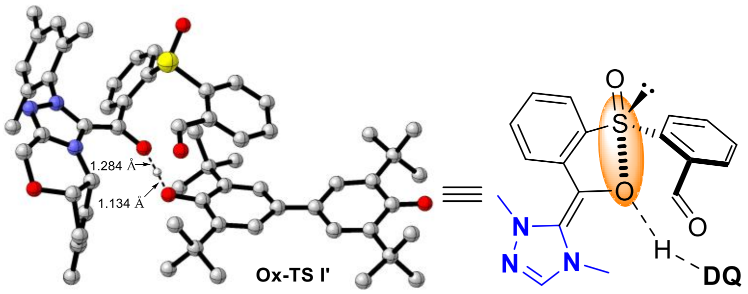

O	-0.71745600	0.15469800	0.39818400
O	0.13599200	-0.64714600	3.47352600
O	2.36093700	-3.36143800	-0.84742900
S	1.58897100	-2.16690500	-0.33273400
C	0.08644200	0.56786200	-0.54051900
C	0.20351800	-0.21264500	-1.80293900
C	0.74372500	-1.50802800	-1.79961800
C	0.85262100	-2.25981500	-2.96394700
H	1.33859100	-3.23586900	-2.91512200
C	0.38529700	-1.72969600	-4.16675000
H	0.46887100	-2.31239700	-5.08526800
C	-0.16952600	-0.45030500	-4.19366200
H	-0.52814700	-0.02697300	-5.13317600
C	-0.26598900	0.29568100	-3.02045900
H	-0.70658900	1.29443200	-3.04266100
C	0.43773400	-1.11034300	2.40037100
H	1.25915800	-0.66159800	1.80405800
C	-0.20640000	-2.32487800	1.82054000
C	-1.21828700	-2.96819200	2.54447600
H	-1.53159500	-2.53417200	3.49562900
C	-1.78820500	-4.14461300	2.06669000
H	-2.57487800	-4.64060300	2.63753200
C	-1.34218200	-4.70022600	0.86402600
H	-1.77962200	-5.63073100	0.49769600
C	-0.33436100	-4.07359100	0.13347400
H	0.04611600	-4.50608100	-0.79437900
C	0.20550600	-2.87914100	0.60081700
C	2.34553600	4.28366200	1.79367600
C	2.00477800	3.33999200	0.68403100
C	-0.02962000	2.59720200	1.89119400
C	0.50454300	3.50690900	3.02692400
H	3.08444600	3.80294600	2.46137300
H	2.79139700	5.19466000	1.37889900
H	-0.23789600	1.59647500	2.29508500
H	1.16833200	2.95475600	3.71349100
O	1.18237000	4.62681700	2.49329200

N	2.62387700	3.18048200	-0.43956100
N	0.95426600	2.47237200	0.81909000
C	-0.76873400	4.03806300	3.68620100
H	-1.12885800	3.32717600	4.44646700
H	-0.57200100	4.99855200	4.18011400
C	-1.30575900	3.30474900	1.47908000
C	-2.01377800	3.23304300	0.28493100
C	-1.72986200	4.13280800	2.52417100
C	-3.15918400	4.02214000	0.14016000
H	-1.69024600	2.58066500	-0.52810800
C	-2.87775800	4.90568200	2.38410100
C	-3.58767200	4.84805400	1.18061200
H	-3.20958900	5.56050100	3.19202500
H	-4.48317900	5.45808300	1.05191200
C	0.91812900	1.69285000	-0.31867400
N	1.96938800	2.16864000	-1.06905100
C	2.31733800	1.85380800	-2.42161100
C	3.13895600	0.75651200	-2.70011400
C	1.79600000	2.68171900	-3.42776200
C	3.37542600	0.45483100	-4.04475600
C	2.07656100	2.35191500	-4.75231400
C	2.84424300	1.22750900	-5.07940600
H	3.99420800	-0.41363800	-4.28553200
H	1.67171200	2.97821800	-5.55102700
C	0.94635500	3.87142900	-3.07119800
H	0.55267200	4.35422500	-3.97351300
H	0.09498100	3.58063300	-2.43474100
H	1.52902100	4.61178900	-2.50333600
C	3.77493400	-0.06910300	-1.61520600
H	3.34928900	0.14433300	-0.62652500
H	3.66382500	-1.14462300	-1.81881100
H	4.85358600	0.14442400	-1.56733000
C	3.06257300	0.83584500	-6.51603000
H	4.04709900	0.37106900	-6.65820900
H	2.30280600	0.10098900	-6.82673400
H	2.98141200	1.70235700	-7.18493000
H	-3.72468800	3.98610900	-0.79211300
H	-1.99154500	0.09680900	0.25062600
C	-5.42708800	-4.42912200	2.51061000
C	-5.41066900	-5.67784900	1.80351400
C	-5.76064600	-6.86991700	2.36795600
C	-6.20453000	-6.89407200	3.78519400
C	-6.23774300	-5.60506800	4.52210500
C	-5.85360100	-4.46396500	3.88015900

C	-4.98929500	-3.22306900	1.88887900
C	-4.73505000	-3.14706400	0.48815600
C	-4.17931300	-2.04701200	-0.12053600
C	-3.78416400	-0.92215600	0.70487100
C	-4.23168100	-0.88464200	2.08325800
C	-4.78346600	-2.02140900	2.62742900
H	-5.06597300	-5.67424300	0.77286000
H	-5.89351200	-3.52623600	4.42704200
H	-5.02454900	-3.98907600	-0.13261300
H	-5.06279800	-2.00387100	3.67576000
C	-6.70469200	-5.59914800	5.97910900
C	-6.68281800	-4.18738800	6.57577200
C	-8.15049200	-6.12284400	6.06887100
C	-5.77201500	-6.48131100	6.83056300
H	-5.66987000	-3.75751500	6.57395500
H	-7.35287600	-3.50327400	6.03384800
H	-7.02462500	-4.23374800	7.61974300
H	-8.22215400	-7.15237700	5.70124100
H	-8.48719600	-6.09701700	7.11683200
H	-8.82679400	-5.48590000	5.47843900
H	-6.10003200	-6.45711300	7.88133500
H	-5.78234800	-7.51984100	6.48202400
H	-4.73969300	-6.10152000	6.78767400
C	-5.70085200	-8.18557800	1.58969000
C	-4.71648700	-9.15370300	2.27260100
C	-7.10341000	-8.81966000	1.52535500
C	-5.22050600	-7.97121900	0.14996900
H	-3.70682100	-8.71584900	2.30308400
H	-5.03342400	-9.38465100	3.29562800
H	-4.66341500	-10.09158800	1.69831800
H	-7.80873400	-8.14088900	1.02189400
H	-7.05842400	-9.75481800	0.94594300
H	-7.48226800	-9.04416200	2.52847400
H	-5.19873900	-8.94008800	-0.36934100
H	-5.89283300	-7.30486600	-0.41104800
H	-4.20412800	-7.55050100	0.11538300
C	-4.11611500	0.39516100	2.92001200
C	-4.81184500	0.25888900	4.28024700
C	-2.64078800	0.70752600	3.18320100
C	-4.78935200	1.56026900	2.17453700
H	-5.87920500	0.01375100	4.17382000
H	-4.33755100	-0.50492400	4.91391200
H	-4.73699000	1.21888900	4.81109200
H	-2.08165400	0.78441700	2.24491700

H	-2.55284500	1.66651700	3.71880900
H	-2.17715400	-0.07755100	3.79642300
H	-4.71357500	2.47654400	2.78086600
H	-4.30722200	1.75124500	1.20985700
H	-5.85637100	1.34470000	2.00737700
C	-3.98278200	-2.02335000	-1.64184000
C	-4.72625100	-3.17266400	-2.33539600
C	-4.51578500	-0.71188800	-2.24803200
C	-2.48775900	-2.18404100	-1.93078100
H	-4.33966900	-4.15814100	-2.03798600
H	-5.80722600	-3.14368400	-2.13094500
H	-4.58260200	-3.08154400	-3.42167400
H	-4.43435500	-0.76117800	-3.34453300
H	-5.57783900	-0.57247100	-1.99268300
H	-3.95481600	0.15874900	-1.89355400
H	-2.27275600	-2.02339700	-2.99889600
H	-1.90065600	-1.46747500	-1.34763700
H	-2.15419700	-3.19521800	-1.65570800
O	-6.53339200	-7.94936100	4.33147200
O	-3.12328900	0.07978700	0.18984600

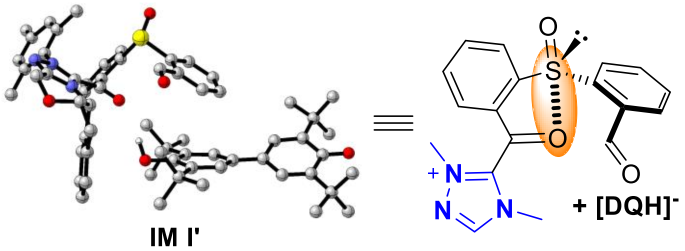

IM I'

O	1.29880800	1.53044700	-0.52861900
O	1.93299300	0.47297800	2.54561000
O	4.16143300	-2.19414300	-1.81357900
S	3.40485500	-1.01726900	-1.25545700
C	2.02965900	1.80303600	-1.45046600
C	2.07220600	1.00730400	-2.71132200
C	2.55809300	-0.30508600	-2.70495800
C	2.58766300	-1.05187400	-3.87658800
H	3.04050500	-2.04482000	-3.85513100
C	2.07327900	-0.50414400	-5.05218600
H	2.08746700	-1.09103400	-5.97130300
C	1.56048200	0.79313600	-5.06020300
H	1.16554700	1.22335600	-5.98099300
C	1.56626200	1.55371200	-3.89306800
H	1.17082100	2.57173700	-3.90100600
C	2.29661500	-0.05304500	1.52298000
H	3.22517100	0.29333700	1.01825200
C	1.58557800	-1.18673500	0.87297300
C	0.50762000	-1.78418300	1.53974300
H	0.18810600	-1.36759000	2.49670300
C	-0.11852300	-2.90402600	1.00108400
H	-0.94947200	-3.37102200	1.53210300
C	0.32333600	-3.43537200	-0.21467900
H	-0.16098800	-4.32090100	-0.63021900
C	1.38292600	-2.84419200	-0.90066900
H	1.74455500	-3.25729700	-1.84453100
C	1.99134700	-1.71404800	-0.36259900
C	4.37760500	5.59310100	0.76557300
C	4.01742800	4.62189200	-0.31759500
C	2.12580400	3.74215000	1.03983000
C	2.75094200	4.61539600	2.15290600
H	5.24129100	5.18996100	1.32497600
H	4.67054900	6.54783700	0.31384700
H	2.01785600	2.70718600	1.39445200
H	3.53588300	4.07666600	2.70860600
O	3.27280800	5.80380000	1.59352800

N	4.59424000	4.44765500	-1.47895400
N	2.99754100	3.73587800	-0.14961800
C	1.53830100	4.99909900	2.99991600
H	1.33127700	4.20866100	3.73838000
H	1.72559500	5.93673200	3.53872800
C	0.77571700	4.39074300	0.81659800
C	-0.07784200	4.33988700	-0.27945400
C	0.42893400	5.08791200	1.97846100
C	-1.31097700	4.99114400	-0.19199800
H	0.18911700	3.81804200	-1.20129000
C	-0.80207400	5.72791300	2.06563700
C	-1.67155300	5.67062700	0.97277700
H	-1.08222900	6.27273500	2.96877900
H	-2.64108400	6.16775300	1.02764000
C	2.95050600	2.98464600	-1.26820000
N	3.93422900	3.43784800	-2.05429500
C	4.25077600	3.04618100	-3.40931700
C	5.00565900	1.89282500	-3.64051700
C	3.73997200	3.86232100	-4.42841400
C	5.18403700	1.51927800	-4.97528900
C	3.96458100	3.44986500	-5.74003100
C	4.66175800	2.27051100	-6.03087500
H	5.74991100	0.60956600	-5.19015500
H	3.56944100	4.05735600	-6.55711300
C	2.96974700	5.11667300	-4.11462400
H	2.55102000	5.54827300	-5.03068200
H	2.13713500	4.92537900	-3.41830400
H	3.62066700	5.86817800	-3.64433500
C	5.63576000	1.08269900	-2.54084100
H	5.25119300	1.33269300	-1.54349100
H	5.48940600	0.00620400	-2.71253000
H	6.72073000	1.26439400	-2.52545900
C	4.81481500	1.79872000	-7.45020200
H	5.77596400	1.29017900	-7.59958400
H	4.01778900	1.07756000	-7.69147500
H	4.73898100	2.63183100	-8.16009900
H	-1.99707600	4.96106500	-1.03900300
H	-0.86500700	1.41220500	-0.23818600
C	-3.78047900	-3.37311200	2.02340300
C	-3.43719100	-4.60307500	1.43392500
C	-3.81864800	-5.83337100	1.95452000
C	-4.62970600	-5.89364600	3.16327200
C	-4.98223600	-4.61723900	3.77230400
C	-4.55293900	-3.42854900	3.19699000

C	-3.32670400	-2.08951500	1.44770100
C	-3.08265900	-1.95272800	0.07335600
C	-2.57658900	-0.77917600	-0.49038200
C	-2.32274400	0.30975000	0.36989200
C	-2.61637100	0.24634000	1.74893900
C	-3.09565100	-0.96623700	2.25506800
H	-2.81666300	-4.57887300	0.53563500
H	-4.84817200	-2.48252100	3.65355600
H	-3.30439400	-2.80315600	-0.56832200
H	-3.27744500	-1.06274300	3.32360200
C	-5.83343700	-4.61631800	5.05006200
C	-6.11934000	-3.20079400	5.56415700
C	-7.18867300	-5.29340200	4.77876100
C	-5.09077500	-5.37352400	6.16553000
H	-5.19284400	-2.65853200	5.80798100
H	-6.68225000	-2.60438500	4.83005900
H	-6.72435700	-3.26239000	6.48195600
H	-7.03028400	-6.31380200	4.41172800
H	-7.79287100	-5.32615600	5.70064100
H	-7.75241300	-4.72490100	4.02212600
H	-5.70875200	-5.41791900	7.07774500
H	-4.85859900	-6.39167300	5.83324500
H	-4.15115800	-4.85561900	6.41535200
C	-3.38559200	-7.14588400	1.28612400
C	-2.55618500	-7.98458500	2.27496800
C	-4.62756400	-7.94369300	0.85010000
C	-2.52166300	-6.91359700	0.04141500
H	-1.63437400	-7.44731500	2.54968300
H	-3.14126700	-8.17407400	3.18208500
H	-2.26812900	-8.94562800	1.81741000
H	-5.20046400	-7.37685700	0.09904600
H	-4.32872000	-8.90416500	0.39817300
H	-5.26902800	-8.13433200	1.71828700
H	-2.24486800	-7.88487200	-0.39680500
H	-3.05652000	-6.33693700	-0.72893100
H	-1.59050800	-6.37955700	0.28731700
C	-2.44308800	1.46969400	2.66646200
C	-2.85451200	1.15749800	4.11122200
C	-0.97646700	1.92821100	2.70506400
C	-3.34078400	2.61589500	2.16774500
H	-3.90586000	0.84196000	4.17897600
H	-2.22704300	0.37022800	4.55446700
H	-2.73502500	2.06517700	4.72082300
H	-0.62639400	2.26228300	1.71978600

H	-0.87563600	2.78064700	3.39731000
H	-0.31879500	1.12019300	3.05574100
H	-3.23478700	3.48774700	2.83346300
H	-3.06320900	2.92330000	1.15235100
H	-4.39705800	2.30563600	2.17325700
C	-2.34288000	-0.67145700	-2.00731400
C	-2.55798600	-2.01942200	-2.70775600
C	-3.33592500	0.34029500	-2.60417600
C	-0.90117700	-0.22325600	-2.31428900
H	-1.86560900	-2.78602700	-2.32658300
H	-3.58574800	-2.38867400	-2.58568700
H	-2.37323100	-1.89836200	-3.78507600
H	-3.16047100	0.44886400	-3.68648600
H	-4.37060800	-0.00362100	-2.45719100
H	-3.22083000	1.32304500	-2.12882000
H	-0.63347200	-0.47512400	-3.35346300
H	-0.77986700	0.86129200	-2.20317900
H	-0.18016900	-0.72408900	-1.64928900
O	-5.00545200	-6.99896900	3.66109100
O	-1.82678300	1.48928700	-0.15958800
